# A metal-catalyzed new approach for α-alkynylation of cyclic amines†
†Electronic supplementary information (ESI) available. See DOI: 10.1039/c8sc04115f


**DOI:** 10.1039/c8sc04115f

**Published:** 2018-11-26

**Authors:** Yifan Cui, Weilong Lin, Shengming Ma

**Affiliations:** a State Key Laboratory of Organometallic Chemistry , Shanghai Institute of Organic Chemistry , Chinese Academy of Sciences , 345 Lingling Lu , Shanghai 200032 , P. R. China . Email: masm@sioc.ac.cn; b University of Chinese Academy of Sciences , Beijing 100049 , P. R. China; c Department of Chemistry , Fudan University , 220 Handan Lu , Shanghai 200433 , P. R. China

## Abstract

The first catalytic α-alkynylation of cyclic amines with the help of the *N*-propargylic group to afford 2-(1-alkynyl) *N*-allylic cyclic amines with an exclusive *E*-stereoselectivity for the *in situ* formed C=C bond has been realized. Based on mechanistic studies, it is proven that the reaction proceeds through metal-mediated *anti*-1,5-hydride transfer forming an iminonium intermediate, which accepts the addition of the *in situ* generated 1-alkynyl metal species. The synthetic application has also been demonstrated.

## Abstract 2

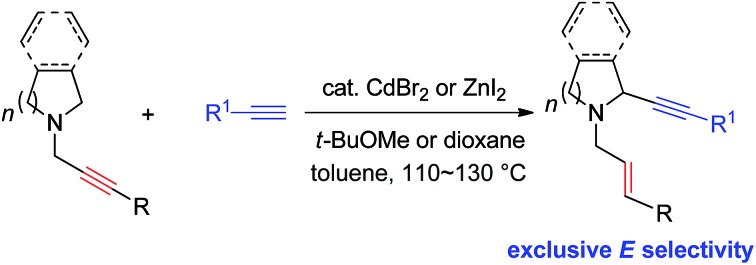
The first catalytic α-alkynylation of cyclic amines with the help of the *N*-propargylic group with an exclusive high *E*-stereoselectivity has been realized.

## Introduction

**Scheme 1:**
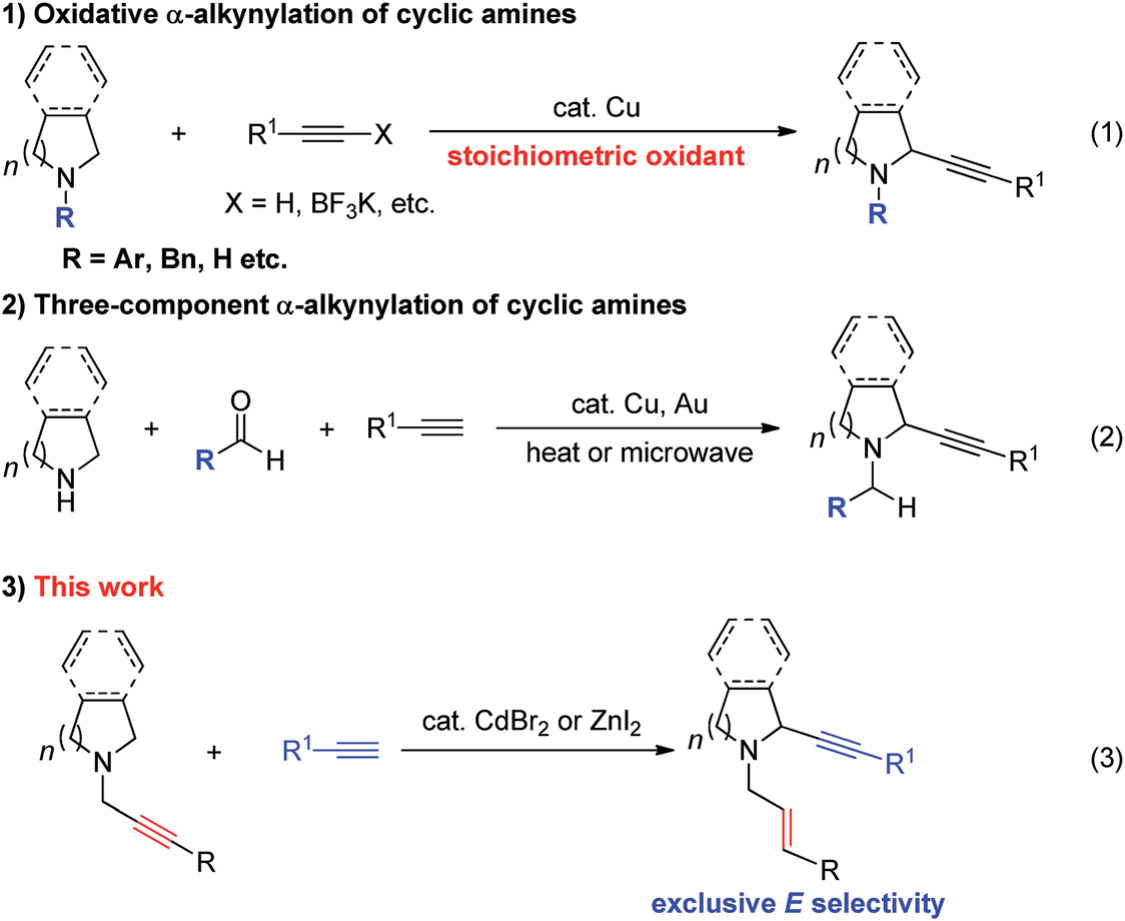
Different approaches for α-alkynylation of cyclic amines.

Due to the synthetic^1^ and bio-importance^2^–^7^ of cyclic amines much attention has been focused on the development of the related methodologies. One straightforward approach is α-functionalization of readily available cyclic amines.^8^ Oxidative coupling of *N*-protected cyclic amines with terminal alkynes or 1-alkynyl trifluoroborate in the presence of a stoichiometric amount of an oxidant (eqn (1)) and the three-component reaction of *N*-non-protected cyclic amines with terminal alkynes and aldehydes have been well established (eqn (2)).^9^,^10^ Starting from 2010, we have reported the ZnX_2_,^11^,^12^ CuI,^13^ or CuBr_2_ 14-mediated allenylation of terminal alkynes (ATA) reaction with aldehydes in the presence of different amines forming allenes. In this reaction, the second step is the metal-mediated 1,5-H transfer reaction of propargylic amines *in situ* formed in the first step, which was proven to be non-stereoselective by Nakamura *et al.* affording allylic propargylic amines with an *E*/*Z* ratio of 58/42–63/37 with acyclic amine.^15^ Herein, we wish to report a highly stereoselective *N*-propargylic cyclic amine-based α-alkynylation providing stereodefined *N*-(*E*)-allylic 2-alkynyl cyclic amines by using CdBr_2_ (or ZnI_2_) as the catalyst (Scheme 1).

## Results and discussion

### Optimization of the reaction

When we studied the mechanism of the Cu-catalyzed allenylation of terminal alkynes in the presence of an amine,^13^ it was observed that the reaction between *N*-alkynylic amine **1a** and phenylacetylene **2a** under CuBr catalysis provided a new product **3aa** in a low yield of 13% with 64% of starting material **1a** being recovered as judged by ^1^H NMR analysis. This new product was identified as α-alkynylated cyclic amine with an *N*-allylic group bearing an exclusive *E* C=C bond (Table 1, entry 1). Due to the importance of cyclic amines, we further optimized the reaction conditions by screening a variety of metal salts such as CuX_2_, ZnX_2_, AgOTf and CdX_2_, and CdBr_2_ 16 turned out to be the best providing the product **3aa** in 42% yield and 52% recovery of **1a** (Table 1, entries 2–7). On increasing the temperature to 120 °C, the yield was improved to 56% with 20% recovery (Table 1, entry 8).

**Table 1:** Optimization of catalytic α-alkynylation of 1-(2-alkynyl) cyclic amine **1a** with **2a**^*a*^

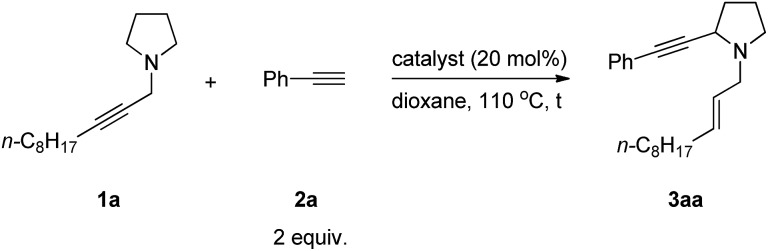				
Entry	Catalyst	Time (h)	Yield of **3aa**^*b*^ (%)	Recovery of **1a**^*b*^ (%)
1	CuBr	12	13	64
2	CuBr_2_	10	13	—
3	ZnCl_2_	10	24	—
4	ZnBr_2_	10	39	—
5	AgOTf	12	5	95
6	CdI_2_	10	40	—
7	CdBr_2_	24	42	53
8^*c*^	CdBr_2_	36	56	20
9^*d*^	CdBr_2_	12	47	26

^*a*^The reaction was conducted using **1a** (1.0 mmol) and alkyne **2a** (2.0 mmol) in 6 mL of dioxane at 110 °C.

^*b*^Determined by ^1^H NMR analysis with CH_2_Br_2_ as the internal standard.

^*c*^The reaction was conducted at 120 °C.

^*d*^The reaction was conducted at 130 °C.

### Effect of solvents

Then solvents were screened: when ^*t*^BuOMe was used as the solvent, the desired product **3aa** could be obtained in 63% yield with complete consumption of **1a** (Table 2, entries 1–7). In addition, reducing the catalyst loading to 10 mol% improved the yield slightly to 66% (Table 2, entries 8–9). Further reducing the catalyst loading resulted in the recovery of **1a** (Table 2, entry 10). Thus, **1a** (1 equiv.), **2a** (2 equiv.), and CdBr_2_ (10 mol%) in ^*t*^BuOMe at 120 °C were defined as the optimized reaction conditions for further study of this reaction.

**Table 2:** Optimization of reaction conditions for catalytic α-alkynylation of *N*-internal 2-alkynylic cyclic amine **1a** with **2a**^*a*^

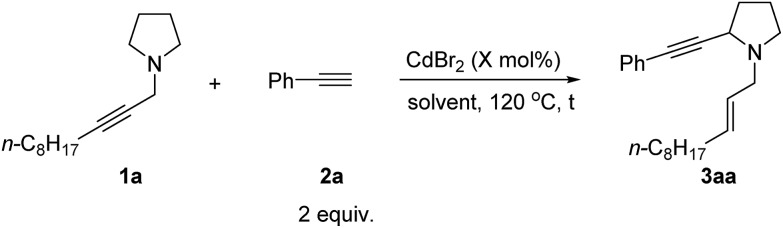					
Entry	*X*	Solvent	*t* (h)	Yield of **3aa**^*b*^ (%)	Recovery of **1b**^*b*^ (%)
1	20	DMF	23	43	25
2	20	DMSO	23	20	35
3	20	Toluene	23	40	—
4	20	THF	23	48	—
5	20	DCE	23	3	—
6	20	CH_3_CN	23	39	—
7	20	^*t*^BuOMe	36	63	—
8	15	^*t*^BuOMe	36	64	—
**9** ^*c*^	**10**	^***t***^ **BuOMe**	**36**	**66**	**—**
10	5	^*t*^BuOMe	36	69	10

^*a*^The reaction was conducted using **1a** (0.5 mmol) and alkyne **2a** (1.0 mmol) in 3 mL of solvent.

^*b*^Determined by ^1^H NMR analysis with CH_2_Br_2_ as the internal standard.

^*c*^The reaction was conducted using **1a** (1.0 mmol) and alkyne **2a** (2.0 mmol) in 6 mL of ^*t*^BuOMe at 120 °C.

### Substrate scope

With the optimal reaction conditions in hand, diversified terminal alkynes were investigated to examine the scope of this α-alkynylation reaction with amine **1a**. Terminal aryl acetylenes bearing electron-donating *p*-Me and *p*-MeO, and electron-withdrawing and synthetically attractive *p*-F, *p*-Cl, *m*-Cl, *p*-NO_2_, *p*-EtOOC, *p*-CN and *p*-Ac groups on the aryl ring could all afford the corresponding product **3** in moderate yields (Table 3, entries 1–10). In addition, alkyl-substituted terminal alkynes, such as 1-decyne (**2k**) and cyclohexylacetylene (**2l**), were found to be sluggish affording the corresponding products in 31% and 40% yields, respectively (Table 3, entries 11 and 12). Interestingly, trimethylsilylacetylene could react with **1a** to furnish **3am** in 76% yield (Table 3, entry 13). Other substituted propargylic amines, such as **1b**, **1c**, **1d** and **1g** could also react smoothly to afford the desired products **3ba**, **3ca**, **3da** and **3ga** in 40–65% yields (Table 3, entries 14–16, 21). *N*-Terminal propargylic amine **1e** was next exposed to the optimized conditions with arylacetylenes substituted with different functional groups, such as electron-donating *p*-MeO and electron-withdrawing *p*-F and *p*-Cl, affording the corresponding products **3ea–3ee** in moderate yields with 30 mol% of ZnI_2_ (Table 3, entries 17–20).

**Table 3:** The scope of catalytic α-alkynylation of *N*-internal 2-alkynylic cyclic amines^*a*^

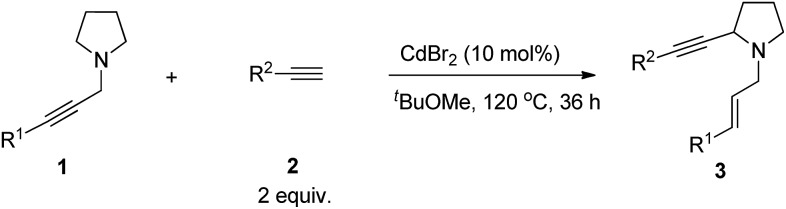			
Entry	**1** (*R*^1^)	**2** (*R*^2^)	Isolated yield of **3**^*b*^ (%)
1	*n*-C_8_H_17_ (**1a**)	C_6_H_5_ (**2a**)	63 (**3aa**)
2	*n*-C_8_H_17_ (**1a**)	*p*-MeC_6_H_4_ (**2b**)	51 (**3ab**)
3^*c*^	*n*-C_8_H_17_ (**1a**)	*p*-MeOC_6_H_4_ (**2c**)	45 (**3ac**)
4	*n*-C_8_H_17_ (**1a**)	*p*-FC_6_H_4_ (**2d**)	55 (**3ad**)
5	*n*-C_8_H_17_ (**1a**)	*p*-ClC_6_H_4_ (**2e**)	67 (**3ae**)
6	*n*-C_8_H_17_ (**1a**)	*m*-ClC_6_H_4_ (**2f**)	66 (**3af**)
7	*n*-C_8_H_17_ (**1a**)	*p*-O_2_NC_6_H_4_ (**2g**)	60 (**3ag**)
8	*n*-C_8_H_17_ (**1a**)	*p*-EtOOCC_6_H_4_ (**2h**)	60 (**3ah**)
9	*n*-C_8_H_17_ (**1a**)	*p*-NCC_6_H_4_ (**2i**)	61 (**3ai**)
10	*n*-C_8_H_17_ (**1a**)	*p*-AcC_6_H_4_ (**2j**)	59 (**3aj**)
11^*d*^	*n*-C_8_H_17_ (**1a**)	*n*-C_8_H_17_ (**2k**)	31 (**3ak**)
12^*e*^	*n*-C_8_H_17_ (**1a**)	Cy (**2l**)	40 (**3al**)
13^*f*^	*n*-C_8_H_17_ (**1a**)	TMS (**2m**)	76 (**3am**)
14	Cy (**1b**)	C_6_H_5_ (**2a**)	65 (**3ba**)
15^*g*^	*n*-C_4_H_9_ (**1c**)	C_6_H_5_ (**2a**)	57 (**3ca**)
16^*h*^	(CH_3_)_2_(OH)C (**1d**)	C_6_H_5_ (**2a**)	62 (**3da**)
17^*i*^	H (**1e**)	C_6_H_5_ (**2a**)	47 (**3ea**)
18^*i*^	H (**1e**)	*p*-MeOC_6_H_4_ (**2c**)	45 (**3ec**)
19^*i*^	H (**1e**)	*p*-FC_6_H_4_ (**2d**)	48 (**3ed**)
20^*i*^	H (**1e**)	*p*-ClC_6_H_4_ (**2e**)	46 (**3ee**)
21^*j*^	Ph (**1g**)	C_6_H_5_ (**2a**)	40 (**3ga**)

^*a*^The reaction was conducted using **1** (1.0 mmol) and 1-alkyne **2** (2.0 mmol) in 6 mL of MTBE at 120 °C for 36 h.

^*b*^ *E*/*Z* > 20 : 1, if any.

^*c*^22% of **1a** was recovered.

^*d*^20% of CdBr_2_ was used and 27% of **1a** was recovered.

^*e*^50% of **1a** was recovered.

^*f*^The reaction was conducted at 130 °C and 3% of **1a** was recovered.

^*g*^15% of CdBr_2_ was used.

^*h*^The reaction was conducted at 130 °C and 4% of **1d** was recovered.

^*i*^The reaction was conducted using **1e** (1.0 mmol), alkyne **2** (2.0 mmol) and ZnI_2_ (0.3 mmol) in 6 mL of dioxane at 110 °C for 10 h.

^*j*^The reaction was conducted in 6 mL of toluene and 25% of **1g** was recovered.

**Scheme 2:**
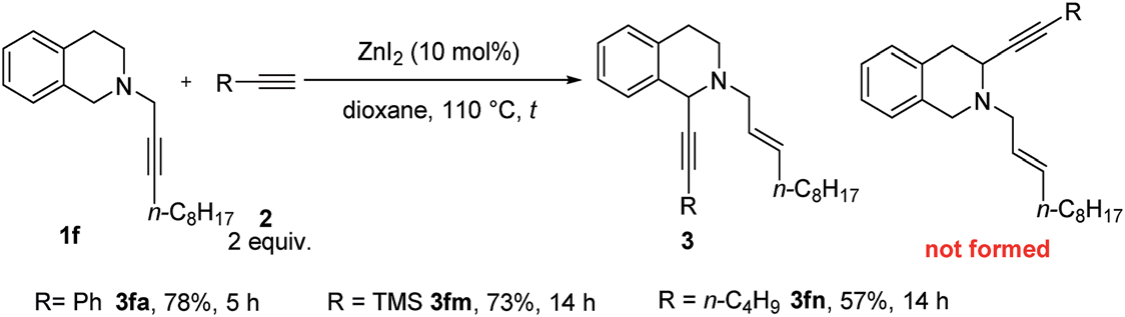
The scope of catalytic α-alkynylation of tetrahydroisoquinoline **1f**. The reaction was conducted using **1f** (1.0 mmol) and alkyne **2** (2.0 mmol) in 6 mL of dioxane at 110 °C.

Tetrahydroisoquinoline is the core skeleton of a variety of natural bio-active compounds and drugs.^17^ We first applied CdBr_2_ in ^*t*^BuOMe to *N*-propargylic tetrahydroisoquinoline derivative **1f** and phenylacetylene **2a**. The 1-alkynated product was obtained exclusively in 57% isolated yield with 24% **1f** recovery. Interestingly, using 10 mol% ZnI_2_ as the catalyst and dioxane as the solvent, the reaction afforded **3fa** in a yield of 78%. Trimethylsilylacetylene (**2m**) and 1-hexyne (**2n**) are also compatible (Scheme 2).

**Scheme 3:**
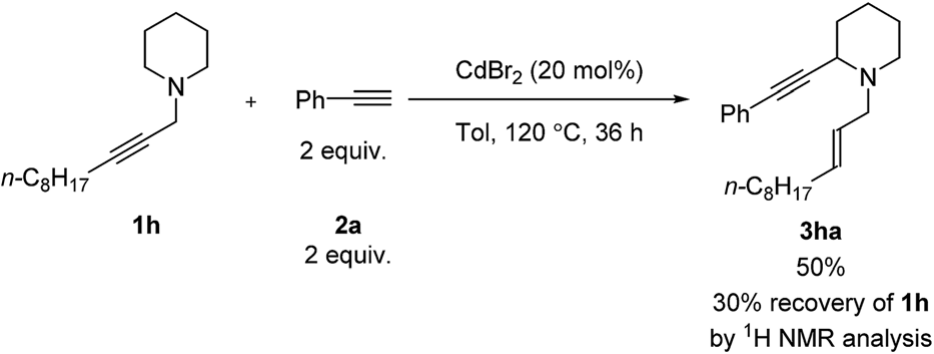
Catalytic α-alkynylation of piperidine **1h**. The reaction was conducted using **1h** (1.0 mmol) and phenylacetylene **2a** (2.0 mmol) in 6 mL of toluene at 120 °C.

For piperidine derivative **1h**, a larger catalyst-loading is required and toluene was also necessary since the reaction in MTBE resulted in 13% yield of the target product with 89% recovery of **1h**. Unfortunately, morpholine did not work (Scheme 3).

**Scheme 4:**
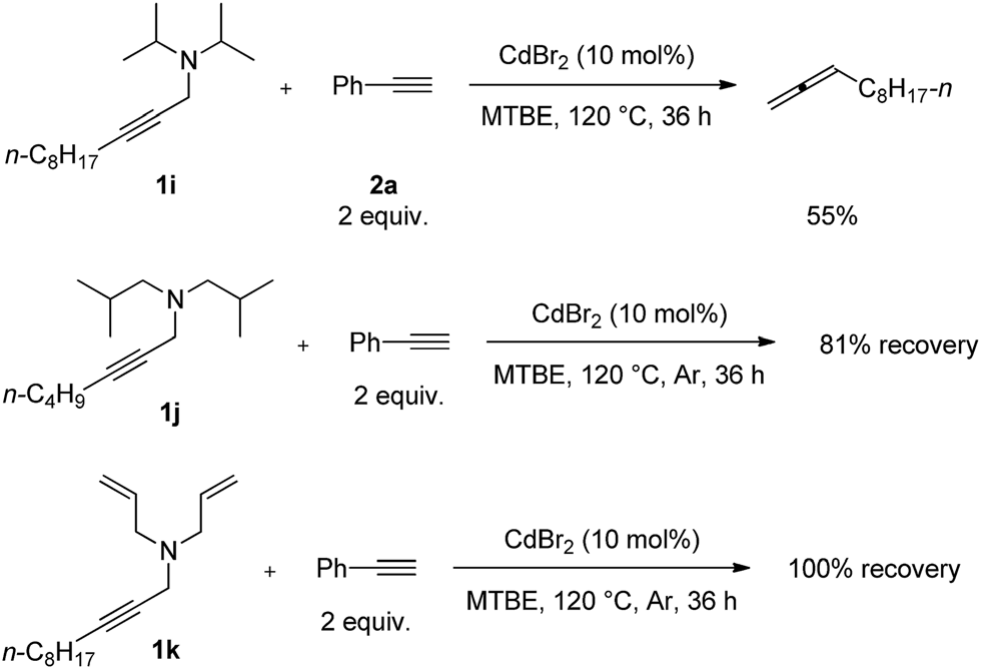
The reaction of non-cyclic amine **1i–1k**. The reaction was conducted using **1i–1k** (1.0 mmol) and phenylacetylene **2a** (2.0 mmol) in 6 mL of MTBE at 120 °C.

Furthermore, several non-cyclic amines were investigated. The reaction of diisopropylamine **1i** with phenylacetylene **2a** generated 55% yield of 1,2-undecadiene^11^–^14^ (Scheme 4). When we applied diisobutylamine **1j** and diallylamine **1k** under the standard reaction conditions, such reactions were not observed.

### Deuterium experiments

**Scheme 5:**
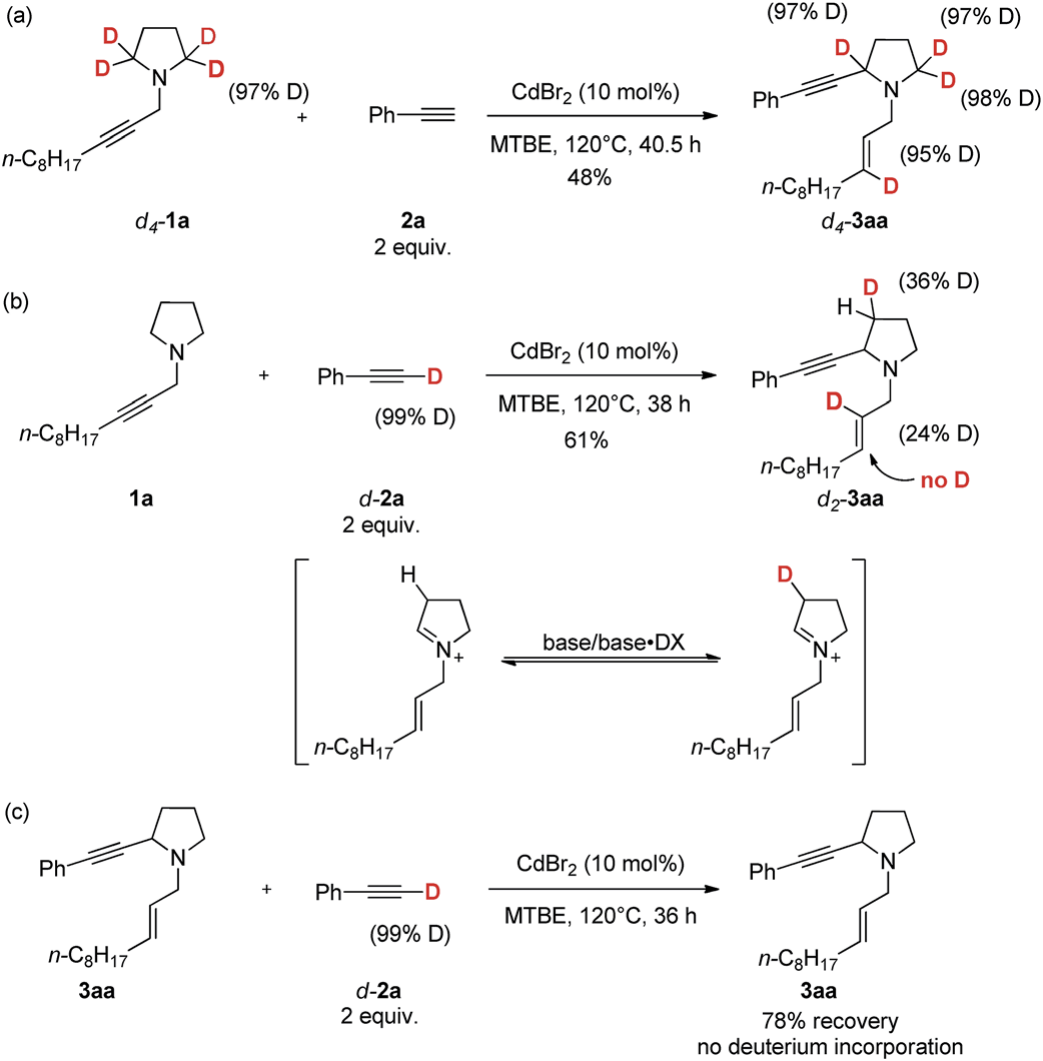
Deuterium labeling experiments.

To gain insight into the mechanism of this reaction, deuterium-labeled *d*_4_-**1a** was treated with **2a** under standard conditions to give *d*_4_-**3aa** with 95% D incorporation, which reveals that the hydrogen at the γ-position of the allylic group comes from the α-position of the amine unit (Scheme 5a). In addition, 24% of deuterium incorporation was observed in the 2-position of the *N*-allylic group in product *d*_2_-**3aa** of the reaction between deuterium-labeled *d*-**2a** and **1a** (Scheme 5b). The control experiment of treating **3aa** with *d*-**2a** led to no deuterium incorporation (Scheme 5c).

**Scheme 6:**
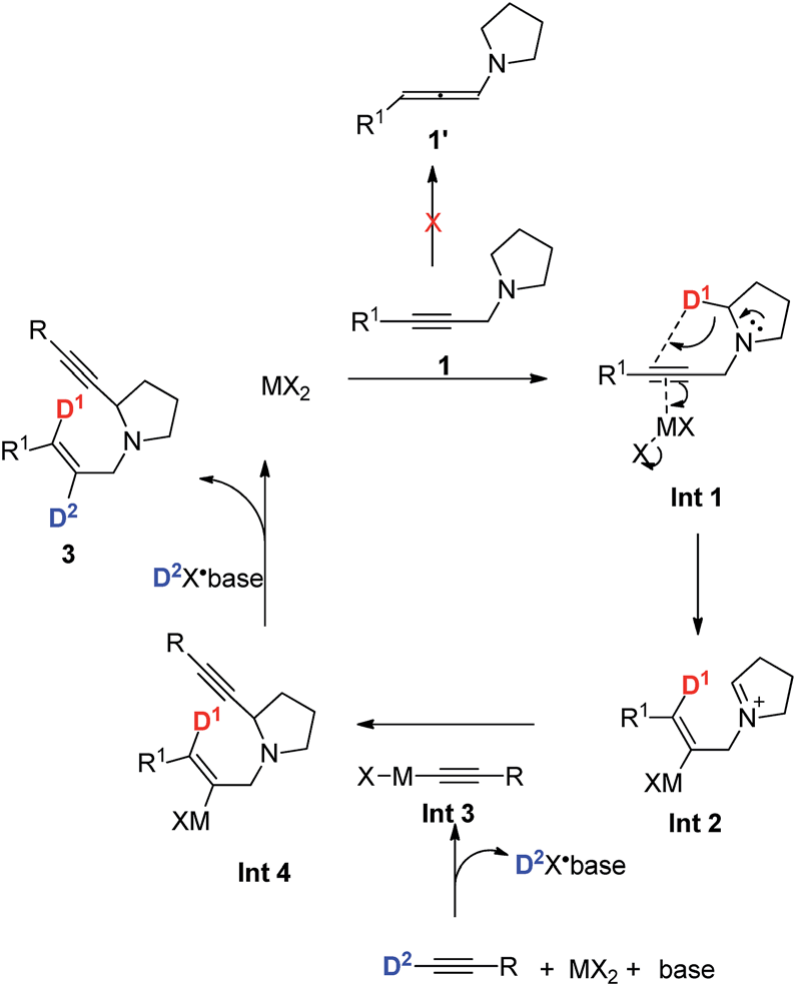
A plausible mechanism for the formation of 3.

Based on the above deuterium labeling experiments and the products in the *E* configuration, a plausible mechanism proposed is shown in Scheme 6. The propargylic amine **1** coordinates to MX_2_ to form **Int 1**, which would undergo *anti*-1,5-hydride transfer to form cationic **Int 2** in the *E* configuration.^15^ Subsequently, 1-alkynyl cadmium species **Int 3**, *in situ* generated from terminal alkyne, CdBr_2_, and amine, would react with the iminium ion **Int 2** to afford the corresponding α-substituted cyclic amine **3** (Scheme 6). In addition, the possibility of forming the product from allenyl amine **1′** is excluded since there is no D-incorporation at the 3-position of the *E*-allylic unit in the product of eqn (b) of Scheme 5. It is believed that CdBr_2_ may coordinate better with the C–C triple bond to trigger the 1,5-H transfer reaction.

**Scheme 7:**
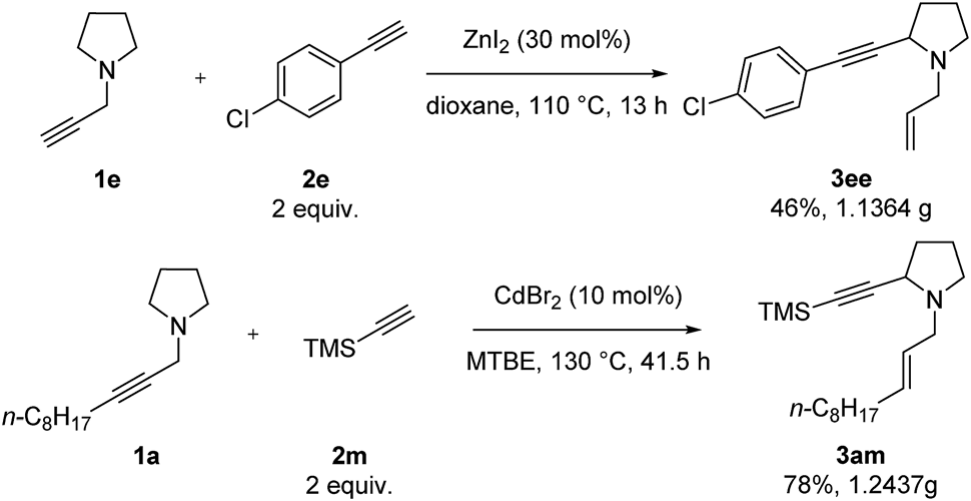
Gram-scale synthesis.

Finally, we conducted a gram-scale synthesis of both **3ee** and **3am** (Scheme 7).

### Synthetic applications

**Scheme 8:**
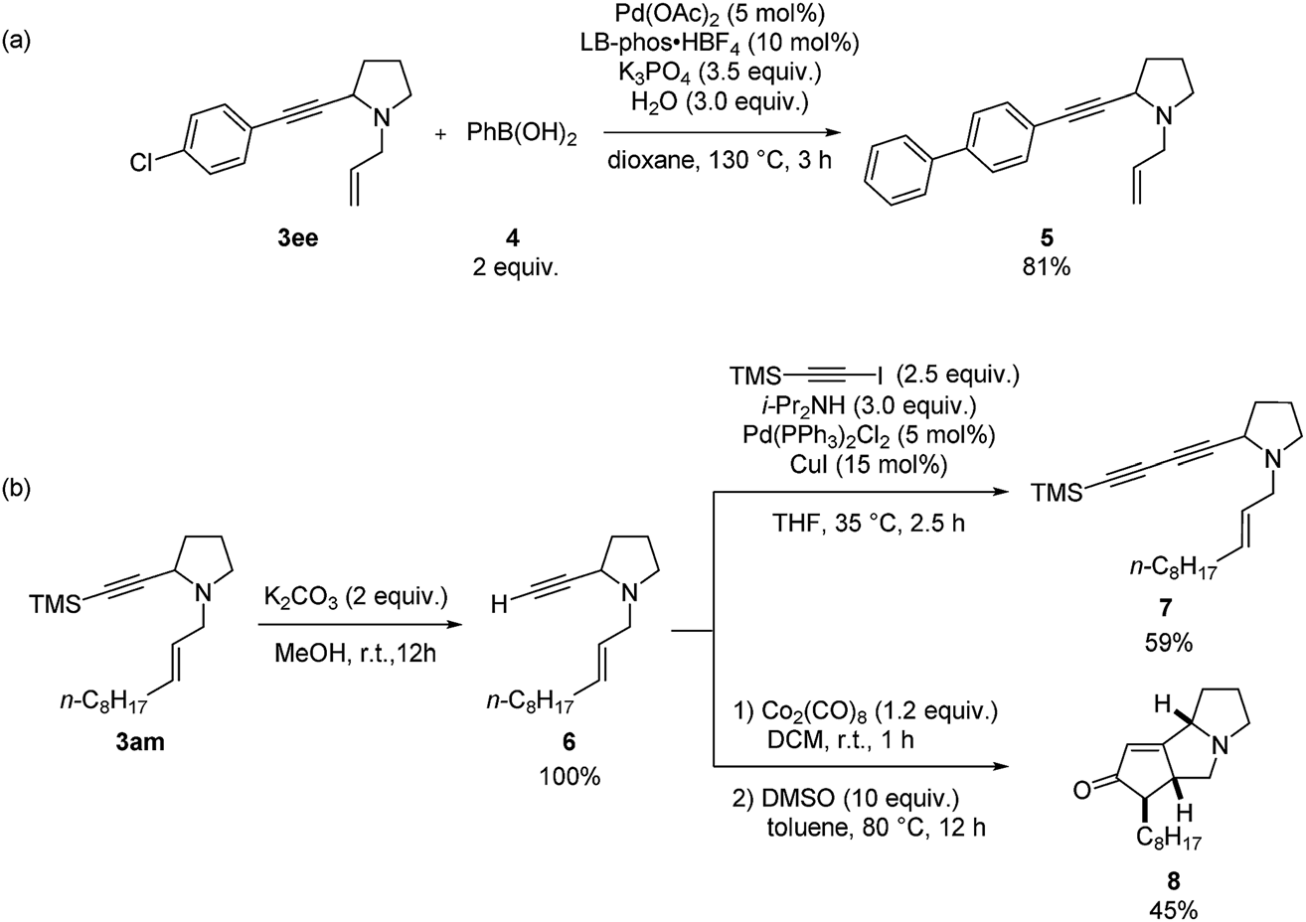
Synthetic applications.

Furthermore, diversified synthetic utilities of these two products were demonstrated. Suzuki coupling between **3ee** and phenyl boronic acid using LB-Phos·HBF_4_ 18 affords **5** in 81% yield (Scheme 8a). Deprotection of the TMS group in **3am** with K_2_CO_3_ in MeOH afforded enyne **6**, which may react with 1-trimethylsilylethynyl iodide to afford conjugated diyne **7** (Scheme 8b). Sequential treatment of **6** with 1.2 equiv. of Co_2_(CO)_8_ and 10 equiv. of DMSO afforded the Pauson–Khand reaction product **8** in 45% yield.^19^

## Conclusions

In conclusion, we have succeeded in developing a catalytic α-alkynylation of *N*-propargylic cyclic amines, providing 1-(2(*E*)-alkenyl) 2-(1-alkynyl) cyclic amines highly stereoselectively. Further studies on identifying the chiral catalyst, the scope of nucleophiles, and their applications to natural products are being actively pursued in the laboratory.

## Conflicts of interest

There are no conflicts to declare.

## Supplementary Material

Supplementary information

Supplementary information
