# Supplementary material for: A metal-catalyzed new approach for α-alkynylation of cyclic amines
Source: Chem Sci. 2018 Nov 26;10(6):1796–801. doi: 10.1039/c8sc04115f (PMC6369436; doi:10.1039/c8sc04115f)
Supplement: Supplementary file 1 [file SC-010-C8SC04115F-s001.pdf]

Electronic Supplementary Material (ESI) for Chemical Science.
This journal is © The Royal Society of Chemistry 2018

A Metal-Catalyzed New Approach for α-Alkynylation of Cyclic Amines

Yifan Cui,[a, b‡] Weilong Lin,[a,b‡] and Shengming Ma[*a,c]

[a] State Key Laboratory of Organometallic Chemistry, Shanghai Institute of Organic Chemistry, Chinese Academy of Sciences, 345 Lingling Lu, Shanghai 200032, P. R. China

[b] University of Chinese Academy of Sciences, Beijing 100049, P. R. China

[c] Department of Chemistry, Fudan University, 220 Handan Lu, Shanghai 200433, P. R. China

[‡] These two authors contributed equally.

General information **S1**

Experimental details and analytical data **S1**

References **S35**

General Information. All reactions have been carried out in oven-dried Schlenk tubes. $CdBr_2$ (98%) was purchased from Alfa and kept in a glove box; ZnI_2 (99%) was purchased from Sinopharm Chemical Reagent Co., Ltd and kept in a glove box. MTBE and 1,4-dioxane were dried over sodium wire with benzophenone as the indicator and distilled freshly before use. Other reagents were used as received without further treatment. All the temperatures are referred to the oil baths used. *N*-internal 2-alkynylic amine **1c**,[1] **1d**,[1] **1e**,[2] **1g**[1] and deuterated phenylacetylene ***d*-2a**[3] were prepared according to literature methods.

1. Synthesis of *N*-propargylic amines

(1) 1-(2-Undecynyl)pyrrolidine (**1a**) (lwl-7-51)

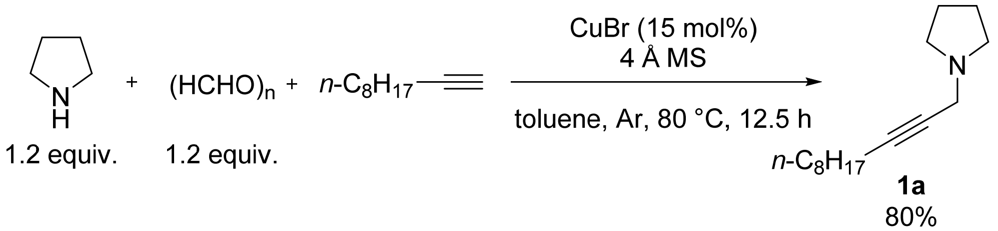

To a flame-dried Schlenk bottle were added CuBr (218.5 mg, 1.5 mmol), 4 Å molecular sieves (3.0125 g), pyrrolidine (855.0 mg, 12 mmol), toluene (10 mL), paraformaldehyde (361.2 mg, 12 mmol), toluene (10 mL), 1-decyne (1.3720 g, 10 mmol), and toluene (10 mL) sequentially under Ar atmosphere. The resulting mixture was then stirred at 80 °C for 12.5 h. After the completion of the reaction as monitored by TLC, the crude reaction mixture was filtrated through a short column of silica gel eluted with EtOAc (50 mL). After evaporation, the residue was purified by column chromatography on silica gel to afford **1a** (1.7821 g, 80%) (eluent: petroleum ether/ ethyl acetate = 1/1) as a liquid: ^{1}H NMR (400 MHz, $CDCl_3$) δ 3.36 (s, 2 H, NCH_2), 2.60 (s, 4 H, 2×NCH_2), 2.18 (t, J = 7.0 Hz, 2 H, CH_2), 1.85-1.73 (m, 4 H, 2×CH_2), 1.54-1.44 (m, 2 H, CH_2), 1.43-1.17 (m, 10 H, 5×CH_2), 0.88 (t, J = 6.6 Hz, 3 H, CH_3); ^{13}C NMR (100 MHz, $CDCl_3$) δ 84.4, 75.6, 52.5, 43.4, 31.8, 29.1, 29.0, 28.81, 28.80, 23.7, 22.6, 18.6, 14.1, 14.0; MS (ESI) *m/z* 222 ($[M+H]^+$); IR (neat) ν = 2927, 2855, 2807, 1459, 1433, 1375, 1349, 1324, 1135 cm^{-1}; HRMS calcd for $C_{15}H_{28}N$ ($[M+H]^+$): 222.2216. Found: 222.2219.

(2) 1-(3-Cyclohexylprop-2-ynyl)pyrrolidine (**1b**) (lwl-8-38)

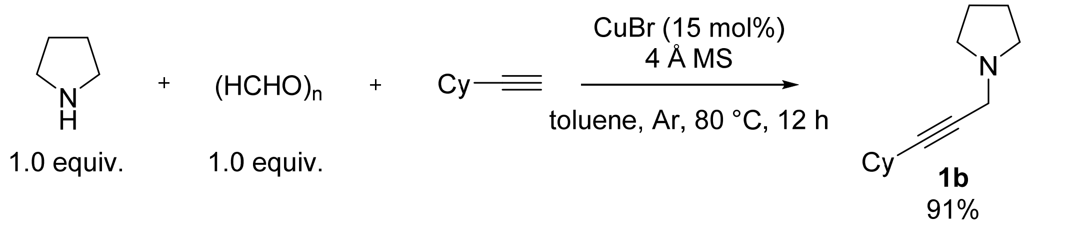

To a flame-dried Schlenk bottle were added CuBr (219.2 mg, 1.5 mmol), 4 Å molecular sieves (3.0020 g), paraformaldehyde (302.1 mg, 10 mmol), toluene (10 mL), pyrrolidine (711.5 mg, 10 mmol), toluene (10 mL), 1-cyclohexylacetylene (1.0751 g, 10 mmol), and toluene (10 mL) sequentially under Ar atmosphere. The resulting mixture was then stirred at 80 °C for 12 h. After the completion of the reaction as monitored by TLC, the crude reaction mixture was filtrated through a short column of silica gel eluted with EtOAc (100 mL). After evaporation, the residue was purified by column chromatography on silica gel to afford **1b** (1.7405 g, 91%) (eluent: petroleum ether/ ethyl acetate = 5/1) as a liquid: ^{1}H NMR (400 MHz, $CDCl_3$) δ 3.37 (s, 2 H, NCH_2), 2.60 (s, 4 H, 2×NCH_2), 2.38 (s, 1 H, CH), 1.86-1.62 (m, 8 H, 4×CH_2), 1.56-1.22 (m, 6 H, 3×CH_2); ^{13}C NMR (100 MHz, $CDCl_3$) δ 88.6, 75.3, 52.4, 43.4, 32.8, 29.0, 25.8, 24.8, 23.7; MS (ESI) *m/z* 192 ($[M+H]^+$); IR (neat) ν = 2926, 2852, 2780, 2727, 1628, 1447, 1371, 1346, 1322, 1294, 1258, 1235, 1198, 1159, 1124, 1030 cm^{-1}; HRMS calcd for $C_{13}H_{22}N$ ($[M+H]^+$): 192.1747. Found: 192.1748.

(3) 2-(2-Undecynyl)-1,2,3,4-tetrahydroisoquinoline (**1f**) (cyf-1-103)

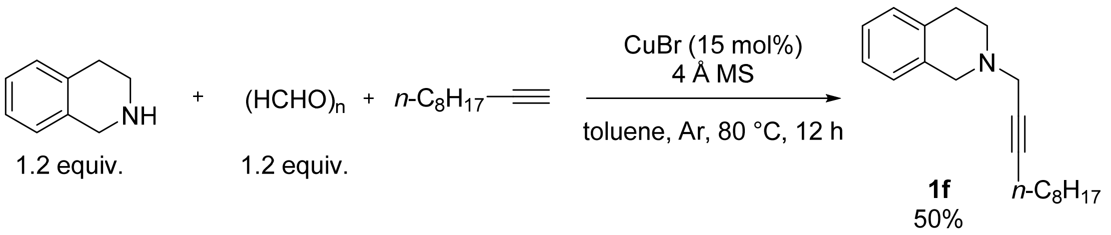

To a flame-dried Schlenk bottle were added CuBr (110.5 mg, 0.75 mmol), 4 Å molecular sieves (1.5 g), paraformaldehyde (191.6 mg, 6.0 mmol), 1,2,3,4-tetrahydroisoquinoline (817.5 mg, 6.0 mmol), toluene (7.5 mL), 1-decyne (726.2 mg, 5.0 mmol), and toluene (7.5 mL) sequentially under Ar atmosphere. The

resulting mixture was then stirred at 80 °C for 12 h. After the completion of the reaction as monitored by TLC, the crude reaction mixture was filtrated through a short column of silica gel eluted with DCM (50 mL). After evaporation, the residue was purified by column chromatography on silica gel to afford **1f** (867.2 mg, 50%) (eluent: petroleum ether/ acetone = 100/1) as a liquid: ^{1}H NMR (400 MHz, $CDCl_3$) δ 7.16-7.07 (m, 3 H, ArH), 7.07-7.01 (m, 1 H, ArH), 3.75 (s, 2 H, NCH_2), 3.46 (s, 2 H, NCH_2), 2.94 (t, J = 5.6 Hz, 2 H, CH_2), 2.81 (t, J = 6.0 Hz, 2 H, CH_2), 2.20 (t, J = 6.8 Hz, 2 H, CH_2), 1.50 (q, J = 7.1 Hz, 2 H, CH_2), 1.43-1.17 (m, 10 H, 5×CH_2), 0.87 (t, J = 6.4 Hz, 3 H, CH_3); ^{13}C NMR (100 MHz, $CDCl_3$) δ 134.7, 133.9, 128.6, 126.6, 126.0, 125.5, 85.7, 74.6, 54.5, 49.8, 47.2, 31.8, 29.3, 29.2, 28.89, 28.86, 22.6, 18.7, 14.1; MS (EI) *m/z* (%) 283 (M^+, 36.97), 282 (100); IR (neat) v = 3022, 2923, 1585, 1497, 1456, 1429, 1381, 1364, 1328, 1277, 1230, 1192, 1132, 1089, 1056, 1039, 1006 cm^{-1}; HRMS calcd for $C_{20}H_{29}N$ (M^+): 283.2300. Found: 283.2294.

(4) 1-(2-Undecynyl) piperidine (**1h**) (cyf-2-94)

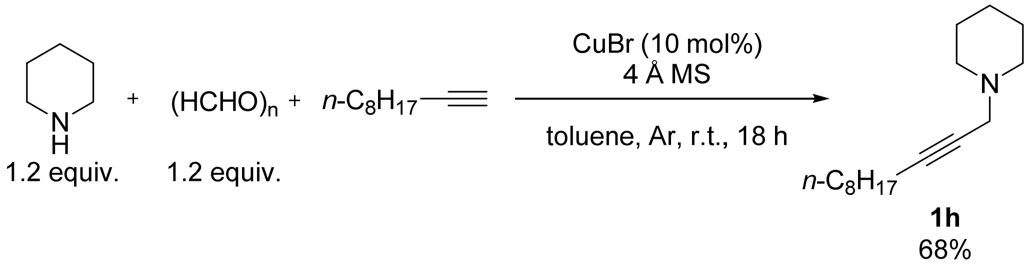

To a flame-dried Schlenk bottle were added CuBr (146.8 mg, 1.0 mmol), 4 Å molecular sieves (3.0113 g), paraformaldehyde (384.2 mg, 12.0 mmol), piperidine (1.0256 g, 12.0 mmol), toluene (15 mL), 1-decyne (1.4535 g, 10.0 mmol), and toluene (15 mL) sequentially under Ar atmosphere. The resulting mixture was then stirred at room temperature for 18 h. After the completion of the reaction as monitored by TLC, the crude reaction mixture was filtrated through a short column of silica gel eluted with ethyl ether (100 mL). After evaporation, the residue was purified by column chromatography on silica gel to afford **1h** (1.6009 g, 68%) (eluent: petroleum ether/ ethyl acetate = 5/1) as a liquid: ^{1}H NMR (400 MHz, $CDCl_3$) δ 3.21 (s, 2 H, NCH_2),

2.48 (s, 4 H, 2×NCH_2), 2.19 (t, J = 7.0 Hz, 2 H, CH_2), 1.62 (quint, J = 5.5 Hz, 4 H, 2×CH_2), 1.55-1.20 (m, 14 H, 7×CH_2), 0.88 (t, J = 6.6 Hz, 3 H, CH_3); ^{13}C NMR (100 MHz, $CDCl_3$) δ 85.1, 75.1, 53.2, 48.0, 31.7, 29.1, 29.0, 28.79, 28.77, 25.8, 23.9, 22.6, 18.6, 14.0; MS (ESI) *m/z* 236 ($[M+H]^+$); IR (neat) *v* = 2927, 2854, 2794, 2752, 2681, 1466, 1453, 1379, 1367, 1340, 1324, 1308, 1298, 1275, 1252, 1203, 1188, 1156, 1105, 1067, 1039 cm^{-1}; HRMS calcd for $C_{16}H_{30}N$ ($[M+H]^+$): 236.2373. Found: 236.2376.

(5) N-(2-undecynyl)-diisopropylamine (**1i**) (cyf-2-88)

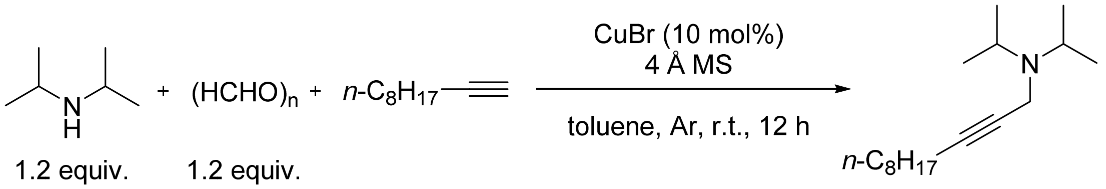

1i
33%

To a flame-dried Schlenk bottle were added CuBr (147.0 mg, 1.0 mmol), 4 Å molecular sieves (3.0117 g), paraformaldehyde (383.7 mg, 12.0 mmol), diisopropylamine (1.2301 g, 12.0 mmol), toluene (15 mL), 1-decyne (1.4551 g, 10.0 mmol), and toluene (15 mL) sequentially under Ar atmosphere. The resulting mixture was then stirred at room temperature for 12 h. After the completion of the reaction as monitored by TLC, the crude reaction mixture was filtrated through a short column of silica gel eluted with EtOAc (100 mL). After evaporation, the residue was purified by column chromatography on silica gel to afford **1i** (822.4 mg, 33%) (eluent: petroleum ether/ ethyl acetate = 100/1) as a liquid: ^{1}H NMR (400 MHz, $CDCl_3$) δ 3.39 (t, J = 2.0 Hz, 2 H, NCH_2), 3.19 (heptet, J = 6.5 Hz, 2 H, 2×NCH), 2.18-2.12 (m, 2 H, CH_2), 1.48 (quint, J = 7.1 Hz, 2 H, CH_2), 1.42-1.21 (m, 10 H, 5×CH_2), 1.09 (d, J = 6.4 Hz, 12 H, 4×CH_3), 0.88 (t, J = 6.6 Hz, 3 H, CH_3); ^{13}C NMR (100 MHz, $CDCl_3$) δ 83.5, 78.7, 48.20, 48.18, 34.3, 31.8, 29.2, 29.1, 28.9, 28.8, 22.6, 20.5, 18.8, 14.1; MS (ESI) *m/z* 252 ($[M+H]^+$); IR (neat) *v* = 2961, 2926, 2855, 1463, 1434, 1379, 1362, 1324, 1204, 1176, 1139, 1118, 1025 cm^{-1}; HRMS calcd for $C_{17}H_{34}N$ ($[M+H]^+$): 252.2686. Found: 252.2686.

2. The $CdBr_2$-catalyzed reaction of propargylic amines with terminal alkynes

(1) (*E*)-2-(Phenylethynyl)-1-(2-undecenyl)pyrrolidine (**3aa**) (lwl-7-195, lwl-7-93)

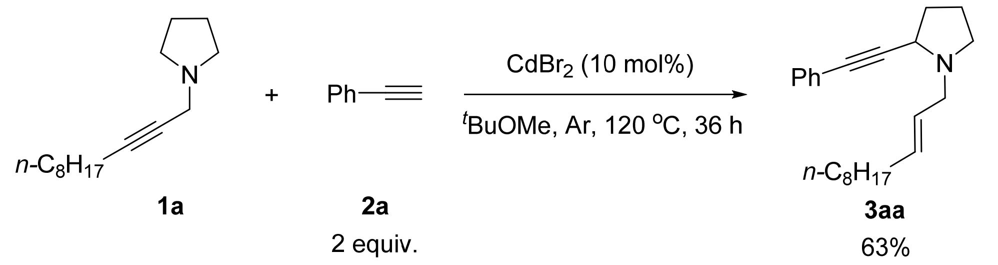

To a dry Schlenk tube with a polytetrafluoroethylene plug was added $CdBr_2$ (27.6 mg, 0.1 mmol). Then the catalyst was dried under vacuum with heating. After cooling to room temperature, **1a** (221.7 mg, 1.0 mmol), MTBE (3 mL), phenylacetylene **2a** (205.1 mg, 2.0 mmol), and MTBE (3 mL) were added sequentially under Ar atmosphere. The resulting mixture was stirred at 120 °C for 36 h. After the completion of the reaction as monitored by TLC, the crude reaction mixture was filtrated through a short column of Al_2O_3 eluted with EtOAc (50 mL). After evaporation, the residue was purified by column chromatography on Al_2O_3 to afford **3aa** (203.3 mg, 63%) (eluent: petroleum ether/ ethyl acetate = 100/1) as a liquid: ^{1}H NMR (400 MHz, $CDCl_3$) δ 7.50-7.37 (m, 2 H, ArH), 7.34-7.21 (m, 3 H, ArH), 5.67 (dt, *J* = 15.2, 6.4 Hz, 1 H, =CH), 5.58 (dt, *J* = 15.2, 6.0 Hz, 1 H, =CH), 3.57 (dd, *J* = 7.3, 5.5 Hz, 1 H, NCH), 3.49 (dd, *J* = 12.8, 5.9 Hz, 1 H, one proton of NCH_2), 3.01 (dd, *J* = 12.5, 7.5 Hz, 1 H, one proton of NCH_2), 2.84 (td, *J* = 8.8, 4.9 Hz, 1 H, one proton of NCH_2), 2.50 (td, *J* = 8.9, 6.5 Hz, 1 H, one proton of NCH_2), 2.23-2.12 (m, 1 H, one proton of CH_2), 2.06-1.75 (m, 5 H, 2×CH_2 and one proton of CH_2), 1.41-1.17 (m, 12 H, 6×CH_2), 0.88 (t, *J* = 6.6 Hz, 3 H, CH_3); ^{13}C NMR (100 MHz, $CDCl_3$) δ 134.1, 131.6, 128.1, 127.8, 126.8, 123.4, 88.7, 84.5, 55.3, 54.5, 51.6, 32.3, 31.8, 31.7, 29.4, 29.24, 29.18, 29.1, 22.6, 22.0, 14.1; MS (EI) *m/z* (%) 323 (M^+, 19.57), 170 (100); IR (neat) *ν* = 2955, 2923, 2853, 2807, 1695, 1599, 1489, 1460, 1443, 1354, 1178 cm^{-1}; HRMS calcd for $C_{23}H_{33}N$ (M^+): 323.2613. Found: 323.2610.

(2) (*E*)-2-(4-tolyethynyl)-1-(2-undecenyl)pyrrolidine (**3ab**) (cyf-2-62)

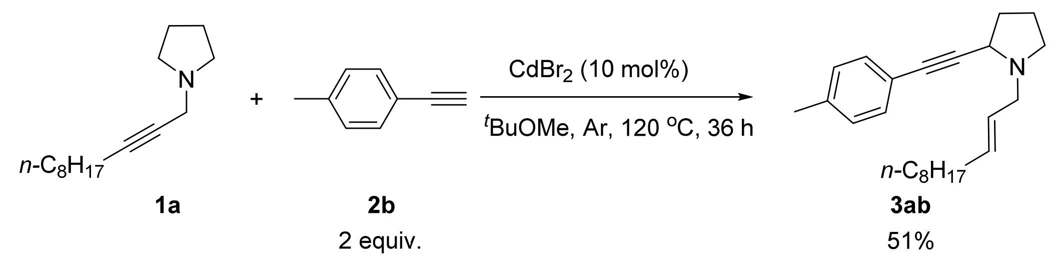

Typical Procedure I: To a dry Schlenk tube with a polytetrafluoroethylene plug was added $CdBr_2$ (27.7 mg, 0.1 mmol). Then the catalyst was dried under vacuum with heating. **1a** (221.1 mg, 1.0 mmol), MTBE (3 mL), 4-tolyacetylene **2b** (238.5 mg, 2.0 mmol), and MTBE (3 mL) were then added sequentially under Ar atmosphere. The resulting mixture was stirred at 120 °C for 36 h. After the completion of the reaction as monitored by TLC, the crude reaction mixture was filtrated through a short column of Al_2O_3 eluted with EtOAc (50 mL). After evaporation, the residue was purified by column chromatography on silica gel to afford **3ab** (172.0 mg, 51%) (eluent: petroleum ether/ acetone = 100/1 (300 mL) to 50/1 (200 mL)) as a liquid: δ 7.32 (d, *J* = 7.6 Hz, 2 H, ArH), 7.09 (d, *J* = 8.0 Hz, 2 H, ArH), 5.66 (dt, *J* = 15.2, 6.4 Hz, 1 H, =CH), 5.57 (dt, *J* = 15.2, 6.6 Hz, 1 H, =CH), 3.55 (t, *J* = 6.2 Hz, 1 H, NCH), 3.48 (dd, *J* = 13.0, 5.8 Hz, 1 H, one proton of NCH_2), 3.00 (dd, *J* = 12.8, 7.2 Hz, 1 H, one proton of NCH_2), 2.84 (td, *J* = 8.5, 5.2 Hz, 1 H, one proton of NCH_2), 2.53-2.44 (m, 1 H, one proton of NCH_2), 2.33 (s, 3 H, CH_3), 2.22-2.12 (m, 1 H, one proton of CH_2), 2.07-1.74 (m, 5 H, 2×CH_2 and one proton of CH_2), 1.42-1.17 (m, 12 H, 6×CH_2), 0.88 (t, *J* = 6.6 Hz, 3 H, CH_3); ^{13}C NMR (100 MHz, $CDCl_3$) δ 137.7, 133.9, 131.5, 128.8, 126.8, 120.2, 87.8, 84.5, 55.3, 54.5, 51.5, 32.3, 31.8, 31.7, 29.4, 29.2, 29.12, 29.06, 22.6, 21.9, 21.3, 14.0; MS (ESI) *m/z* 338 ($[M+H]^+$); IR (neat) *v* = 2954, 2922, 2852, 2805, 1668, 1509, 1456, 1376, 1319, 1277, 1257, 1212, 1139, 1107, 1049, 1020 cm^{-1}; HRMS calcd for $C_{24}H_{36}N$ ($[M+H]^+$): 338.2842. Found: 338.2838.

(3) (*E*)-2-((4-Methoxyphenyl)ethynyl)-1-(2-undecenyl)pyrrolidine (**3ac**) (cyf-2-60)

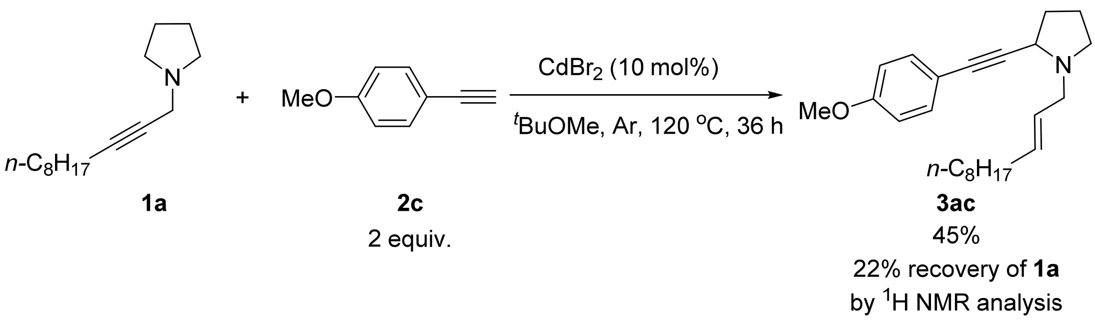

Following **Typical Procedure I**, the reaction of $CdBr_2$ (27.7 mg, 0.1 mmol), **1a** (221.6 mg, 1.0 mmol), 4-methoxyphenylacetylene **2c** (270.2 mg, 2.0 mmol), and MTBE (6.0 mL) afforded **3ac** (159.1 mg, 45%) (22% recovery of **1a** as determined by ^{1}H NMR analysis of the crude product using CH_2Br_2 as internal standard) (eluent: petroleum ether/ acetone = 100/1 (300 mL) to 50/1 (200 mL)) as a liquid: ^{1}H NMR (400 MHz, $CDCl_3$) δ 7.36 (d, J = 8.8 Hz, 2 H, ArH), 6.82 (d, J = 8.8 Hz, 2 H, ArH), 5.66 (dt, J = 14.8, 6.4 Hz, 1 H, =CH), 5.58 (dt, J = 15.2, 6.6 Hz, 1 H, =CH), 3.80 (s, 3 H, OCH_3), 3.57-3.46 (m, 2 H, NCH and one proton of NCH_2), 2.99 (dd, J = 12.8, 7.2 Hz, 1 H, one proton of NCH_2), 2.85 (td, J = 8.7, 4.9 Hz, 1 H, one proton of NCH_2), 2.52-2.44 (m, 1 H, one proton of NCH_2), 2.23-2.12 (m, 1 H, one proton of CH_2), 2.08-1.74 (m, 5 H, 2×CH_2 and one proton of CH_2), 1.42-1.18 (m, 12 H, 6×CH_2）, 0.88 (t, J = 6.8 Hz, 3 H, CH_3); ^{13}C NMR (100 MHz, $CDCl_3$) δ 159.2, 134.0, 133.0, 126.7, 115.4, 113.7, 87.0, 84.3, 55.3, 55.1, 54.6, 51.5, 32.3, 31.8, 31.7, 29.4, 29.2, 29.15, 29.08, 22.6, 21.9, 14.0; MS (ESI) m/z 354 ([M+H]$^+$); IR (neat) ν = 2954, 2922, 2852, 2806, 1606, 1570, 1508, 1461, 1441, 1376, 1354, 1319, 1288, 1245, 1171, 1105, 1033 cm^{-1}; HRMS calcd for $C_{24}H_{36}NO$ ([M+H]$^+$): 354.2791. Found: 354.2785.

(4) (*E*)-2-((4-Fluorophenyl)ethynyl)-1-(2-undecenyl)pyrrolidine (**3ad**) (cyf-2-58)

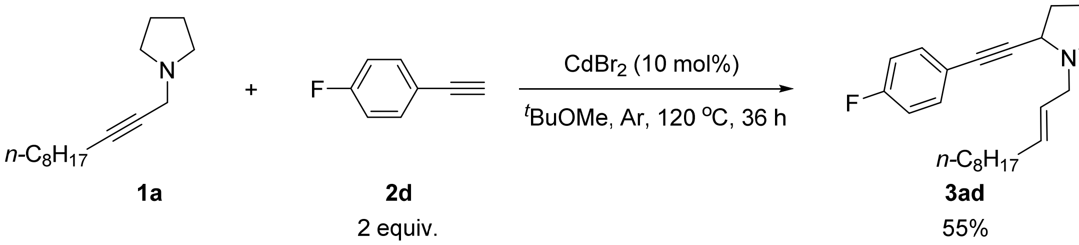

Following **Typical Procedure I**, the reaction of $CdBr_2$ (27.7 mg, 0.1 mmol), **1a** (222.0 mg, 1.0 mmol), 4-fluorophenylacetylene **2d** (245.3 mg, 2.0 mmol), and MTBE

(6.0 mL) afforded **3ad** (187.8 mg, 55%) (eluent: petroleum ether/ acetone = 100/1 (300 mL) to 50/1 (200 mL)) as a liquid: ^{1}H NMR (400 MHz, $CDCl_3$) δ 7.40 (dd, *J* = 8.0, 6.0 Hz, 2 H, ArH), 6.98 (t, *J* = 8.8 Hz, 2 H, ArH), 5.66 (dt, *J* = 15.6, 6.4 Hz, 1 H, =CH), 5.57 (dt, *J* = 15.2, 6.6 Hz, 1 H, =CH), 3.57-3.43 (m, 2 H, NCH and one proton of NCH_2), 2.99 (dd, *J* = 12.8, 7.2 Hz, 1 H, one proton of NCH_2), 2.85 (td, *J* = 8.4, 5.1 Hz, 1 H, one proton of NCH_2), 2.54-2.43 (m, 1 H, one proton of NCH_2), 2.23-2.12 (m, 1 H, one proton of CH_2), 2.07-1.74 (m, 5 H, 2×CH_2 and one proton of CH_2), 1.42-1.20 (m, 12 H, 6×CH_2, 0.88 (t, *J* = 6.8 Hz, 3 H, CH_3); ^{13}C NMR (100 MHz, $CDCl_3$) δ 162.2 (*J* = 247.1 Hz), 134.1, 133.5 (*J* = 8.5 Hz), 126.7, 119.4 (*J* = 3.6 Hz), 115.4 (*J* = 21.8 Hz), 88.5, 83.4, 55.4, 54.5, 51.6, 32.3, 31.9, 31.8, 29.4, 29.3, 29.25, 29.17, 22.6, 22.0, 14.1; ^{19}F NMR (376 MHz, $CDCl_3$) δ -112.1- -112.3 (m); MS (EI) *m/z* (%) 341 (M^+, 8.03), 83 (100); IR (neat) *v* = 2955, 2924, 2853, 2806, 1601, 1505, 1462, 1354, 1320, 1228, 1154, 1112, 1091 cm^{-1}; Raman: *v* = 2231 cm^{-1}; HRMS calcd for $C_{23}H_{32}NF$ (M^+): 341.2519. Found: 341.2514.

(5) (*E*)-2-((4-Chlorophenyl)ethynyl)-1-(2-undecenyl)pyrrolidine (**3ae**) (lwl-7-198)

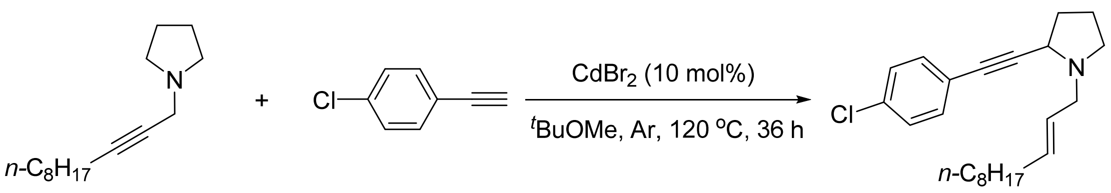

Following **Typical Procedure I**, the reaction of $CdBr_2$ (27.6 mg, 0.1 mmol), **1a** (220.5 mg, 1.0 mmol), 4-chlorophenylacetylene **2e** (273.1 mg, 2.0 mmol), and MTBE (6.0 mL) afforded **3ae** (238.9 mg, 67%) (eluent: petroleum ether/ ethyl acetate/ dichloromethane/ aqueous ammonia = 20/1/1/0.08) as a liquid: ^{1}H NMR (400 MHz, $CDCl_3$) δ 7.34 (d, *J* = 8.3 Hz, 2 H, ArH), 7.26 (d, *J* = 8.5 Hz, 2 H, ArH), 5.66 (dt, *J* = 15.3, 6.4 Hz, 1 H, =CH), 5.57 (dt, *J* = 15.5, 6.3 Hz, 1 H, =CH), 3.54 (t, *J* = 6.4 Hz, 1 H, NCH), 3.47 (dd, *J* = 12.7, 5.5 Hz, 1 H, one proton of NCH_2), 2.99 (dd, *J* = 12.9, 7.3 Hz, 1 H, one proton of NCH_2), 2.85 (td, *J* = 8.7, 4.9 Hz, 1 H, one proton of NCH_2), 2.53-2.41 (m, 1 H, one proton of NCH_2), 2.23-2.11 (m, 1 H, one proton of CH_2),

2.07-1.74 (m, 5 H, 2×CH_2 and one proton of CH_2), 1.42-1.15 (m, 12 H, 6×CH_2), 0.88 (t, J = 6.7 Hz, 3 H, CH_3); ^{13}C NMR (100 MHz, $CDCl_3$) δ 134.1, 133.8, 132.9, 128.5, 126.7, 121.8, 89.9, 83.4, 55.4, 54.5, 51.2, 32.3, 31.8, 31.7, 29.4, 29.25, 29.2, 29.1, 22.6, 22.0, 14.1; MS (EI) *m/z* (%) 359 ($M^+(Cl^{37})$, 2.11), 357 ($M^+(Cl^{35})$, 6.13), 83 (100); IR (neat) v = 2955, 2923, 2853, 2807, 1488, 1462, 1374, 1353, 1319, 1256, 1139, 1092, 1014 cm^{-1}; HRMS calcd for $C_{23}H_{32}NCl$ ($M^+(Cl^{35})$): 357.2223. Found: 357.2225.

(6) (*E*)-2-((3-Chlorophenyl)ethynyl)-1-(2-undecenyl)pyrrolidine (**3af**) (lwl-8-57)

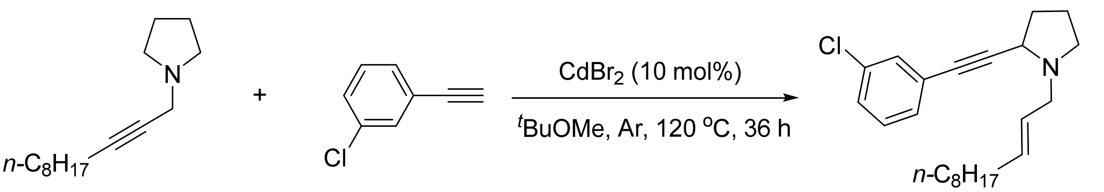

1a **2f** 2 equiv. **3af** 66%

Following **Typical Procedure I**, the reaction of $CdBr_2$ (27.2 mg, 0.1 mmol), **1a** (221.5 mg, 1.0 mmol), 3-chlorophenylacetylene **2f** (273.2 mg, 2.0 mmol), and MTBE (6.0 mL) afforded **3af** (235.1 mg, 66%) (eluent: petroleum ether/ ethyl acetate/ dichloromethane/ aqueous ammonia = 20/1/1/0.08) as a liquid: ^{1}H NMR (400 MHz, $CDCl_3$) δ 7.41 (s, 1 H, ArH), 7.32-7.17 (m, 3 H, ArH), 5.67 (dt, J = 15.3, 6.4 Hz, 1 H, =CH), 5.56 (dt, J = 15.4, 6.1 Hz, 1 H, =CH), 3.56 (dd, J = 7.3, 5.3 Hz, 1 H, NCH), 3.46 (dd, J = 12.7, 5.7 Hz, 1 H, one proton of NCH_2), 3.00 (dd, J = 12.8, 7.3 Hz, 1 H, one proton of NCH_2), 2.83 (td, J = 8.8, 5.2 Hz, 1 H, one proton of NCH_2), 2.51 (td, J = 8.9, 6.4 Hz, 1 H, one proton of NCH_2), 2.23-2.12 (m, 1 H, one proton of CH_2), 2.07-1.87 (m, 4 H, CH_2 and 2×one proton of CH_2), 1.87-1.75 (m, 1 H, one proton of CH_2), 1.43-1.16 (m, 12 H, 6×CH_2), 0.88 (t, J = 6.8 Hz, 3 H, CH_3); ^{13}C NMR (100 MHz, $CDCl_3$) δ 134.1, 133.9, 131.5, 129.7, 129.3, 128.1, 126.6 125.0, 90.2, 83.1, 55.3, 54.4, 51.6, 32.3, 31.8, 31.6, 29.4, 29.24, 29.15, 29.1, 22.6, 22.0, 14.1; MS (EI) *m/z* (%) 359 ($M^+(Cl^{37})$, 9.85), 357 ($M^+(Cl^{35})$, 31.73), 204 (100); IR (neat) v = 3066, 2925, 2854, 2807, 1695, 1593, 1562, 1474, 1406, 1377, 1354, 1318, 1246, 1139, 1112, 1093,

1078 cm^{-1}; HRMS calcd for $C_{23}H_{32}NCl$ ($M^+(Cl^{35})$): 357.2223. Found: 357.2231.

(7) (*E*)-2-((4-Nitrophenyl)ethynyl)-1-(2-undecenyl)pyrrolidine (**3ag**) (lwl-8-10)

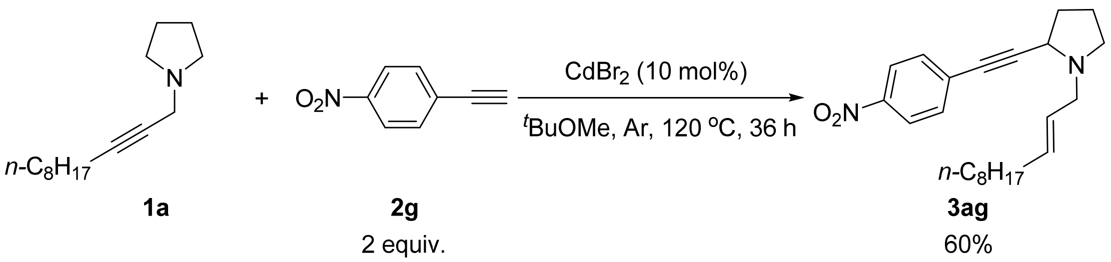

To a dry Schlenk tube with a polytetrafluoroethylene plug was added $CdBr_2$ (27.4 mg, 0.1 mmol). Then the catalyst was dried under vacuum with heating. After cooling to room temperature, **1a** (221.5 mg, 1.0 mmol), MTBE (3 mL), 4-nitrophenylacetylene **2g** (295.1 mg, 2.0 mmol), and MTBE (3 mL) were added sequentially under Ar atmosphere. The resulting mixture was stirred at 120 °C for 36 h. After the completion of the reaction as monitored by TLC, the crude reaction mixture was filtrated through a short column of Al_2O_3 eluted with EtOAc (50 mL). After evaporation, the residue was purified by column chromatography on Al_2O_3 to afford **3ag** (222.2 mg, 60%) (eluent: petroleum ether/ dichloromethane / ethyl acetate = 200/20/1 to 100/10/1) as a liquid: ^1H NMR (400 MHz, $CDCl_3$) δ 8.17 (d, *J* = 8.8 Hz, 2 H, ArH), 7.56 (d, *J* = 8.6 Hz, 2 H, ArH), 5.68 (dt, *J* = 15.1, 6.6 Hz, 1 H, =CH), 5.57 (dt, *J* = 15.4, 6.1 Hz, 1 H, =CH), 3.62 (dd, *J* = 7.2, 5.5 Hz, 1 H, NCH), 3.48 (dd, *J* = 12.7, 5.7 Hz, 1 H, one proton of NCH_2), 3.03 (dd, *J* = 12.7, 7.2 Hz, 1 H, one proton of NCH_2), 2.87 (td, *J* = 8.8, 5.2 Hz, 1 H, one proton of NCH_2), 2.56-2.48 (m, 1 H, one proton of NCH_2), 2.28-2.16 (m, 1 H, one proton of CH_2), 2.10-1.79 (m, 5 H, $2\times CH_2$ and one proton of CH_2), 1.43-1.17 (m, 12 H, $6\times CH_2$), 0.87 (t, *J* = 6.8 Hz, 3 H, CH_3); ^{13}C NMR (100 MHz, $CDCl_3$) δ 146.8, 134.5, 132.3, 130.3, 126.3, 123.4, 94.9, 82.9, 55.4, 54.5, 51.7, 32.3, 31.8, 31.5, 29.4, 29.2, 29.14, 29.10, 22.6, 22.1, 14.1; MS (ESI) *m/z* 369 ($[M+H]^+$); IR (neat) ν = 2924, 2854, 2805, 2224, 1594, 1520, 1491, 1461, 1377, 1344, 1318, 1285, 1256, 1175, 1139, 1108, 1013 cm^{-1}; HRMS calcd for $C_{23}H_{33}N_2O_2$ ($[M+H]^+$): 369.2537. Found: 369.2538.

(8) (*E*)-2-((4-Ethoxycarbonylphenyl)ethynyl)-1-(2-undecenyl)pyrrolidine (**3ah**) (cyf-2-63)

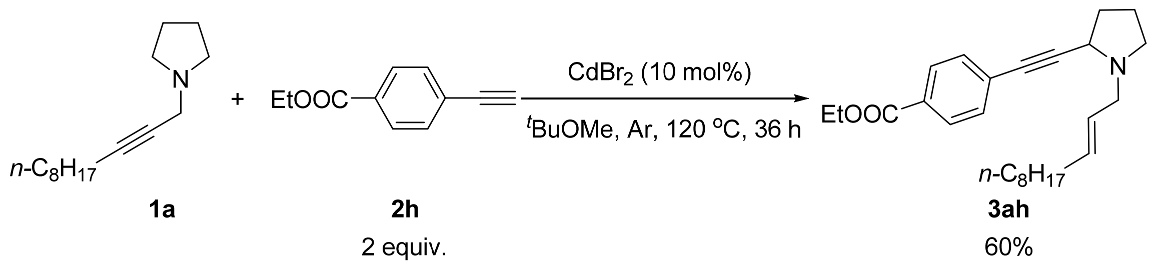

Following **Typical Procedure I**, the reaction of $CdBr_2$ (27.7 mg, 0.1 mmol), **1a** (222.0 mg, 1.0 mmol), 4-ethoxycarbonylphenylacetylene **2h** (356.1 mg, 2.0 mmol), and MTBE (6.0 mL) afforded **3ah** (237.2 mg, 60%) (eluent: petroleum ether/ acetone = 100/1 (300 mL) to 50/1 (300 mL)) as a liquid: ^{1}H NMR (400 MHz, $CDCl_3$) δ 7.97 (d, *J* = 8.4 Hz, 2 H, ArH), 7.47 (d, *J* = 8.0 Hz, 2 H, ArH), 5.67 (dt, *J* = 15.2, 6.4 Hz, 1 H, =CH), 5.57 (dt, *J* = 15.2, 6.4 Hz, 1 H, =CH), 4.37 (q, *J* = 7.1 Hz, 2 H, OCH_2), 3.58 (dd, *J* = 7.4, 5.4 Hz, 1 H, NCH), 3.47 (dd, *J* = 12.6, 5.8 Hz, 1 H, one proton of NCH_2), 3.02 (dd, *J* = 12.8, 7.2 Hz, 1 H, one proton of NCH_2), 2.85 (td, *J* = 8.7, 5.1 Hz, 1 H, one proton of NCH_2), 2.56-2.47 (m, 1 H, one proton of NCH_2), 2.25-2.14 (m, 1 H, one proton of CH_2), 2.08-1.76 (m, 5 H, 2×CH_2 and one proton of CH_2), 1.44-1.17 (m, 15 H, 6×CH_2 and CH_3), 0.87 (t, *J* = 6.6 Hz, 3 H, CH_3); ^{13}C NMR (100 MHz, $CDCl_3$) δ 165.9, 134.1, 131.4, 129.4, 129.2, 127.9, 126.6, 92.0, 83.9, 60.9, 55.3, 54.4, 51.6, 32.2, 31.8, 31.6, 29.3, 29.2, 29.1, 29.0, 22.6, 22.0, 14.2, 14.0; MS (EI) *m/z* (%) 395 (M^+, 9.22), 256 (100); IR (neat) *v* = 2956, 2924, 2853, 2805, 2224, 1720, 1605, 1462, 1404, 1367, 1269, 1174, 1102, 1021 cm^{-1}; Raman: *v* = 2228 cm^{-1}; HRMS calcd for $C_{26}H_{37}NO_2$ (M^+): 395.2824. Found: 395.2819.

(9) (*E*)-2-((4-cyanophenyl)ethynyl)-1-(2-undecenyl)pyrrolidine (**3ai)** (lwl-8-58)

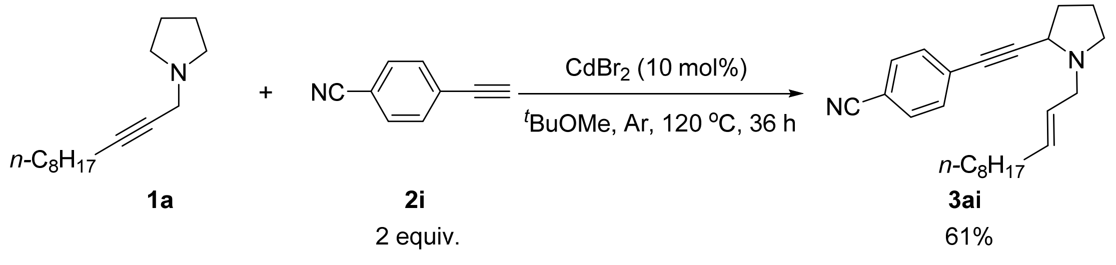

Following **Typical Procedure I**, the reaction of $CdBr_2$ (27.5 mg, 0.1 mmol), **1a** (222.0 mg, 1.0 mmol), 4-cyanophenylacetylene **2i** (254.1 mg, 2.0 mmol), and MTBE (6.0 mL) afforded **3ai** (213.1 mg, 61%) (eluent: petroleum ether/ ethyl acetate/ dichloromethane/ aqueous ammonia = 250/25/12.5/1) as a liquid: ^{1}H NMR (400 MHz, $CDCl_3$) δ 7.58 (d, J = 8.2 Hz, 2 H, ArH), 7.50 (d, J = 8.2 Hz, 2 H, ArH), 5.66 (dt, J = 15.3, 6.5 Hz, 1 H, =CH), 5.56 (dt, J = 15.4, 6.4 Hz, 1 H, =CH), 3.58 (dd, J = 7.5, 5.7 Hz, 1 H, NCH), 3.46 (dd, J = 12.8, 5.8 Hz, 1 H, one proton of NCH_2), 3.01 (dd, J = 12.7, 7.3 Hz, 1 H, one proton of NCH_2), 2.86 (td, J = 8.6, 4.8 Hz, 1 H, one proton of NCH_2), 2.55-2.46 (m, 1 H, one proton of NCH_2), 2.26-2.15 (m, 1 H, one proton of CH_2), 2.07-1.89 (m, 4 H, CH_2 and 2×one proton of CH_2), 1.89-1.77 (m, 1 H, one proton of CH_2), 1.41-1.18 (m, 12 H, 6×CH_2), 0.88 (t, J = 6.9 Hz, 3 H, CH_3); ^{13}C NMR (100 MHz, $CDCl_3$) δ 134.3, 132.1, 131.9, 128.3, 126.5, 118.5, 111.1, 94.0, 83.0, 55.4, 54.4, 51.7, 32.3, 31.8, 31.6, 29.4, 29.2, 29.14, 29.10, 22.6, 22.1, 14.1; MS (ESI) *m/z* 349 ($[M+H]^+$); IR (neat) v = 2964, 2926, 2854, 2806, 2228, 1604, 1500, 1461, 1406, 1377, 1354, 1319, 1270, 1256, 1178, 1139, 1107 cm^{-1}; HRMS calcd for $C_{24}H_{33}N_2$ ($[M+H]^+$): 349.2638. Found: 349.2640.

(10)(*E*)-2-((4-Acetylphenyl)ethynyl)-1-(2-undecenyl)pyrrolidine (**3aj**) (lwl-9-68)

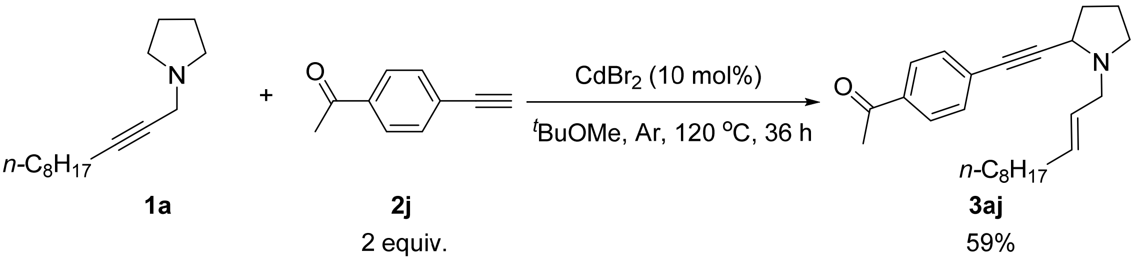

Following **Typical Procedure I**, the reaction of $CdBr_2$ (27.5 mg, 0.1 mmol), **1a** (221.5 mg, 1.0 mmol), 4-acetylphenylacetylene **2j** (288.7 mg, 2.0 mmol), and MTBE (6.0 mL) afforded **3aj** (215.7 mg, 59%) (eluent: petroleum ether/ acetone = 40/1) as a liquid: ^{1}H NMR (400 MHz, $CDCl_3$) δ 7.89 (d, J = 8.0 Hz, 2 H, ArH), 7.50 (d, J = 7.6 Hz, 2 H, ArH), 5.67 (dt, J = 15.2, 6.6 Hz, 1 H, =CH), 5.58 (dt, J = 15.2, 6.8 Hz, 1 H, =CH), 3.59 (t, J = 6.2 Hz, 1 H, NCH), 3.48 (dd, J = 12.8, 5.6 Hz, 1 H, one proton of NCH_2), 3.02 (dd, J = 12.6, 7.4 Hz, 1 H, one proton of NCH_2), 2.90-2.81 (m, 1 H, one

proton of NCH_2), 2.59 (s, 3 H, CH_3), 2.51 (q, J = 7.9 Hz, 1 H, one proton of NCH_2), 2.26-2.14 (m, 1 H, one proton of CH_2), 2.08-1.75 (m, 5 H, 2×CH_2 and one proton of CH_2), 1.43-1.15 (m, 12 H, 6×CH_2), 0.87 (t, J = 6.2 Hz, 3 H, CH_3); ^{13}C NMR (100 MHz, $CDCl_3$) δ 197.2, 135.9, 134.2, 131.7, 128.3, 128.1, 126.6, 92.7, 83.8, 55.4, 54.5, 51.7, 32.3, 31.8, 31.6, 29.4, 29.2, 29.15, 29.08, 26.5, 22.6, 22.0, 14.0; MS (EI) *m/z* (%) 365 (M^+, 16.03), 83 (100); IR (neat) v = 2955, 2922, 2853, 2804, 1685, 1600, 1460, 1440, 1403, 1356, 1318, 1283, 1260, 1178, 1138, 1109, 1015 cm^{-1}; HRMS calcd for $C_{25}H_{36}ON^+$ ($M+H^+$): 366.2791. Found: 366.2790.

(11) (*E*)-2-(Decynyl)-1-(2-undecenyl)pyrrolidine (**3ak**) (lwl-8-1)

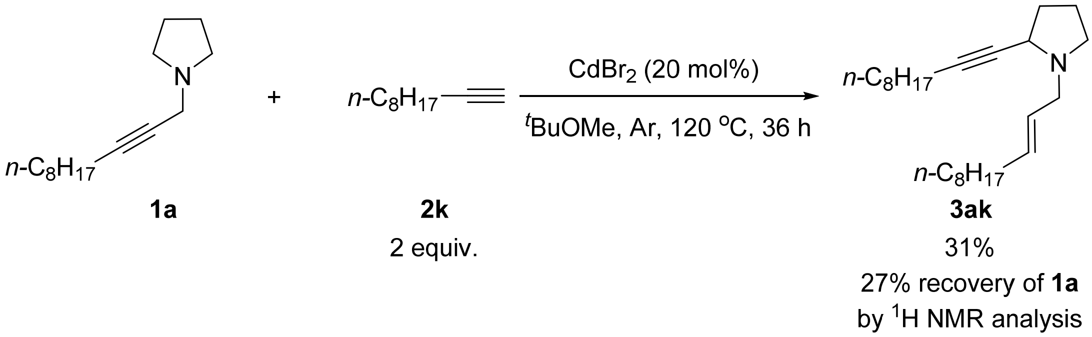

Following **Typical Procedure I**, the reaction of $CdBr_2$ (54.5 mg, 0.2 mmol) **1a** (220.5 mg, 1.0 mmol), 1-decyne **2k** (278.1 mg, 2.0 mmol), and MTBE (6.0 mL) afforded **3ak** (111.4 mg, 31%) (27% recovery of **1a** as determined by ^1H NMR analysis of the crude product using CH_2Br_2 as internal standard) (eluent: petroleum ether/ ethyl acetate/ dichloromethane/ aqueous ammonia = 20/1/1/0.08) as a liquid: ^1H NMR (400 MHz, $CDCl_3$) δ 5.63 (dt, J = 15.2, 6.4 Hz, 1 H, =CH), 5.54 (dt, J = 15.6, 6.0 Hz, 1 H, =CH), 3.44 (dd, J = 12.8, 5.6 Hz, 1 H, one proton of NCH_2), 3.30-3.21 (m, 1 H, NCH), 2.87 (dd, J = 14.8, 7.6 Hz, 1 H, one proton of NCH_2), 2.79 (td, J = 8.5, 4.8 Hz, 1 H, one proton of NCH_2), 2.37 (td, J = 8.9, 6.1 Hz, 1 H, one proton of NCH_2), 2.19 (td, J = 7.2, 2.0 Hz, 2 H, CH_2), 2.11-1.96 (m, 3 H, CH_2 and one proton of CH_2), 1.91-1.79 (m, 2 H, CH_2), 1.79-1.67 (m, 1 H, one proton of CH_2), 1.55-1.45 (m, 2 H, CH_2), 1.43-1.18 (m, 22 H, CH_2×11), 0.88 (t, J = 6.6 Hz, 6 H, 2×CH_3); ^{13}C NMR (100 MHz, $CDCl_3$) δ 133.7, 126.9, 84.6, 79.0, 55.2, 54.3, 51.4, 32.3, 31.84, 31.80, 29.4, 29.3, 29.2, 29.12, 29.06, 28.9, 28.8, 22.63, 22.62, 21.8, 18.7, 14.1; MS (EI) *m/z* (%)

359 (M^+, 55.37), 246 (100); IR (neat) v = 2925, 2854, 2809, 1494, 1443, 1377, 1354, 1320, 1184, 1143, 1107 cm^{-1}; HRMS calcd for $C_{25}H_{45}N$ (M^+): 359.3552. Found: 359.3546.

(12) (*E*)-2-(Cyclohexylethynyl)-1-(2-undecenyl)pyrrolidine (**3al**) (lwl-7-200)

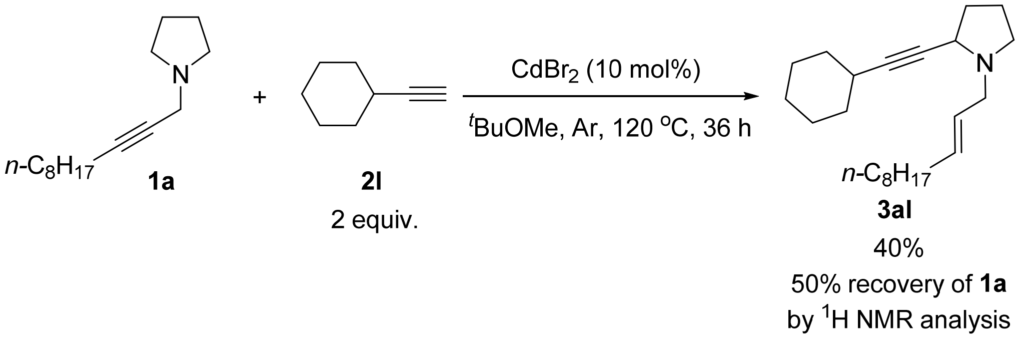

Following **Typical Procedure I**, the reaction of $CdBr_2$ (27.5 mg, 0.1 mmol), **1a** (221.5 mg, 1.0 mmol), cyclohexylacetylene **2l** (216.1 mg, 2.0 mmol), and MTBE (6.0 mL) afforded **3al** (131.5 mg, 40%) (50% recovery of **1a** as determined by ^1H NMR analysis of the crude product using CH_2Br_2 as internal standard) (eluent: petroleum ether/ ethyl acetate/ dichloromethane/ aqueous ammonia = 20/1/1/0.08) as a liquid: ^1H NMR (400 MHz, $CDCl_3$) δ 5.63 (dt, J = 15.3, 6.4 Hz, 1 H,=CH), 5.54 (dt, J = 15.3, 6.4 Hz, 1 H, =CH), 3.43 (dd, J = 12.5, 5.5 Hz, 1 H, one proton of NCH_2), 3.29 (t, J = 5.4 Hz, 1 H, NCH), 2.88 (dd, J = 12.7, 7.2 Hz, 1 H, one proton of NCH_2), 2.78 (td, J = 8.8, 4.9 Hz, 1 H, one proton of NCH_2), 2.44-2.33 (m, 2 H, CH and one proton of NCH_2), 2.11-1.96 (m, 3 H, CH_2 and one proton of CH_2), 1.91-1.64 (m, 7 H, 3×CH_2 and one proton of CH_2), 1.54-1.14 (m, 18 H, 9×CH_2), 0.88 (t, J = 6.7 Hz, 3 H, CH_3); ^{13}C NMR (100 MHz, $CDCl_3$) δ 133.8, 126.9, 88.9, 78.8, 55.1, 54.2, 51.4, 32.91, 32.87, 32.3, 31.9, 31.8, 29.4, 29.3, 29.2, 29.1, 29.0, 25.9, 24.8, 22.6, 21.7, 14.1; MS (EI) *m/z* (%) 329 (M^+, 45.69), 176 (100); IR (neat) v = 2927, 2853, 2809, 1448, 1377, 1362, 1351, 1319, 1298, 1234, 1184, 1141, 1111 cm^{-1}; HRMS calcd for $C_{23}H_{39}N$ (M^+): 329.3083. Found: 329.3080.

(13) (*E*)-2-((trimethylsilyl)ethynyl)-1-(2-undecenyl)pyrrolidine (**3am**) (cyf-1-017)

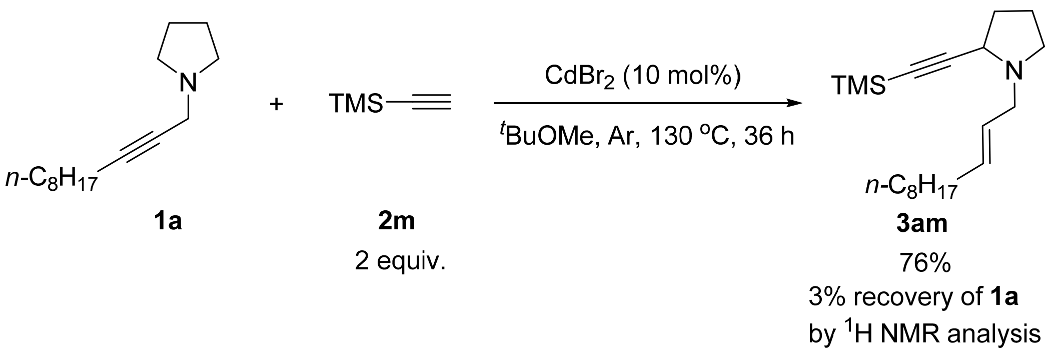

Typical Procedure II: To a dry Schlenk tube with a polytetrafluoroethylene plug was added $CdBr_2$ (27.6 mg, 0.1 mmol). Then the catalyst was dried under vacuum with heating. **1a** (222.0 mg, 1.0 mmol), MTBE (3 mL), trimethylsilylacetylene **2m** (201.6 mg, 2.0 mmol), and MTBE (3 mL) were then added sequentially under Ar atmosphere. The resulting mixture was stirred at 130 °C for 36 h. After the completion of the reaction as monitored by TLC, the crude reaction mixture was filtrated through a short column of silica gel eluted with EtOAc (50 mL). After evaporation, the residue was purified by column chromatography on silica gel to afford **3am** (242.4 mg, 76%) (3% recovery of 1a as determined by ^{1}H NMR analysis of the crude product using CH_2Br_2 as internal standard) (eluent: petroleum ether/ ethyl acetate = 100/1 (300 mL) to petroleum ether/ acetone = 100/1 (400 mL)) as a liquid: ^{1}H NMR (400 MHz, $CDCl_3$) δ 5.63 (dt, *J* = 15.2, 6.4 Hz, 1 H, =CH), 5.54 (dt, *J* = 15.2, 6.6 Hz, 1 H, =CH), 3.41 (dd, *J* = 12.8, 5.4 Hz, 1 H, one proton of NCH_2), 3.33 (dd, *J* = 7.0, 5.4 Hz, 1 H, NCH) , 2.92 (dd, *J* = 12.4, 7.6 Hz, 1 H, one proton of NCH_2), 2.82-2.70 (m, 1 H, one proton of NCH_2), 2.43 (dd, *J* = 14.6, 7.8 Hz, 1 H, one proton of NCH_2), 2.15-1.97 (m, 3 H, CH_2 and one proton of CH_2), 1.96-1.66 (m, 3 H, CH_2 and one proton of CH_2), 1.46-1.16 (m, 12 H, 6×CH_2), 0.88 (t, *J* = 6.8 Hz, 3 H, CH_3), 0.16 (s, 9 H, 3×$SiCH_3$); ^{13}C NMR (100 MHz, $CDCl_3$) δ 134.1, 126.7, 105.4, 88.6, 55.1, 54.5, 51.4, 32.3, 31.8, 31.6, 29.4, 29.3, 29.2, 29.1, 22.6, 21.9, 14.1, 0.1; MS (EI) *m/z* (%) 319 (M^+, 16.60), 139 (100); IR (neat) *v* = 2957, 2923, 2853, 2811, 2158, 1460, 1376, 1350, 1317, 1249, 1182, 1138, 1117, 1090 cm^{-1}; HRMS calcd for $C_{20}H_{37}NSi$ (M^+): 319.2695. Found: 319.2690.

(14) (*E*)-1-(3-Cyclohexylallyl)-2-(phenylethynyl)pyrrolidine (**3ba**) (lwl-8-51)

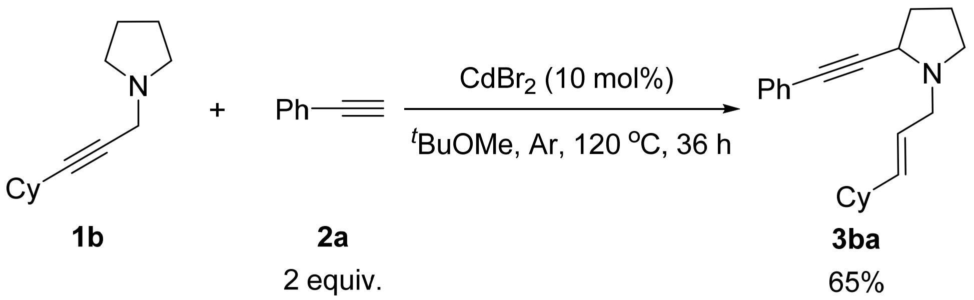

To a dry Schlenk tube with a polytetrafluoroethylene plug was added $CdBr_2$ (27.5 mg, 0.1 mmol). Then the catalyst was dried under vacuum with heating. **1b** (191.2 mg, 1.0 mmol), MTBE (3 mL), phenylacetylene **2a** (204.3 mg, 2.0 mmol), and MTBE (3 mL) were then added sequentially under Ar atmosphere. The resulting mixture was stirred at 120 °C for 36 h. After the completion of the reaction as monitored by TLC, the crude reaction mixture was filtrated through a short column of Al_2O_3 eluted with EtOAc (50 mL). After evaporation, the residue was purified by column chromatography on neutral Al_2O_3 to afford **3ba** (190.2 mg, 65%) (eluent: petroleum ether/ ethyl acetate/ dichloromethane = 100/1/5) as a liquid: ^{1}H NMR (400 MHz, $CDCl_3$) δ 7.47-7.38 (m, 2 H, ArH), 7.33-7.23 (m, 3 H, ArH), 5.63 (dd, *J* = 15.4, 6.2 Hz, 1 H, =CH), 5.54 (dt, *J* = 15.3, 6.4 Hz, 1 H, =CH), 3.58 (dd, *J* = 7.4, 5.5 Hz, 1 H, NCH), 3.49 (dd, *J* = 12.8, 5.8 Hz, 1 H, one proton of NCH_2), 3.01 (dd, *J* = 12.8, 7.2 Hz, 1 H, one proton of NCH_2), 2.85 (td, *J* = 8.8, 5.2 Hz, 1 H, one proton of NCH_2), 2.55-2.45 (m, 1 H, one proton of NCH_2), 2.24-2.12 (m, 1 H, one proton of CH_2), 2.07-1.86 (m, 3 H, CH_2 and one proton of CH_2), 1.86-1.58 (m, 5 H, 2×CH_2 and one proton of CH_2), 1.33-1.00 (m, 5 H, 2×CH_2 and one proton of CH_2), 0.91-0.78 (m, 1 H, one proton of CH_2); ^{13}C NMR (100 MHz, $CDCl_3$) δ 139.9, 131.6, 128.1, 127.8, 124.0, 123.3, 88.6, 84.5, 55.4, 54.5, 51.5, 40.4, 32.8, 32.8, 31.6, 26.1, 26.0, 21.9; MS (EI) *m/z* (%) 293 (M^+, 32.37), 210 (100); IR (neat) 2922, 2850, 2807, 1598, 1489, 1445, 1377, 1356, 1319, 1257, 1138, 1115, 1069 cm^{-1}; HRMS calcd for $C_{21}H_{27}N$ (M^+): 293.2144. Found: 293.2138.

(15) (*E*)-1-(2-Heptenyl)-2-(phenylethynyl)pyrrolidine (**3ca**) (cyf-1-012)

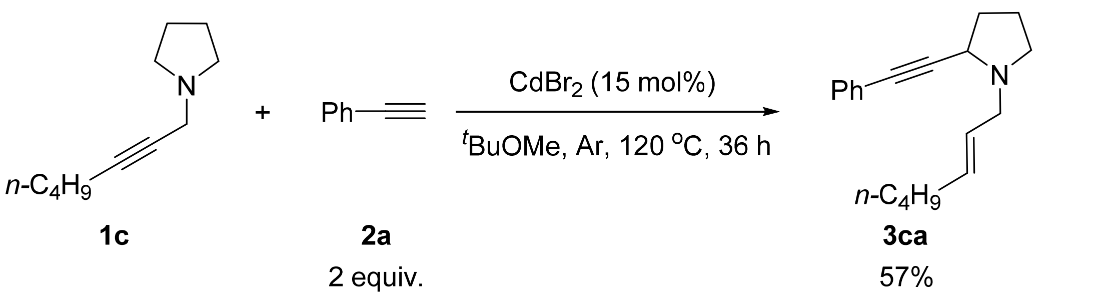

Following **Typical Procedure II**, the reaction of $CdBr_2$ (41.5 mg, 0.15 mmol), **1c** (165.0 mg, 1.0 mmol), phenylacetylene **2a** (210.5 mg, 2.0 mmol), and MTBE (6.0 mL) afforded **3ca** (152.2 mg, 57%) (eluent: petroleum ether/ acetone = 200/1 (450 mL) to 50/1 (250 mL)) as a liquid: ^{1}H NMR (400 MHz, $CDCl_3$) δ 7.48-7.37 (m, 2 H, ArH), 7.32-7.23 (m, 3 H, ArH), 5.72-5.52 (m, 2 H, 2×=CH), 3.56 (t, J = 6.4 Hz, 1 H, NCH), 3.48 (dd, J = 12.4, 5.2 Hz, 1 H, one proton of NCH_2), 3.01 (dd, J = 12.6, 7.0 Hz, 1 H, one proton of NCH_2), 2.88-2.79 (m, 1 H, one proton of NCH_2), 2.50 (dd, J = 15.4, 8.2 Hz, 1 H, one proton of NCH_2), 2.24-2.11 (m, 1 H, one proton of CH_2), 2.08-1.87 (m, 4 H, CH_2 and 2×one proton of CH_2), 1.85-1.73 (m, 1 H, one proton of CH_2), 1.41-1.19 (m, 4 H, 2×CH_2), 0.88 (t, J = 6.6 Hz, 3 H, CH_3); ^{13}C NMR (100 MHz, $CDCl_3$) δ 133.9, 131.6, 128.1, 127.8, 126.8, 123.3, 88.7, 84.5, 55.3, 54.5, 51.5, 31.9, 31.7, 31.3, 22.1, 22.0, 13.8; MS (EI) *m/z* (%) 267 (M^+, 14.93), 83 (100); IR (neat) *ν* = 2955, 2923, 2871, 2804, 1598, 1489, 1459, 1442, 1355, 1320, 1255, 1138, 1109, 1069 cm^{-1}; HRMS calcd for $C_{19}H_{25}N$ (M^+): 267.1987. Found: 267.1990.

(16) (*E*)-1-(4-methyl-4-hydroxyl-2-pentenyl)-2-(phenylethynyl)pyrrolidine (**3da**) (lwl-9-70)

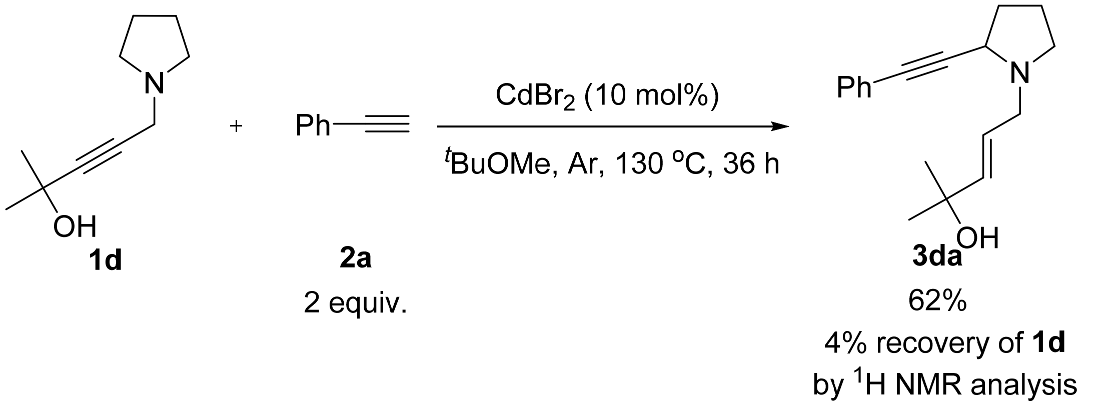

Following **Typical Procedure I**, the reaction of $CdBr_2$ (27.2 mg, 0.1 mmol), **1d**

(167.0 mg, 1.0 mmol), phenylacetylene **2a** (204.5 mg, 2.0 mmol), and MTBE (6.0 mL) afforded **3da** (166.8 mg, 62%) (4% recovery of **1d** as determined by ^{1}H NMR analysis of the crude product using CH_2Br_2 as internal standard) (eluent: petroleum ether/ acetone = 20/1 to 10/1) as a liquid: ^{1}H NMR (400 MHz, $CDCl_3$) δ 7.46-7.39 (m, 2 H, ArH), 7.34-7.26 (m, 3 H, ArH), 5.91-5.74 (m, 2 H, 2×=CH), 3.62-3.46 (m, 2 H, NCH and one proton of CH_2), 3.08 (dd, J = 13.0, 6.6 Hz, 1 H, one proton of NCH_2), 2.87 (dd, J = 13.6, 8.4 Hz, 1 H, one proton of NCH_2), 2.51 (dd, J = 16.0, 8.0 Hz, 1 H, one proton of NCH_2), 2.26-2.12 (m, 1 H, one proton of CH_2), 2.09-1.75 (m, 4 H, OH and 3×one proton of CH_2), 1.32 (s, 6 H, 2×CH_3); ^{13}C NMR (100 MHz, $CDCl_3$) δ 141.0, 131.6, 128.2, 127.9, 123.9, 123.2, 88.6, 54.7, 70.5, 54.9, 54.7, 51.8, 31.7, 29.61, 29.59, 22.0; MS (ESI) m/z 270 ($[M+H]^+$); IR (neat) v = 3371, 2970, 2876, 2805, 1598, 1489, 1460, 1443, 1358, 1326, 1233, 1148, 1070 cm^{-1}; HRMS calcd for $C_{18}H_{24}ON$ ($[M+H]^+$): 270.1852. Found: 270.1851.

(17) (*E*)-1-cinnamyl-2-(phenylethynyl)pyrrolidine (**3ga**) (cyf-2-103)

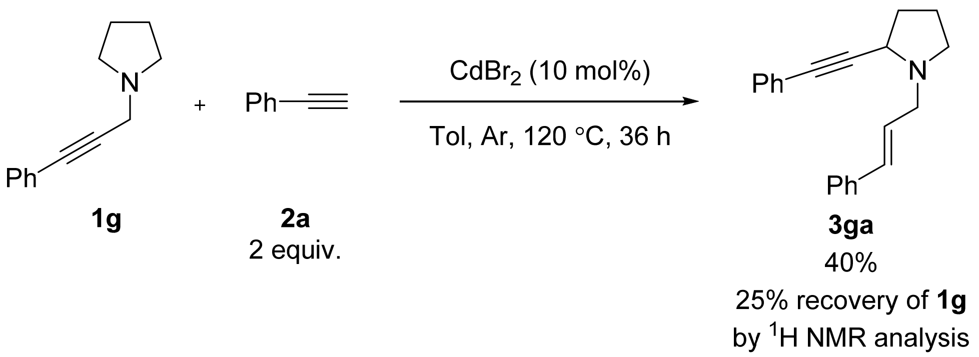

Following **Typical Procedure I**, the reaction of $CdBr_2$ (27.7 mg, 0.1 mmol), **1g** (185.4 mg, 1.0 mmol), phenylacetylene **2a** (211.9 mg, 2.0 mmol), and toluene (6.0 mL) afforded **3ga** (115.0 mg, 40%) (25% recovery of **1g** as determined by ^{1}H NMR analysis of the crude product using CH_2Br_2 as internal standard) (eluent: petroleum ether/ acetone = 40/1) as a liquid: ^{1}H NMR (400 MHz, $CDCl_3$) δ 7.48-7.40 (m, 2 H, ArH), 7.40-7.34 (m, 2 H, ArH), 7.32-7.23 (m, 5 H, ArH), 7.23-7.16 (m, 1 H, ArH), 6.59 (d, J = 16.0 Hz, 1 H, =CH), 6.38 (ddd, J = 16.0, 7.4, 6.2 Hz, 1 H, =CH), 3.69 (ddd, J = 13.6, 6.4, 1.1 Hz, 1 H, NCH), 3.62 (dd, J = 7.4, 5.8 Hz, 1 H, one proton of NCH_2), 3.24 (dd, J = 13.4, 7.4 Hz, 1 H, one proton of NCH_2), 2.91 (td, J = 8.8, 4.9 Hz,

1 H, one proton of NCH_2), 2.55 (td, *J* = 8.8, 6.4 Hz, 1 H, one proton of NCH_2), 2.25-2.13 (m, 1 H, one proton of CH_2), 2.09-1.88 (m, 2 H, 2×one proton of CH_2), 1.87-1.75 (m, 1 H, one proton of CH_2); ^{13}C NMR (100 MHz, $CDCl_3$) δ 137.2, 132.4, 131.8, 128.6, 128.3, 128.0, 127.41, 127.39, 126.4, 123.4, 88.7, 84.8, 55.6, 54.9, 52.0, 31.9, 22.2; MS (ESI) *m/z* 288 ($[M+H]^+$); IR (neat) *ν* = 3081, 3058, 3025, 2953, 2909, 2875, 2798, 1598, 1574, 1489, 1443, 1354, 1320, 1264, 1213, 1179, 1113, 1069, 1027 cm^{-1}; HRMS calcd for $C_{21}H_{22}N$ ($[M+H]^+$): 288.1747. Found: 288.1745.

3. The ZnI_2-catalyzed reaction of propargylic amines with terminal alkynes

(1) 1-Allyl-2-(phenylethynyl)pyrrolidine (**3ea**) (lwl-8-53)

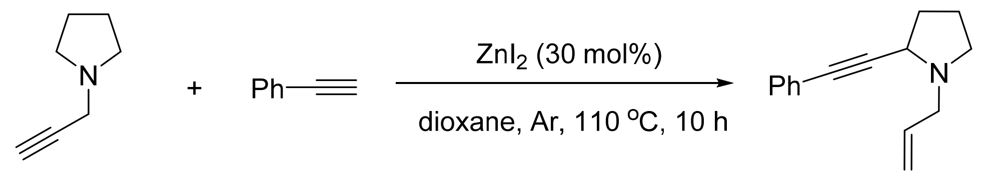

Typical Procedure III: To a dry Schlenk tube with a polytetrafluoroethylene plug was added ZnI_2 (95.8 mg, 0.3 mmol). Then the catalyst was heated under vacuum until sublimation of ZnI_2. After cooling to room temperature, **1e** (109.2 mg, 1.0 mmol), dioxane (3 mL), phenylacetylene **2a** (204.3 mg, 2.0 mmol), and dioxane (3 mL) were added sequentially under Ar atmosphere. Then the Schenk tube was stirred at 110 °C for 10 h. After the completion of the reaction as monitored by TLC, the crude reaction mixture was transferred with EtOAc (50 mL). After evaporation, the residue was purified by columm chromatography on basic Al_2O_3 to afford **3ea** (101.1 mg, 47%) (eluent: petroleum ether/ ethyl acetate/ dichloromethane = 100/1/5) as a liquid: ^1H NMR (400 MHz, $CDCl_3$) δ 7.52-7.38 (m, 2 H, ArH), 7.35-7.25 (m, 3 H, ArH), 6.05-5.90 (m, 1 H, =CH), 5.26 (d, *J* = 17.2 Hz, 1 H, one proton of $=CH_2$), 5.13 (d, *J* = 10.0 Hz, 1 H, one proton of $=CH_2$), 3.67-3.52 (m, 2 H, NCH and one proton of NCH_2), 3.06 (dd, *J* = 13.4, 7.6 Hz, 1 H, one proton of NCH_2), 2.86 (td, *J* = 8.8, 5.2 Hz, 1 H, one proton of NCH_2), 2.51 (td, *J* = 8.8, 6.4 Hz, 1 H, one proton of NCH_2), 2.26-2.12

(m, 1 H, one proton of CH_2), 2.09-1.88 (m, 2 H, CH_2), 1.88-1.75 (m, 1 H, one proton of CH_2); ^{13}C NMR (100 MHz, $CDCl_3$) δ 135.6, 131.6, 128.1, 127.9, 123.2, 117.2, 88.5, 84.6, 56.2, 54.7, 51.6, 31.7, 22.0; MS (EI) *m/z* (%) 211 (M^+, 42.03), 210 (100); IR (neat) *v* = 3078, 3008, 2976, 2911, 2876, 2806, 1643, 1598, 1489, 1459, 1443, 1419, 1357, 1320, 1182, 1141, 1110, 1070 cm^{-1}; HRMS calcd for $C_{15}H_{17}N$ (M^+): 211.1361. Found: 211.1359.

(2) 1-Allyl-2-((4-methoxyphenyl)ethynyl)pyrrolidine (**3ec**) (lwl-8-54)

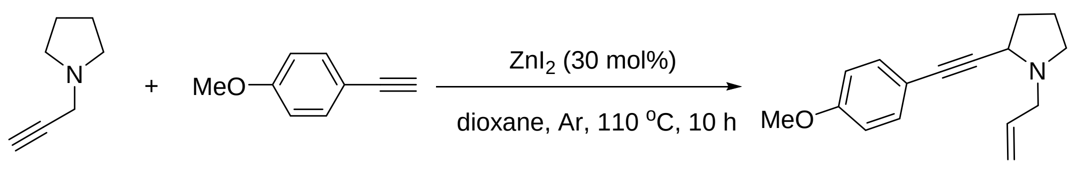

1e **2c** 2 equiv. **3ec** 45%

Following **Typical Procedure III**, the reaction of ZnI_2 (95.8 mg, 0.3 mmol), **1e** (109.2 mg, 1.0 mmol), 4-methoxyphenylacetylene **2c** (264.3 mg, 2.0 mmol), and dioxane (6.0 mL) afforded **3ec** (108.7.0 mg, 45%) (eluent: petroleum ether/ ethyl acetate/ dichloromethane = 100/1/5) as a liquid: ^{1}H NMR (400 MHz, $CDCl_3$) δ 7.37 (dt, *J* = 8.8, 2.5 Hz, 2 H, ArH), 7.35 (dt, *J* = 8.8, 2.4 Hz, 2 H, ArH), 6.03-5.91 (m, 1 H, =CH), 5.25 (dd, *J* = 17.2, 1.2 Hz, 1 H, one proton of =CH_2), 5.13 (d, *J* = 10.0 Hz, 1 H, one proton of =CH_2), 3.79 (s, 3 H, CH_3), 3.62-3.52 (m, 2 H, NCH and one proton of NCH_2), 3.04 (dd, *J* = 13.1, 7.7 Hz, 1 H, one proton of NCH_2), 2.87 (td, *J* = 8.8, 4.8 Hz, 1 H, one proton of NCH_2), 2.48 (td, *J* = 8.9, 6.3 Hz, 1 H, one proton of NCH_2), 2.23-2.12 (m, 1 H, one proton of CH_2), 2.06-1.88 (m, 2 H, CH_2), 1.87-1.75 (m, 1 H, one proton of CH_2); ^{13}C NMR (100 MHz, $CDCl_3$) δ 159.2, 135.7, 133.0, 117.2, 115.4, 113.7, 87.0, 84.3, 56.3, 55.2, 54.8, 51.7, 31.7, 22.0; MS (EI) *m/z* (%) 241 (M^+, 47.36), 240 (100); IR (neat) *v* = 3075, 2956, 2910, 2876, 2835, 2805, 1643, 1606, 1570, 1509, 1463, 1442, 1418, 1357, 1320, 1290, 1248, 1172, 1142, 1106, 1033 cm^{-1}; HRMS calcd for $C_{16}H_{19}NO$ (M^+): 241.1467. Found:241.1461.

(3) 1-Allyl-2-((4-fluorophenyl)ethynyl)pyrrolidine (**3ed**) (lwl-8-55)

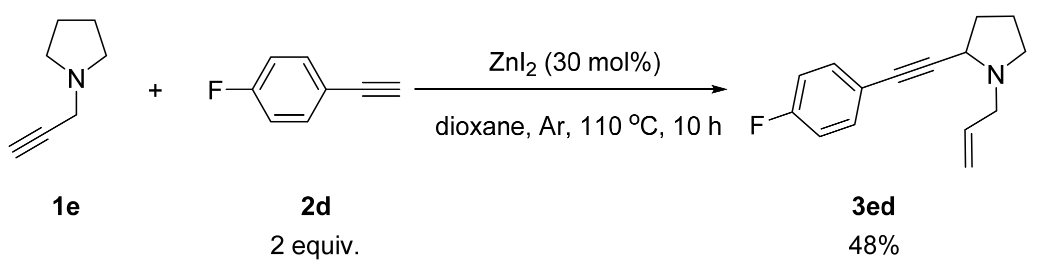

Following **Typical Procedure III**, the reaction of ZnI_2 (95.8 mg, 0.3 mmol), **1e** (109.2 mg, 1.0 mmol), 4-fluorophenylacetylene **2d** (240.2 mg, 2.0 mmol), and dioxane (6.0 mL) afforded **3ed** (110.6 mg, 48%) (eluent: petroleum ether/ ethyl acetate/ dichloromethane = 100/1/5) as a liquid: ^{1}H NMR (400 MHz, $CDCl_3$) δ 7.46-7.36 (m, 2 H, ArH), 6.98 (t, J = 8.6 Hz, 2 H, ArH), 6.03-5.90 (m, 1 H, =CH), 5.25 (dd, J = 16.8, 1.2 Hz, 1 H, one proton of $=CH_2$), 5.13 (d, J = 10.0 Hz, 1 H, one proton of $=CH_2$), 3.60-3.50 (m, 2 H, NCH and one proton of NCH_2), 3.04 (dd, J = 12.8, 7.2 Hz, 1 H, one proton of NCH_2), 2.86 (td, J = 8.9, 4.9 Hz, 1 H, one proton of NCH_2), 2.49 (td, J = 8.8, 6.4 Hz, 1 H, one proton of NCH_2), 2.24-2.12 (m, 1 H, one proton of CH_2), 2.07-1.87 (m, 2 H, CH_2), 1.87-1.75 (m, 1 H, one proton of CH_2); ^{13}C NMR (100 MHz, $CDCl_3$) δ 162.2 (d, J = 247.6 Hz), 135.6, 133.5 (d, J = 8.2 Hz), 119.3 (d, J = 2.9 Hz), 117.2, 115.4 (d, J = 21.6 Hz), 88.3 (d, J = 1.5 Hz), 83.4, 56.3, 54.7, 51.7, 31.7, 22.0; ^{19}F NMR (376 MHz, $CDCl_3$) δ -112.1; MS (EI) *m/z* (%) 229 (M^+, 43.68), 228 (100); IR (neat) v = 3077, 2977, 2912, 2877, 2806, 1643, 1601, 1506, 1460, 1444, 1419, 1357, 1321, 1231, 1156, 1110, 1092 cm^{-1}; HRMS calcd for $C_{15}H_{16}NF$ (M^+): 229.1267. Found: 229.1266.

(4) 1-Allyl-2-((4-chlorophenyl)ethynyl)pyrrolidine (**3ee**) (lwl-8-56)

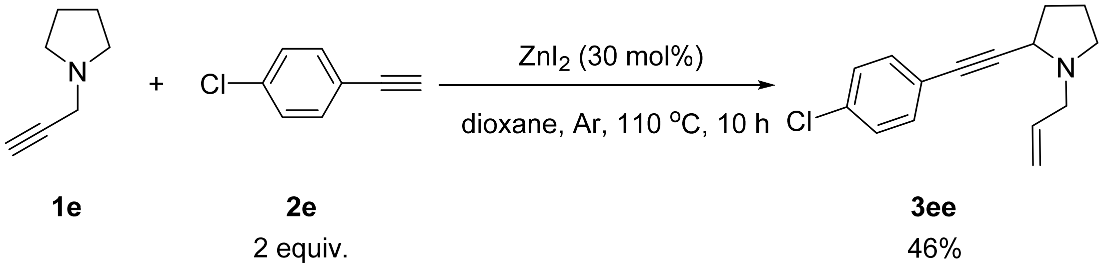

Following **Typical Procedure III**, the reaction of ZnI_2 (95.8 mg, 0.3 mmol), **1e** (109.2 mg, 1.0 mmol), 4-chlorophenylacetylene **2e** (273.2 mg, 2.0 mmol), and

dioxane (6.0 mL) afforded **3ee** (113.2 mg, 46%) (eluent: petroleum ether/ ethyl acetate/ dichloromethane = 100/1/5) as a liquid: ^{1}H NMR (400 MHz, $CDCl_3$) δ 7.35 (d, *J* = 8.4 Hz, 2 H, ArH), 7.26 (d, *J* = 8.4 Hz, 2 H, ArH), 6.03-5.90 (m, 1 H, =CH), 5.25 (dd, *J* = 17.2, 1.2 Hz, 1 H, one proton of $=CH_2$), 5.13 (d, *J* = 10.0 Hz, 1 H, one proton of $=CH_2$), 3.61-3.50 (m, 2 H, NCH and one proton of NCH_2), 3.04 (dd, *J* = 13.2, 7.6 Hz, 1 H, one proton of NCH_2), 2.86 (td, *J* = 8.7, 4.9 Hz, 1 H, one proton of NCH_2), 2.49 (td, *J* = 8.8, 6.4 Hz, 1 H, one proton of NCH_2), 2.24-2.13 (m, 1 H, one proton of NCH_2), 2.07-1.87 (m, 2 H, CH_2), 1.87-1.75 (m, 1 H, one proton of CH_2); ^{13}C NMR (100 MHz, $CDCl_3$) δ 135.5, 133.8, 132.8, 128.5, 121.7, 117.3, 89.7, 83.4, 56.3, 54.7, 51.7, 31.6, 22.0; MS (EI) *m/z* (%) 247 ($M^+(Cl^{37})$, 13.82), 245 ($M^+(Cl^{35})$, 42.31), 244 (100); IR (neat) *ν* = 3078, 2977, 2957, 2914, 2876, 2805, 1643, 1490, 1460, 1443, 1419, 1398, 1356, 1319, 1255, 1181, 1141, 1092, 1014 cm^{-1}; HRMS calcd for $C_{15}H_{16}NCl$ ($M^+(Cl^{35})$): 245.0971. Found: 245.0979.

4. The ZnI_2-catalyzed reaction of 1-propargylic 1,2,3,4-tetrahydroisoquinoline with terminal alkynes

(1) (*E*)-1-(phenylethynyl)-2-(2-undecenyl)-1,2,3,4-tetrahydroisoquinoline (**3fa**) (cyf-1-136)

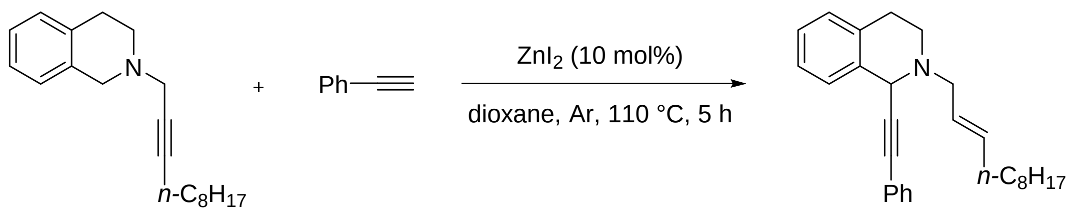

1f **2a** 2 equiv. **3fa** 78%

Typical Procedure IV: To a dry Schlenk tube with a polytetrafluoroethylene plug was added ZnI_2 (32.3 mg, 0.1 mmol). Then the catalyst was dried with heating under vacuum. After cooling to room temperature, **2a** (211.0 mg, 2.0 mmol), dioxane (3 mL), **1f** (283.0 mg, 1.0 mmol), and dioxane (3 mL) were added sequentially under Ar atmosphere. Then the Schenk tube was stirred at 110 °C for 5 hours. After the completion of the reaction as monitored by TLC, the crude reaction mixture was

diluted with 5 mL of dichloromethane and filtered through a short column of silica gel (eluent: 50 mL of Et_2O). After evaporation, the residue was purified by column chromatography on silica gel to afford **3fa** (301.4 mg, 78%) (eluent: petroleum ether/ diethyl ether/ dicholoromethane = 40/1/1) as a liquid: ^{1}H NMR (400 MHz, $CDCl_3$) δ 7.44-7.37 (m, 2 H, ArH), 7.34-7.23 (m, 4 H, ArH), 7.21-7.09 (m, 3 H, ArH), 5.76 (dt, *J* = 15.2, 6.8 Hz, 1 H, =CH), 5.57 (td, *J* = 15.2, 6.8 Hz, 1 H, =CH), 4.88 (s, 1 H, NCH), 3.40-3.29 (m, 2 H, CH_2), 3.08-2.96 (m, 2 H, CH_2), 2.89-2.77 (m, 2 H, CH_2), 2.08 (q, *J* = 6.8 Hz, 2 H, CH_2), 1.46-1.20 (m, 12 H, 6×CH_2), 0.88, (t, *J* = 6.6 Hz, 3 H, CH_3); ^{13}C NMR (100 MHz, $CDCl_3$) δ 135.40, 135.33, 134.0, 131.7, 129.0, 128.1, 127.9, 127.8, 126.9, 126.2, 125.8, 123.2, 87.4, 86.6, 57.6, 54.3, 45.4, 32.4, 31.9, 29.5, 29.3, 29.22, 29.17, 28.8, 22.7, 14.1; MS (EI) *m/z* (%) 385 (M^+, 9.93), 145 (100); IR (neat) *ν* = 2954, 2922, 2852, 2820, 1598, 1489, 1454, 1356, 1321, 1286, 1261, 1189, 1133, 1099, 1070, 1050, 1028, 1007 cm^{-1}; HRMS calcd for $C_{28}H_{35}N$ (M^+): 385.2770. Found: 385.2772.

(2) (*E*)-1-((trimethylsilyl)ethynyl)-2-(2-undecenyl)-1,2,3,4-tetrahydroisoquinoline (**3fm**) (cyf-1-137B)

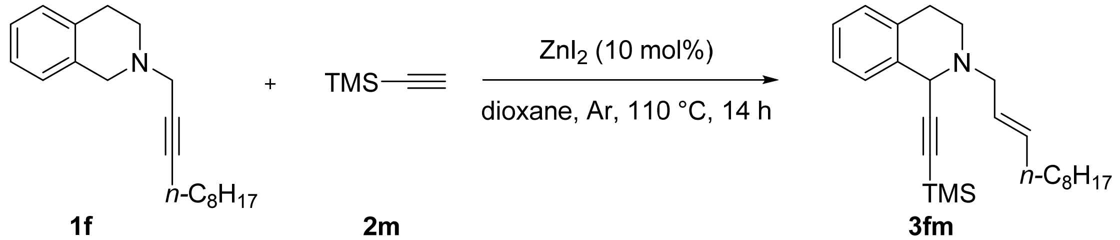

1f **2m** 2 equiv. **3fm** 73%

Following **Typical Procedure IV**, the reaction of ZnI_2 (32.4 mg, 0.1 mmol), trimethylsilylacetylene **2m** (200.9 mg, 2.0 mmol), **1f** (283.7 mg, 1.0 mmol), and dioxane (6.0 mL) afforded **3fm** (277.3 mg, 73%) (eluent: petroleum ether/ diethyl ether/ dicholoromethane = 30/1/1) as a liquid: ^{1}H NMR (400 MHz, $CDCl_3$) δ 7.27-7.19 (m, 1 H, ArH), 7.19-7.04 (m, 3 H, ArH), 5.72 (td, *J* = 15.8, 6.8 Hz, 1 H, =CH), 5.53 (td, *J* = 15.2, 6.8 Hz, 1 H, =CH), 4.65 (s, 1 H, NCH), 3.33-3.19 (m, 2 H, CH_2), 3.02-2.88 (m, 2 H, CH_2), 2.83-2.70 (m, 2 H, CH_2), 2.07 (q, *J* = 7.1 Hz, 2 H, CH_2), 1.46-1.18 (m, 12 H, 6×CH_2), 0.89 (t, *J* = 6.8 Hz, 3 H, CH_3), 0.15 (m, 9 H,

$3\times SiCH_3$); ^{13}C NMR (100 MHz, $CDCl_3$) δ 135.3, 135.1, 133.9, 128.9, 127.8, 126.8, 126.1, 125.7, 103.4, 90.6, 57.4, 54.4, 45.3, 32.4, 31.9, 29.5, 29.3, 29.22, 29.15, 28.7, 22.7, 14.1, 0.2; MS (EI) *m/z* (%) 381 ($[M]^+$, 29.89), 228 (100); IR (neat) *ν* = 2956, 2922, 2853, 2821, 2156, 1494, 1454, 1356, 1321, 1286, 1261, 1189, 1133, 1099, 1076, 1070 cm^{-1}; HRMS calcd for $C_{25}H_{39}NSi$ (M^+): 381.2852. Found: 381.2844.

(3) (*E*)-1-(1-hexynyl)-2-(2-undecenyl)-1,2,3,4-tetrahydroisoquinoline (**3fn**) (cyf-1-137A)

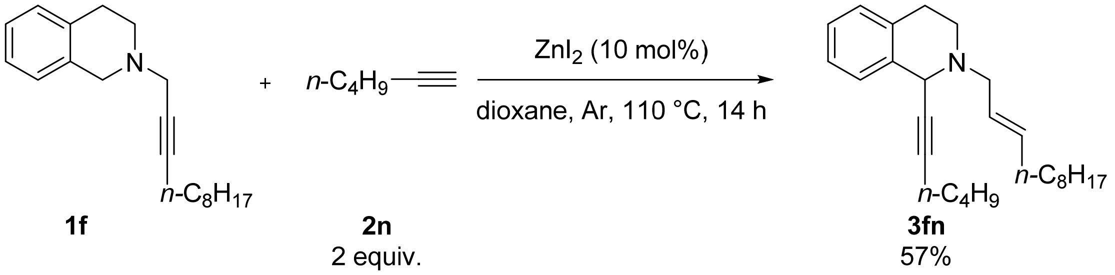

Following **Typical Procedure IV**, the reaction of ZnI_2 (32.2 mg, 0.1 mmol), 1-hexyne **2n** (169.8 mg, 2.0 mmol), **1f** (283.3 mg, 1.0 mmol), and dioxane (6.0 mL) afforded **3fn** (208.8 mg, 57%) (eluent: petroleum ether/ diethyl ether/ dicholoromethane = 40/1/1) as a liquid: ^1H NMR (400 MHz, $CDCl_3$) δ 7.28-7.19 (m, 1 H, ArH), 7.17-7.04 (m, 3 H, ArH), 5.71 (td, *J* = 15.2, 6.8 Hz, 1 H, =CH), 5.54 (td, *J* = 16.0, 6.8 Hz, 1 H, =CH), 4.62 (s, 1 H, NCH), 3.25 (qd, *J* = 13.5, 6.7 Hz, 2 H, CH_2), 3.01-2.88 (m, 2 H, CH_2), 2.85-2.69 (m, 2 H, CH_2), 2.20 (td, *J* = 6.8, 1.6 Hz, 2 H, CH_2), 2.06 (q, *J* = 6.8 Hz, 2 H, CH_2), 1.53-1.20 (m, 16 H, $8\times CH_2$), 0.93-0.83 (m, 6 H, $2\times CH_3$); ^{13}C NMR (100 MHz, $CDCl_3$) δ 136.2, 135.0, 133.7, 128.9, 127.6, 126.6, 126.3, 125.6, 86.8, 77.6, 57.4, 54.0, 45.2, 32.4, 31.9, 31.0, 29.5, 29.3, 29.23, 29.16, 28.8, 22.7, 22.0, 18.5, 14.1, 13.6; MS (EI) *m/z* (%) 365 ($[M]^+$, 22.97), 212 (100); IR (neat) *ν* = 2955, 2923, 2853, 1658, 1493, 1456, 1357, 1323, 1288, 1260, 1192, 1132, 1113, 1075, 1050 cm^{-1}; HRMS calcd for $C_{26}H_{39}N$ (M^+): 365.3083. Found: 365.3081.

5. The $CdBr_2$-catalyzed reaction of 1-(2-Undecynyl) piperidine with phenylacetylene. (*E*)-2-(Phenylethynyl)-1-(2-undecenyl)piperidine (**3ha**) (cyf-2-112)

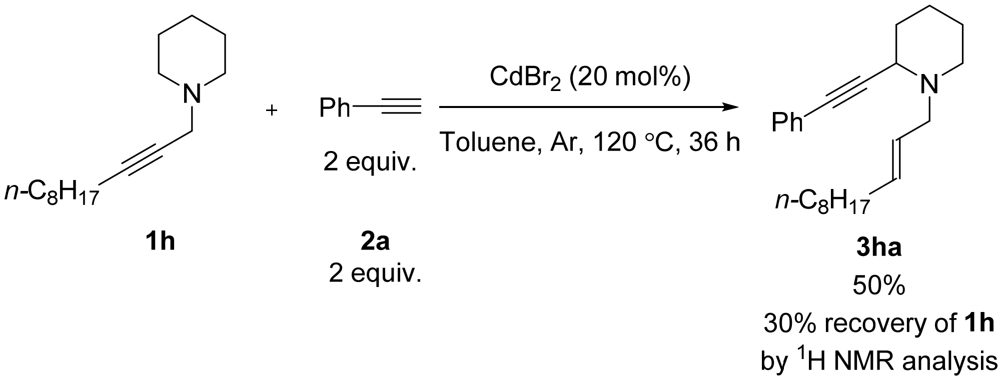

Following **Typical Procedure I**, the reaction of $CdBr_2$ (55.4 mg, 0.2 mmol), **1h** (235.2 mg, 1.0 mmol), phenylacetylene **2a** (212.6 mg, 2.0 mmol), and toluene (6.0 mL) afforded **3ha** (168.8 mg, 50%) (30% recovery of **1h** as determined by ^{1}H NMR analysis of the crude product using CH_2Br_2 as internal standard) (eluent: petroleum ether/ ethyl acetate = 50/1) as a liquid: ^{1}H NMR (400 MHz, $CDCl_3$) δ 7.49-7.41 (m, 2 H, ArH), 7.34-7.27 (m, 3 H, ArH), 5.66 (dt, J = 15.6, 7.4 Hz, 1 H, =CH), 5.49 (dt, J = 15.2, 6.8 Hz, 1 H, =CH), 3.82 (s, 1 H, NCH), 3.17 (dd, J = 12.8, 5.8 Hz, 1 H, one proton of NCH_2), 3.08 (dd, J = 13.2, 7.4 Hz, 1 H, one proton of NCH_2), 2.62-2.46 (m, 2 H, NCH_2), 2.03 (q, J = 6.9 Hz, 2 H, CH_2), 1.91-1.80 (m, 2 H, CH_2), 1.78-1.50 (m, 4 H, 2×CH_2), 1.44-1.18 (m, 12 H, 6×CH_2), 0.88 (t, J = 6.8 Hz, 3 H, CH_3); ^{13}C NMR (100 MHz, $CDCl_3$) δ 134.9, 131.7, 128.2, 127.8, 126.2, 123.5, 87.2, 86.4, 58.6, 51.7, 49.1, 32.4, 31.8, 31.4, 29.4, 29.3, 29.2, 29.1, 25.7, 22.6, 20.9, 14.1; MS (EI) *m/z* (%) 337 (M^+, 8.77), 184 (100); IR (neat) ν = 2922, 2852, 2814, 1598, 1489, 1441, 1377, 1351, 1319, 1292, 1260, 1201, 1123, 1068, 1041, 1027 cm^{-1}; HRMS calcd for $C_{24}H_{35}N$ (M^+): 337.2770. Found: 337.2760.

6. The $CdBr_2$-catalyzed reaction of propargylic amine li with terminal alkyne.

1,2-Undecadiene (**3ia**) (cyf-2-92)[4]

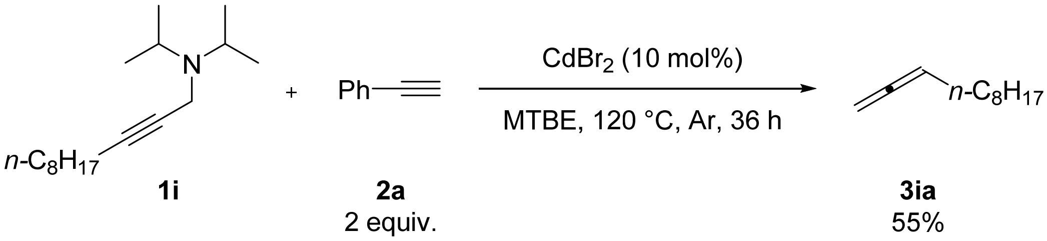

Following **Typical Procedure I**, the reaction of $CdBr_2$ (27.6 mg, 0.1 mmol), **1i**

(251.7 mg, 1.0 mmol), phenylacetylene **2a** (208.1 mg, 2.0 mmol), and MTBE (6.0 mL) afforded **3ia** (83.8 mg, 55%) (eluent: petroleum ether) as a liquid: ^{1}H NMR (400 MHz, $CDCl_3$) δ 5.01 (quint, *J* = 6.8 Hz, 1 H, =CH), 4.64 (dt, *J* = 6.4, 3.2 Hz, 2 H, =CH_2), 2.03-1.94 (m, 2 H, CH_2), 1.45-1.19 (m, 12 H, 6×CH_2), 0.88 (t, *J* = 6.8 Hz, 3 H, CH_3); ^{13}C NMR (100 MHz, $CDCl_3$) δ 208.5, 90.1, 74.5, 31.9, 29.4, 29.3, 29.14, 29.10, 28.3, 22.7, 14.1; MS (EI) *m/z* (%) 152 (M^+, 1.0), 54 (100); IR (neat) *ν* = 2957, 2923, 2854, 1957, 1463, 1378, 1260, 1096, 1016 cm^{-1}.

7. Mechanistic studies

(1) 1-(2-Undecynyl)pyrrolidine-2,2,5,5-*d*$_4$ (*d*$_4$-**1a**) (cyf-1-060)

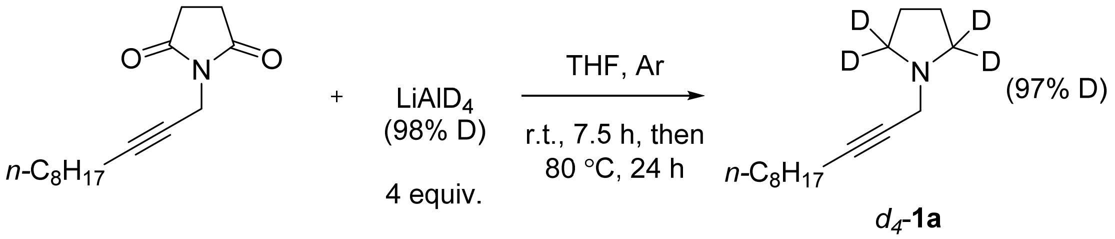

d$_4$-**1a**
45%

To a flame-dried Schlenk tube were added $LiAlD_4$ (168.5 mg, 4.0 mmol) and 4 mL of THF under Ar atmosphere. The reaction mixture was cooled to 0 °C with an ice bath and 1-(2-undecynyl)pyrrolidine-2,5-dione (249.5 mg, 1.0 mmol) was then added. After being stirred for 7.5 hours at room temperature, the reaction mixture was stirred at 80 °C with a pre-heated oil bath for 24 hours. After being cooled to room temperature, 6 mL of a saturated aqueous solution of Na_2SO_4 was added. After filtration, extraction with EtOAc (3×10 mL), and evaporation, the residue was purified by chromatography on silica gel to afford *d*$_4$-**1a** (100.6 mg, 45%, 97% deuteration) (eluent: petroleum ether/ acetone = 30/1) as a liquid: ^{1}H NMR (400 MHz, $CDCl_3$) δ 3.39-3.33 (m, 2 H, CH_2), 2.22-2.14 (m, 2 H, CH_2), 1.78 (s, 4 H, 2×CH_2), 1.49 (quint, *J* = 7.1 Hz, 2 H, CH_2), 1.43-1.18 (m, 10 H, 5×CH_2), 0.88 (t, *J* = 6.6 Hz, 3 H, CH_3); ^{13}C NMR (100 MHz, $CDCl_3$) δ 84.3, 75.5, 51.6 (quint, $J_{C\text{-}D}$ = 20.3 Hz), 43.3, 31.7, 29.1, 29.0, 28.8, 28.76, 28.73, 23.4, 22.5, 18.6, 13.9; MS (EI) *m/z* (%) 225 ($[M]^+$, 34.66), 224 (100); IR (neat) *ν* = 2955, 2924, 2873, 2854 , 2771, 2199, 2056, 1464, 1433, 1359, 1326, 1260, 1214, 1125, 1068, 1017 cm^{-1}; HRMS calcd for $C_{15}NH_{23}D_4$

(M^+): 225.2395. Found: 225.2393.

The following signals are discernible for **1a**: ^{1}H NMR (400 MHz, $CDCl_3$) δ 2.57 (s, 4 H, 2×CH_2). ^{13}C NMR (100 MHz, $CDCl_3$) δ 52.2.

(2) (*E*)-2-(Phenylethynyl)-1-(3-deuterio-2-undecenyl)-2,5,5-trideuteriopyrrolidine (*d*$_4$-**3aa**) (cyf-1-089)

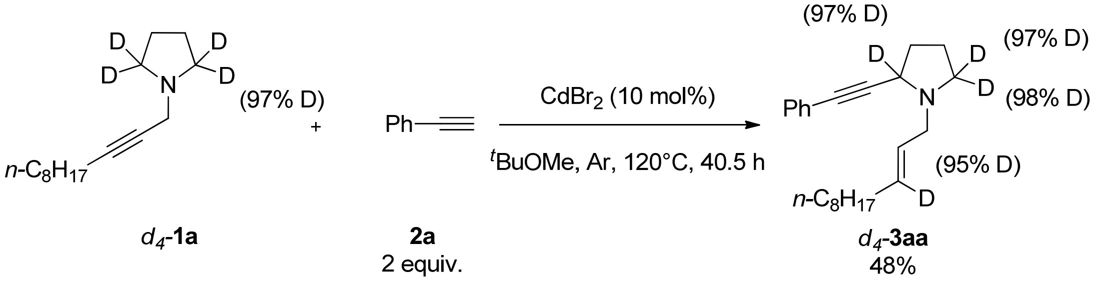

To a dry Schlenk tube with a polytetrafluoroethylene plug was added $CdBr_2$ (8.3 mg, 0.03 mmol). Then the catalyst was dried under vacuum with heating. *d*$_4$-**1a** (67.8 mg, 0.3 mmol), MTBE (1 mL), phenylacetylene **2a** (63.4 mg, 0.6 mmol), and MTBE (1 mL) were then added sequentially under Ar atmosphere. The resulting mixture was stirred at 120 °C for 40.5 h. After the completion of the reaction as monitored by TLC, the crude reaction mixture was filtrated through a short column of silica gel eluted with Et_2O (50 mL). After evaporation, the residue was purified by column chromatography on silica gel to afford *d*$_4$-**3aa** (46.9 mg, 48%, greater than 95% of incorporation) (eluent: petroleum ether/ acetone = 100/1) as a liquid: ^{1}H NMR (400 MHz, $CDCl_3$) δ 7.47-7.39 (m, 2 H, ArH), 7.36-7.25 (m, 3 H, ArH), 5.57 (t, *J* = 6.6 Hz, 1 H, =CH), 3.47 (dd, *J* = 12.6, 6.2 Hz, 1 H, one proton of NCH_2), 3.03 (dd, *J* = 12.8, 7.6 Hz, 1 H, one proton of NCH_2), 2.23-2.12 (m, 1 H, one proton of CH_2), 2.07-1.86 (m, 4 H, CH_2 and 2×one proton of CH_2), 1.86-1.75 (m, 1 H, one proton of CH_2), 1.41-1.20 (m, 12 H, 6×CH_2), 0.88 (t, *J* = 6.6 Hz, 3 H, CH_3); ^{13}C NMR (100 MHz, $CDCl_3$) δ 133.8 (t, $J_{C\text{-}D}$ = 22.6 Hz), 131.7, 128.2, 127.8, 126.6, 123.4, 88.7, 84.6, 55.2, 54.1 (t, $J_{C\text{-}D}$ = 21.8 Hz), 50.8 (quint, $J_{C\text{-}D}$ = 20.9 Hz), 32.2, 31.9, 31.6, 29.4, 29.3, 29.2, 29.1, 22.7, 21.8, 14.1; MS (EI) *m/z* (%) 327 ([M]$^+$, 5.10), 213 (100); IR (neat) *v* = 3056, 2954, 2853, 2195, 2060, 1598, 1573, 1489, 1463, 1443, 1363, 1311, 1285, 1259, 1203, 1117, 1068, 1027 cm^{-1}; HRMS calcd for $C_{23}H_{29}D_4N$ (M^+): 327.2864. Found:

327.2858.

The following signals are discernible for (**3aa**):^{1}H NMR (400 MHz, $CDCl_3$) δ 5.65 (dt, *J* = 15.6, 6.4 Hz, 1 H, =CH), 3.58 (dd, *J* = 7.4, 5.8 Hz, 1 H, NCH), 2.85 (dd, *J* = 9.2, 6.0 Hz, 1 H, one proton of NCH_2), 2.49 (dd, *J* = 8.2, 6.6 Hz, 1 H, one proton of NCH_2). ^{13}C NMR (100 MHz, $CDCl_3$) δ 134.1, 54.5.

(3) (*E*)-2-(phenylethynyl)-1-(2-deuterio-2-undecenyl)-3-deuteriopyrrolidine (d_2-**3aa**) (cyf-1-075)

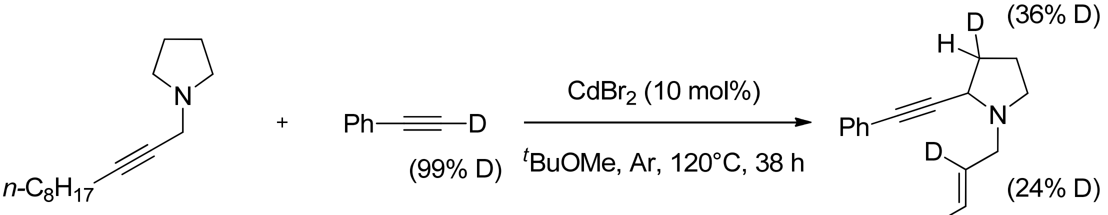

1a d-**2a** 2 equiv. d_2-**3aa** 61%

To a dry Schlenk tube with a polytetrafluoroethylene plug was added $CdBr_2$ (27.7 mg, 0.1 mmol). Then the catalyst was dried under vacuum with heating. **1a** (222.0 mg, 1.0 mmol), MTBE (3 mL), deuterated phenylacetylene ***d*-2a** (207.0 mg, 2.0 mmol), and MTBE (3 mL) were then added sequentially under Ar atmosphere. The resulting mixture was stirred at 120 °C for 38 h. After the completion of the reaction as monitored by TLC, the crude reaction mixture was filtrated through a short column of silica gel eluted with Et_2O (50 mL). After evaporation, the residue was purified by column chromatography on silica gel to afford d_2-**3aa** (198.0 mg, 61%, D-incorporation: =CD: (1-0.76) × 100% = 24%, CHD: (1-0.64) × 100% = 36%) (eluent: petroleum ether/ acetone = 200/1 (200 mL) to 100/1 (600 mL)) as a liquid: ^{1}H NMR (400 MHz, $CDCl_3$) δ 7.50-7.39 (m, 2 H, ArH), 7.34-7.24 (m, 3 H, ArH), 5.67 (dt, *J* = 15.2, 6.2 Hz, 1 H, =CH), 3.61-3.54 (m, 1 H, NCH), 3.52-3.45 (m, 1 H, one proton of NCH_2), 3.02 (dd, *J* = 12.6, 7.0 Hz, 1 H, one proton of NCH_2), 2.88-2.80 (m, 1 H, one proton of NCH_2), 2.51 (dd, *J* = 15.4, 8.6 Hz, 1 H, one proton of NCH_2), 2.09-1.87 (m, 4 H, CH_2 and 2×one proton of CH_2), 1.86-1.74 (m, 1 H, one proton of CH_2), 1.46-1.14 (m, 12 H, 6×CH_2), 0.88 (t, *J* = 6.6 Hz, 3 H, CH_3); ^{13}C NMR (100

MHz, $CDCl_3$) δ 134.0, 131.6, 128.1, 127.8, 126.4 (t, $J_{C\text{-}D}$ = 23.5 Hz), 123.4, 88.7, 84.5, 55.2, 54.45, 51.6, 32.27, 31.8, 31.2 (t, $J_{C\text{-}D}$ = 19.9 Hz), 29.4, 29.25, 29.19, 29.1, 22.6, 21.9, 14.1; MS (EI) *m/z* (%) 325 ($[M(C_{23}H_{31}D_2N)]^+$, 13.27), 324 ($[M(C_{23}H_{32}DN)]^+$, 32.36), 323 ($[M(C_{23}H_{33}N)]^+$, 44.31), 211 (100); IR (neat) *v* = 3058, 3021, 2955, 2922, 2853, 2807, 1598, 1573, 1489, 1459, 1443, 1375, 1321, 1267, 1158, 1116, 1070, 1028 cm^{-1}; HRMS calcd for $C_{23}H_{31}D_2N$ (M^+): 325.2739. Found: 325.2747.

The following signals are discernible for (**3aa**): ^1H NMR (400 MHz, $CDCl_3$) δ 5.58 (dt, *J* = 15.2, 6.6 Hz, 1 H, =CH), 1.86-1.74 (m, 1 H, one proton of CH_2). ^{13}C NMR (100 MHz, $CDCl_3$) δ 134.1, 126.7, 55.3, 54.51, 32.33, 31.7, 22.0.

(4) (*E*)-2-(Phenylethynyl)-1-(2-undecenyl)pyrrolidine (**3aa**) (cyf-1-101)

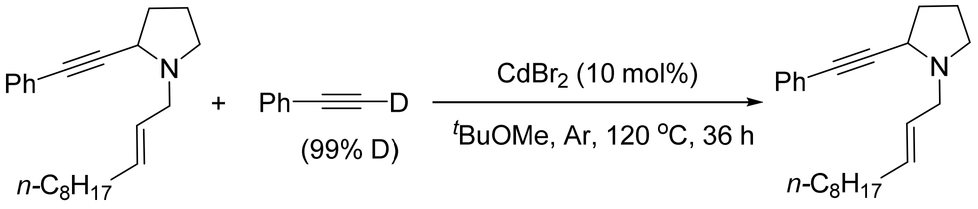

3aa *d*-**2a** 2 equiv. **3aa** 78% recovery

To a dry Schlenk tube with a polytetrafluoroethylene plug was added $CdBr_2$ (11.2 mg, 0.04 mmol). Then the catalyst was dried under vacuum with heating. After cooling to room temperature, deuterated phenylacetylene ***d*-2a** (83.5 mg, 0.8 mmol), MTBE (1.2 mL), **3aa** (128.9 mg, 0.4 mmol), and MTBE (1.2 mL) were added sequentially under Ar atmosphere. The resulting mixture was stirred at 120 °C for 36 h. The crude reaction mixture was diluted with 5 mL of DCM and filtrated through a short pad of silica gel eluted with DCM (30 mL). After evaporation, the residue was purified by chromatography on silica gel to afford **3aa** (101.3 mg, 78%) (eluent: petroleum ether/ acetone = 100/1 (200 mL) to 50/1 (200 mL)) as a liquid: ^1H NMR (400 MHz, $CDCl_3$) δ 7.47-7.39 (m, 2 H, ArH), 7.33-7.25 (m, 3 H, ArH), 5.67 (dt, *J* = 15.2, 6.2 Hz, 1 H, =CH), 5.58 (dt, *J* = 15.2, 6.4 Hz, 1 H, =CH), 3.56 (dd, *J* = 6.8, 5.6 Hz, 1 H, NCH), 3.49 (dd, *J* = 12.6, 5.4 Hz, 1 H, one proton of NCH_2), 3.00 (dd, *J* = 12.8, 7.2 Hz, 1 H, one proton of NCH_2), 2.85 (td, *J* = 8.7, 5.2 Hz, 1 H, one proton of NCH_2), 2.55-2.45 (m, 1 H, one proton of NCH_2), 2.24-2.12 (m, 1 H, one proton of

CH_2), 2.08-1.87 (m, 4 H, CH_2 and 2×one proton of CH_2), 1.87-1.75 (m, 1 H, one proton of CH_2), 1.42-1.18 (m, 12 H, 6×CH_2), 0.88 (t, *J* = 6.8 Hz, 3 H, CH_3); ^{13}C NMR (100 MHz, $CDCl_3$) δ 134.1, 131.6, 128.1, 127.8, 126.7, 123.4, 88.7, 84.5, 55.3, 54.5, 51.6, 32.3, 31.8, 31.7, 29.4, 29.24, 29.18, 29.1, 22.6, 22.0, 14.1.

8. Gram-scale synthesis.

(1) 1-Allyl-2-((4-chlorophenyl)ethynyl)pyrrolidine (**3ee**) (cyf-1-051)

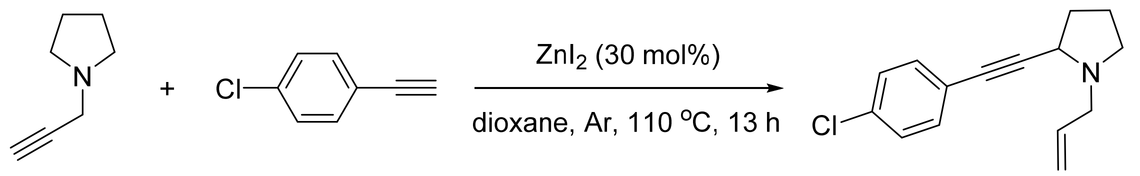

1e **2e** 2 equiv. **3ee** 46%

To a dry Schlenk tube with a polytetrafluoroethylene plug was added ZnI_2 (967.0 mg, 3.0 mmol). Then the catalyst was heated under vacuum until sublimation of ZnI_2. After cooling to room temperature, 4-chlorophenylacetylene **2e** (2.7890 g, 20.0 mmol), **1e** (1.0920 g, 10.0 mmol) and dioxane (50 mL) were added sequentially under Ar atmosphere. Then the Schlenk tube was stirred at 110 °C for 13 h. After the completion of the reaction as monitored by TLC, the crude reaction mixture was filtrated through a short columm of silica gel eluted with DCM (60 mL) and EtOAc (100 mL). After evaporation, the residue was purified by columm chromatography on silica gel to afford **3ee** (1.1364 g, 46%) (eluent: petroleum ether/ acetone = 200/1 (500 mL) to 50/1 (1000 mL)) as a liquid: ^1H NMR (400 MHz, $CDCl_3$) δ 7.38-7.32 (m, 2 H, ArH), 7.29-7.23 (m, 2 H, ArH), 6.03-5.89 (m, 1 H, =CH), 5.25 (d, *J* = 17.2 Hz, 1 H, one proton of =CH_2), 5.13 (d, *J* = 10.0 Hz, 1 H, one proton of =CH_2), 3.59-3.51 (m, 2 H, NCH and one proton of NCH_2), 3.04 (dd, *J* = 13.2, 7.6 Hz, 1 H, one proton of NCH_2), 2.85 (td, *J* = 8.7, 4.9 Hz, 1 H, one proton of NCH_2), 2.54-2.45 (m, 1 H, one proton of NCH_2), 2.24-2.13 (m, 1 H, one proton of CH_2), 2.07-1.87 (m, 2 H, 2×one proton of CH_2), 1.87-1.75 (m, 1 H, one proton of CH_2); ^{13}C NMR (100 MHz, $CDCl_3$) δ 135.4, 133.8, 132.8, 128.4, 121.7, 117.3, 89.6, 83.5, 56.2, 54.6, 51.6, 31.6, 22.0.

(2) (*E*)-2-((trimethylsilyl)ethynyl)-1-(2-undecenyl)pyrrolidine (**3am**) (cyf-1-069)

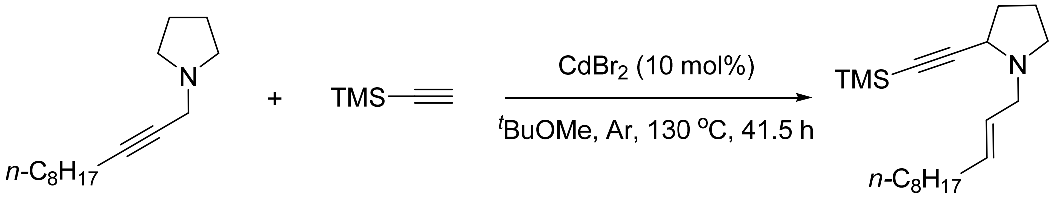

1a **2m** 2 equiv. **3am** 78%

Following **Typical Procedure II**, the reaction of $CdBr_2$ (138.6 mg, 0.5 mmol), **1a** (1.1067 g, 5.0 mmol), trimethylsilylacetylene **2m** (1.0025 g, 10.0 mmol), and MTBE (30 mL) afforded **3am** (1.2437 g, 78%) (eluent: petroleum ether/ acetone = 200/1 to 100/1) as a liquid: ^{1}H NMR (400 MHz, $CDCl_3$) δ 5.63 (dt, *J* = 15.2, 6.4 Hz, 1 H, =CH), 5.54 (dt, *J* = 16.0, 6.6 Hz, 1 H, =CH), 3.42 (dd, *J* = 12.4, 6.0 Hz, 1 H, one proton of NCH_2), 3.32 (dd, *J* = 7.4, 5.4 Hz, 1 H, NCH) , 2.91 (dd, *J* = 12.6, 7.4 Hz, 1 H, one proton of NCH_2), 2.82-2.72 (m, 1 H, one proton of NCH_2), 2.48-2.38 (m, 1 H, one proton of NCH_2), 2.15-1.97 (m, 3 H, CH_2 and one proton of CH_2), 1.96-1.67 (m, 3 H, CH_2 and one proton of CH_2), 1.43-1.15 (m, 12 H, 6×CH_2), 0.88 (t, *J* = 6.8 Hz, 3 H, CH_3), 0.16 (s, 9 H, 3×$SiCH_3$); ^{13}C NMR (100 MHz, $CDCl_3$) δ 134.0, 126.7, 105.4, 88.6, 55.2, 54.5, 51.5, 32.3, 31.9, 31.6, 29.4, 29.3, 29.2, 29.1, 22.6, 21.9, 14.1, 0.1.

9. Synthetic applications

(1) 2-(4-phenylphenylethynyl)-1-allylpyrrolidine (**5**) (cyf-1-081)

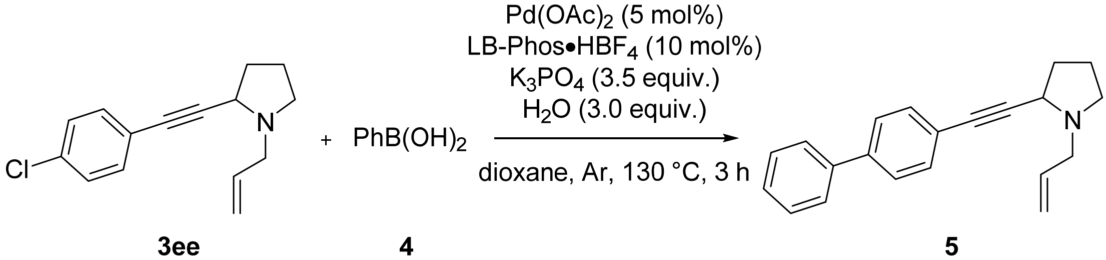

3ee **4** 2 equiv. **5** 81%

To a dry Schlenk tube with a polytetrafluoroethylene plug was added K_3PO_4 (383.3 mg, 1.75 mmol). Then it was dried under vacuum with a heating gun. $Pd(OAc)_2$ (5.8 mg, 0.025 mmol), LB-Phos·HBF_4 (23.2 mg, 0.05 mmol), phenylboronic acid **4** (124.4 mg, 1 mmol), and dioxane (0.5 mL) were added

sequentially under Ar atmosphere. After being stirred for 5 min at room temperature, 1-allyl-2-((4-chlorophenyl)ethynyl)pyrrolidine **3ee** (123.0 mg, 0.5 mmol), dioxane (0.5 mL) and 27.0 μL of H_2O (27.0 mg, 1.5 mmol) were added. The resulting mixture was heated at 130 °C with a preheated oil bath. After 3 hours, it was allowed to cool to room temperature. The reaction mixture was diluted with 5 mL of dichloromethane and filtered through a short column of silica gel (eluent: 50 mL of Et_2O). After evaporation, the residue was purified by column chromatography on silica gel to afford **5** (116.8 mg, 81%) (eluent: petroleum ether/ acetone = 200:1 (400 mL) to 100:1 (300 mL)) as a liquid: ^1H NMR (400 MHz, $CDCl_3$) δ 7.68-7.29 (m, 9 H, ArH), 6.07-5.90 (m, 1 H, =CH), 5.27 (d, *J* = 16.8 Hz, 1 H, one proton of $=CH_2$), 5.14 (d, *J* = 10.0 Hz, 1 H, one proton of $=CH_2$), 3.67-3.52 (m, 2 H, NCH and one proton of NCH_2), 3.08 (dd, *J* = 12.8, 7.6 Hz, 1 H, one proton of NCH_2), 2.94-2.82 (m, 1 H, one proton of NCH_2), 2.52 (q, *J* = 8.0 Hz, 1 H, one proton of NCH_2), 2.29-2.14 (m, 1 H, one proton of CH_2), 2.11-1.76 (m, 3 H, 3×one proton of CH_2); ^{13}C NMR (100 MHz, $CDCl_3$) δ 140.6, 140.3, 135.6, 132.1, 128.8, 127.5, 126.94, 126.85, 122.2, 117.3, 89.2, 84.5, 56.3, 54.8, 51.7, 31.7, 22.0; MS (EI) *m/z* (%) 287 (M^+, 61.40) 286 (100); IR (neat) *v* = 3076, 3030, 2975, 2955, 2910, 2875, 1643, 1600, 1518, 1485, 1459, 1444, 1418, 1403, 1354, 1318, 1284, 1259, 1180, 1159, 1140, 1108, 1077, 1007 cm^{-1}; HRMS calcd for $C_{21}H_{21}N$ (M^+): 287.1674. Found: 287.1676.

(2) (*E*)-2-ethynyl-1-(2-undecenyl)pyrrolidine (**6**) (cyf-1-087)

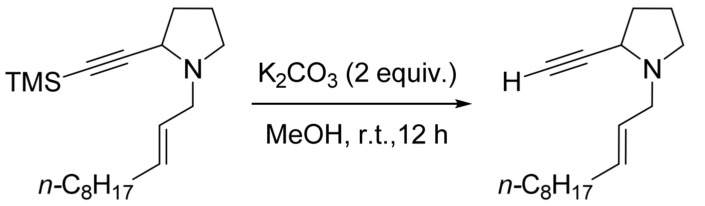

3am **6** 100%

To a round-bottom flask were added (*E*)-2-((trimethylsilyl)ethynyl)-1-(2-undecenyl)-pyrrolidine **3am** (63.9 mg, 0.2 mmol), MeOH (3 mL), and K_2CO_3 (55.7 mg, 0.4 mmol) sequentially. The resulting mixture was allowed to stir at room temperature for 12 h. MeOH was then evaporated under

vacuum and the residue was diluted with 5 mL of dichloromethane and filtrated through a short column of silica gel (eluent: 25 mL of Et_2O). After evaporation, the residue was purified by chromatography on silica gel to afford **6** (50.2 mg, 100%) (eluent: petroleum ether/ acetone = 50:1) as a liquid: ^{1}H NMR (400 MHz, $CDCl_3$) δ 5.64 (dt, J = 15.6, 6.8 Hz, 1 H, =CH), 5.53 (dt, J = 15.2, 6.8 Hz, 1 H, =CH), 3.44-3.36 (m, 2 H, NCH and one proton of NCH_2), 2.96 (dd, J = 13.0, 7.4 Hz, 1 H, one proton of NCH_2), 2.79-2.71 (m, 1 H, one proton of NCH_2), 2.54-2.45 (m, 1 H, one proton of NCH_2), 2.29-2.25 (m, 1 H, ≡CH), 2.18-2.06 (m, 1 H, one proton of CH_2), 2.06-1.66 (m, 5 H, 2×CH_2 and one proton of CH_2), 1.41-1.16 (m, 12 H, 6×CH_2), 0.88 (t, J = 6.6 Hz, 3 H, CH_3); ^{13}C NMR (100 MHz, $CDCl_3$) δ 134.1, 126.6, 83.1, 72.2, 54.9, 53.6, 51.3, 32.3, 31.8, 31.6, 29.4, 29.3, 29.2, 29.1, 22.6, 21.9, 14.1; MS (EI) m/z (%) 247 (M^+, 24.18) 134 (100); IR (neat) v = 3309, 2955, 2922, 2872, 2853, 2812, 1460, 1376, 1351, 1319, 1115, 1088 cm^{-1}; HRMS calcd for $C_{17}H_{29}N$ (M^+): 247.2300. Found: 247.2302.

(3) (*E*)-2-(4-trimethylsilylbuta-1,3-diynyl)-1-(2-undecenyl)pyrrolidine (**7**) (cyf-1-120B)

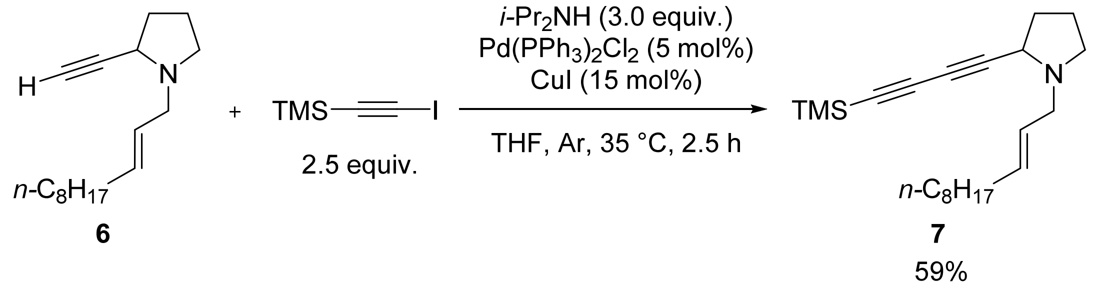

To a flame-dried Schlenk tube were added $Pd(PPh_3)_2Cl_2$ (6.9 mg, 0.01 mmol), CuI (5.9 mg, 0.03 mmol), (iodoethynyl)trimethylsilane (122.0 mg, 0.5 mmol), THF (1 mL), diisopropylamine (84.0 μL, d = 0.718 g/cm^3, 60.3 mg, 0.6 mmol), (*E*)-2-ethynyl-1-(2-undecenyl)pyrrolidine **6** (49.4 mg, 0.2 mmol), and THF (0.5 mL) under Ar atmosphere. The resulting mixture was heated at 35 °C with a preheated oil bath. After the completion of the reaction as monitored by TLC, the crude reaction mixture was diluted with 5 mL of dichloromethane and filtrated through a short

column of silica gel (eluent: 25 mL of Et_2O). After evaporation, the residue was purified by chromatography on silica gel to afford **7** (40.2 mg, 59%) (eluent: petroleum ether/ dichloromethane/ ethyl ether = 20:1:1) as a liquid: ^{1}H NMR (400 MHz, $CDCl_3$) δ 5.66 (dt, *J* = 15.2, 6.8 Hz, 1 H, =CH), 5.50 (dt, *J* = 15.6, 7.0 Hz, 1 H, =CH), 3.57 (dd, *J* = 7.4, 3.8 Hz, 1 H, NCH), 3.32 (dd, *J* = 12.6, 6.2 Hz, 1 H, one proton of NCH_2), 3.02 (dd, *J* = 12.6, 7.4 Hz, 1 H, one proton of NCH_2), 2.72-2.56 (m, 2 H, NCH_2), 2.17-1.72 (m, 6 H, 3×CH_2), 1.42-1.20 (m, 12 H, 6×CH_2), 0.88 (t, *J* = 6.8 Hz, 3 H, CH_3), 0.20 (s, 9 H, 3×$SiCH_3$); ^{13}C NMR (100 MHz, $CDCl_3$) δ 134.4, 126.4, 87.8, 85.3, 78.1, 69.7, 54.8, 54.0, 51.2, 32.3, 31.9, 31.4, 29.4, 29.3, 29.15, 29.1, 22.7, 22.0, 14.1, -0.4; MS (ESI) *m/z* 344 ($[M+H]^+$); IR (neat) *ν* = 2957, 2923, 2853, 2811, 2226, 2207, 2175, 2101, 1461, 1375, 1315, 1250, 1151, 1108, 1074, 1022 cm^{-1}; HRMS calcd for $C_{22}H_{38}NSi$ ($[M+H]^+$): 344.2768. Found: 344.2752.

(5) (5a*R**,6*R**,8b*S**)-6-octyl-1,2,3,5a,6,8b-hexahydrocyclopenta[*a*]pyrrolizin-7(5*H*)-one (**8**) (cyf-1-165)

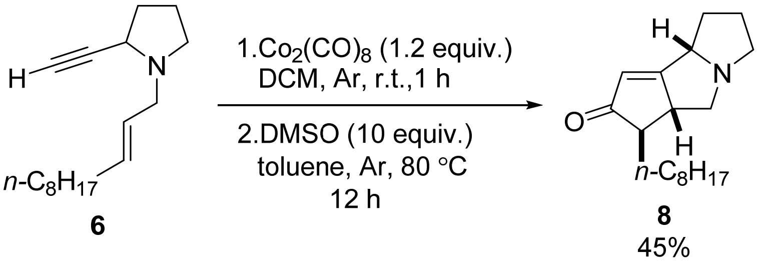

6 **8** 45%

To a flame-dried Schlenk tube were added $Co_2(CO)_8$ (82.7 mg, 0.24 mmol), dicholoromethane (3 mL), (*E*)-2-ethynyl-1-(2-undecenyl)pyrrolidine **6** (49.5 mg, 0.2 mmol), and dicholoromethane (3 mL) sequentially under Ar atmosphere. The resulting mixture was stirred at room temperature for 1 hour. After evaporation of the solvent, the Schlenk tube was added dry toluene (4 mL) and DMSO (140 μL, d = 1.10 g/cm^3, 2.0 mmol). The reaction mixture was then heated at 80 °C with a preheated oil bath. After 12 hours the crude reaction mixture was quenched with 5 mL of H_2O and 1 mL of NH_3 aqueous solution. After separation of organic phase, extraction with DCM (3×10 mL), the combined organic phase was dried over anhydrous Na_2SO_4, After filtration and evaporation, the residue was purified by preparative thin layer chromatography on silica gel (20 × 20 cm) (dichloromethane/

methanol = 30:1 to 15:1) to afford (5a*R**,6*R**,8b*S**)-6-octyl-1,2,3,5a,6,8b-hexahydrocyclopenta[*a*]pyrrolizin-7(5*H*)-one **8** (24.9 mg, 45%) as a liquid: ^{1}H NMR (400 MHz, $CDCl_3$) δ 6.00 (s, 1 H, =CH), 4.17 (t, *J* = 8.0 Hz, 1 H, NCH), 3.33 (t, *J* = 6.6 Hz, 1 H, one proton of NCH_2), 3.20 (dd, *J* = 11.6, 8.0 Hz, 1 H, one proton of NCH_2), 2.98-2.88 (m, 1 H, CH), 2.78-2.63 (m, 2 H, 2×one proton of CH_2), 2.32-2.21 (m, 1 H, one proton of CH_2), 2.10-1.83 (m, 4 H, CH_2, CH and one proton of CH_2), 1.65-1.52 (m, 1 H, one proton of CH_2), 1.46-1.19 (m, 13 H, 6×CH_2 and one proton of CH_2), 0.88 (t, *J* = 6.6 Hz, 3 H, CH_3); ^{13}C NMR (100 MHz, $CDCl_3$) δ 211.6, 187.8, 123.3, 57.6, 62.1, 56.5, 53.3, 47.9, 32.2, 31.8, 29.5, 29.4, 29.2, 28.7, 28.0, 27.8, 22.6, 14.1; MS (ESI) *m/z* 276 ([M+H]$^+$); IR (neat) v = 2923, 2855, 1700, 1457, 1373, 1258, 1084, 1017 cm^{-1}; HRMS calcd for $C_{18}H_{30}NO$ ([M+H]$^+$): 276.2322. Found: 276.2318.

References:

1. Bieber, L. W.; Da Silva, M. F. *Tetrahedron Lett.* **2004**, *45*, 8281.
2. Biel, J. H.; DiPierro, F. *J. Am. Chem. Soc.* **1958**, *80*, 4609.
3. Bew, S. P.; Hiatt-Gipson, G. D.; Lovell, J. A.; Poullain, C. *Org. Lett.* **2012**, *14*, 456.
4. Kuang J.; Ma, S. *J. Org. Chem.* **2009**, *74*, 1763.
