# Supplementary material for: A metal-catalyzed new approach for α-alkynylation of cyclic amines
Source: Chem Sci. 2018 Nov 26;10(6):1796–801. doi: 10.1039/c8sc04115f (PMC6369436; doi:10.1039/c8sc04115f)
Supplement: Supplementary file 2 [file SC-010-C8SC04115F-s002.pdf]

Electronic Supplementary Material (ESI) for Chemical Science.
This journal is © The Royal Society of Chemistry 2018

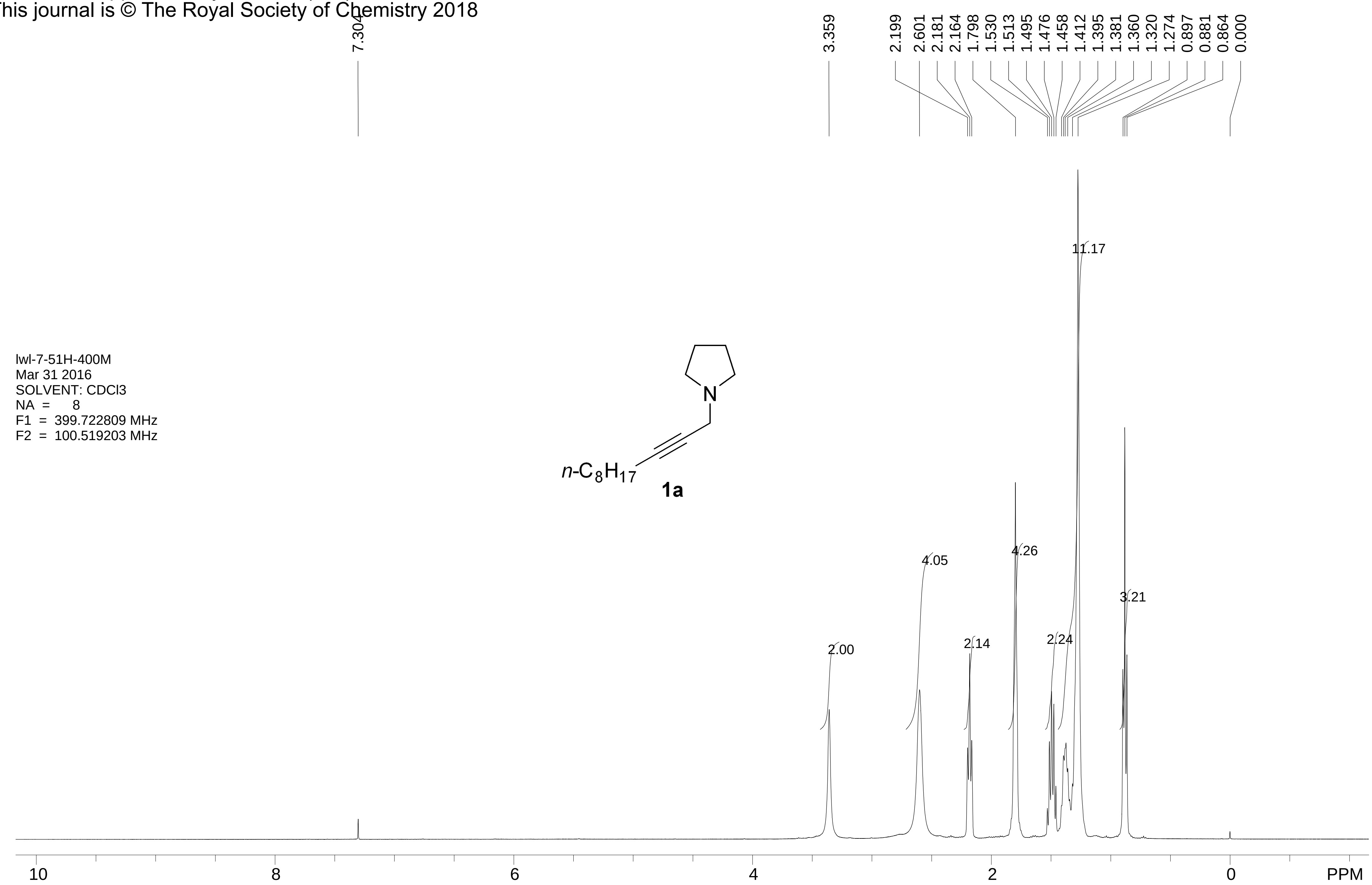

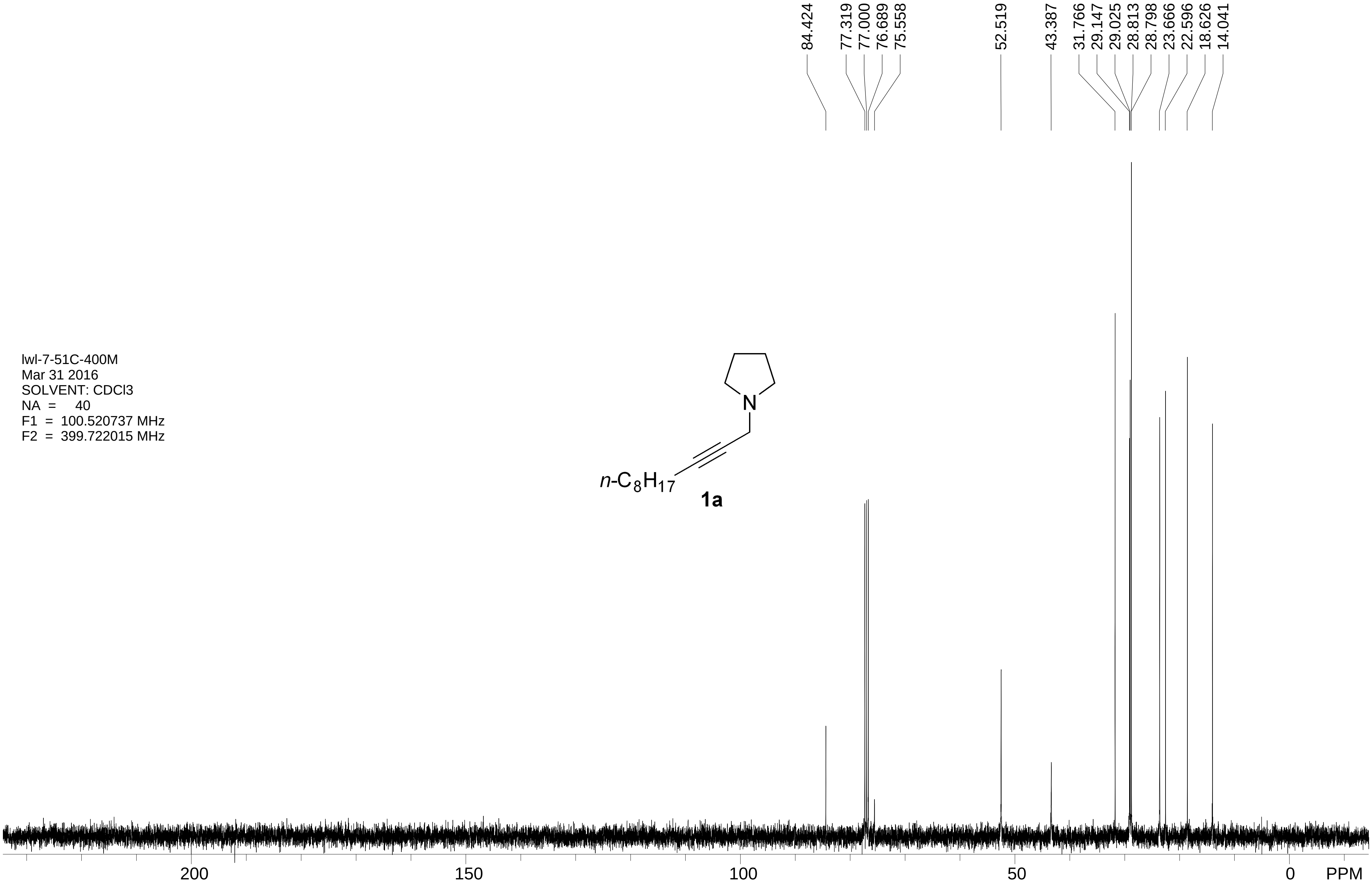
84.424
77.319
77.000
76.689
75.558
52.519
43.387
31.766
29.147
29.025
28.813
28.798
23.666
22.596
18.626
14.041
lwl-7-51C-400M
Mar 31 2016
SOLVENT: CDCl3
NA = 40
F1 = 100.520737 MHz
F2 = 399.722015 MHz
N
n-C8H17
1a
200
150
100
50
0
PPM

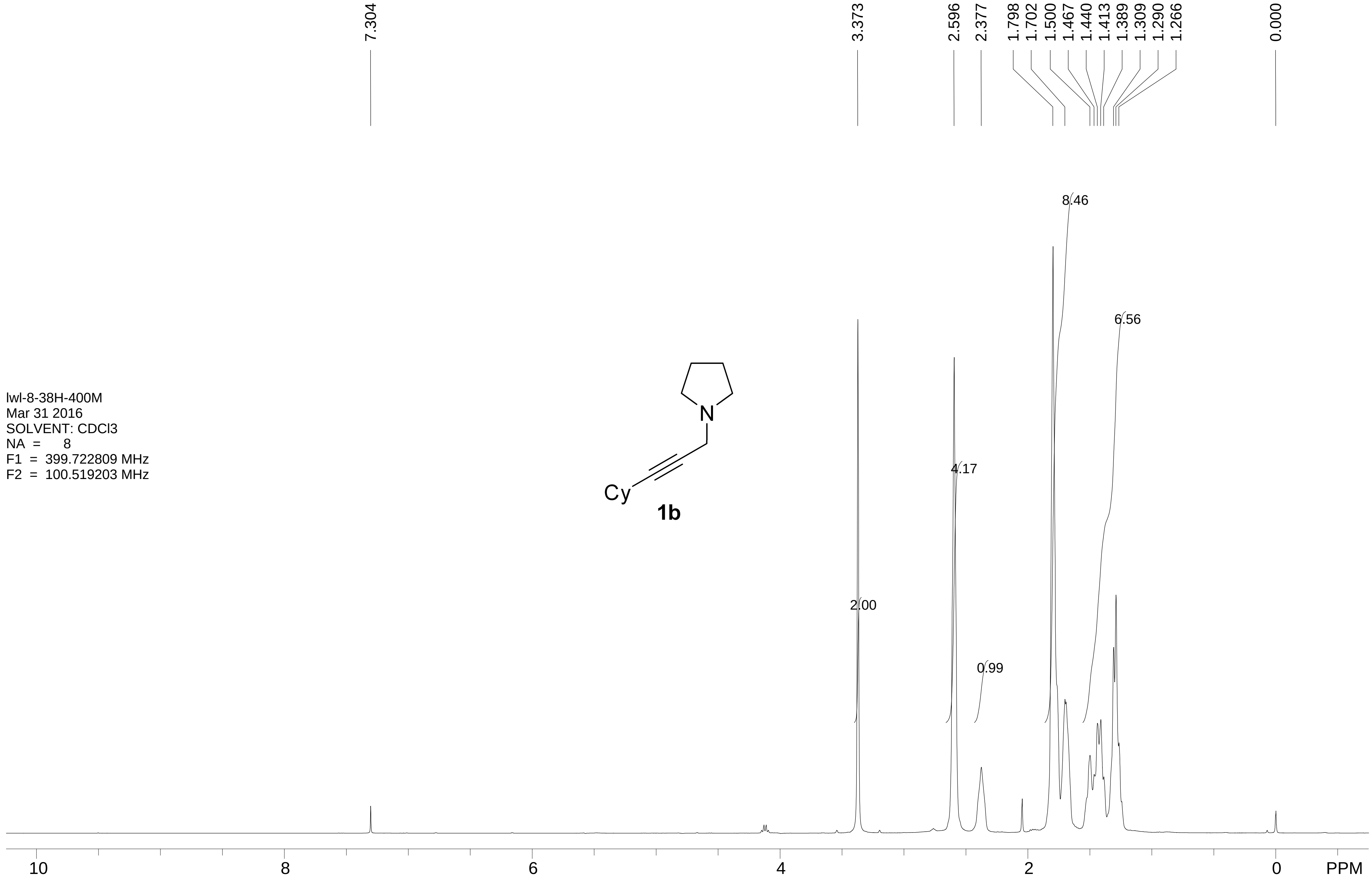
7.304
3.373
2.596
2.377
1.798
1.702
1.500
1.467
1.440
1.413
1.389
1.309
1.290
1.266
0.000
8.46
6.56
4.17
2.00
0.99
lwl-8-38H-400M
Mar 31 2016
SOLVENT: CDCl3
NA = 8
F1 = 399.722809 MHz
F2 = 100.519203 MHz
N
Cy
1b
10
8
6
4
2
0
PPM

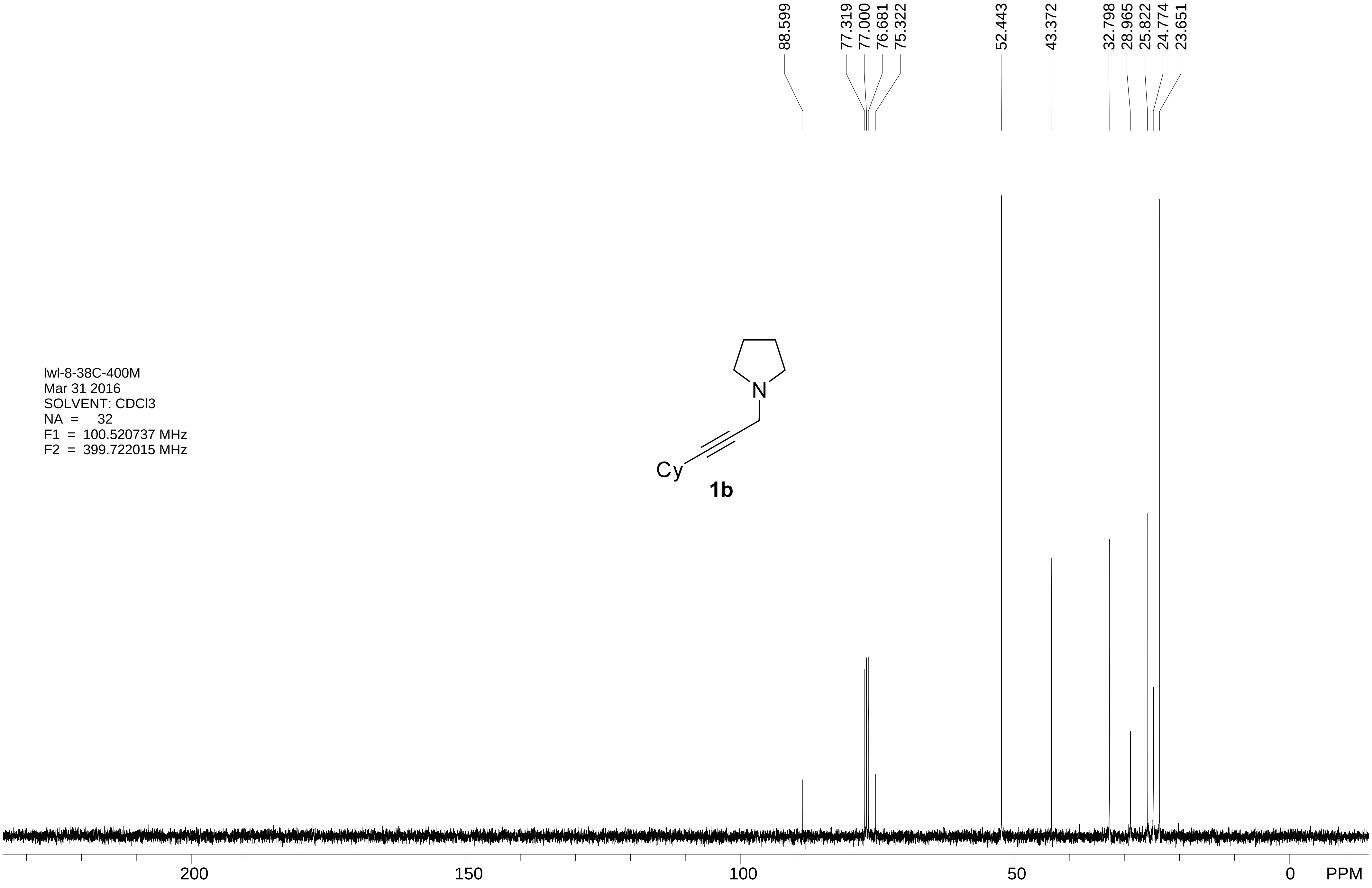
88.599
77.319
77.000
76.681
75.322
52.443
43.372
32.798
28.965
25.822
24.774
23.651
lwl-8-38C-400M
Mar 31 2016
SOLVENT: CDCl3
NA = 32
F1 = 100.520737 MHz
F2 = 399.722015 MHz
N
Cy
1b
200
150
100
50
0
PPM

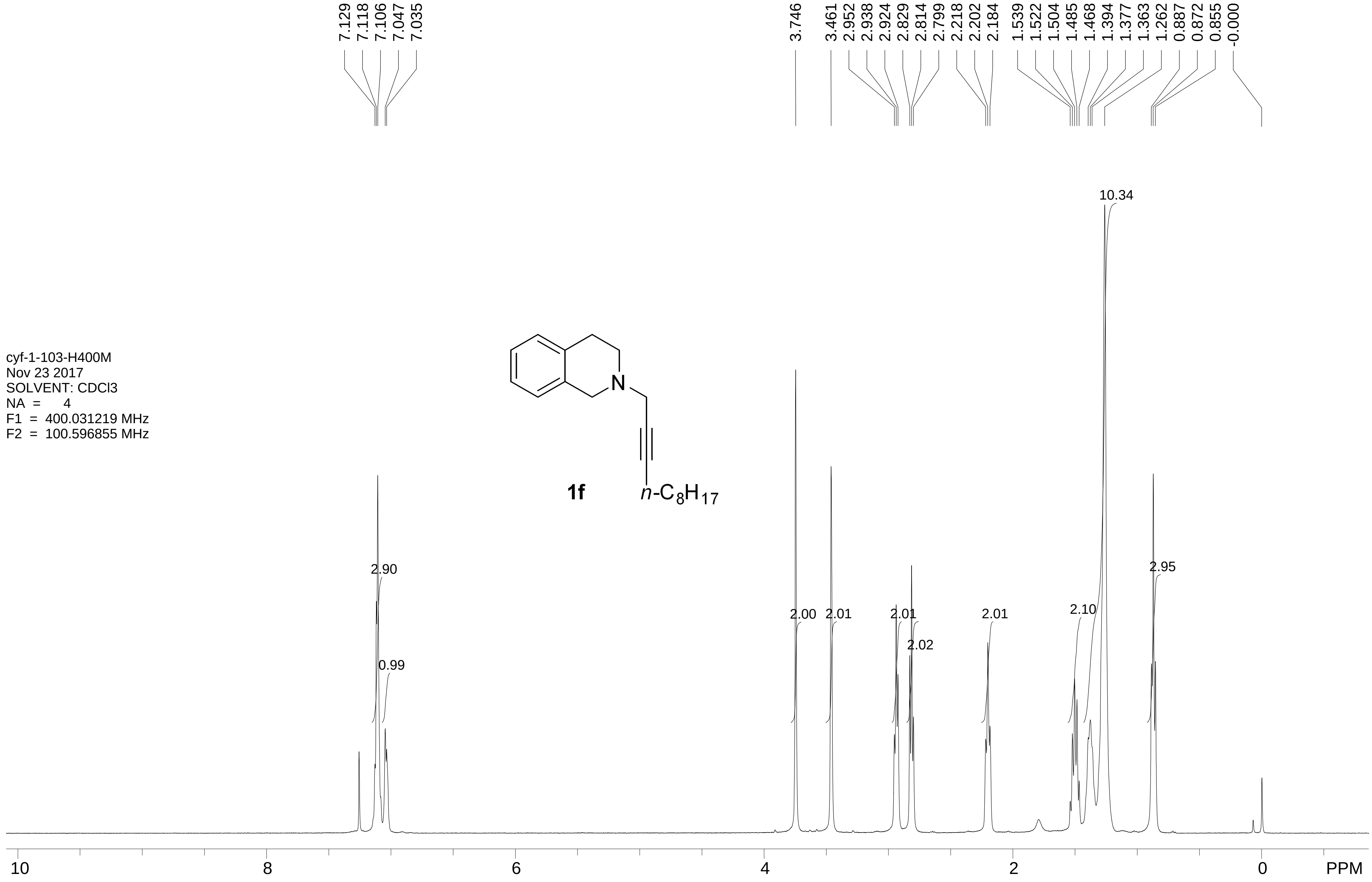
cyf-1-103-H400M
Nov 23 2017
SOLVENT: CDCl3
NA = 4
F1 = 400.031219 MHz
F2 = 100.596855 MHz
1f
n-C8H17
N
7.129 7.118 7.106 7.047 7.035
3.746
3.461 2.952 2.938 2.924 2.829 2.814 2.799 2.218 2.202 2.184
1.539 1.522 1.504 1.485 1.468 1.394 1.377 1.363 1.262 0.887 0.872 0.855 -0.000
2.90
0.99
2.00
2.01
2.01
2.02
2.01
2.10
10.34
2.95
10
8
6
4
2
0
PPM

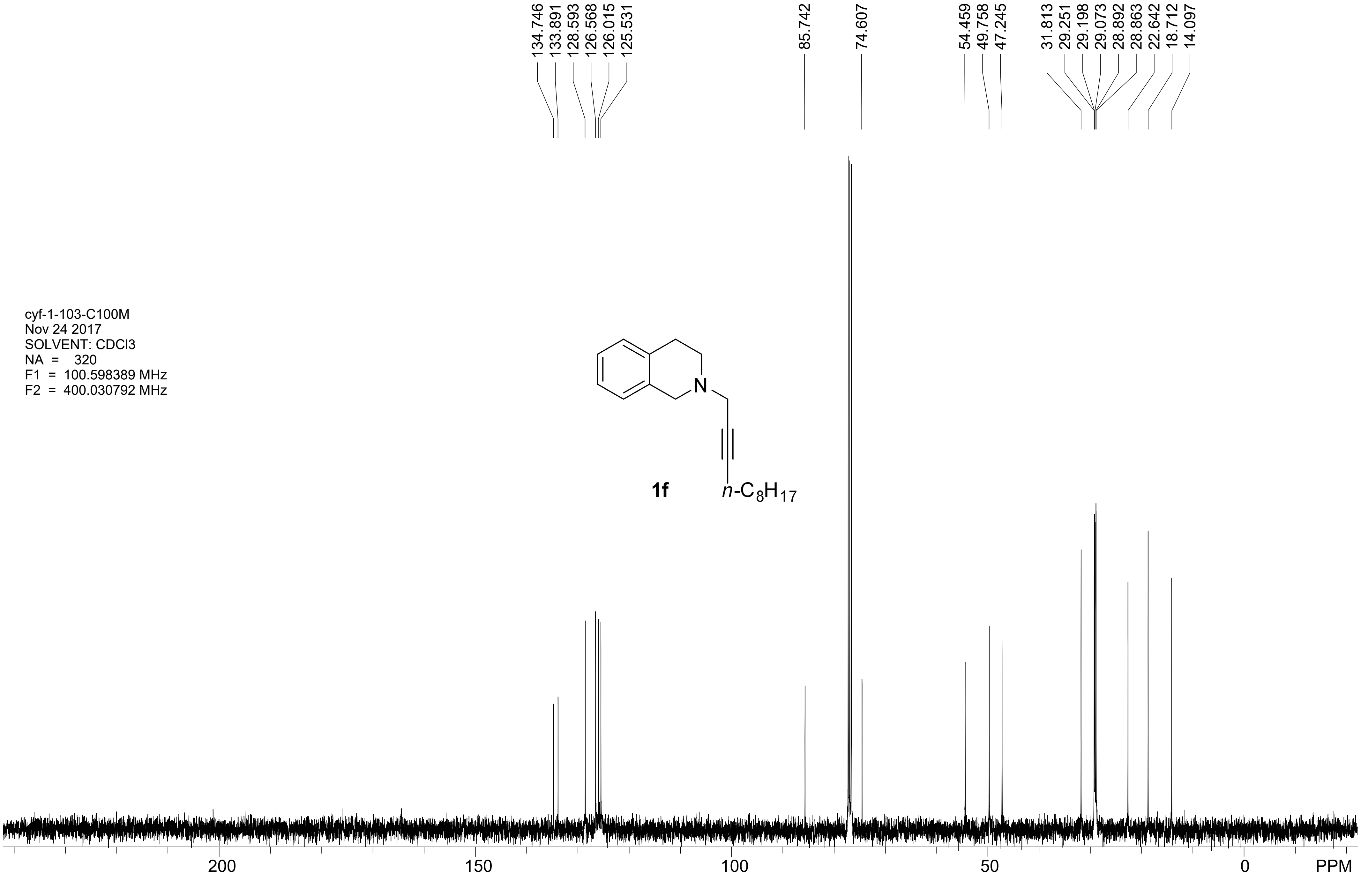
134.746
133.891
128.593
126.568
126.015
125.531
85.742
74.607
54.459
49.758
47.245
31.813
29.251
29.198
29.073
28.892
28.863
22.642
18.712
14.097
cyf-1-103-C100M
Nov 24 2017
SOLVENT: CDCl3
NA = 320
F1 = 100.598389 MHz
F2 = 400.030792 MHz
N
1f
n-C8H17
200
150
100
50
0
PPM

cyf-2-94-h
Oct 12 2018
SOLVENT: CDCl3
NA = 8
F1 = 399.722626 MHz
F2 = 100.519203 MHz

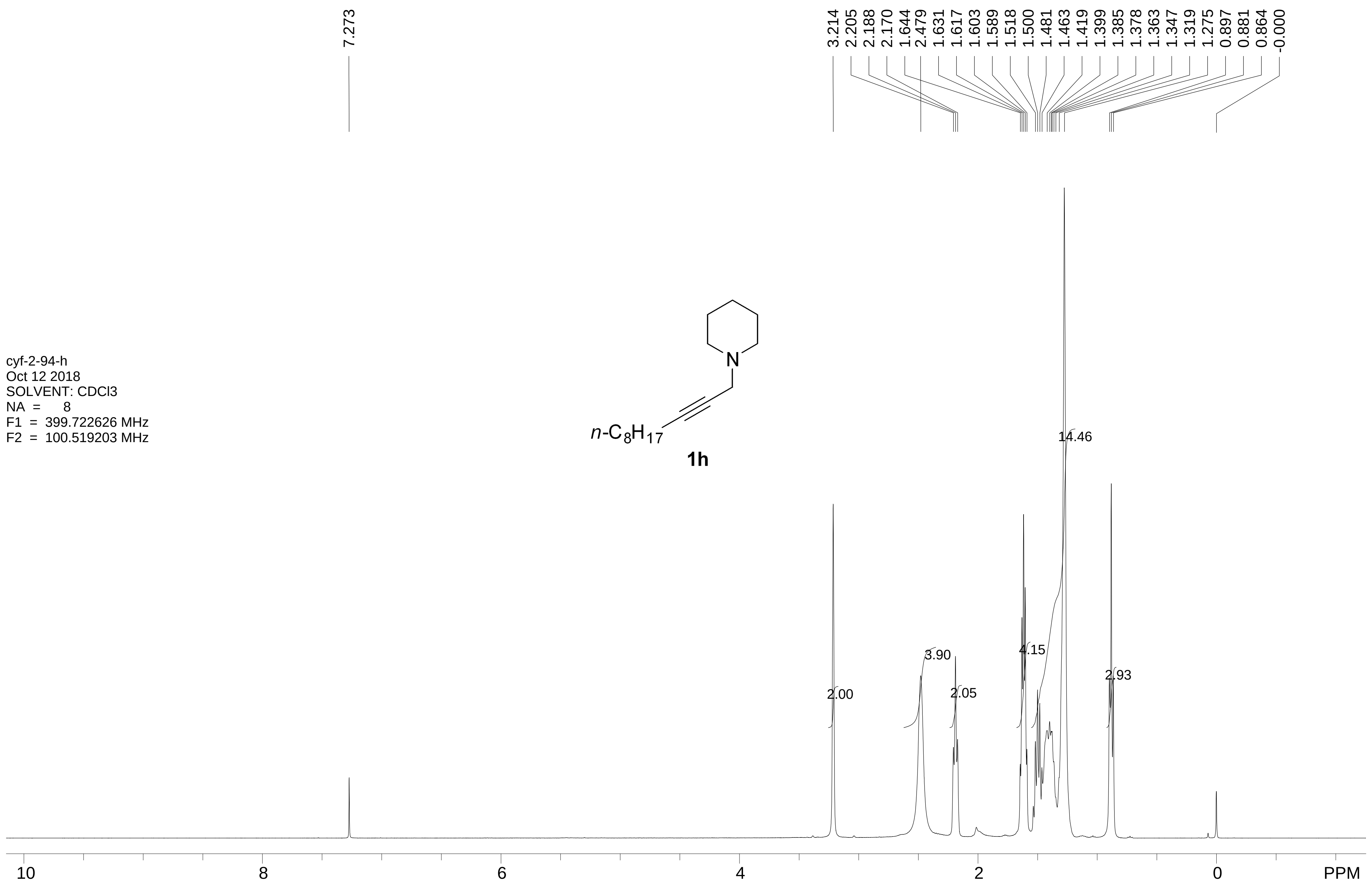

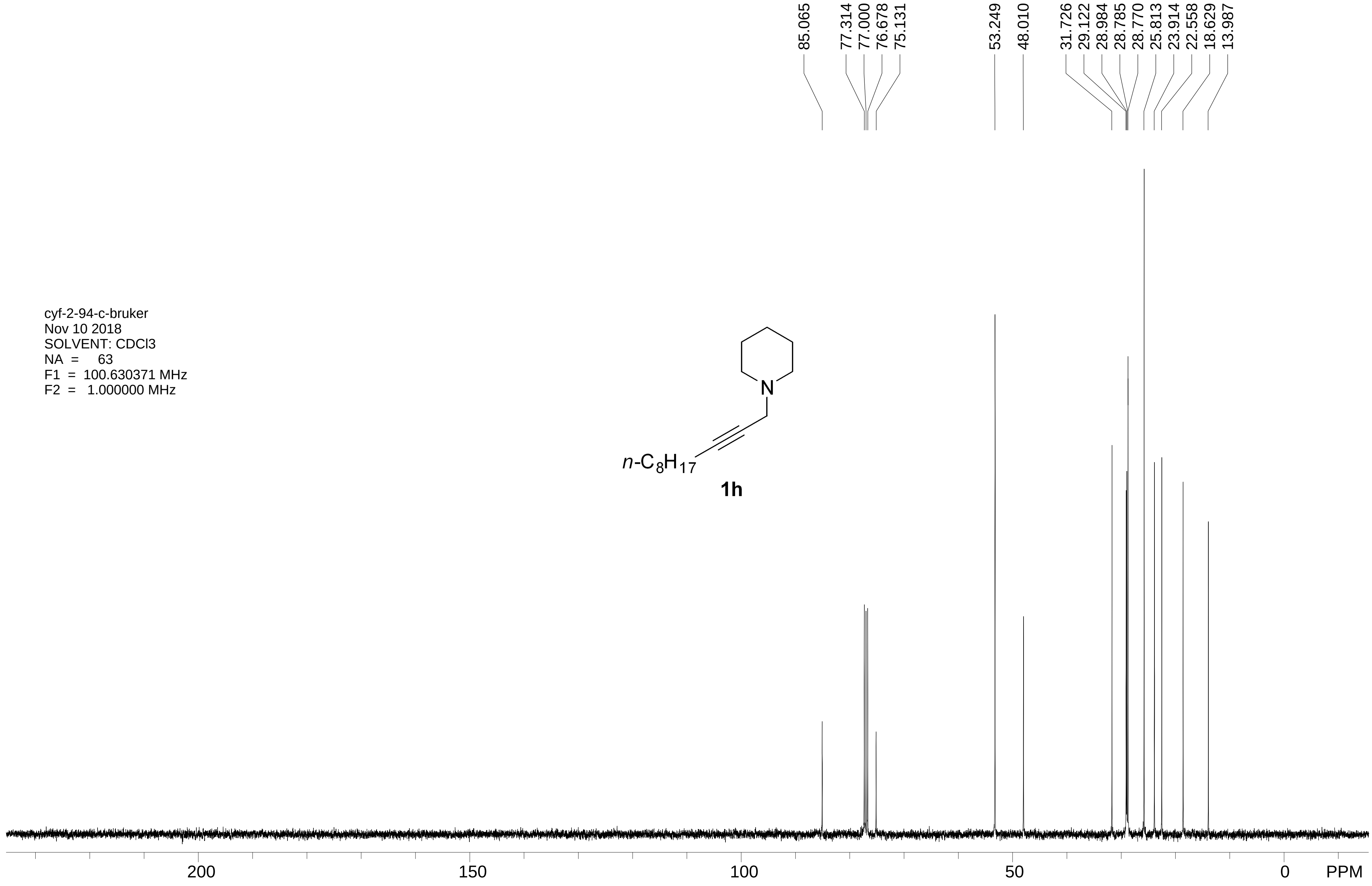
85.065
77.314
77.000
76.678
75.131
53.249
48.010
31.726
29.122
28.984
28.785
28.770
25.813
23.914
22.558
18.629
13.987
cyf-2-94-c-bruker
Nov 10 2018
SOLVENT: CDCl3
NA = 63
F1 = 100.630371 MHz
F2 = 1.000000 MHz
N
n-C8H17
1h
200
150
100
50
0
PPM

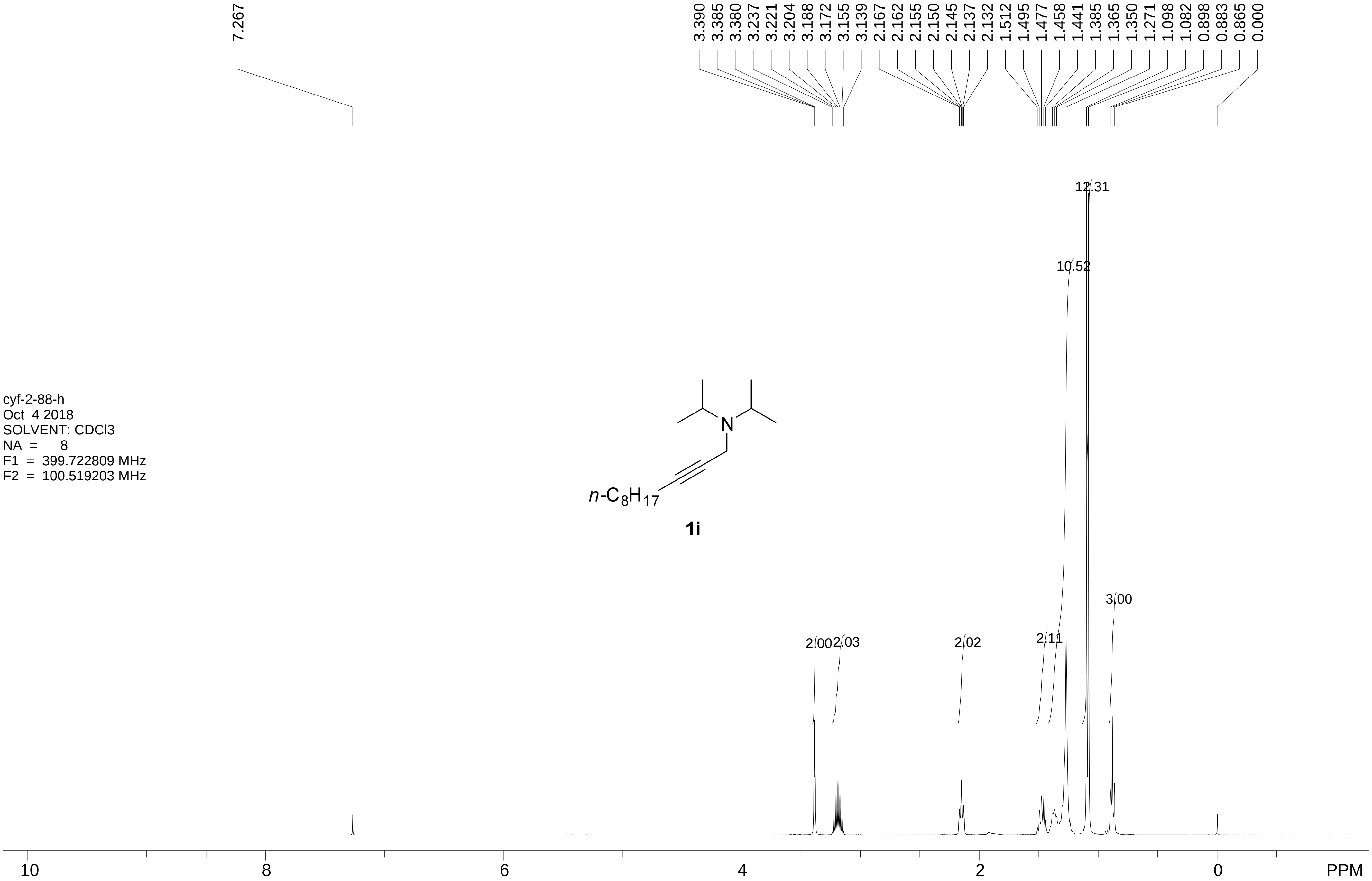

cyf-2-88-h
Oct 4 2018
SOLVENT: CDCl3
NA = 8
F1 = 399.722809 MHz
F2 = 100.519203 MHz

1i

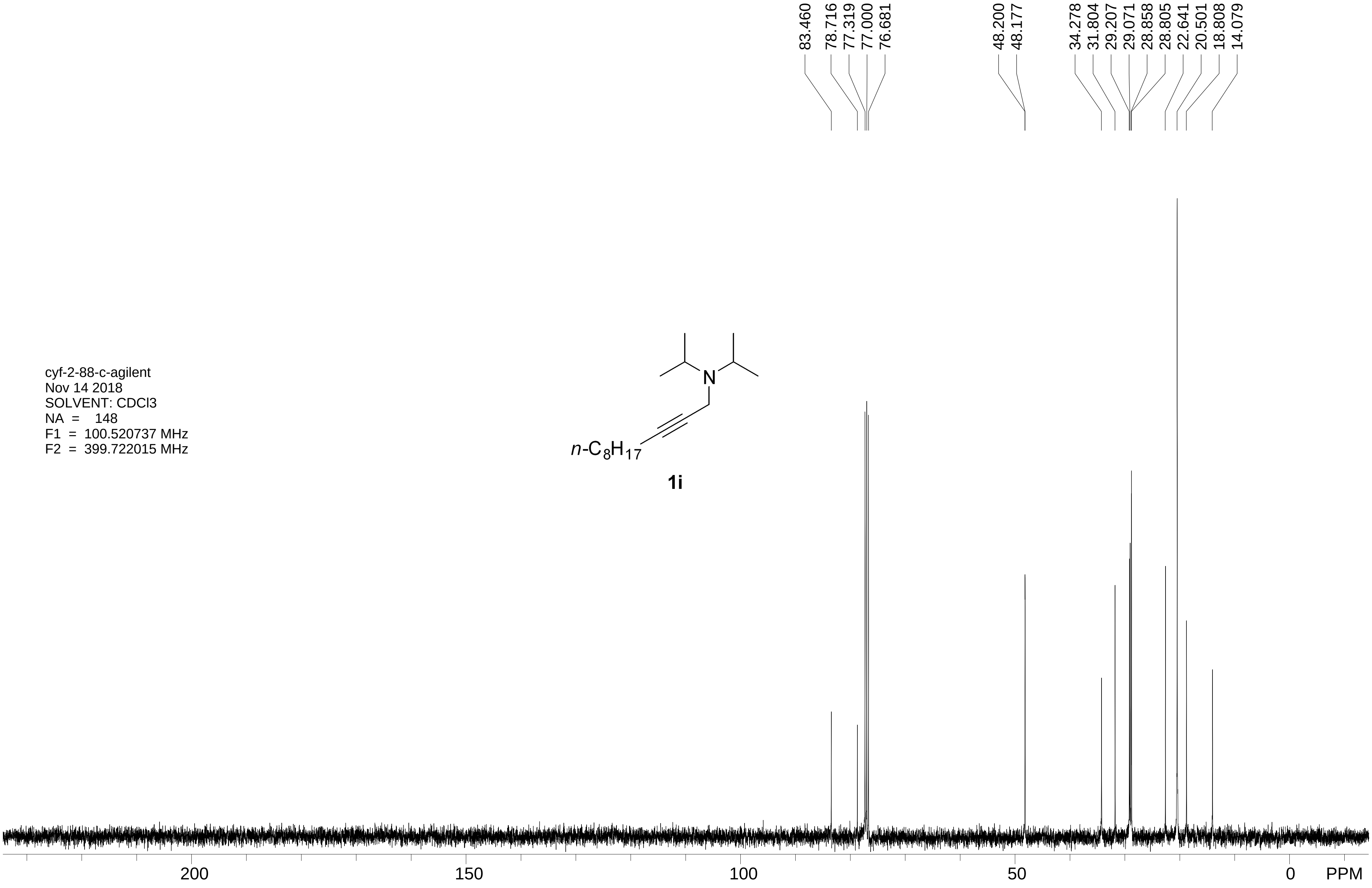
83.460
78.716
77.319
77.000
76.681
48.200
48.177
34.278
31.804
29.207
29.071
28.858
28.805
22.641
20.501
18.808
14.079
cyf-2-88-c-agilent
Nov 14 2018
SOLVENT: CDCl3
NA = 148
F1 = 100.520737 MHz
F2 = 399.722015 MHz
N
n-C8H17
1i
200
150
100
50
0
PPM

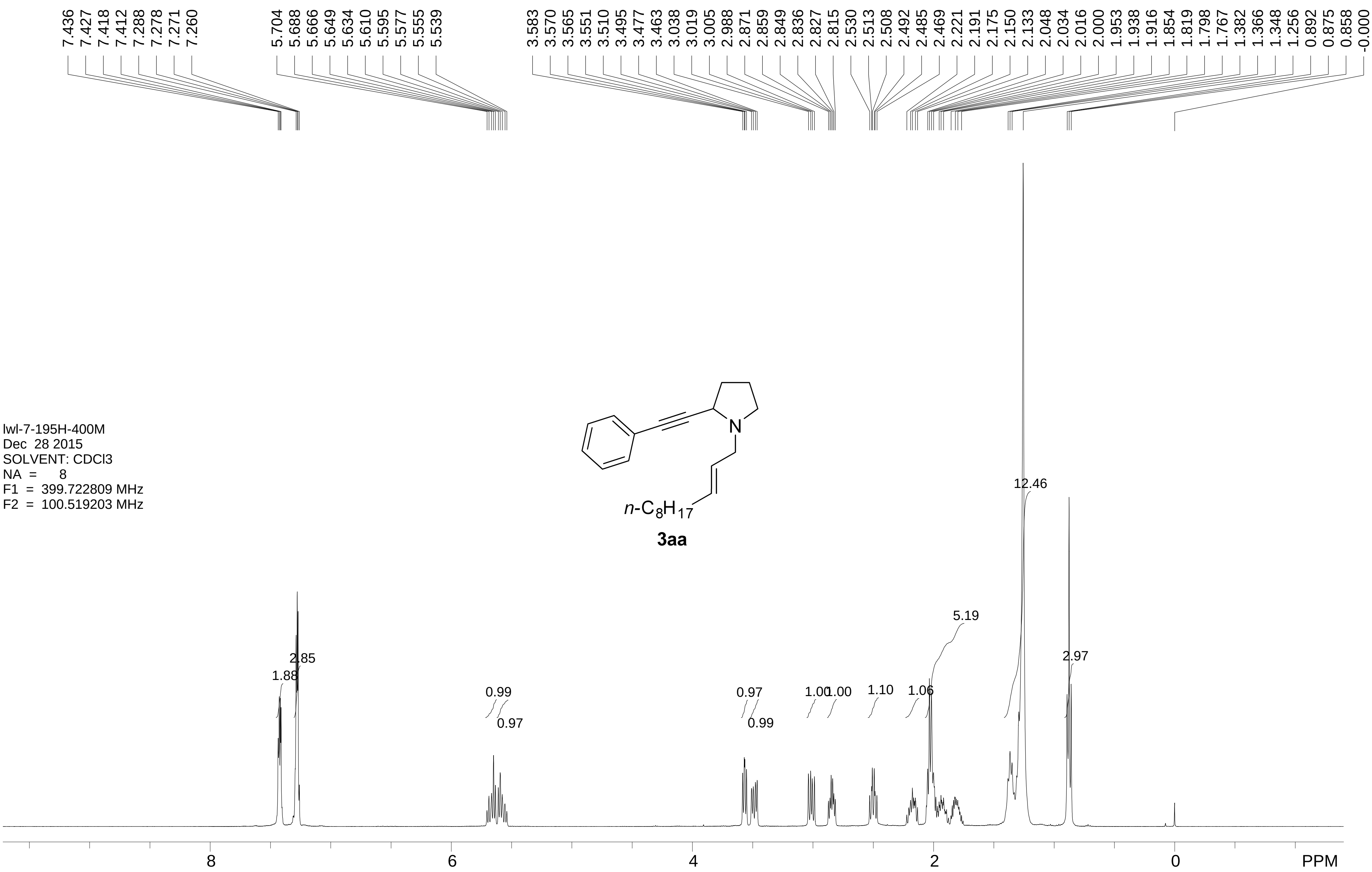
7.436 7.427 7.418 7.412 7.288 7.278 7.271 7.260
5.704 5.688 5.666 5.649 5.634 5.610 5.595 5.577 5.555 5.539
3.583 3.570 3.565 3.551 3.510 3.495 3.477 3.463 3.038 3.019 3.005 2.988 2.871 2.859 2.849 2.836 2.827 2.815 2.530 2.513 2.508 2.492 2.485 2.469 2.221 2.191 2.175 2.150 2.133 2.048 2.034 2.016 2.000 1.953 1.938 1.916 1.854 1.819 1.798 1.767 1.382 1.366 1.348 1.256 0.892 0.875 0.858 -0.000
lwl-7-195H-400M
Dec 28 2015
SOLVENT: CDCl3
NA = 8
F1 = 399.722809 MHz
F2 = 100.519203 MHz
N
n-C8H17
3aa
12.46
5.19
2.85
1.88
2.97
0.99
0.97
0.97
0.99
1.00
1.00
1.10
1.06
8
6
4
2
0
PPM

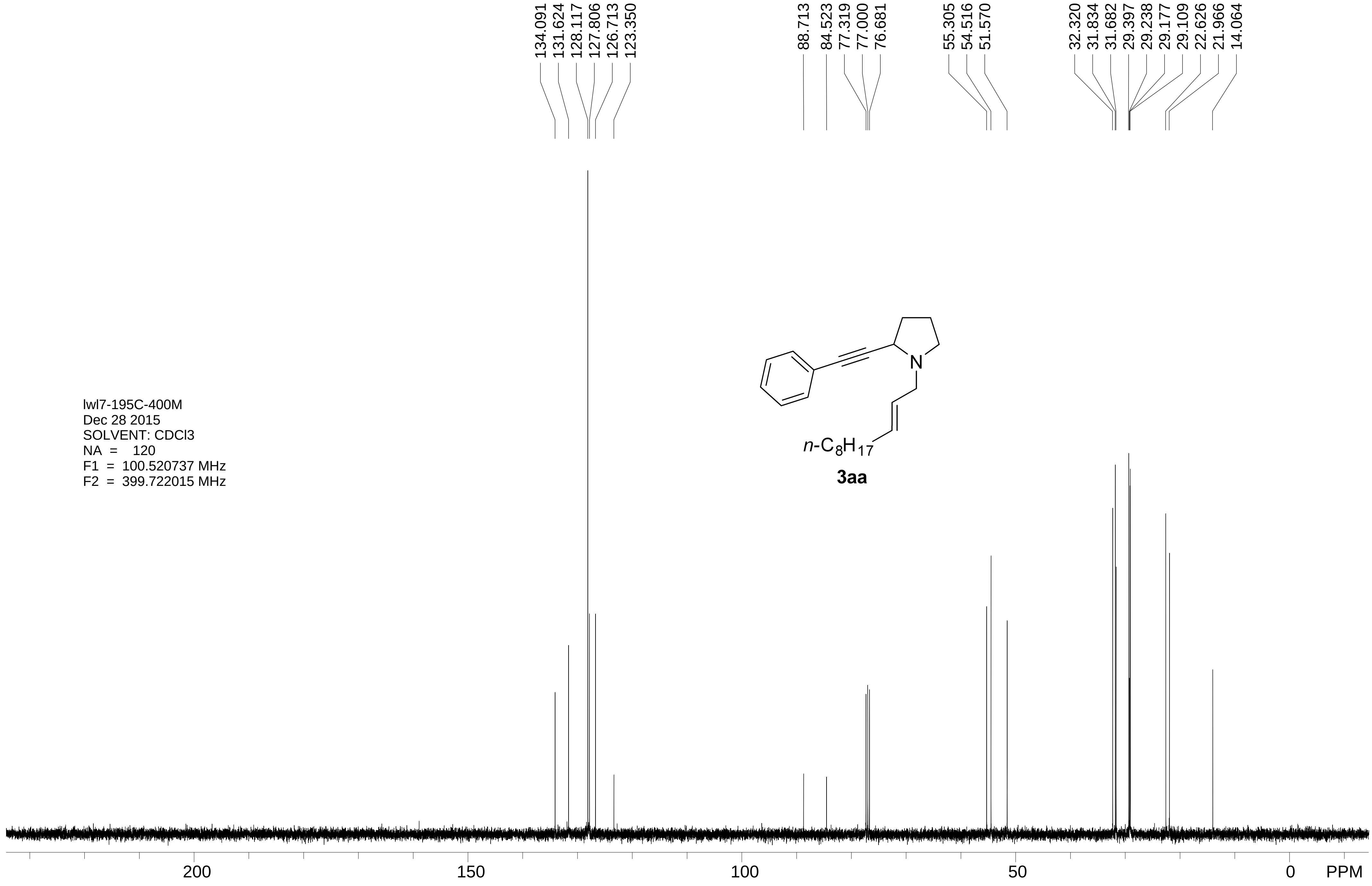
134.091
131.624
128.117
127.806
126.713
123.350
88.713
84.523
77.319
77.000
76.681
55.305
54.516
51.570
32.320
31.834
31.682
29.397
29.238
29.177
29.109
22.626
21.966
14.064
lwl7-195C-400M
Dec 28 2015
SOLVENT: CDCl3
NA = 120
F1 = 100.520737 MHz
F2 = 399.722015 MHz
N
n-C8H17
3aa
200
150
100
50
0
PPM

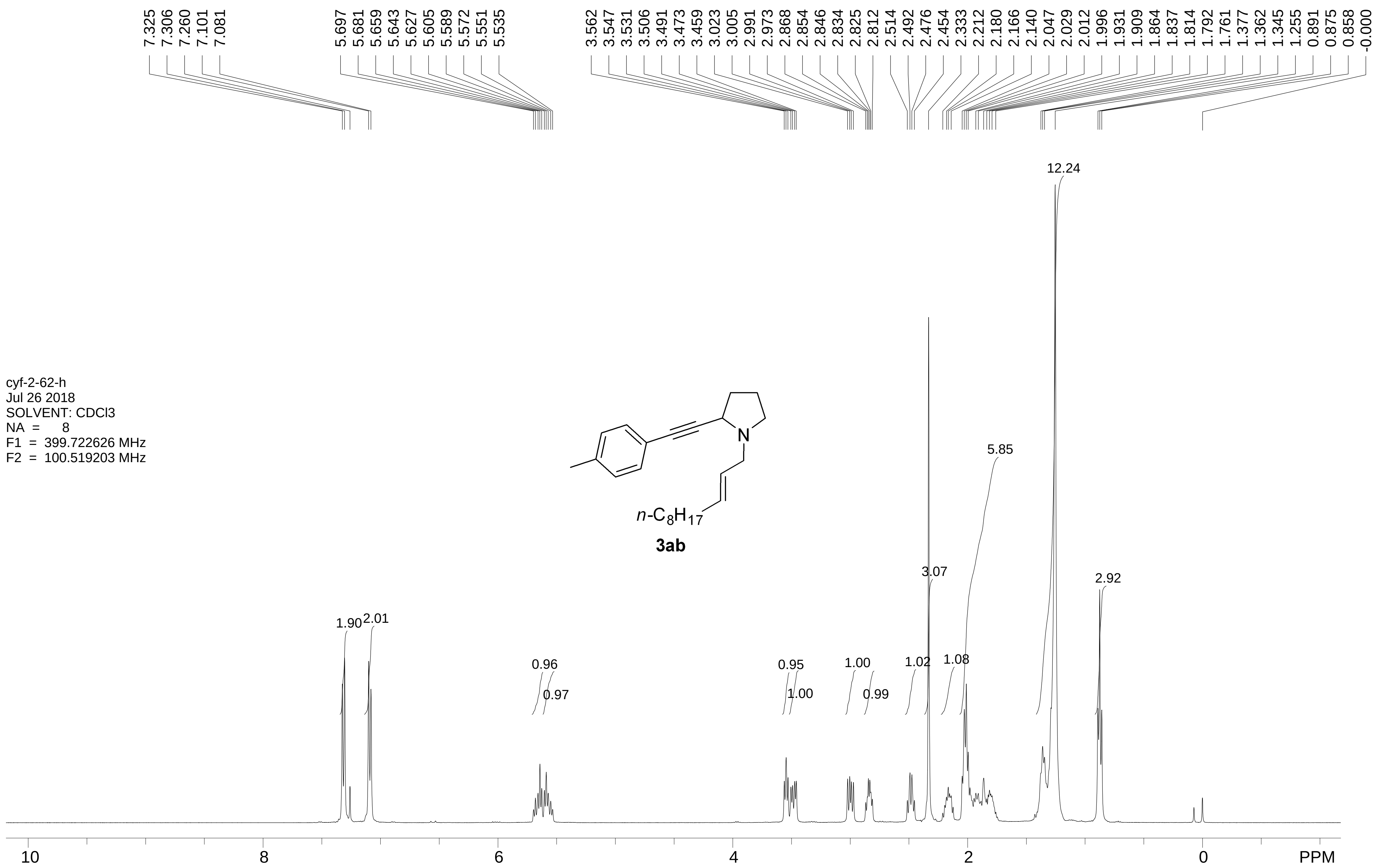
7.325 7.306 7.260 7.101 7.081
5.697 5.681 5.659 5.643 5.627 5.605 5.589 5.572 5.551 5.535
3.562 3.547 3.531 3.506 3.491 3.473 3.459 3.023 3.005 2.991 2.973 2.868 2.854 2.846 2.834 2.825 2.812 2.514 2.492 2.476 2.454 2.333 2.212 2.180 2.166 2.140 2.047 2.029 2.012 1.996 1.931 1.909 1.864 1.837 1.814 1.792 1.761 1.377 1.362 1.345 1.255 0.891 0.875 0.858 -0.000
cyf-2-62-h
Jul 26 2018
SOLVENT: CDCl3
NA = 8
F1 = 399.722626 MHz
F2 = 100.519203 MHz
N
n-C8H17
3ab
12.24
5.85
3.07
2.92
1.90
2.01
0.96
0.97
0.95
1.00
1.00
0.99
1.02
1.08
10
8
6
4
2
0
PPM

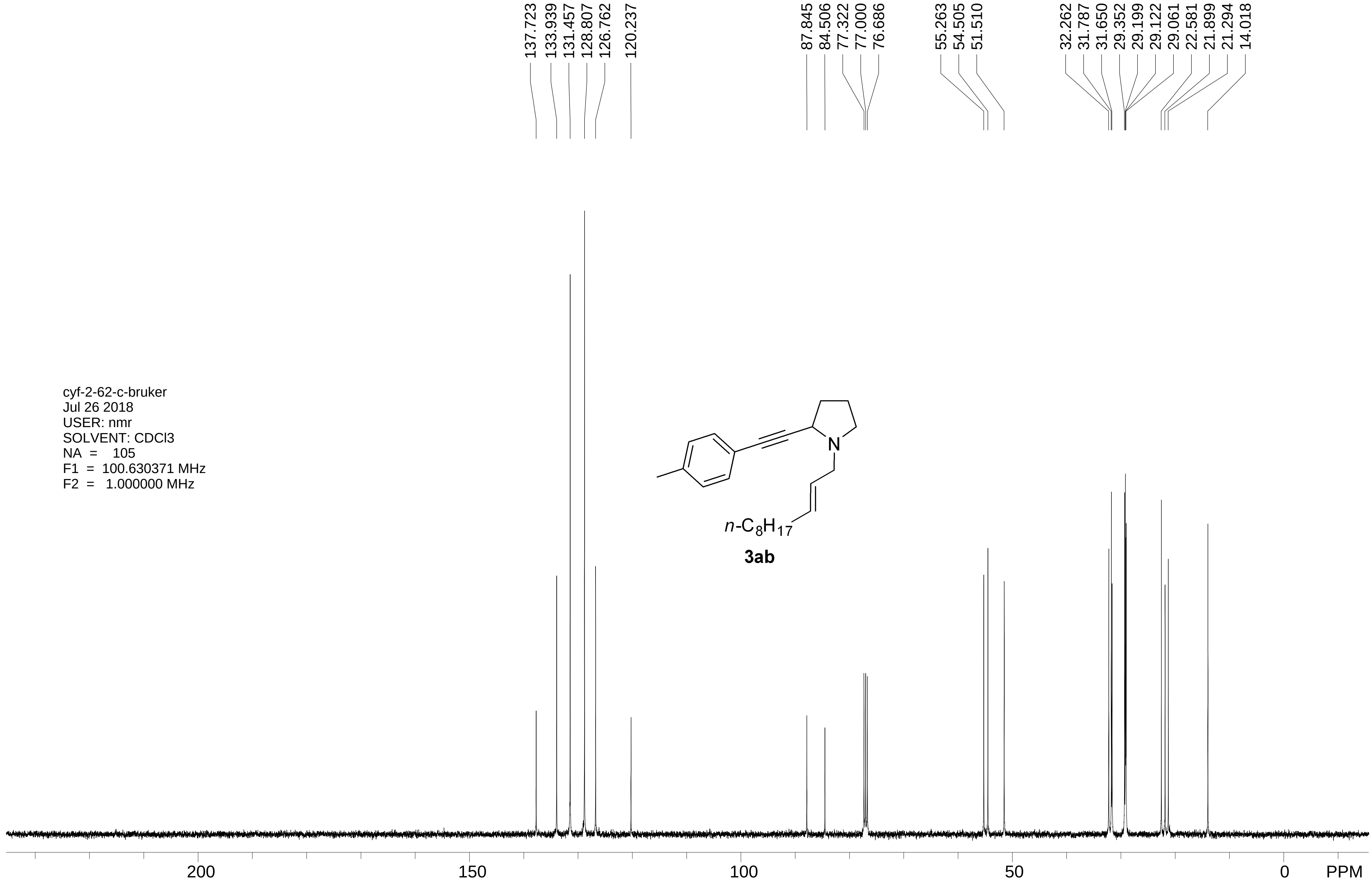
137.723
133.939
131.457
128.807
126.762
120.237
87.845
84.506
77.322
77.000
76.686
55.263
54.505
51.510
32.262
31.787
31.650
29.352
29.199
29.122
29.061
22.581
21.899
21.294
14.018
cyf-2-62-c-bruker
Jul 26 2018
USER: nmr
SOLVENT: CDCl3
NA = 105
F1 = 100.630371 MHz
F2 = 1.000000 MHz
N
n-C8H17
3ab
200
150
100
50
0
PPM

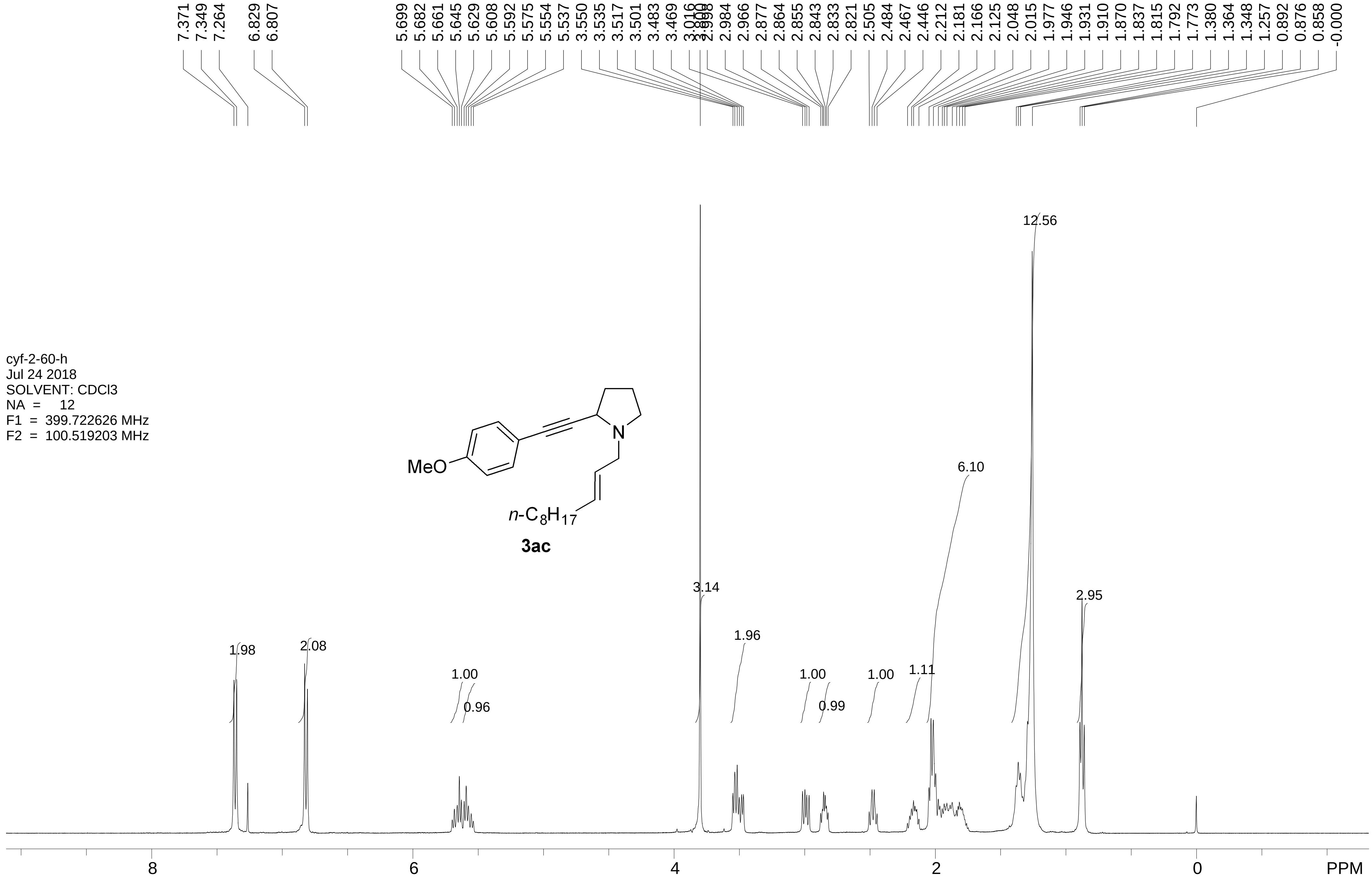
7.371
7.349
7.264
6.829
6.807
5.699
5.682
5.661
5.645
5.629
5.608
5.592
5.575
5.554
5.537
3.550
3.535
3.517
3.501
3.483
3.469
3.016
2.998
2.984
2.966
2.877
2.864
2.855
2.843
2.833
2.821
2.505
2.484
2.467
2.446
2.212
2.181
2.166
2.125
2.048
2.015
1.977
1.946
1.931
1.910
1.870
1.837
1.815
1.792
1.773
1.380
1.364
1.348
1.257
0.892
0.876
0.858
-0.000
cyf-2-60-h
Jul 24 2018
SOLVENT: CDCl3
NA = 12
F1 = 399.722626 MHz
F2 = 100.519203 MHz
MeO
N
n-C8H17
3ac
1.98
2.08
1.00
0.96
3.14
1.96
1.00
0.99
1.00
1.11
6.10
12.56
2.95
8
6
4
2
0
PPM

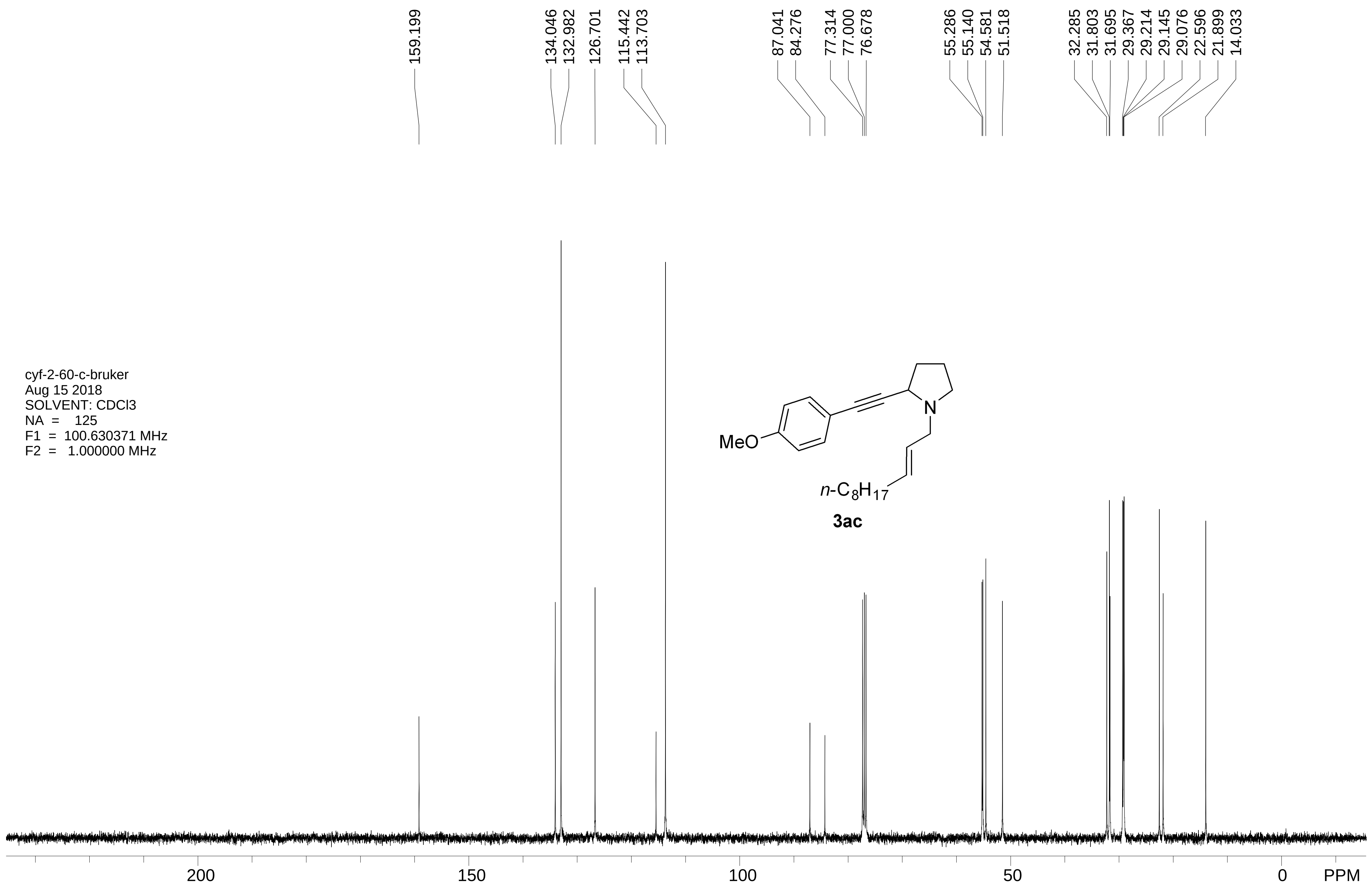
159.199
134.046
132.982
126.701
115.442
113.703
87.041
84.276
77.314
77.000
76.678
55.286
55.140
54.581
51.518
32.285
31.803
31.695
29.367
29.214
29.145
29.076
22.596
21.899
14.033
cyf-2-60-c-bruker
Aug 15 2018
SOLVENT: CDCl3
NA = 125
F1 = 100.630371 MHz
F2 = 1.000000 MHz
MeO
N
n-C8H17
3ac
200
150
100
50
0
PPM

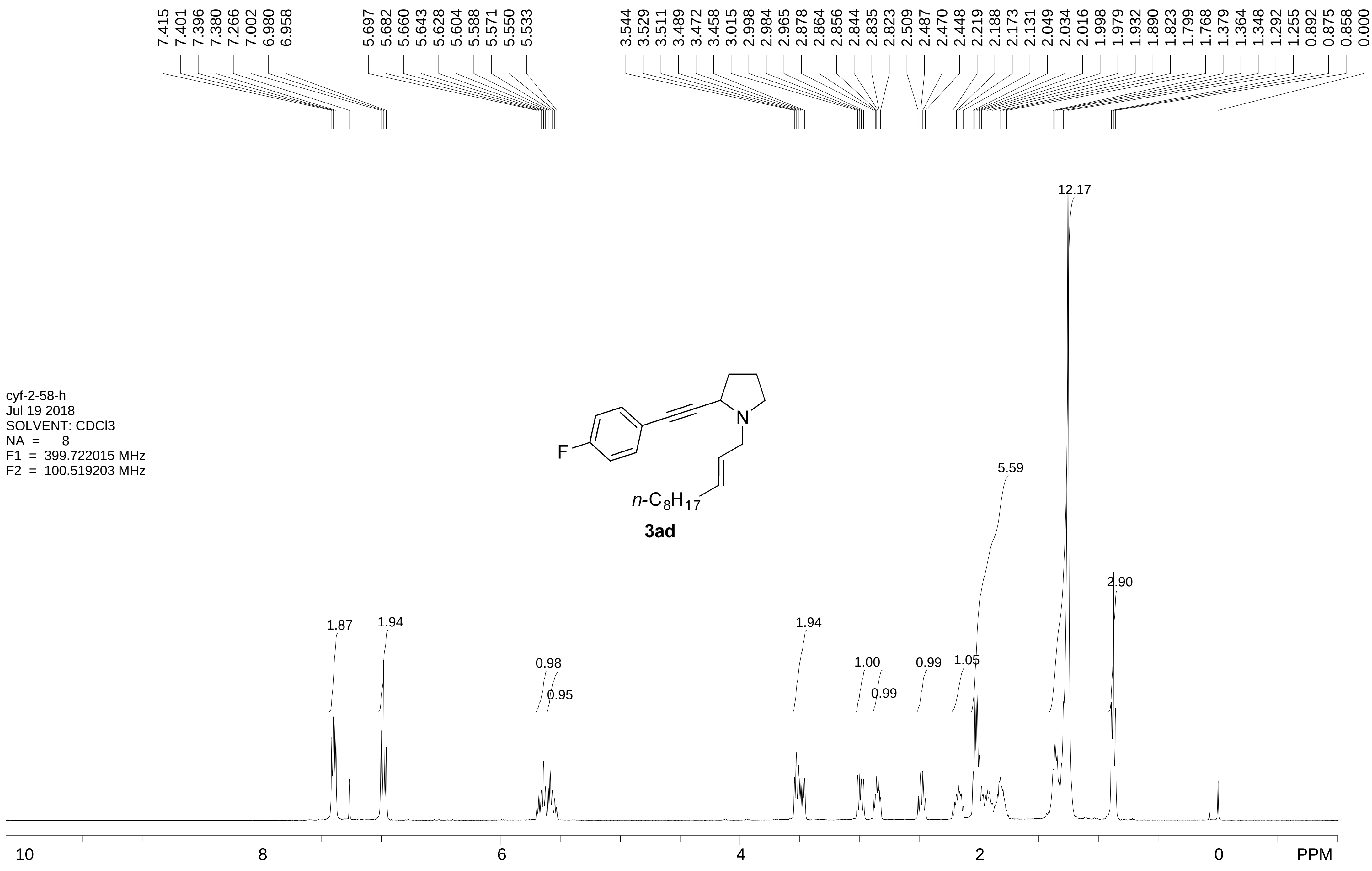
7.415 7.401 7.396 7.380 7.266 7.002 6.980 6.958
5.697 5.682 5.660 5.643 5.628 5.604 5.588 5.571 5.550 5.533
3.544 3.529 3.511 3.489 3.472 3.458 3.015 2.998 2.984 2.965 2.878 2.864 2.856 2.844 2.835 2.823 2.509 2.487 2.470 2.448 2.219 2.188 2.173 2.131 2.049 2.034 2.016 1.998 1.979 1.932 1.890 1.823 1.799 1.768 1.379 1.364 1.348 1.292 1.255 0.892 0.875 0.858 0.000
12.17
5.59
2.90
1.87
1.94
0.98
0.95
1.94
1.00
0.99
0.99
1.05
cyf-2-58-h
Jul 19 2018
SOLVENT: CDCl3
NA = 8
F1 = 399.722015 MHz
F2 = 100.519203 MHz
F
N
n-C8H17
3ad
10
8
6
4
2
0
PPM

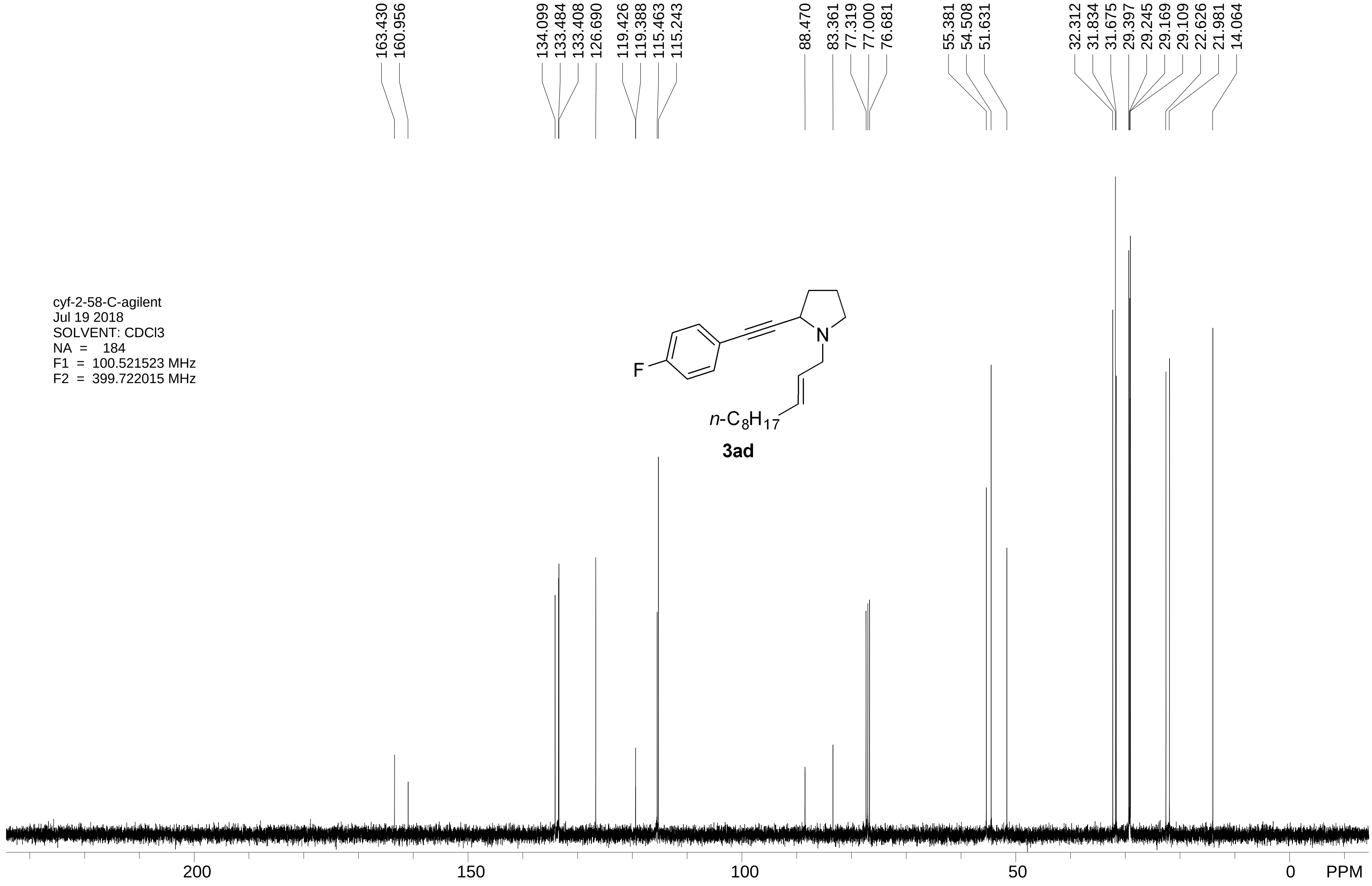
163.430
160.956
134.099
133.484
133.408
126.690
119.426
119.388
115.463
115.243
88.470
83.361
77.319
77.000
76.681
55.381
54.508
51.631
32.312
31.834
31.675
29.397
29.245
29.169
29.109
22.626
21.981
14.064
cyf-2-58-C-agilent
Jul 19 2018
SOLVENT: CDCl3
NA = 184
F1 = 100.521523 MHz
F2 = 399.722015 MHz
F
N
n-C8H17
3ad
200
150
100
50
0
PPM

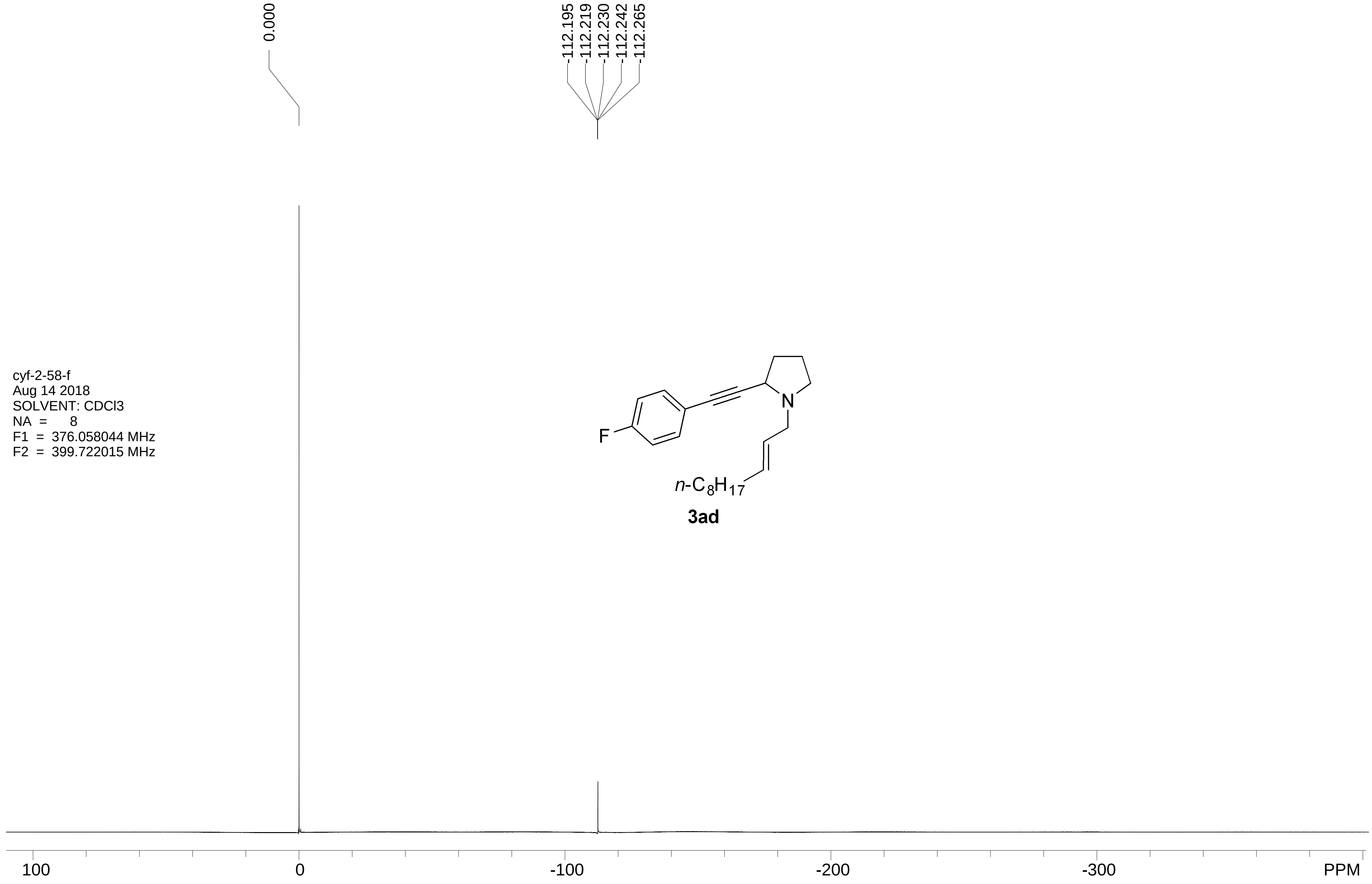
0.000
-112.195
-112.219
-112.230
-112.242
-112.265
cyf-2-58-f
Aug 14 2018
SOLVENT: CDCl3
NA = 8
F1 = 376.058044 MHz
F2 = 399.722015 MHz
F
N
n-C8H17
3ad
100
0
-100
-200
-300
PPM

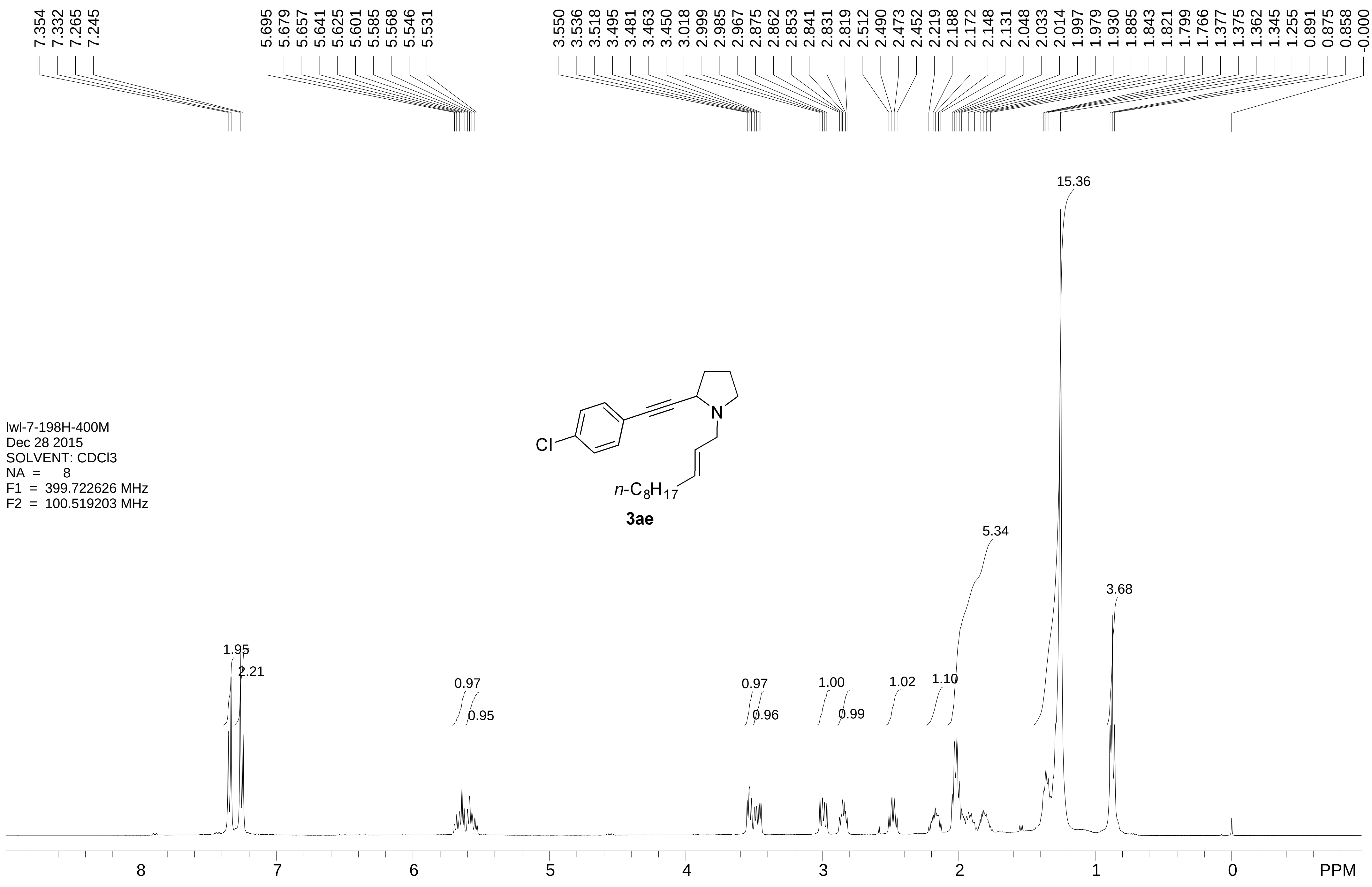
7.354 7.332 7.265 7.245
5.695 5.679 5.657 5.641 5.625 5.601 5.585 5.568 5.546 5.531
3.550 3.536 3.518 3.495 3.481 3.463 3.450 3.018 2.999 2.985 2.967 2.875 2.862 2.853 2.841 2.831 2.819 2.512 2.490 2.473 2.452 2.219 2.188 2.172 2.148 2.131 2.048 2.033 2.014 1.997 1.979 1.930 1.885 1.843 1.821 1.799 1.766 1.377 1.375 1.362 1.345 1.255 0.891 0.875 0.858 -0.000
lwl-7-198H-400M
Dec 28 2015
SOLVENT: CDCl3
NA = 8
F1 = 399.722626 MHz
F2 = 100.519203 MHz
Cl
N
n-C8H17
3ae
15.36
5.34
3.68
1.95
2.21
0.97
0.95
0.97
0.96
1.00
0.99
1.02
1.10
8
7
6
5
4
3
2
1
0
PPM

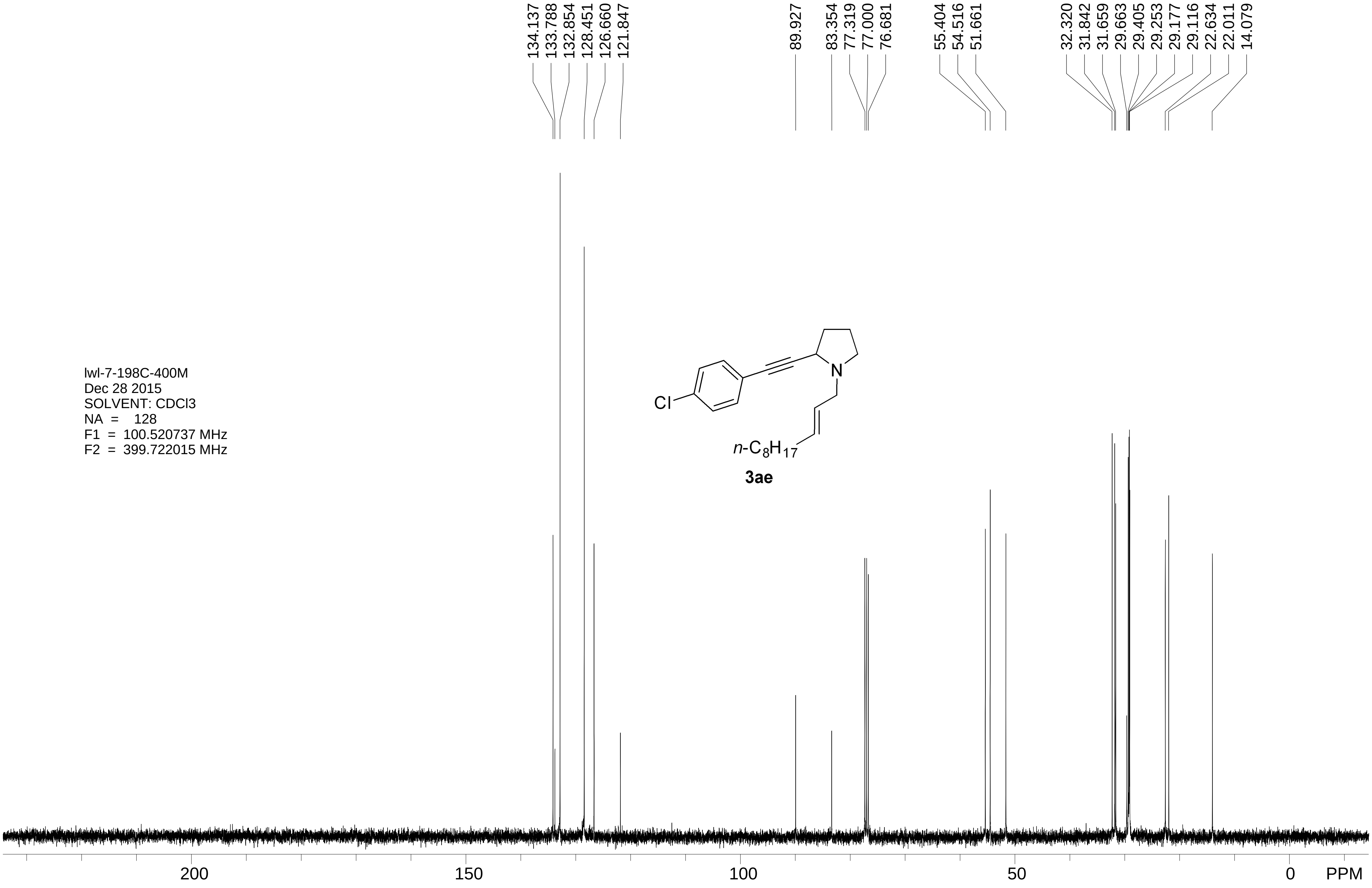
134.137
133.788
132.854
128.451
126.660
121.847
89.927
83.354
77.319
77.000
76.681
55.404
54.516
51.661
32.320
31.842
31.659
29.663
29.405
29.253
29.177
29.116
22.634
22.011
14.079
lwl-7-198C-400M
Dec 28 2015
SOLVENT: CDCl3
NA = 128
F1 = 100.520737 MHz
F2 = 399.722015 MHz
Cl
N
n-C8H17
3ae
200
150
100
50
0
PPM

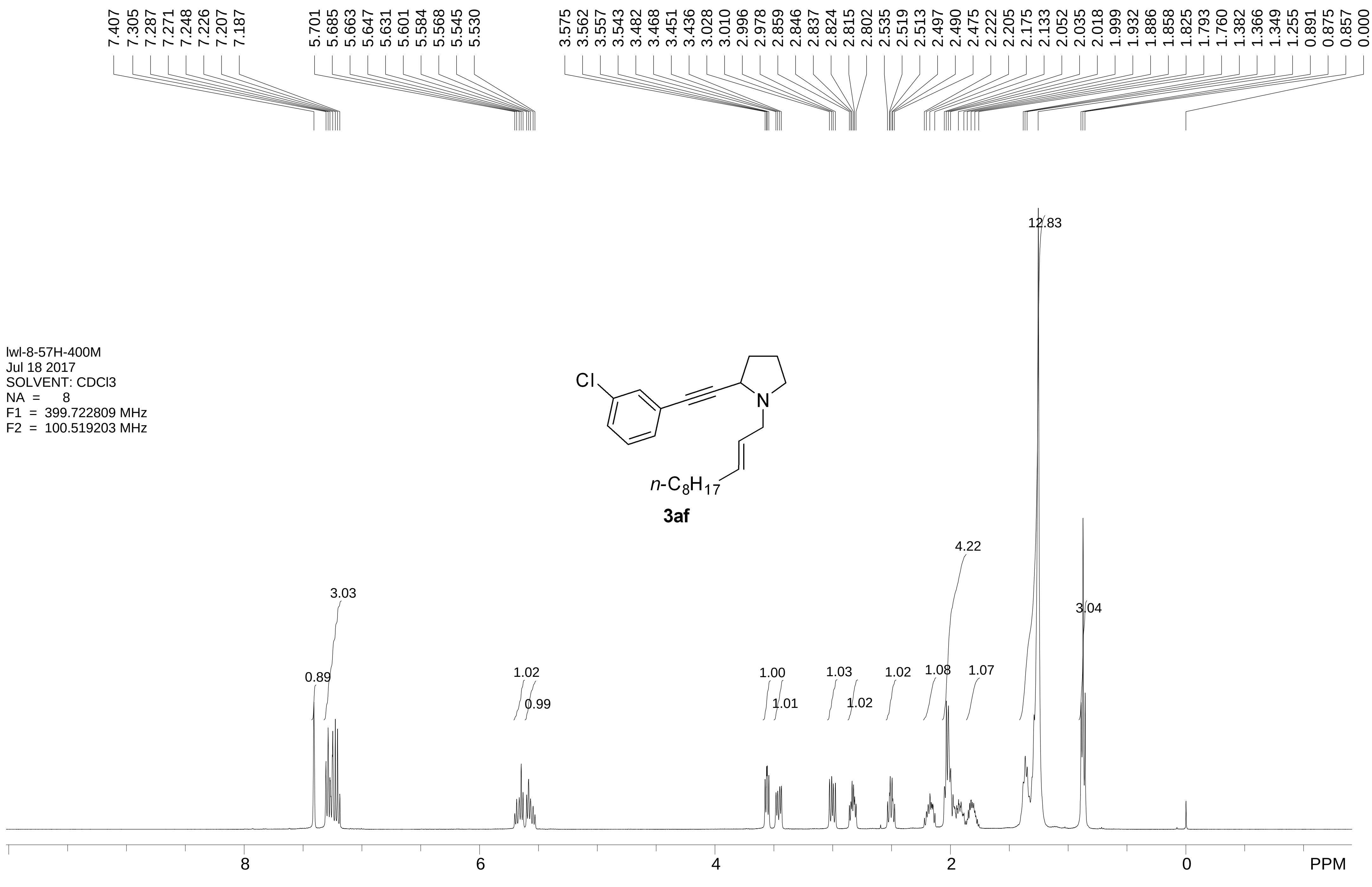
7.407 7.305 7.287 7.271 7.248 7.226 7.207 7.187
5.701 5.685 5.663 5.647 5.631 5.601 5.584 5.568 5.545 5.530
3.575 3.562 3.557 3.543 3.482 3.468 3.451 3.436 3.028 3.010 2.996 2.978 2.859 2.846 2.837 2.824 2.815 2.802 2.535 2.519 2.513 2.497 2.490 2.475 2.222 2.205 2.175 2.133 2.052 2.035 2.018 1.999 1.932 1.886 1.858 1.825 1.793 1.760 1.382 1.366 1.349 1.255 0.891 0.875 0.857 0.000
lwl-8-57H-400M
Jul 18 2017
SOLVENT: CDCl3
NA = 8
F1 = 399.722809 MHz
F2 = 100.519203 MHz
Cl
N
n-C8H17
3af
12.83
4.22
3.03
3.04
0.89
1.02
0.99
1.00
1.01
1.03
1.02
1.02
1.08
1.07
8
6
4
2
0
PPM

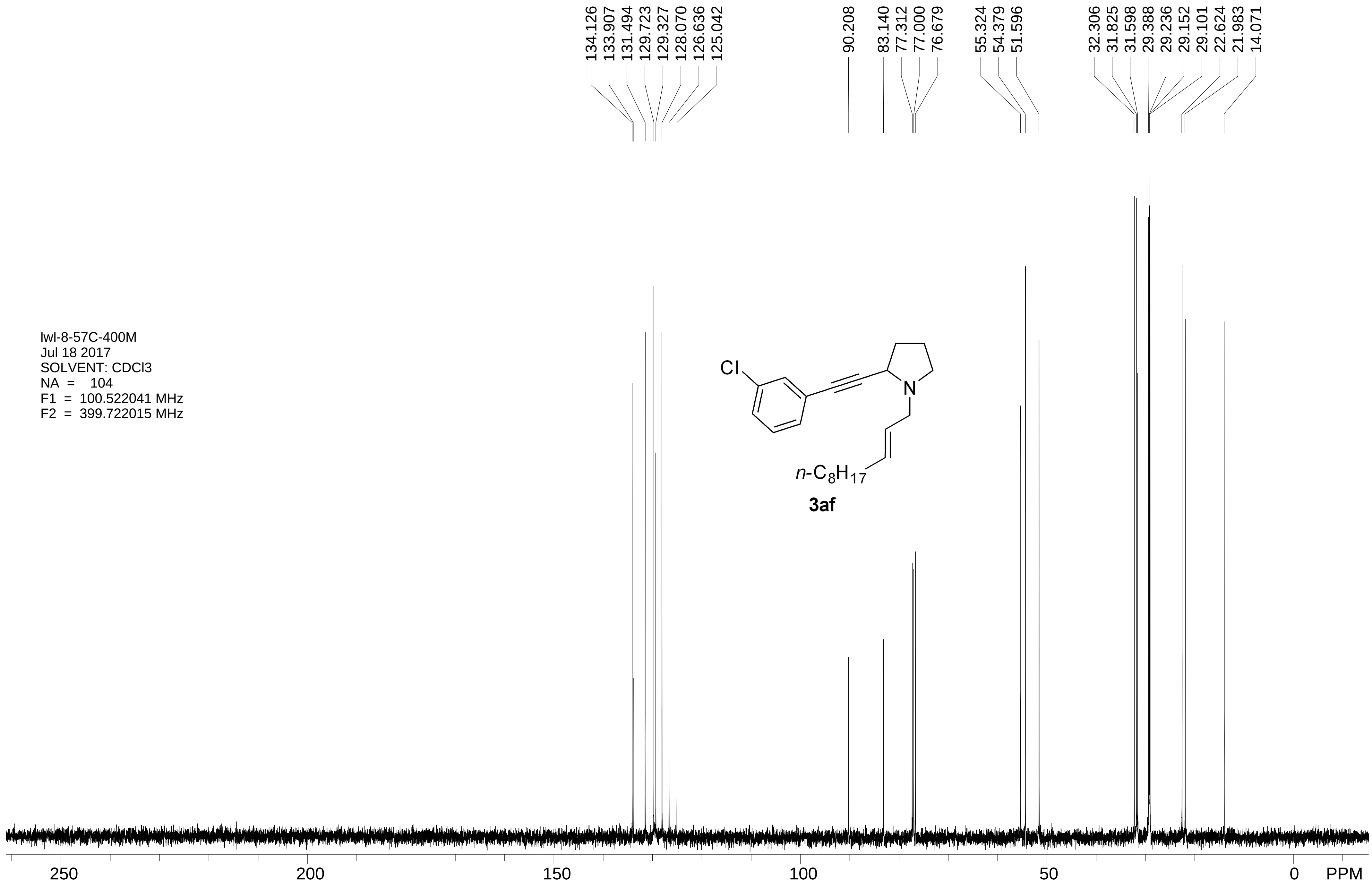
134.126
133.907
131.494
129.723
129.327
128.070
126.636
125.042
90.208
83.140
77.312
77.000
76.679
55.324
54.379
51.596
32.306
31.825
31.598
29.388
29.236
29.152
29.101
22.624
21.983
14.071
lwl-8-57C-400M
Jul 18 2017
SOLVENT: CDCl3
NA = 104
F1 = 100.522041 MHz
F2 = 399.722015 MHz
Cl
N
n-C8H17
3af
250
200
150
100
50
0
PPM

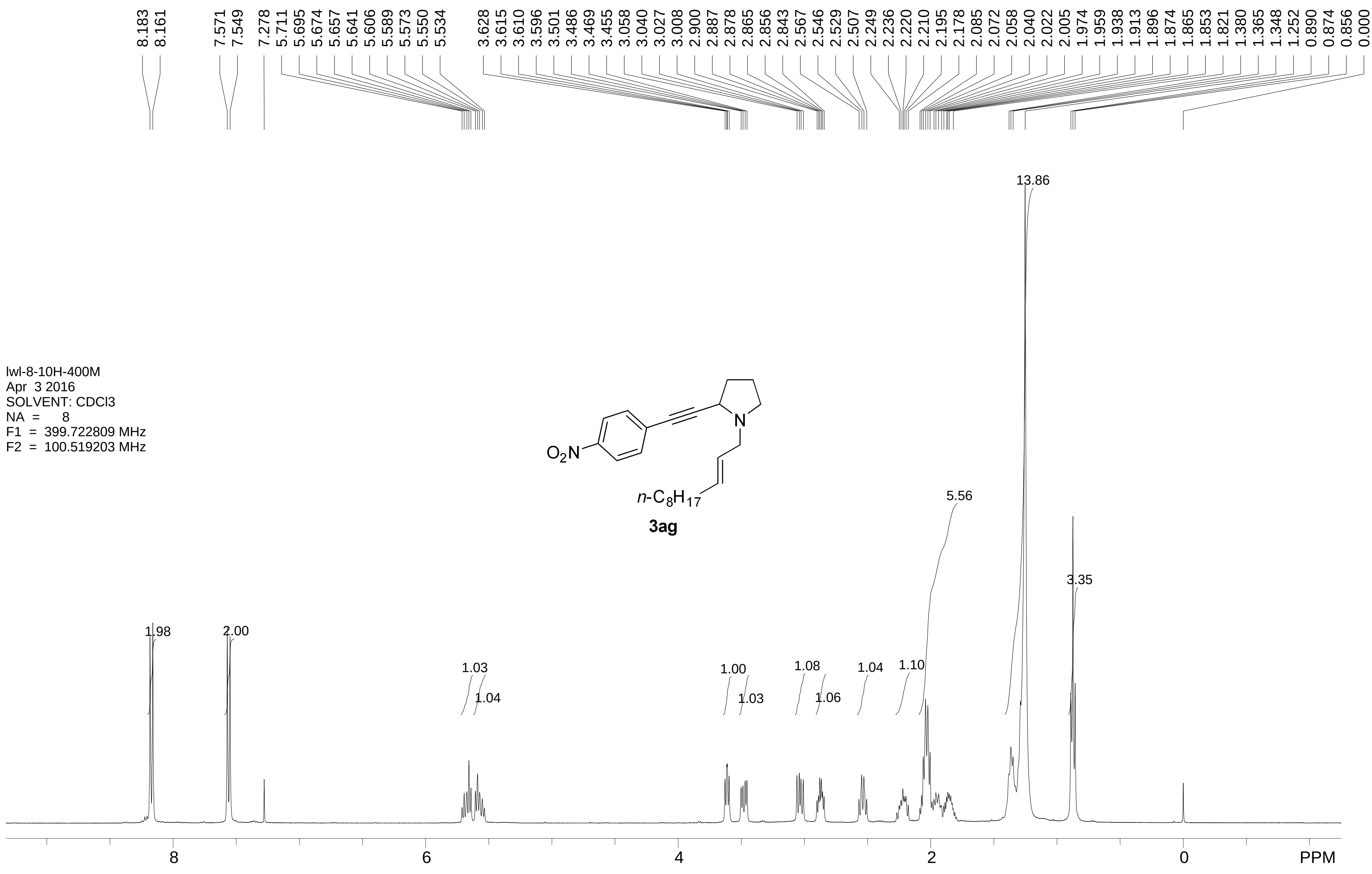
8.183
8.161
7.571
7.549
7.278
5.711
5.695
5.674
5.657
5.641
5.606
5.589
5.573
5.550
5.534
3.628
3.615
3.610
3.596
3.501
3.486
3.469
3.455
3.058
3.040
3.027
3.008
2.900
2.887
2.878
2.865
2.856
2.843
2.567
2.546
2.529
2.507
2.249
2.236
2.220
2.210
2.195
2.178
2.085
2.072
2.058
2.040
2.022
2.005
1.974
1.959
1.938
1.913
1.896
1.874
1.865
1.853
1.821
1.380
1.365
1.348
1.252
0.890
0.874
0.856
0.000
lwl-8-10H-400M
Apr 3 2016
SOLVENT: CDCl3
NA = 8
F1 = 399.722809 MHz
F2 = 100.519203 MHz
O2N
N
n-C8H17
3ag
13.86
5.56
3.35
1.98
2.00
1.03
1.04
1.00
1.03
1.08
1.06
1.04
1.10
8
6
4
2
0
PPM

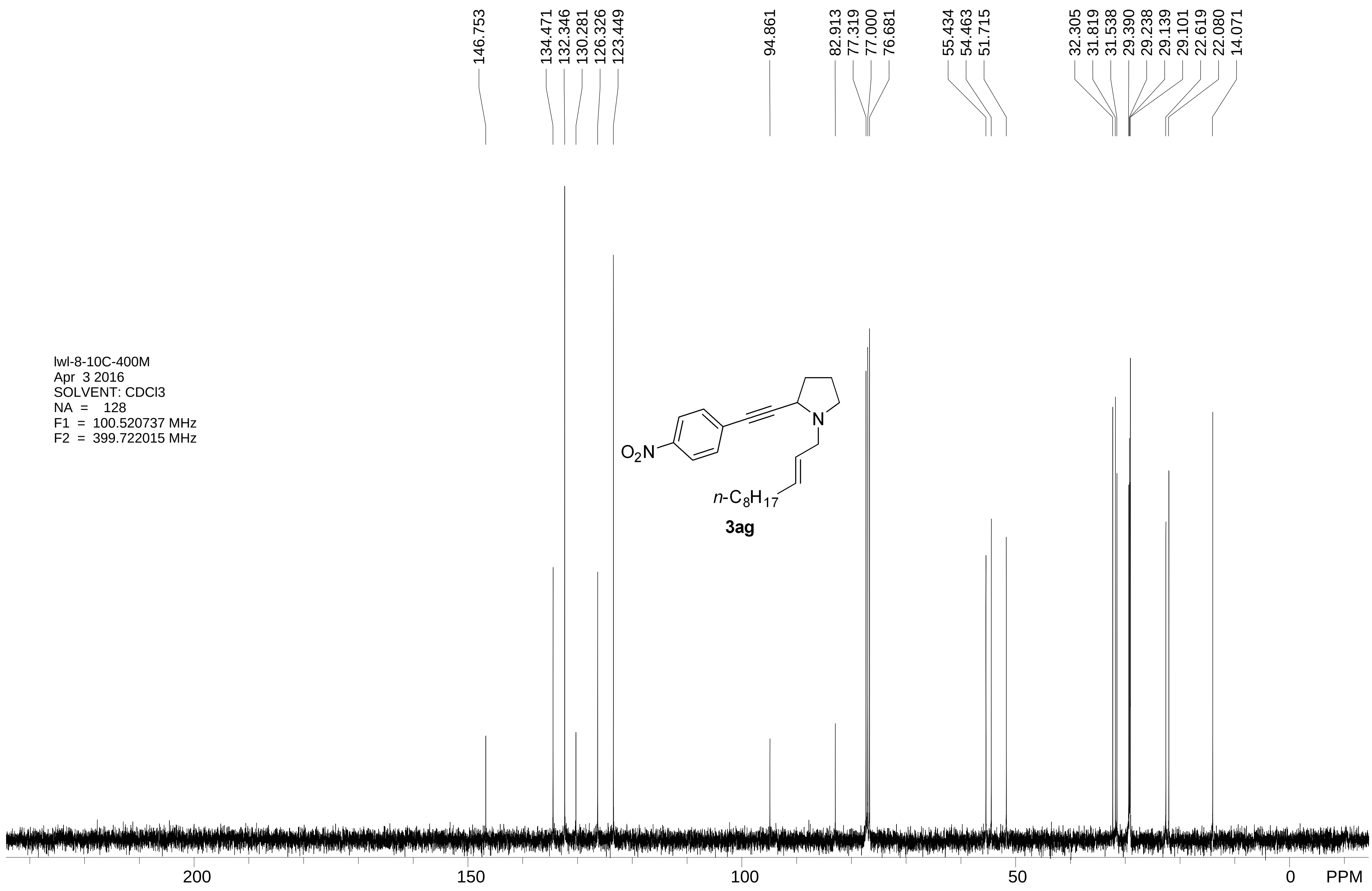
146.753
134.471
132.346
130.281
126.326
123.449
94.861
82.913
77.319
77.000
76.681
55.434
54.463
51.715
32.305
31.819
31.538
29.390
29.238
29.139
29.101
22.619
22.080
14.071
lwl-8-10C-400M
Apr 3 2016
SOLVENT: CDCl3
NA = 128
F1 = 100.520737 MHz
F2 = 399.722015 MHz
O2N
N
n-C8H17
3ag
200
150
100
50
0
PPM

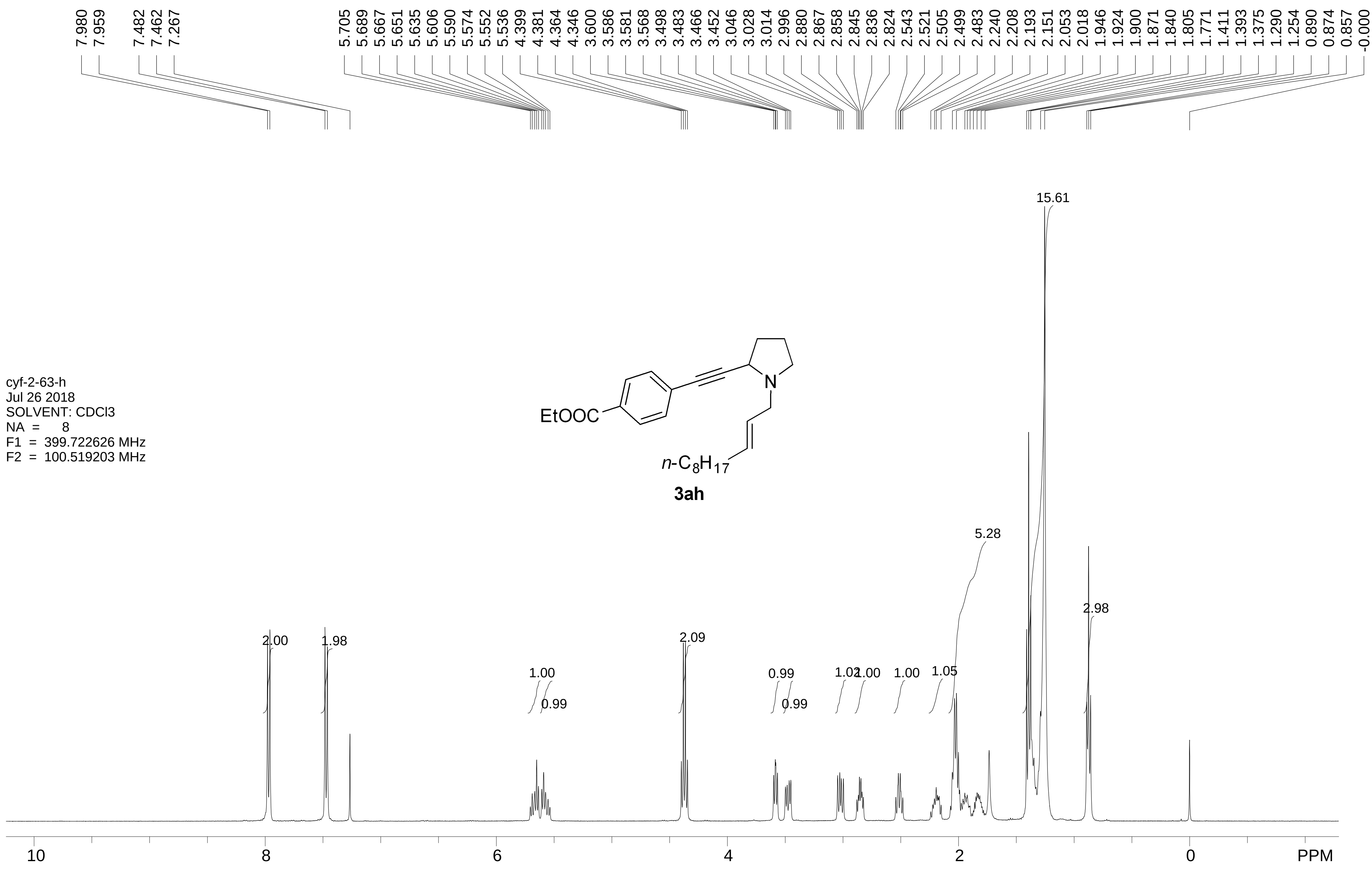
7.980
7.959
7.482
7.462
7.267
5.705
5.689
5.667
5.651
5.635
5.606
5.590
5.574
5.552
5.536
4.399
4.381
4.364
4.346
3.600
3.586
3.581
3.568
3.498
3.483
3.466
3.452
3.046
3.028
3.014
2.996
2.880
2.867
2.858
2.845
2.836
2.824
2.543
2.521
2.505
2.499
2.483
2.240
2.208
2.193
2.151
2.053
2.018
1.946
1.924
1.900
1.871
1.840
1.805
1.771
1.411
1.393
1.375
1.290
1.254
0.890
0.874
0.857
-0.000
cyf-2-63-h
Jul 26 2018
SOLVENT: CDCl3
NA = 8
F1 = 399.722626 MHz
F2 = 100.519203 MHz
EtOOC
N
n-C8H17
3ah
15.61
5.28
2.98
2.00
1.98
2.09
1.00
0.99
0.99
0.99
1.02
1.00
1.00
1.05
10
8
6
4
2
0
PPM

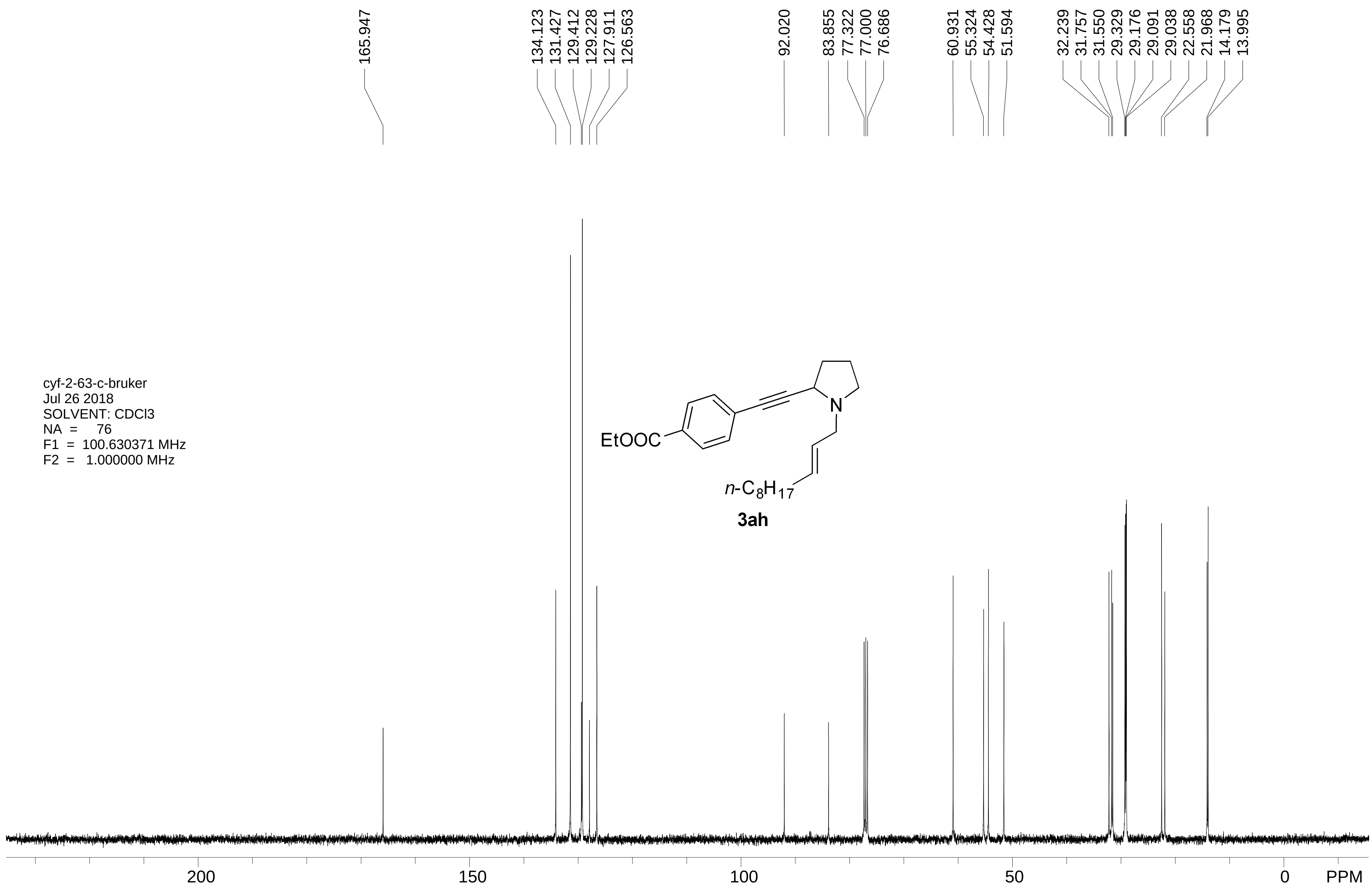
165.947
134.123
131.427
129.412
129.228
127.911
126.563
92.020
83.855
77.322
77.000
76.686
60.931
55.324
54.428
51.594
32.239
31.757
31.550
29.329
29.176
29.091
29.038
22.558
21.968
14.179
13.995
cyf-2-63-c-bruker
Jul 26 2018
SOLVENT: CDCl3
NA = 76
F1 = 100.630371 MHz
F2 = 1.000000 MHz
EtOOC
N
n-C8H17
3ah
200
150
100
50
0
PPM

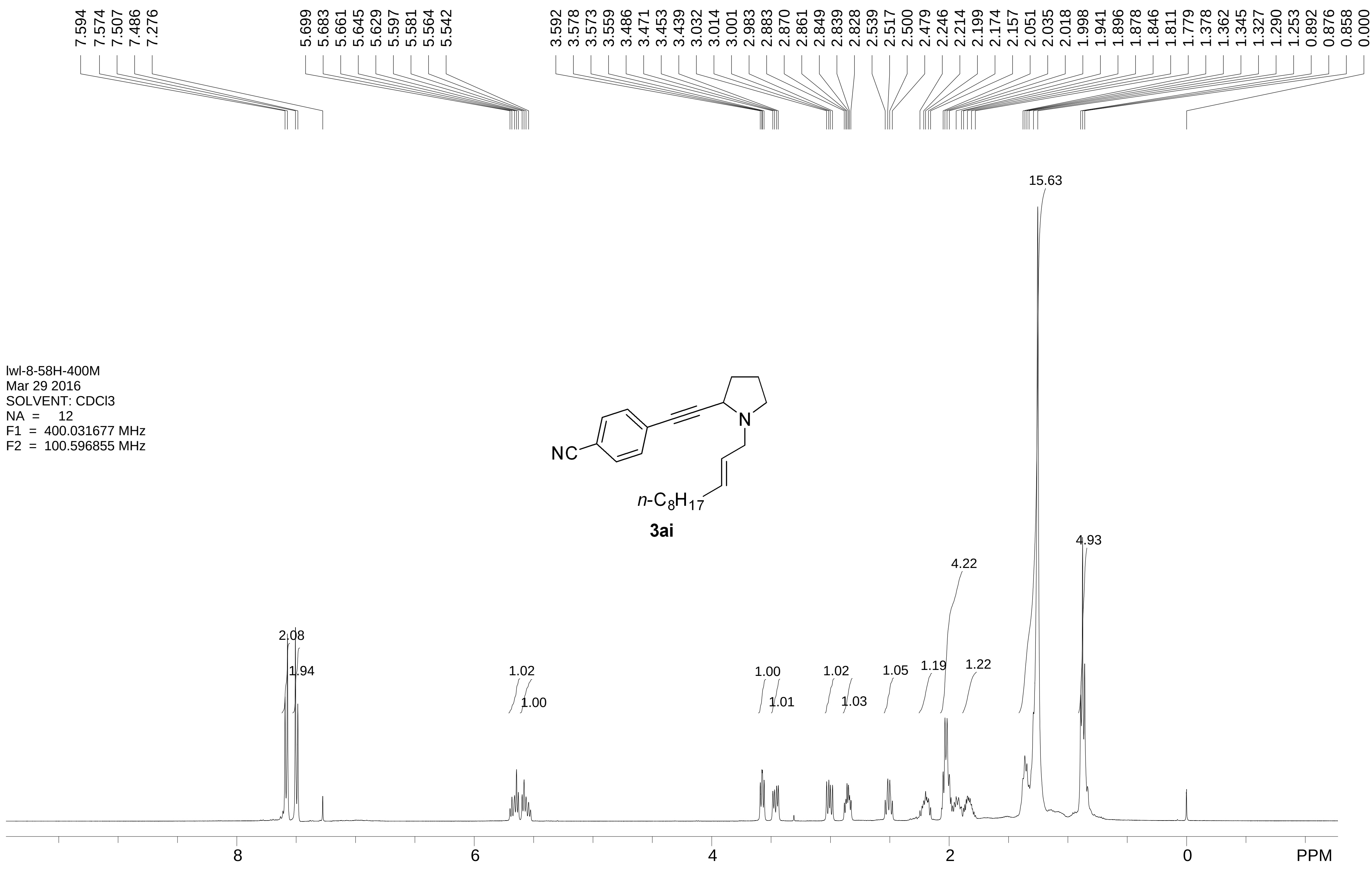
7.594 7.574 7.507 7.486 7.276
5.699 5.683 5.661 5.645 5.629 5.597 5.581 5.564 5.542
3.592 3.578 3.573 3.559 3.486 3.471 3.453 3.439 3.032 3.014 3.001 2.983 2.883 2.870 2.861 2.849 2.839 2.828 2.539 2.517 2.500 2.479 2.246 2.214 2.199 2.174 2.157 2.051 2.035 2.018 1.998 1.941 1.896 1.878 1.846 1.811 1.779 1.378 1.362 1.345 1.327 1.290 1.253 0.892 0.876 0.858 0.000
lwl-8-58H-400M
Mar 29 2016
SOLVENT: CDCl3
NA = 12
F1 = 400.031677 MHz
F2 = 100.596855 MHz
NC
N
n-C8H17
3ai
15.63
4.93
4.22
2.08
1.94
1.02
1.00
1.00
1.01
1.02
1.03
1.05
1.19
1.22
8
6
4
2
0
PPM

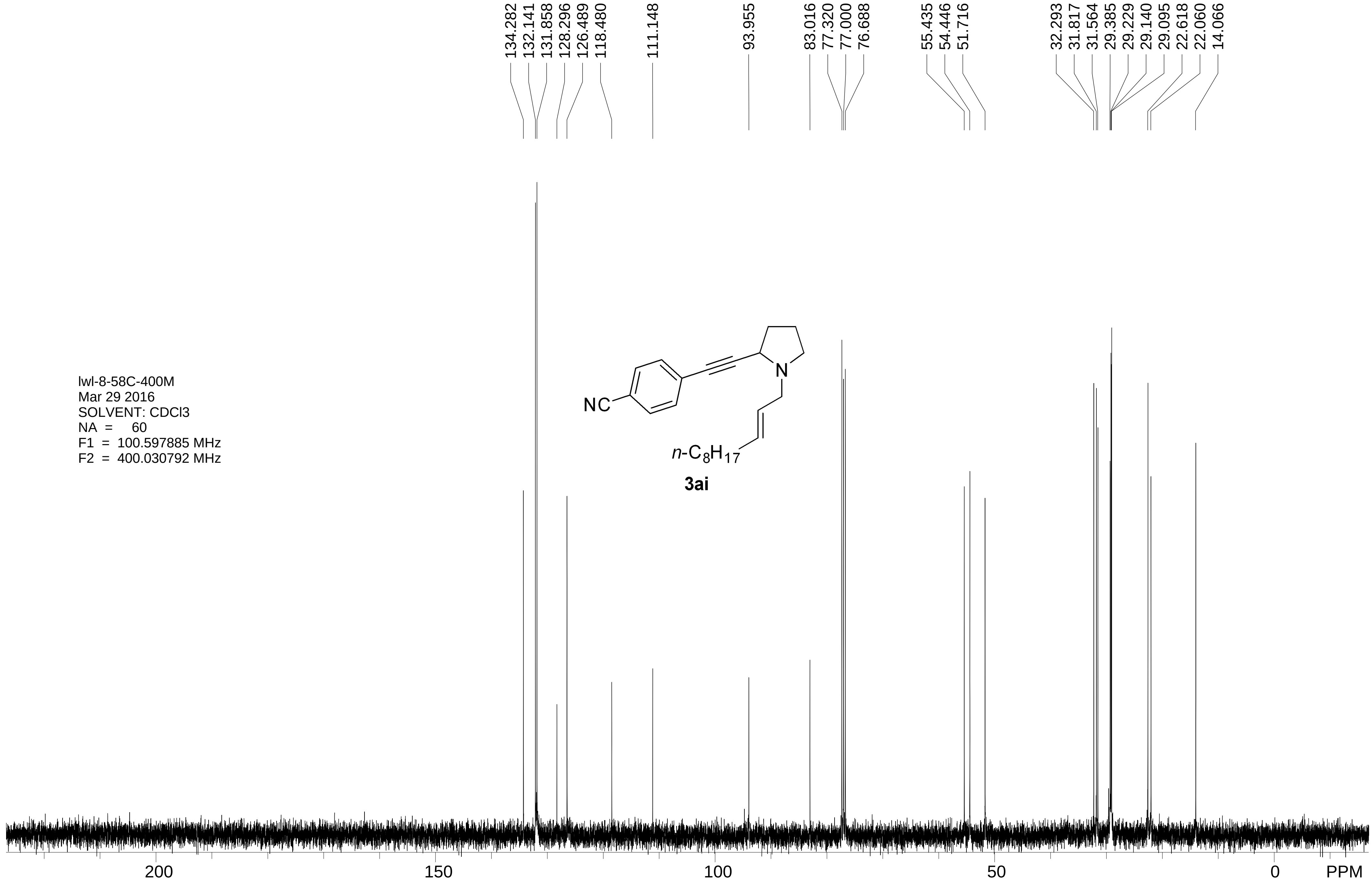
134.282
132.141
131.858
128.296
126.489
118.480
111.148
93.955
83.016
77.320
77.000
76.688
55.435
54.446
51.716
32.293
31.817
31.564
29.385
29.229
29.140
29.095
22.618
22.060
14.066
lwl-8-58C-400M
Mar 29 2016
SOLVENT: CDCl3
NA = 60
F1 = 100.597885 MHz
F2 = 400.030792 MHz
NC
N
n-C8H17
3ai
200
150
100
50
0
PPM

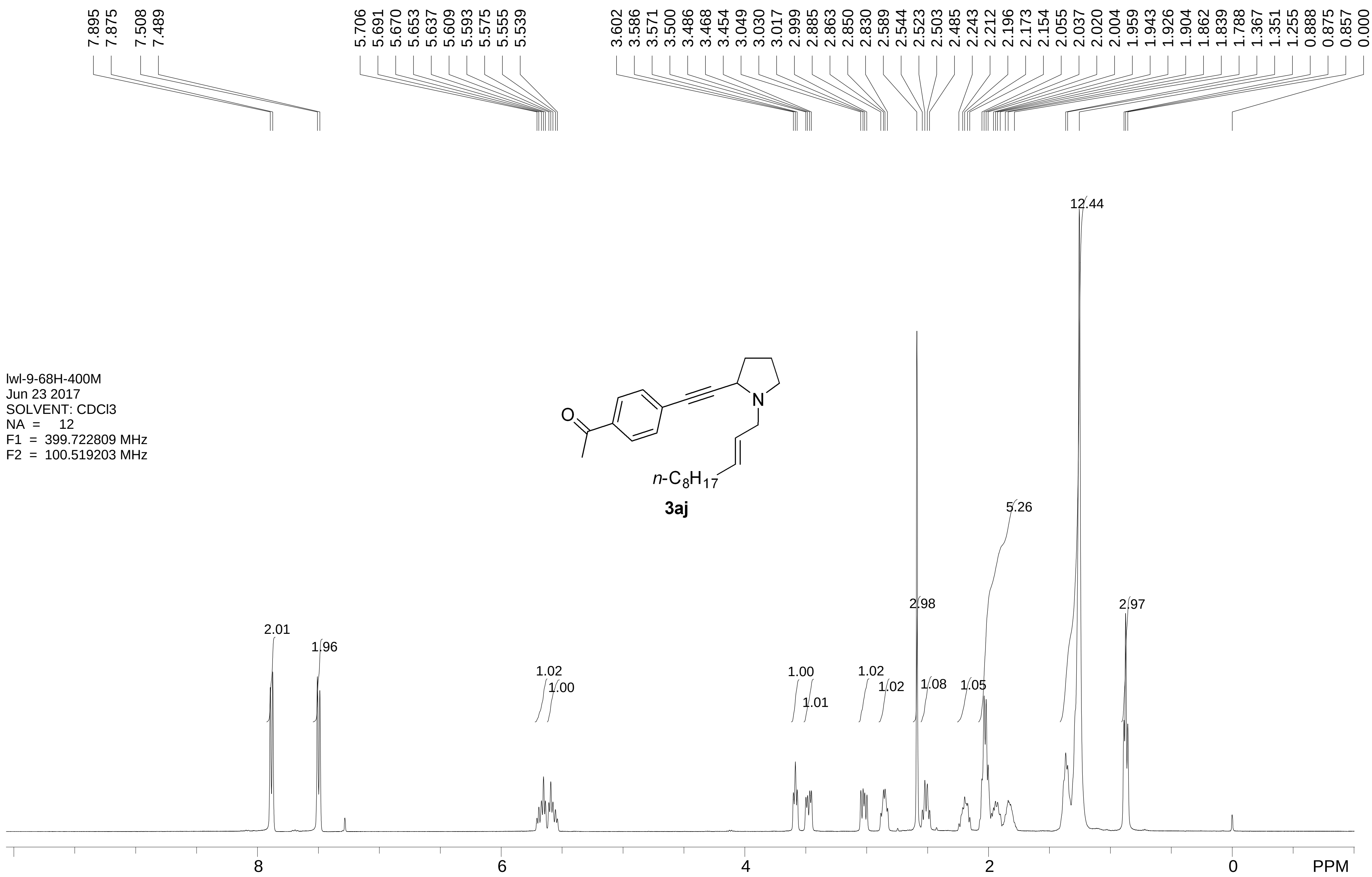
lwl-9-68H-400M
Jun 23 2017
SOLVENT: CDCl3
NA = 12
F1 = 399.722809 MHz
F2 = 100.519203 MHz
7.895 7.875 7.508 7.489 5.706 5.691 5.670 5.653 5.637 5.609 5.593 5.575 5.555 5.539 3.602 3.586 3.571 3.500 3.486 3.468 3.454 3.049 3.030 3.017 2.999 2.885 2.863 2.850 2.830 2.589 2.544 2.523 2.503 2.485 2.243 2.212 2.196 2.173 2.154 2.055 2.037 2.020 2.004 1.959 1.943 1.926 1.904 1.862 1.839 1.788 1.367 1.351 1.255 0.888 0.875 0.857 0.000
2.01 1.96 1.02 1.00 1.00 1.01 1.02 1.02 2.98 1.08 1.05 5.26 12.44 2.97
O
N
n-C8H17
3aj
8 6 4 2 0 PPM

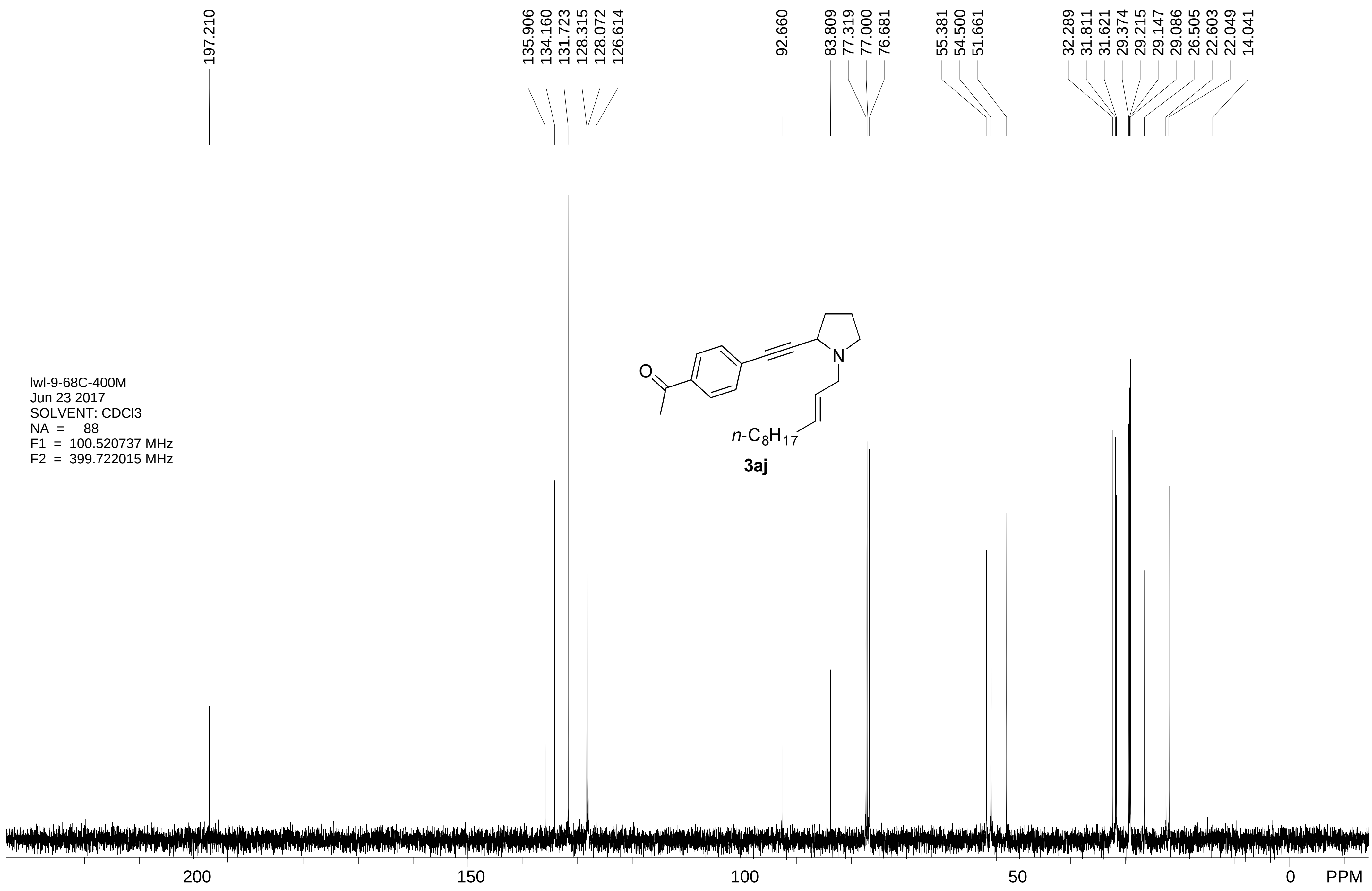
197.210
135.906
134.160
131.723
128.315
128.072
126.614
92.660
83.809
77.319
77.000
76.681
55.381
54.500
51.661
32.289
31.811
31.621
29.374
29.215
29.147
29.086
26.505
22.603
22.049
14.041
lwl-9-68C-400M
Jun 23 2017
SOLVENT: CDCl3
NA = 88
F1 = 100.520737 MHz
F2 = 399.722015 MHz
O
N
n-C8H17
3aj
200
150
100
50
0
PPM

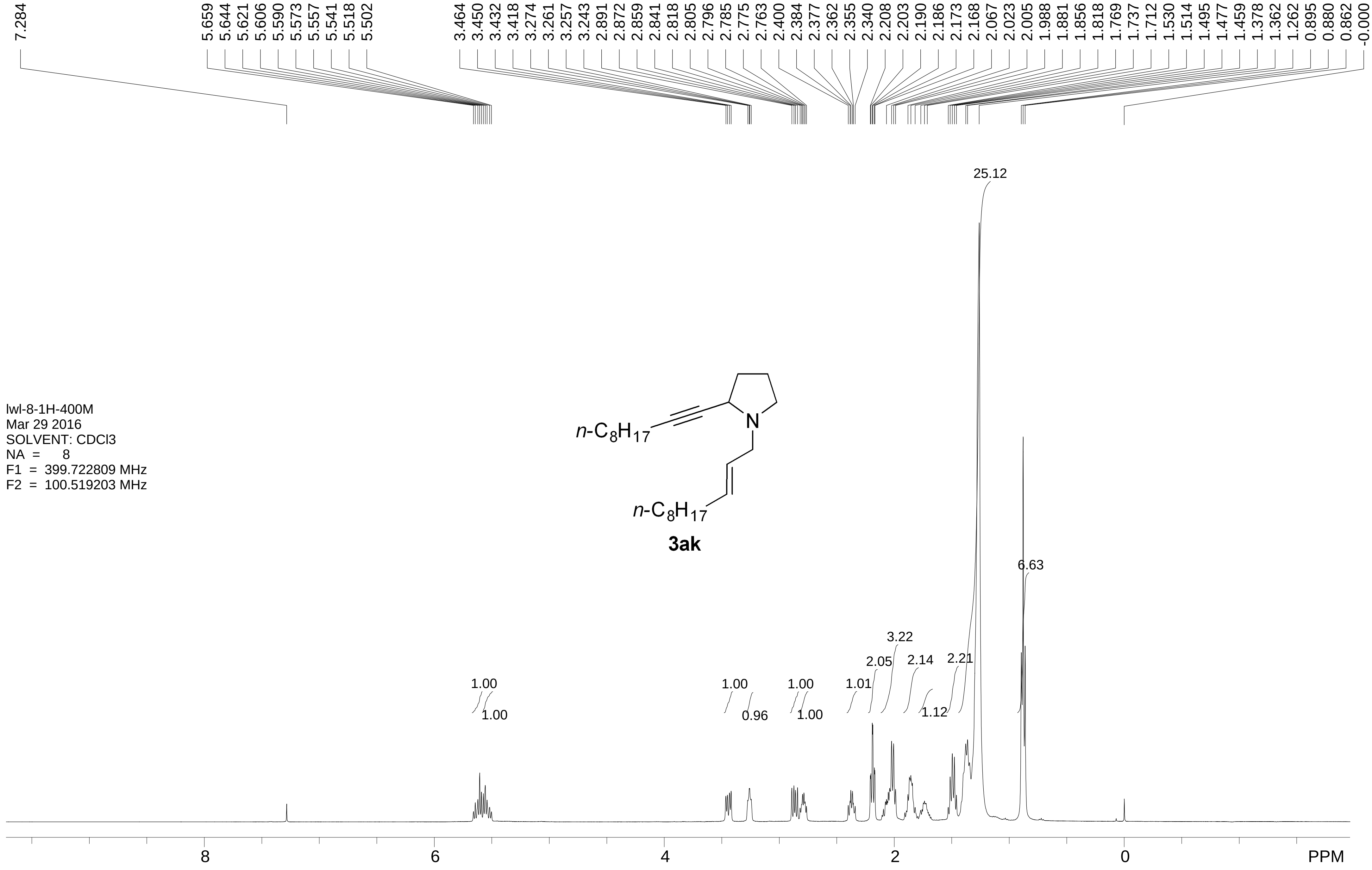
7.284
5.659 5.644 5.621 5.606 5.590 5.573 5.557 5.541 5.518 5.502
3.464 3.450 3.432 3.418 3.274 3.261 3.257 3.243 2.891 2.872 2.859 2.841 2.818 2.805 2.796 2.785 2.775 2.763 2.400 2.384 2.377 2.362 2.355 2.340 2.208 2.203 2.190 2.186 2.173 2.168 2.067 2.023 2.005 1.988 1.881 1.856 1.818 1.769 1.737 1.712 1.530 1.514 1.495 1.477 1.459 1.378 1.362 1.262 0.895 0.880 0.862 -0.000
25.12
6.63
3.22
2.05
2.14
2.21
1.00
1.00
1.00
0.96
1.00
1.00
1.01
1.12
lwl-8-1H-400M
Mar 29 2016
SOLVENT: CDCl3
NA = 8
F1 = 399.722809 MHz
F2 = 100.519203 MHz
n-C8H17
N
n-C8H17
3ak
8
6
4
2
0
PPM

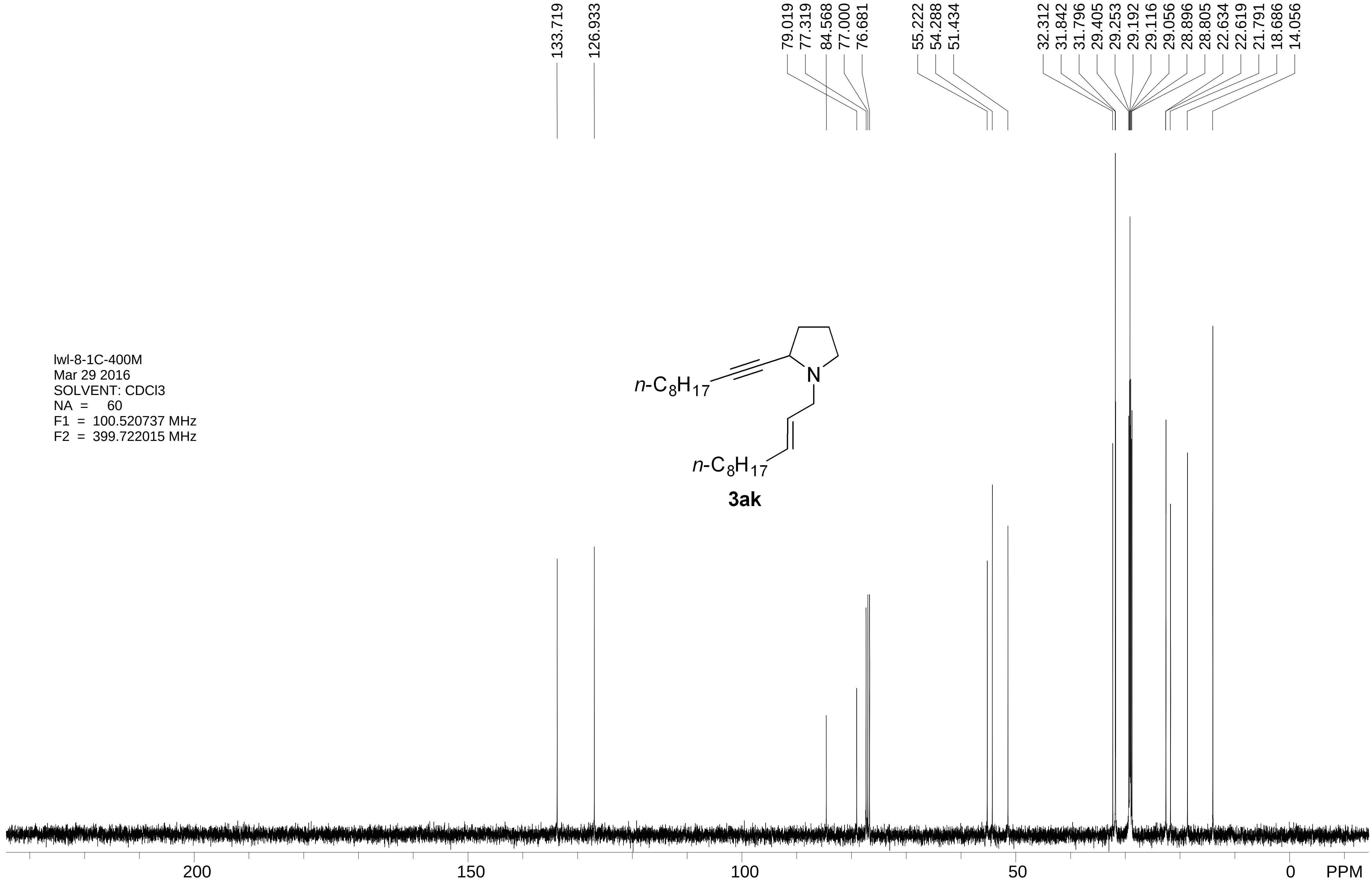
133.719
126.933
79.019
77.319
84.568
77.000
76.681
55.222
54.288
51.434
32.312
31.842
31.796
29.405
29.253
29.192
29.116
29.056
28.896
28.805
22.634
22.619
21.791
18.686
14.056
lwl-8-1C-400M
Mar 29 2016
SOLVENT: CDCl3
NA = 60
F1 = 100.520737 MHz
F2 = 399.722015 MHz
n-C8H17
N
n-C8H17
3ak
200
150
100
50
0
PPM

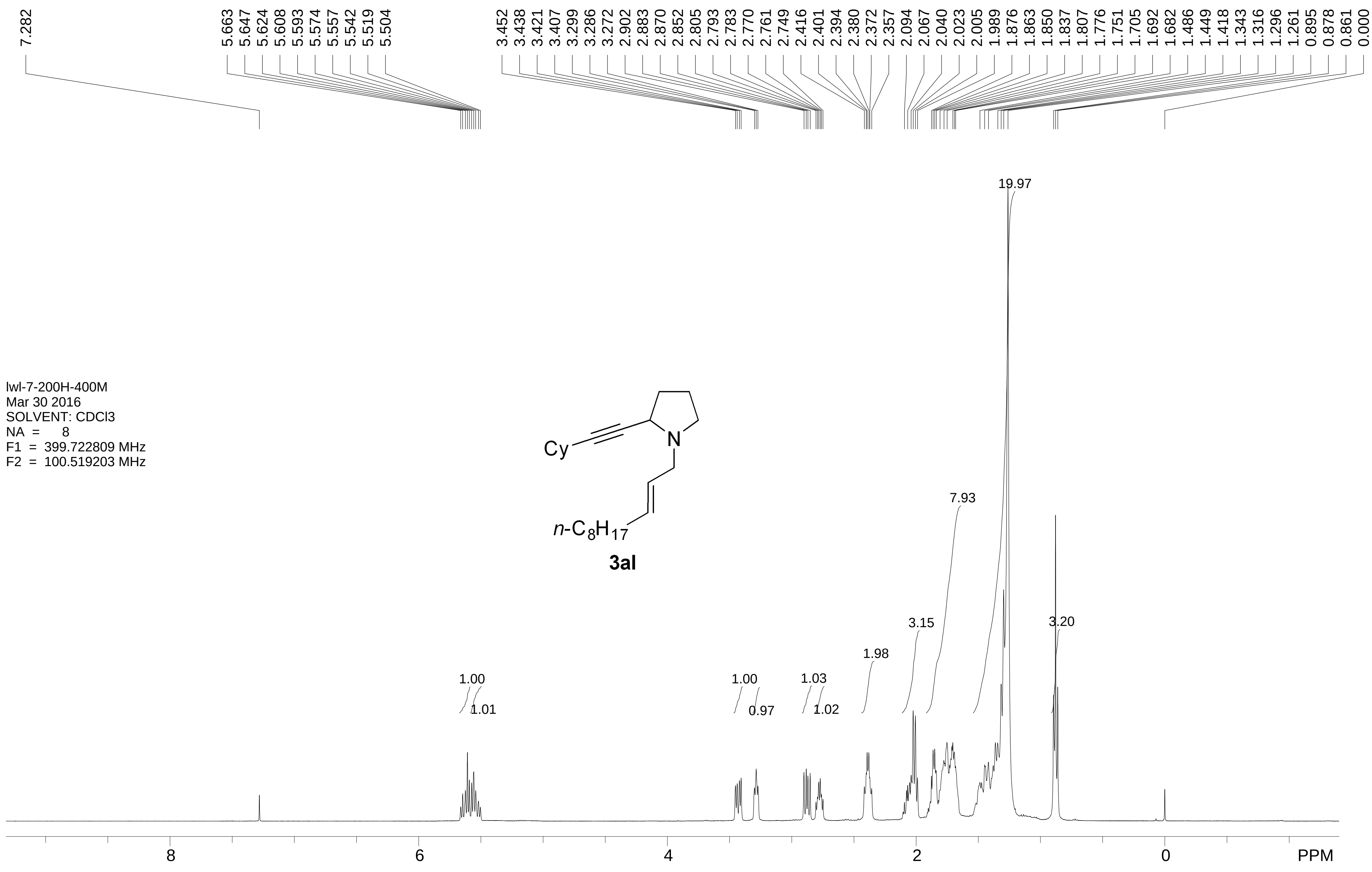
7.282
5.663 5.647 5.624 5.608 5.593 5.574 5.557 5.542 5.519 5.504
3.452 3.438 3.421 3.407 3.299 3.286 3.272 2.902 2.883 2.870 2.852 2.805 2.793 2.783 2.770 2.761 2.749 2.416 2.401 2.394 2.380 2.372 2.357 2.094 2.067 2.040 2.023 2.005 1.989 1.876 1.863 1.850 1.837 1.807 1.776 1.751 1.705 1.692 1.682 1.486 1.449 1.418 1.343 1.316 1.296 1.261 0.895 0.878 0.861 0.000
lwl-7-200H-400M
Mar 30 2016
SOLVENT: CDCl3
NA = 8
F1 = 399.722809 MHz
F2 = 100.519203 MHz
Cy
N
n-C8H17
3al
1.00 1.01 1.00 0.97 1.03 1.02 1.98 3.15 7.93 19.97 3.20
8 6 4 2 0 PPM

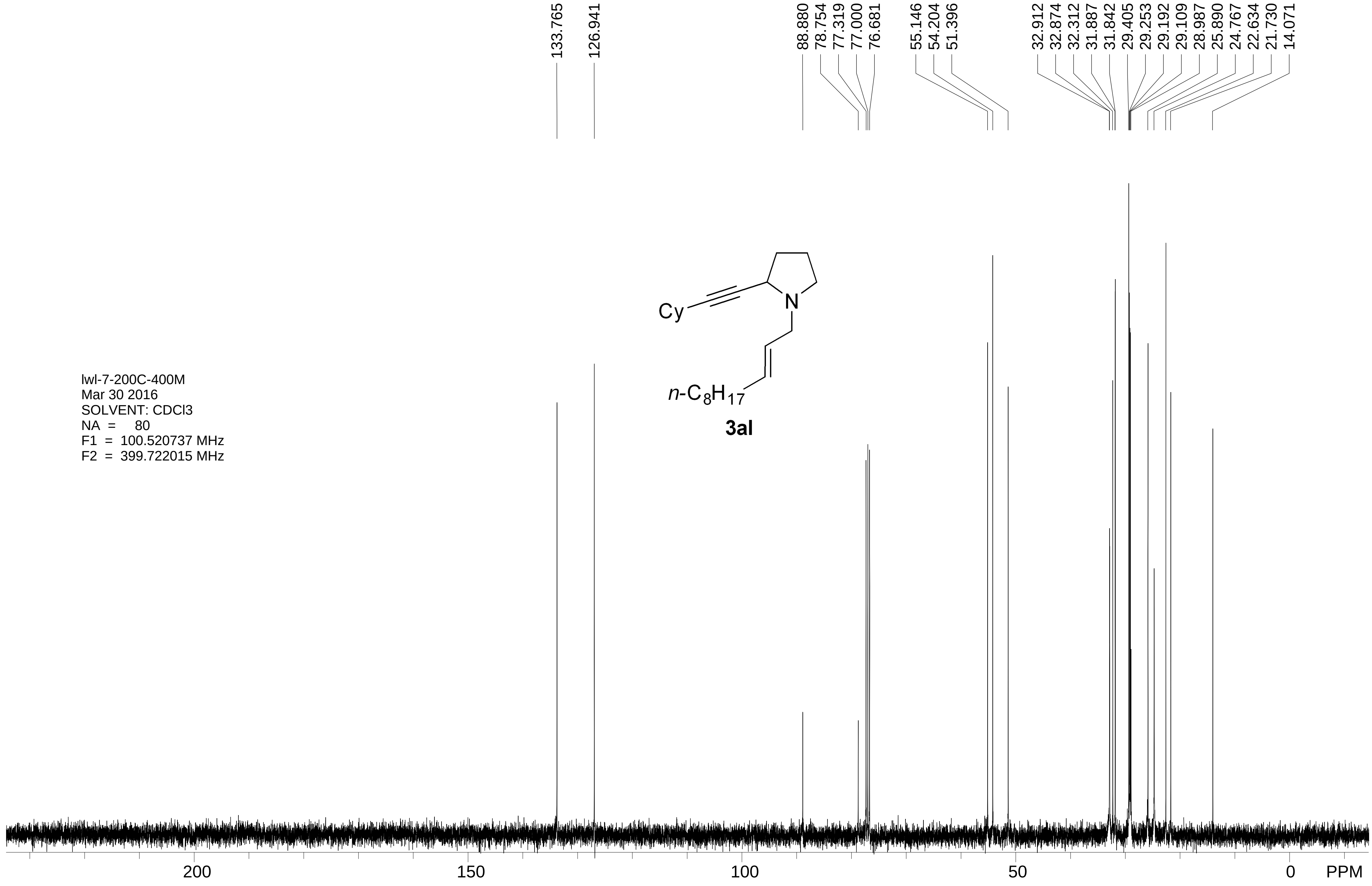

133.765
126.941
88.880
78.754
77.319
77.000
76.681
55.146
54.204
51.396
32.912
32.874
32.312
31.887
31.842
29.405
29.253
29.192
29.109
28.987
25.890
24.767
22.634
21.730
14.071
Cy
N
n-C8H17
3al
lwl-7-200C-400M
Mar 30 2016
SOLVENT: CDCl3
NA = 80
F1 = 100.520737 MHz
F2 = 399.722015 MHz
200
150
100
50
0
PPM

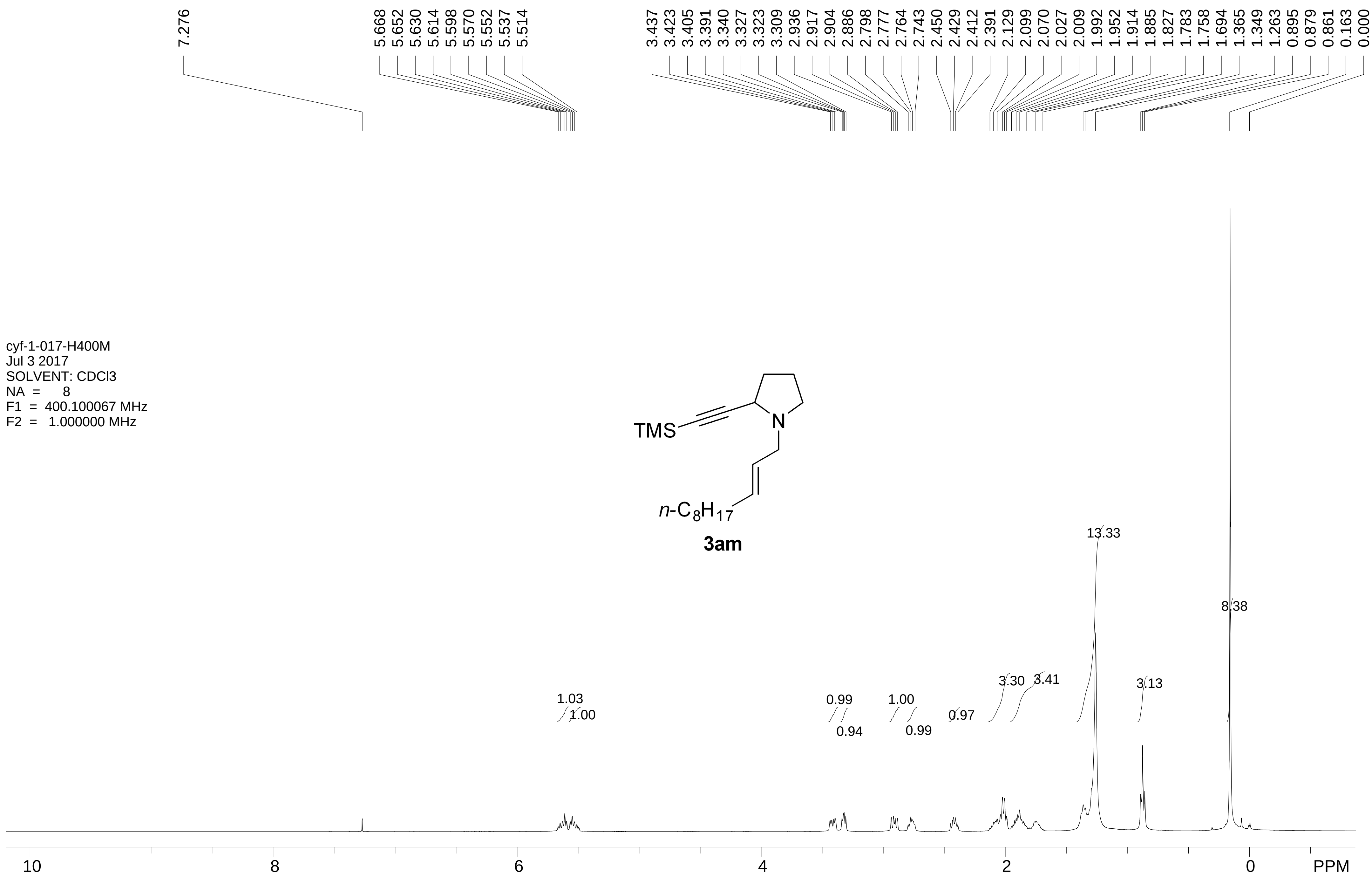

7.276
5.668 5.652 5.630 5.614 5.598 5.570 5.552 5.537 5.514
3.437 3.423 3.405 3.391 3.340 3.327 3.323 3.309 2.936 2.917 2.904 2.886 2.798 2.777 2.764 2.743 2.450 2.429 2.412 2.391 2.129 2.099 2.070 2.027 2.009 1.992 1.952 1.914 1.885 1.827 1.783 1.758 1.694 1.365 1.349 1.263 0.895 0.879 0.861 0.163 0.000
cyf-1-017-H400M
Jul 3 2017
SOLVENT: CDCl3
NA = 8
F1 = 400.100067 MHz
F2 = 1.000000 MHz
TMS
N
n-C8H17
3am
13.33
8.38
3.30
3.41
3.13
1.03
1.00
0.99
0.94
1.00
0.99
0.97
10
8
6
4
2
0
PPM

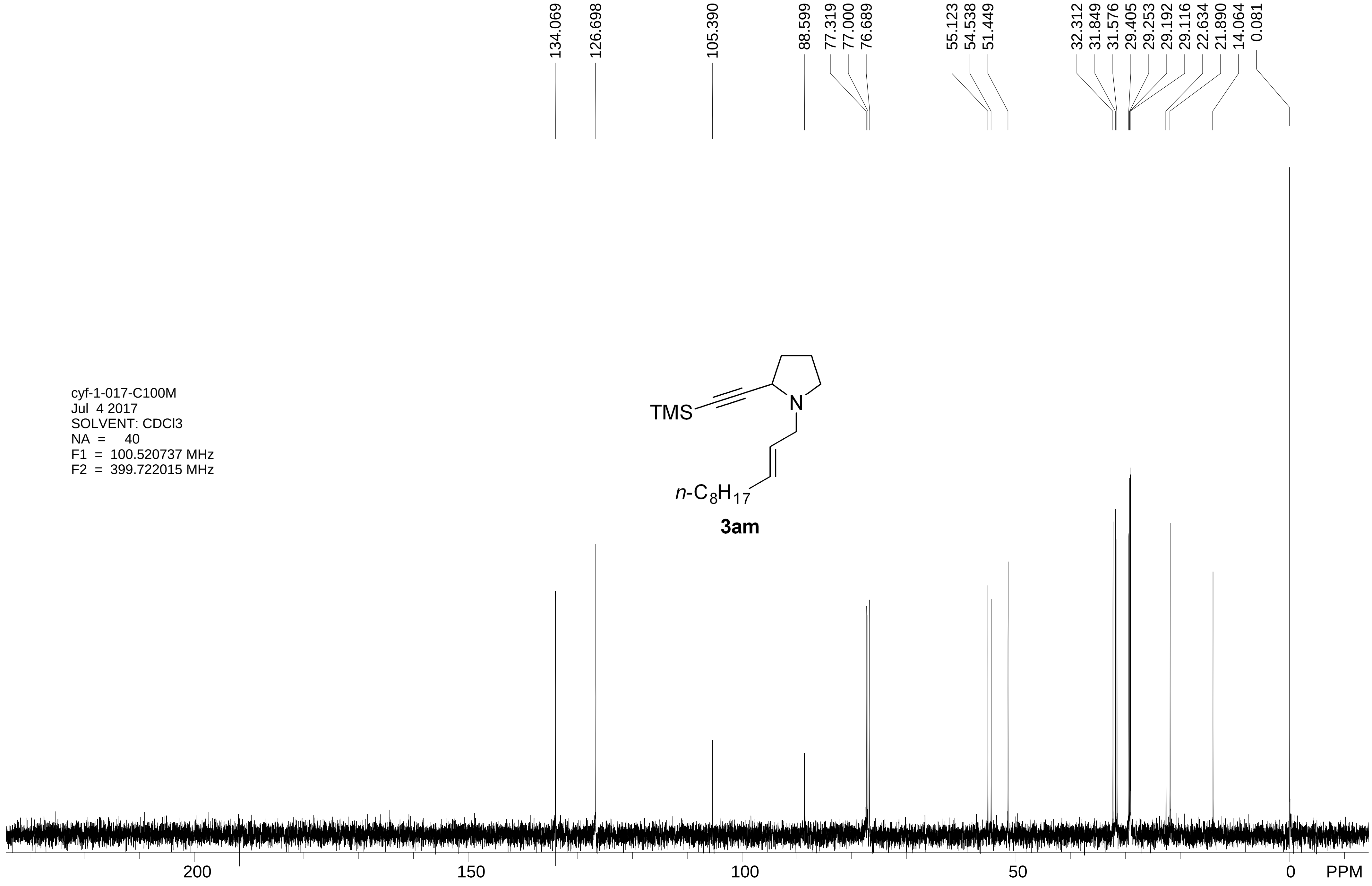
134.069
126.698
105.390
88.599
77.319
77.000
76.689
55.123
54.538
51.449
32.312
31.849
31.576
29.405
29.253
29.192
29.116
22.634
21.890
14.064
0.081
cyf-1-017-C100M
Jul 4 2017
SOLVENT: CDCl3
NA = 40
F1 = 100.520737 MHz
F2 = 399.722015 MHz
TMS
N
n-C8H17
3am
200
150
100
50
0
PPM

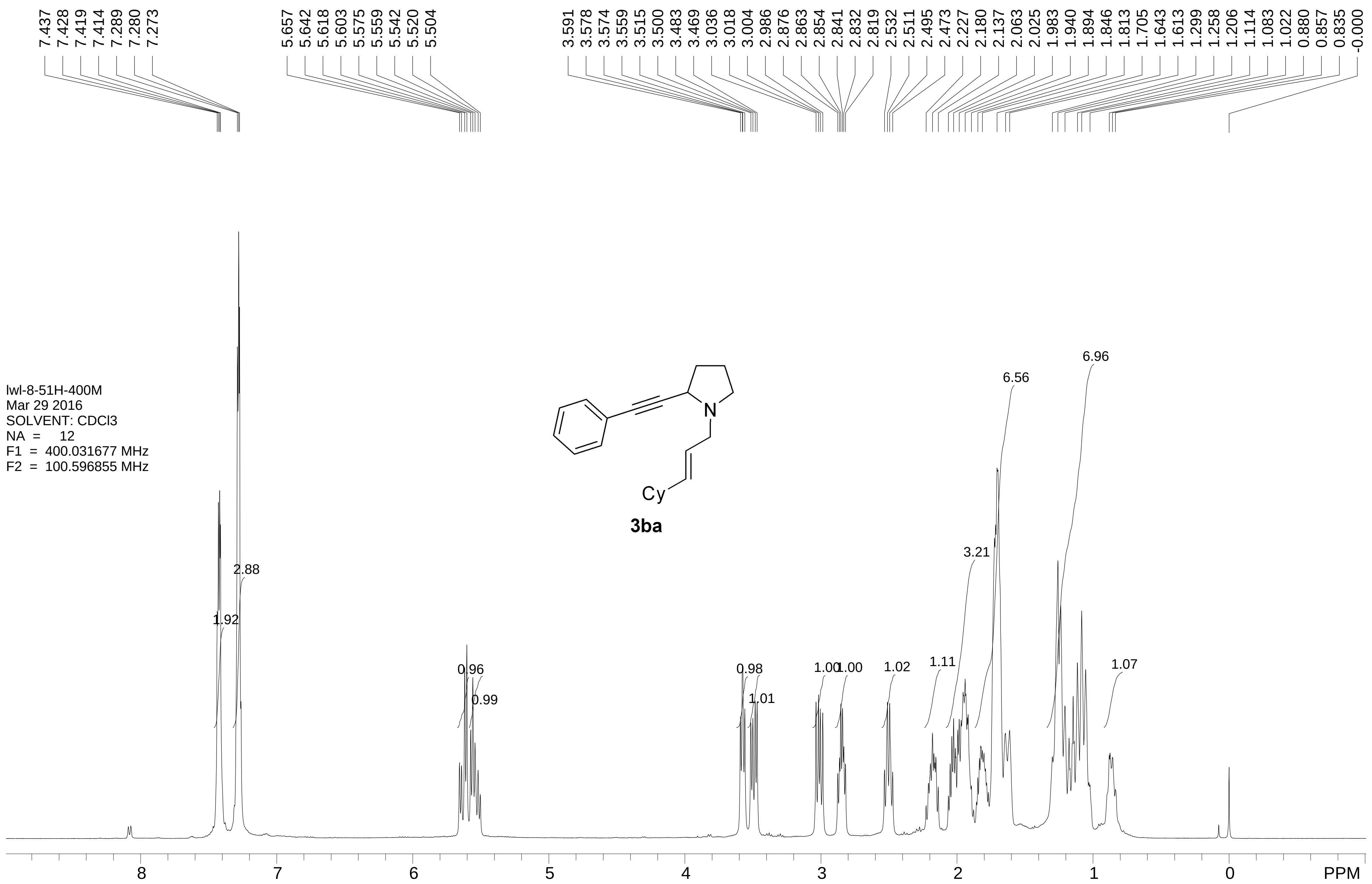
lwl-8-51H-400M
Mar 29 2016
SOLVENT: CDCl3
NA = 12
F1 = 400.031677 MHz
F2 = 100.596855 MHz
7.437 7.428 7.419 7.414 7.289 7.280 7.273
5.657 5.642 5.618 5.603 5.575 5.559 5.542 5.520 5.504
3.591 3.578 3.574 3.559 3.515 3.500 3.483 3.469 3.036 3.018 3.004 2.986 2.876 2.863 2.854 2.841 2.832 2.819 2.532 2.511 2.495 2.473 2.227 2.180 2.137 2.063 2.025 1.983 1.940 1.894 1.846 1.813 1.705 1.643 1.613 1.299 1.258 1.206 1.114 1.083 1.022 0.880 0.857 0.835 -0.000
N
Cy
3ba
1.92 2.88 0.96 0.99 0.98 1.01 1.00 1.00 1.02 1.11 3.21 6.56 6.96 1.07
8 7 6 5 4 3 2 1 0 PPM

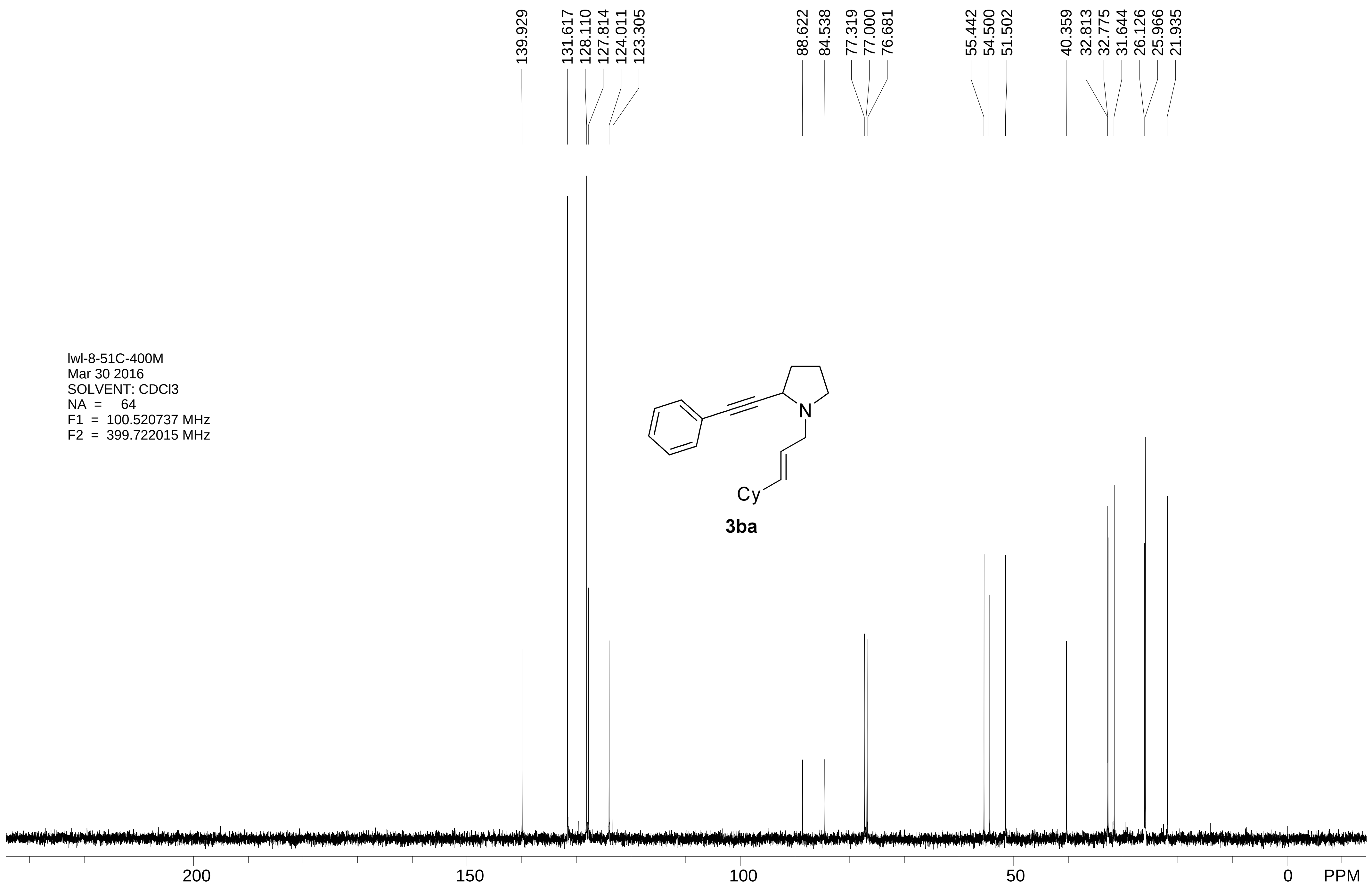
139.929
131.617
128.110
127.814
124.011
123.305
88.622
84.538
77.319
77.000
76.681
55.442
54.500
51.502
40.359
32.813
32.775
31.644
26.126
25.966
21.935
lwl-8-51C-400M
Mar 30 2016
SOLVENT: CDCl3
NA = 64
F1 = 100.520737 MHz
F2 = 399.722015 MHz
N
Cy
3ba
200
150
100
50
0
PPM

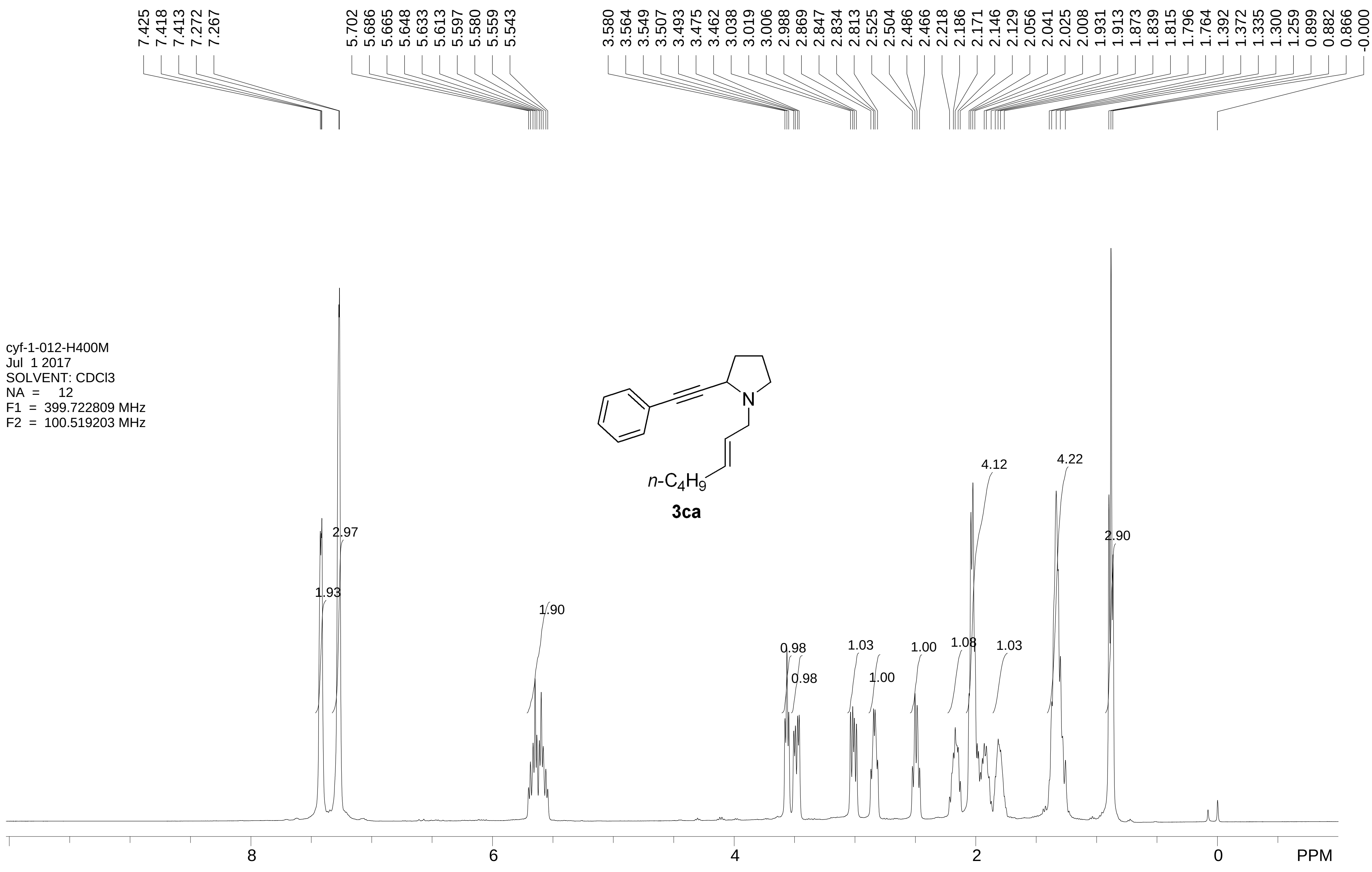
7.425
7.418
7.413
7.272
7.267
5.702
5.686
5.665
5.648
5.633
5.613
5.597
5.580
5.559
5.543
3.580
3.564
3.549
3.507
3.493
3.475
3.462
3.038
3.019
3.006
2.988
2.869
2.847
2.834
2.813
2.525
2.504
2.486
2.466
2.218
2.186
2.171
2.146
2.129
2.056
2.041
2.025
2.008
1.931
1.913
1.873
1.839
1.815
1.796
1.764
1.392
1.372
1.335
1.300
1.259
0.899
0.882
0.866
-0.000
cyf-1-012-H400M
Jul 1 2017
SOLVENT: CDCl3
NA = 12
F1 = 399.722809 MHz
F2 = 100.519203 MHz
N
n-C4H9
3ca
2.97
1.93
1.90
0.98
0.98
1.03
1.00
1.00
1.08
1.03
4.12
4.22
2.90
8
6
4
2
0
PPM

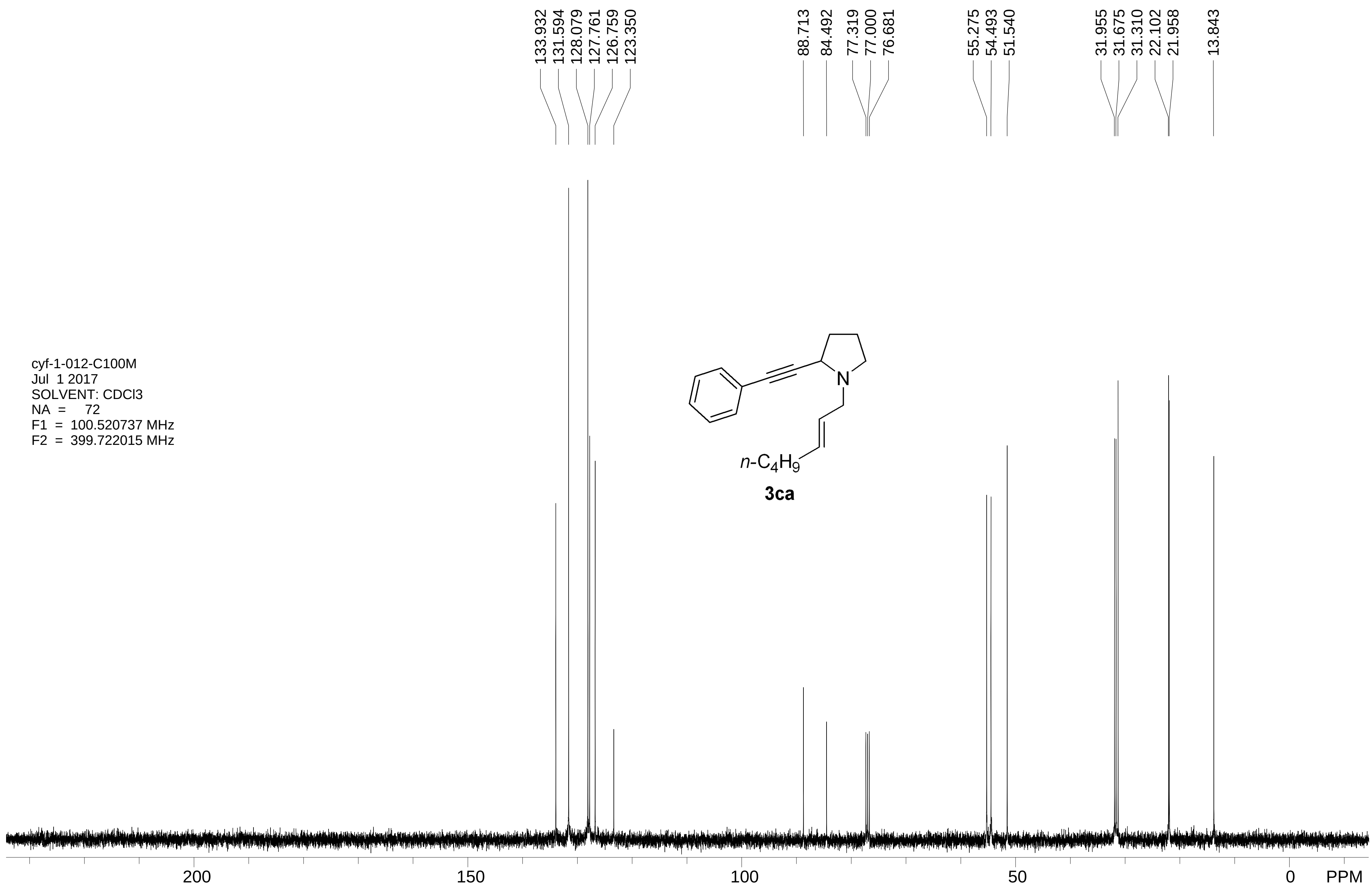
133.932
131.594
128.079
127.761
126.759
123.350
88.713
84.492
77.319
77.000
76.681
55.275
54.493
51.540
31.955
31.675
31.310
22.102
21.958
13.843
cyf-1-012-C100M
Jul 1 2017
SOLVENT: CDCl3
NA = 72
F1 = 100.520737 MHz
F2 = 399.722015 MHz
N
n-C4H9
3ca
200
150
100
50
0
PPM

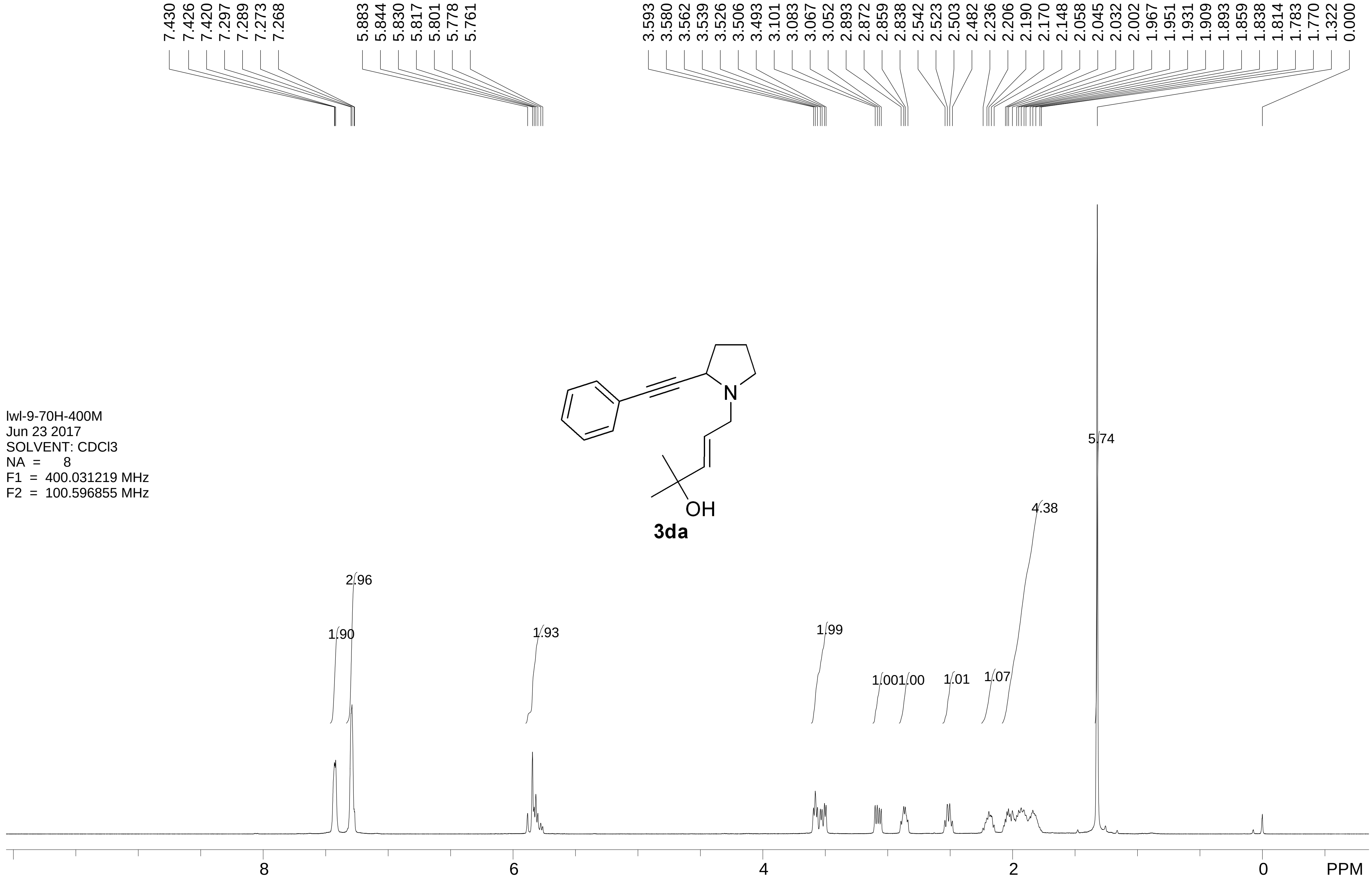
7.430
7.426
7.420
7.297
7.289
7.273
7.268
5.883
5.844
5.830
5.817
5.801
5.778
5.761
3.593
3.580
3.562
3.539
3.526
3.506
3.493
3.101
3.083
3.067
3.052
2.893
2.872
2.859
2.838
2.542
2.523
2.503
2.482
2.236
2.206
2.190
2.170
2.148
2.058
2.045
2.032
2.002
1.967
1.951
1.931
1.909
1.893
1.859
1.838
1.814
1.783
1.770
1.322
0.000
lwl-9-70H-400M
Jun 23 2017
SOLVENT: CDCl3
NA = 8
F1 = 400.031219 MHz
F2 = 100.596855 MHz
N
OH
3da
5.74
4.38
2.96
1.90
1.93
1.99
1.00
1.00
1.01
1.07
8
6
4
2
0
PPM

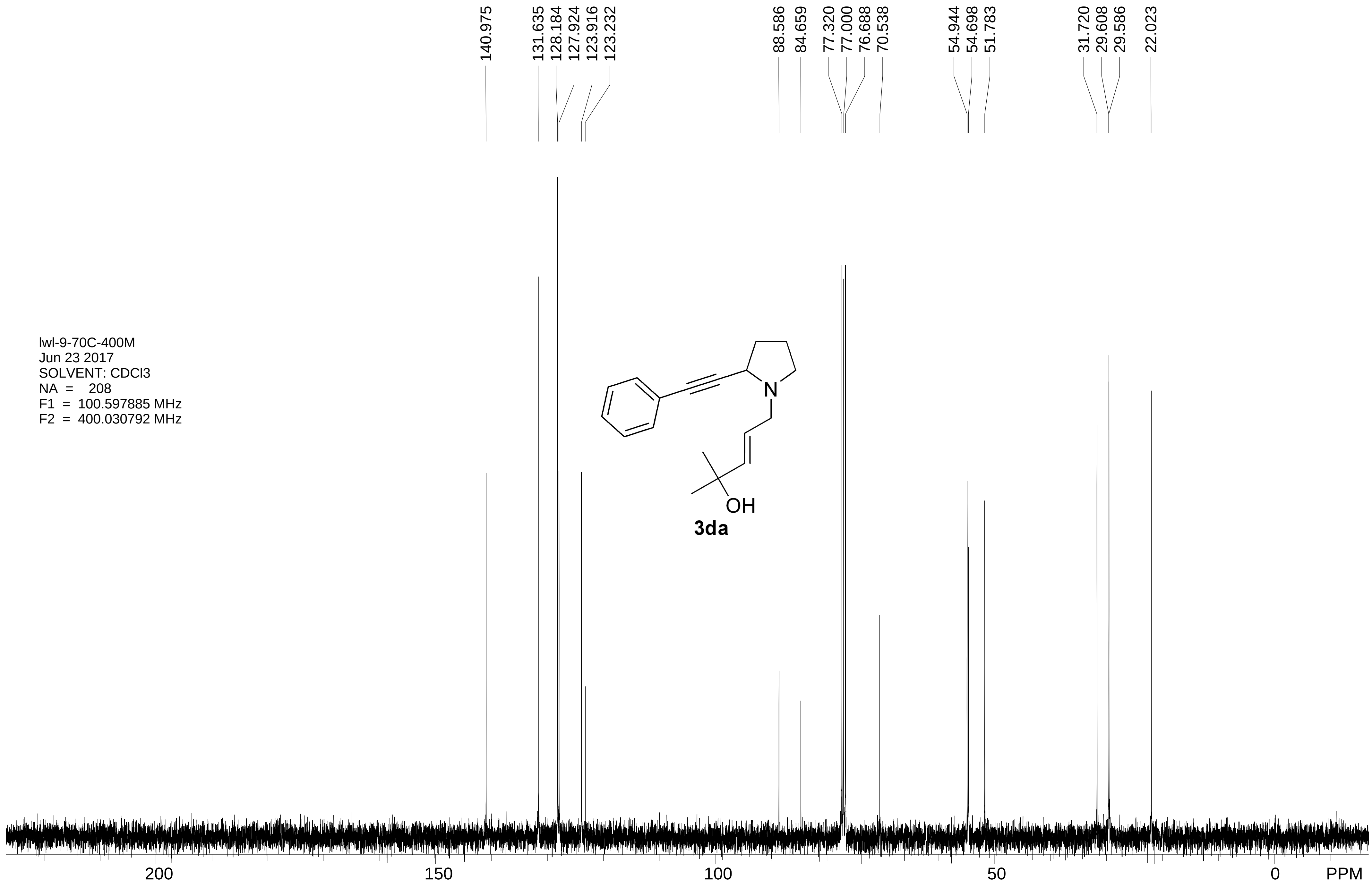
140.975
131.635
128.184
127.924
123.916
123.232
88.586
84.659
77.320
77.000
76.688
70.538
54.944
54.698
51.783
31.720
29.608
29.586
22.023
lwl-9-70C-400M
Jun 23 2017
SOLVENT: CDCl3
NA = 208
F1 = 100.597885 MHz
F2 = 400.030792 MHz
N
OH
3da
200
150
100
50
0
PPM

lwl-8-53H-400M
Mar 29 2016
SOLVENT: CDCl3
NA = 8
F1 = 399.722015 MHz
F2 = 100.519203 MHz

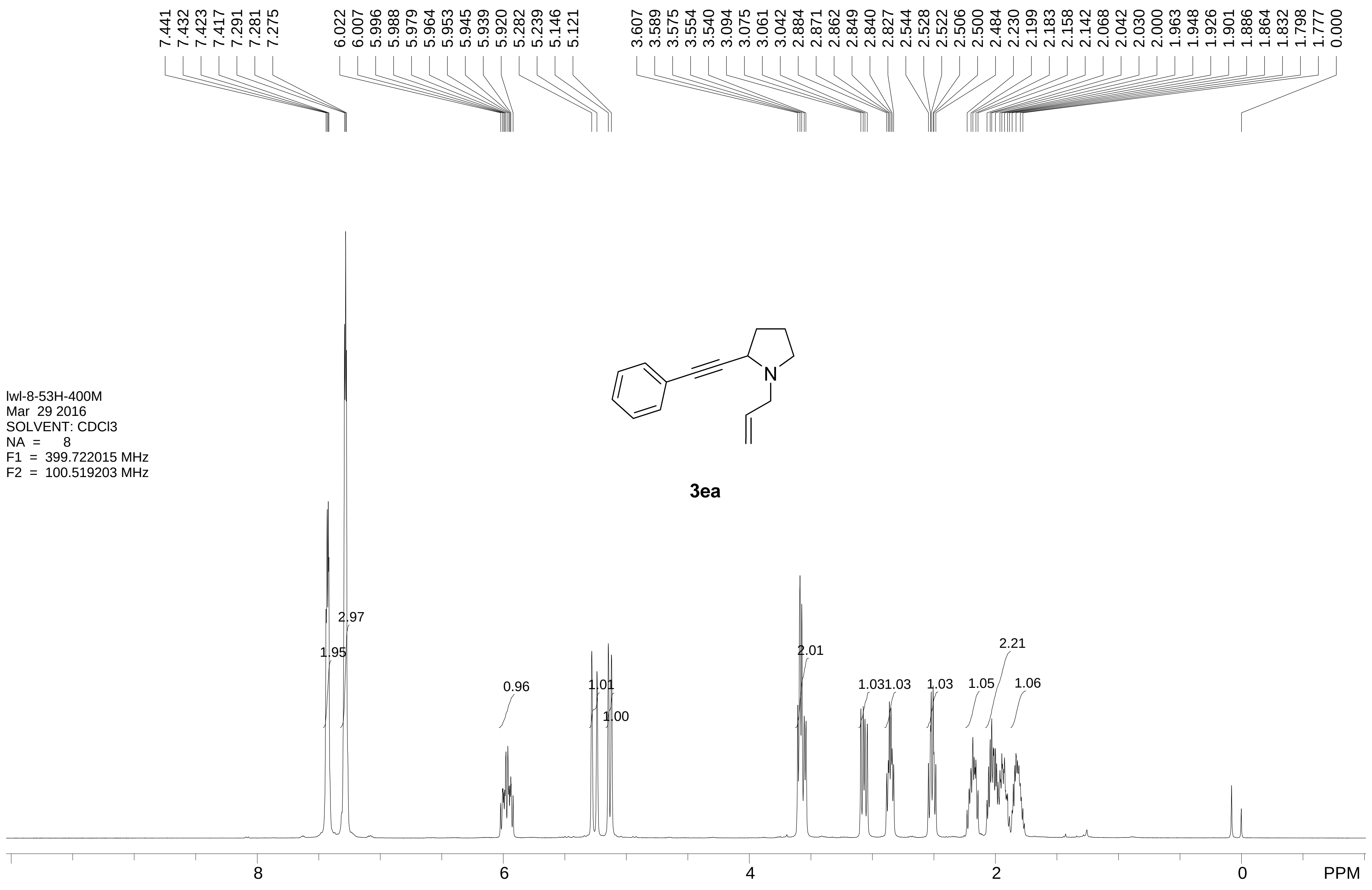

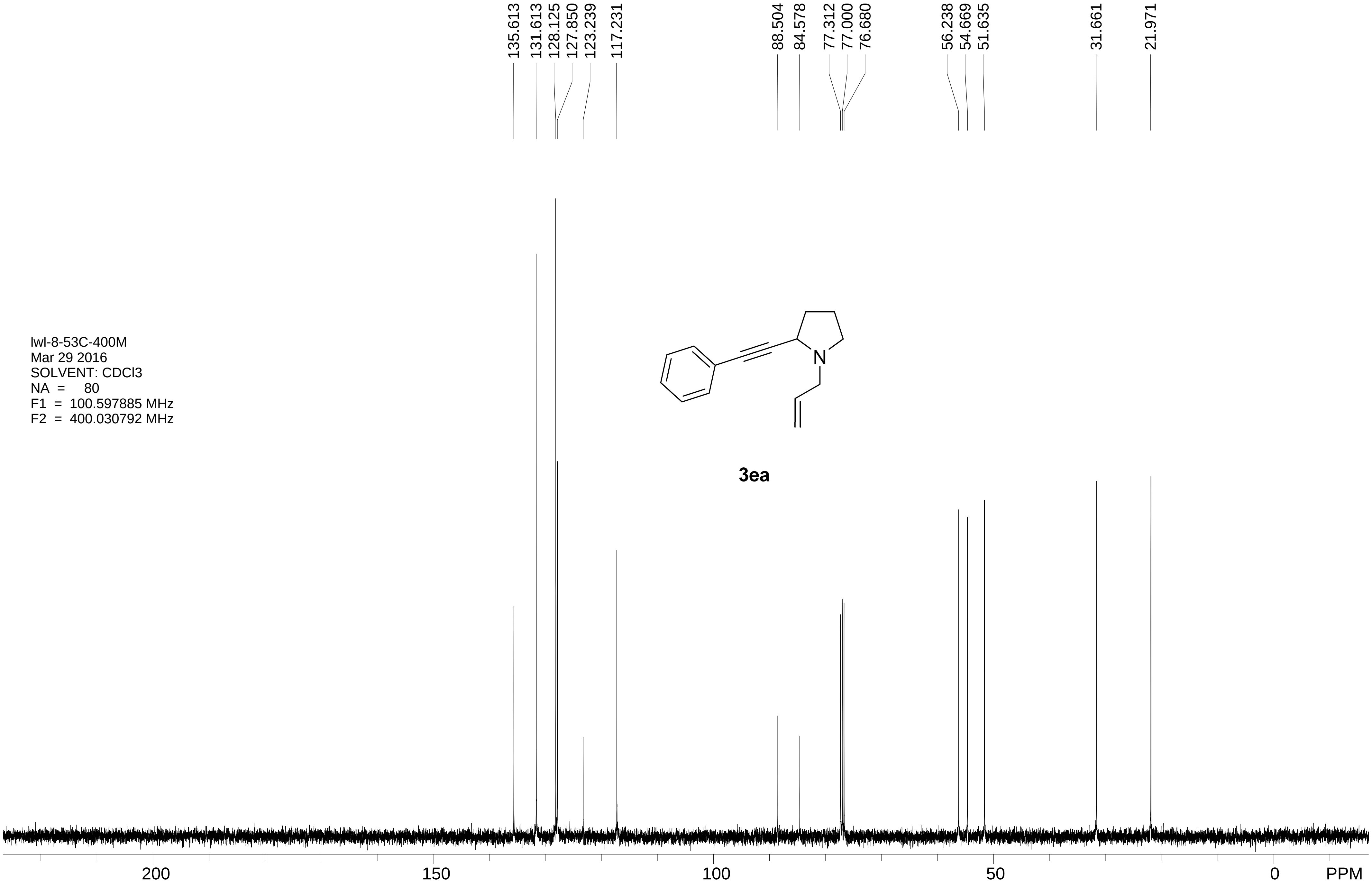
135.613
131.613
128.125
127.850
123.239
117.231
88.504
84.578
77.312
77.000
76.680
56.238
54.669
51.635
31.661
21.971
lwl-8-53C-400M
Mar 29 2016
SOLVENT: CDCl3
NA = 80
F1 = 100.597885 MHz
F2 = 400.030792 MHz
N
3ea
200
150
100
50
0
PPM

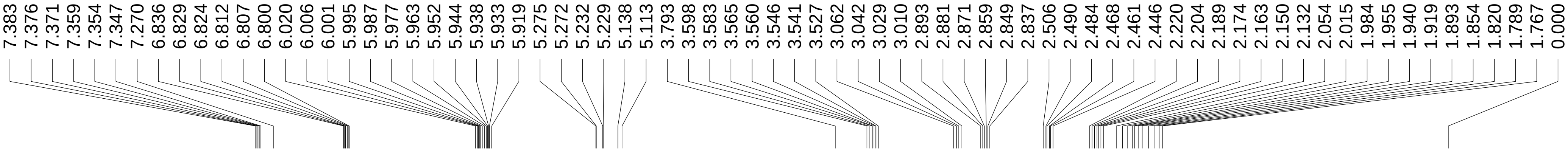

lwl-8-54H-400M
Mar 30 2016
SOLVENT: CDCl3
NA = 8
F1 = 399.722809 MHz
F2 = 100.519203 MHz

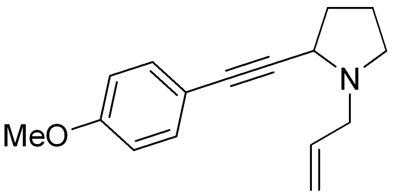

3ec

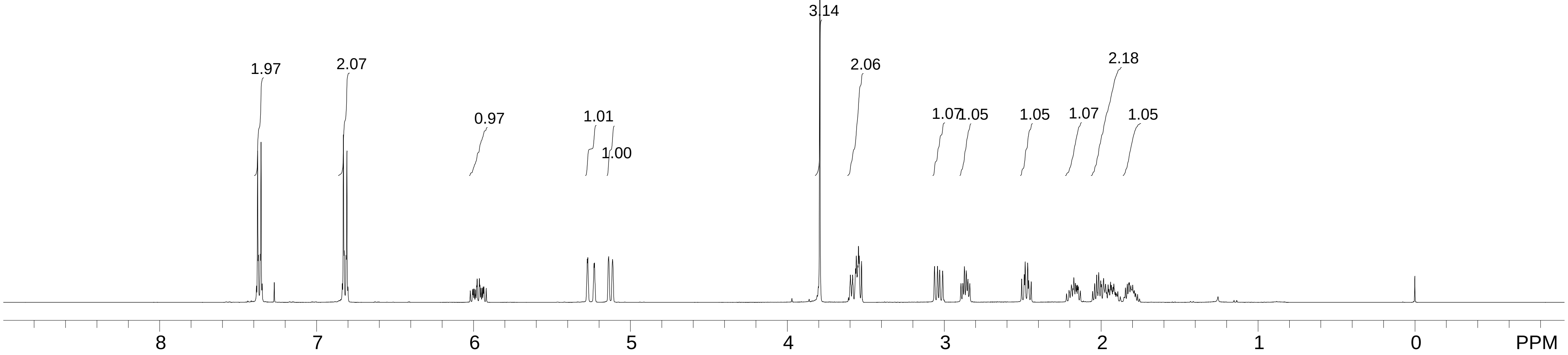

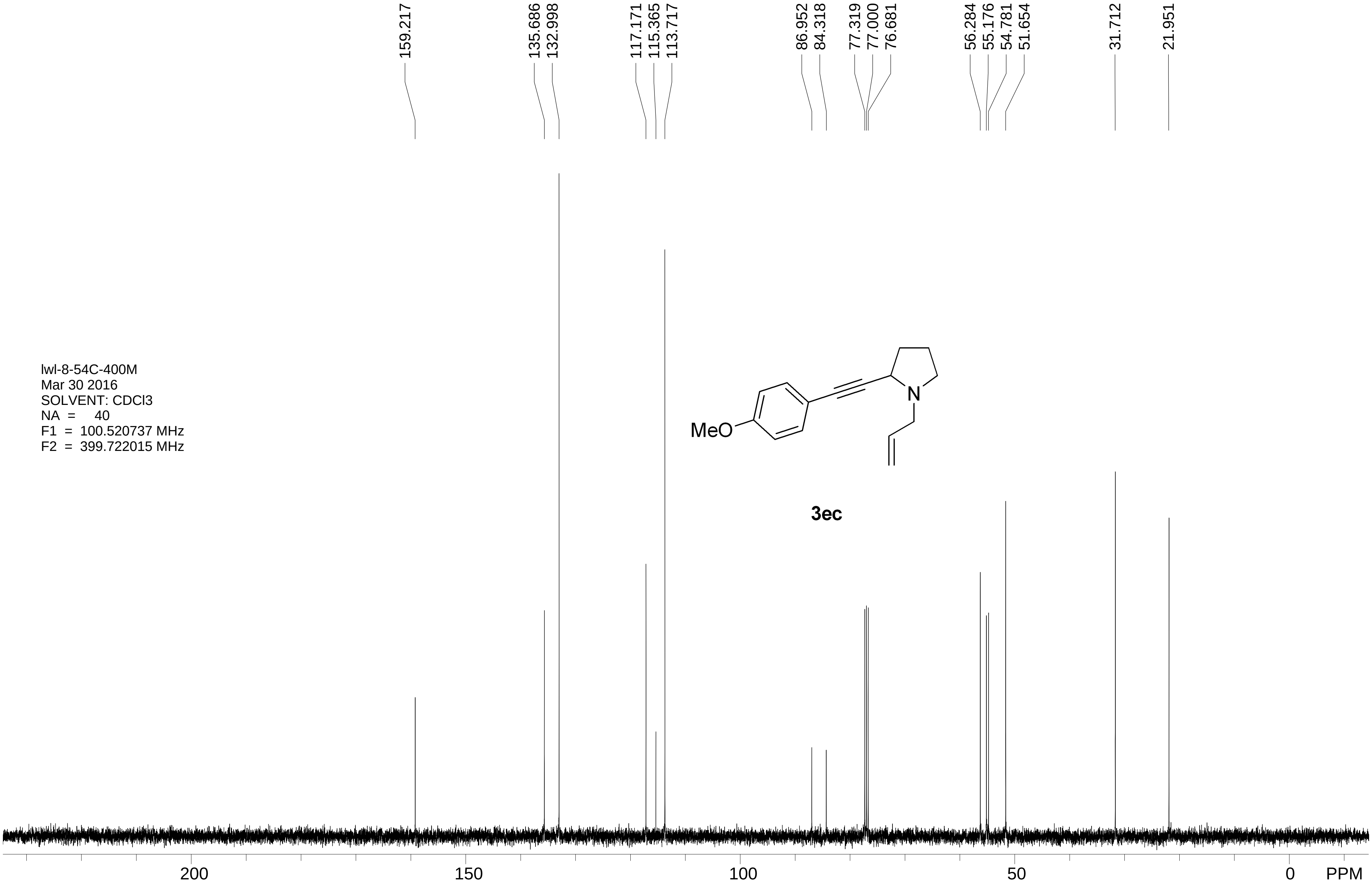
159.217
135.686
132.998
117.171
115.365
113.717
86.952
84.318
77.319
77.000
76.681
56.284
55.176
54.781
51.654
31.712
21.951
lwl-8-54C-400M
Mar 30 2016
SOLVENT: CDCl3
NA = 40
F1 = 100.520737 MHz
F2 = 399.722015 MHz
MeO
N
3ec
200
150
100
50
0
PPM

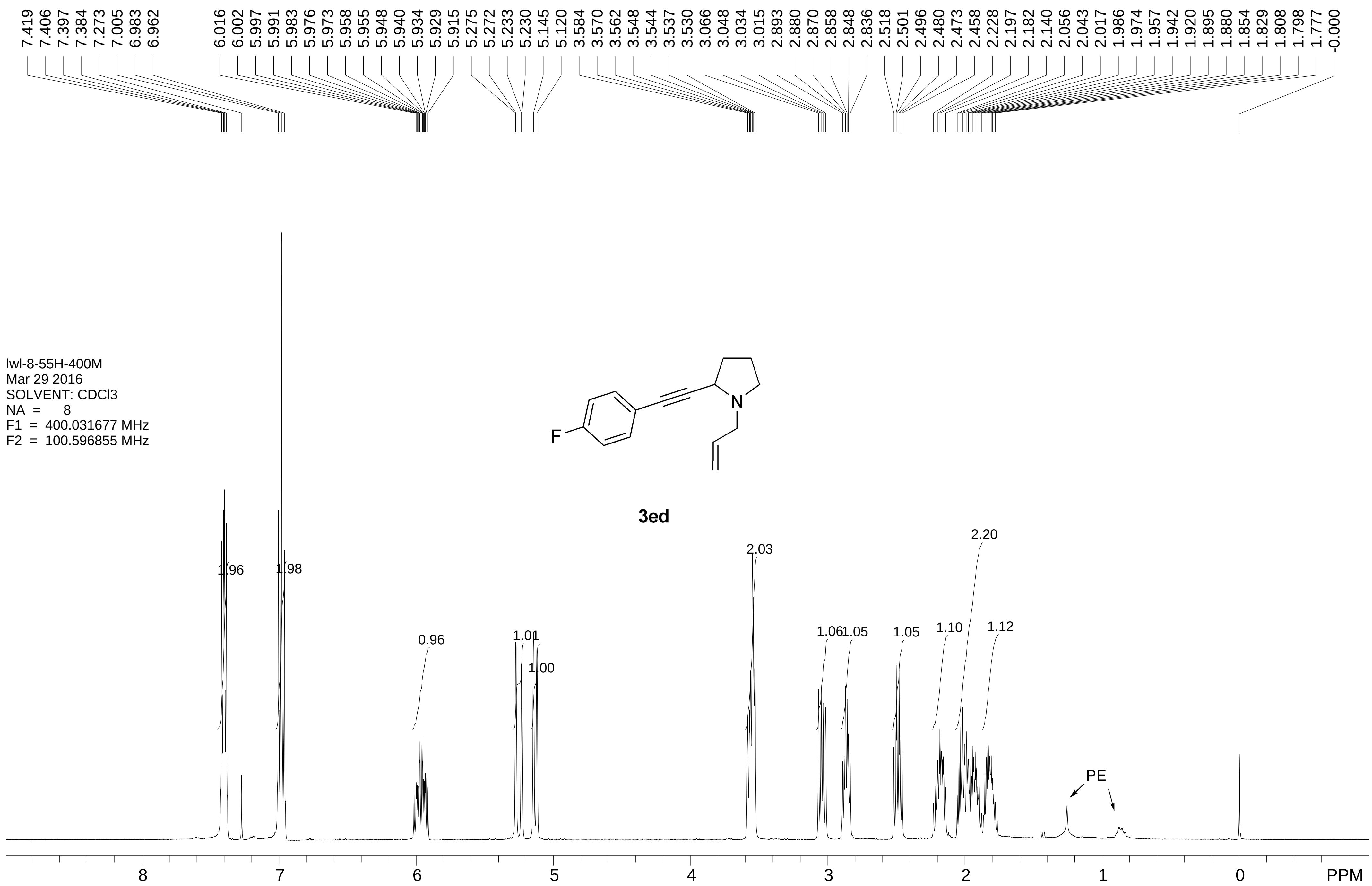
7.419
7.406
7.397
7.384
7.273
7.005
6.983
6.962
6.016
6.002
5.997
5.991
5.983
5.976
5.973
5.958
5.955
5.948
5.940
5.934
5.929
5.915
5.275
5.272
5.233
5.230
5.145
5.120
3.584
3.570
3.562
3.548
3.544
3.537
3.530
3.066
3.048
3.034
3.015
2.893
2.880
2.870
2.858
2.848
2.836
2.518
2.501
2.496
2.480
2.473
2.458
2.228
2.197
2.182
2.140
2.056
2.043
2.017
1.986
1.974
1.957
1.942
1.920
1.895
1.880
1.854
1.829
1.808
1.798
1.777
-0.000
lwl-8-55H-400M
Mar 29 2016
SOLVENT: CDCl3
NA = 8
F1 = 400.031677 MHz
F2 = 100.596855 MHz
F
N
3ed
1.96
1.98
0.96
1.01
1.00
2.03
1.06
1.05
1.05
1.10
2.20
1.12
PE
8
7
6
5
4
3
2
1
0
PPM

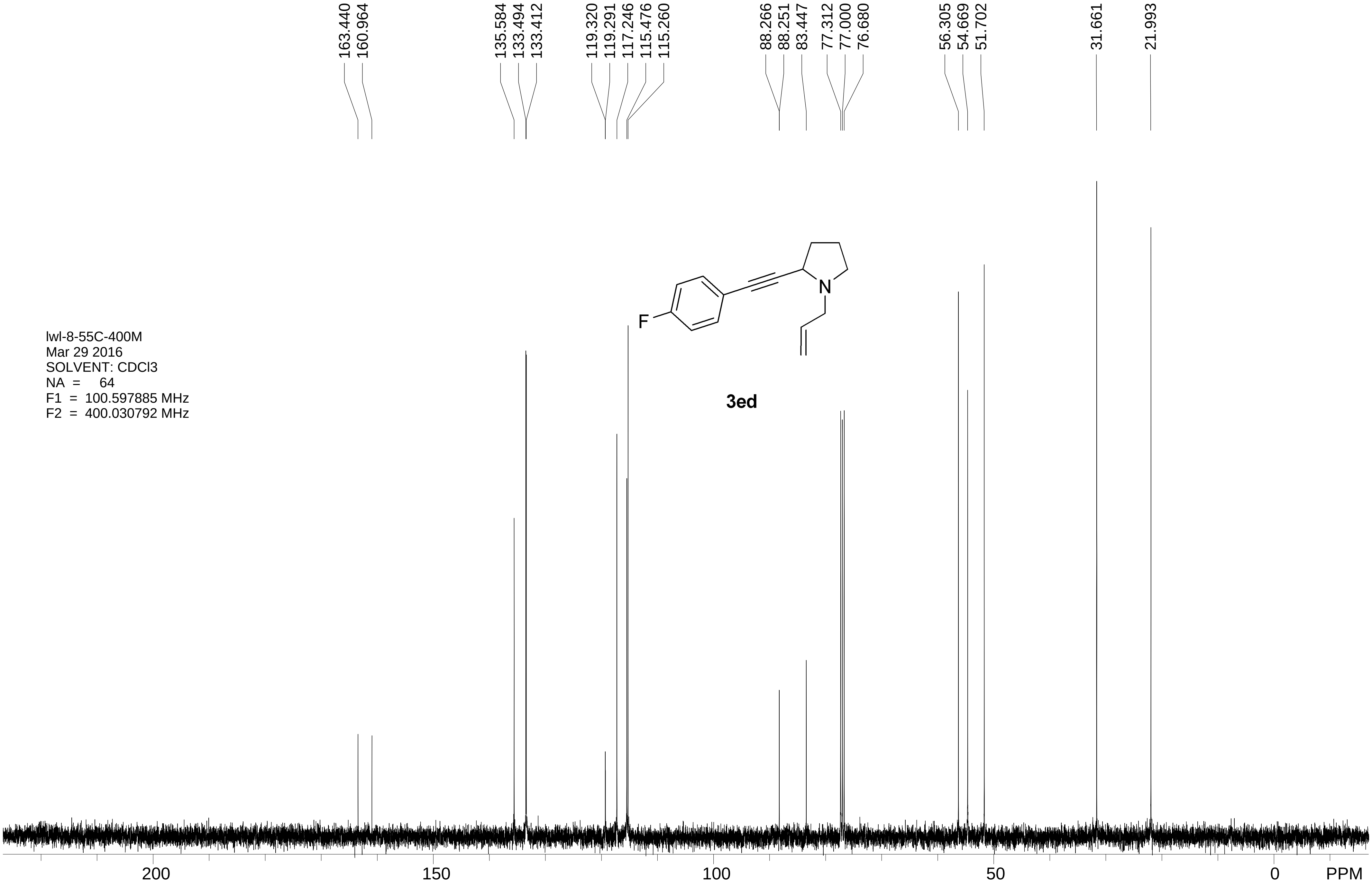
163.440
160.964
135.584
133.494
133.412
119.320
119.291
117.246
115.476
115.260
88.266
88.251
83.447
77.312
77.000
76.680
56.305
54.669
51.702
31.661
21.993
lwl-8-55C-400M
Mar 29 2016
SOLVENT: CDCl3
NA = 64
F1 = 100.597885 MHz
F2 = 400.030792 MHz
200
150
100
50
0
PPM
F
N

3ed

lwl-8-55F-400M
Apr 6 2016
SOLVENT: CDCl3
NA =   8
F1 = 376.076111 MHz
F2 = 100.519203 MHz

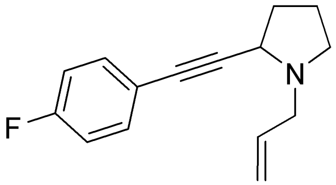

3ed

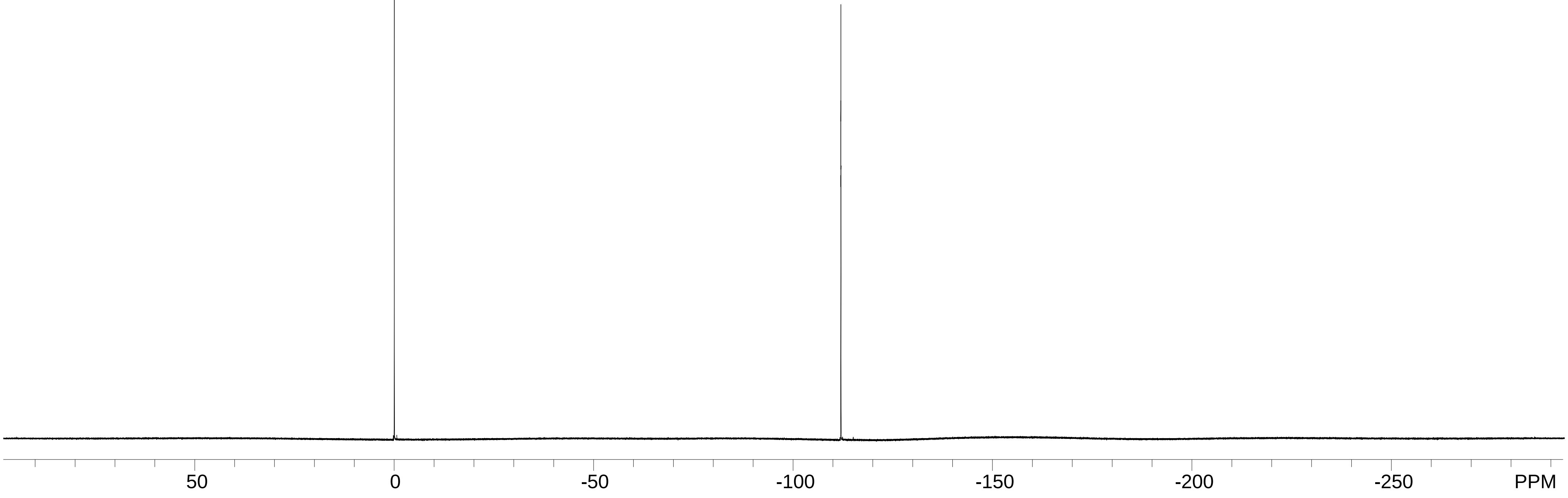

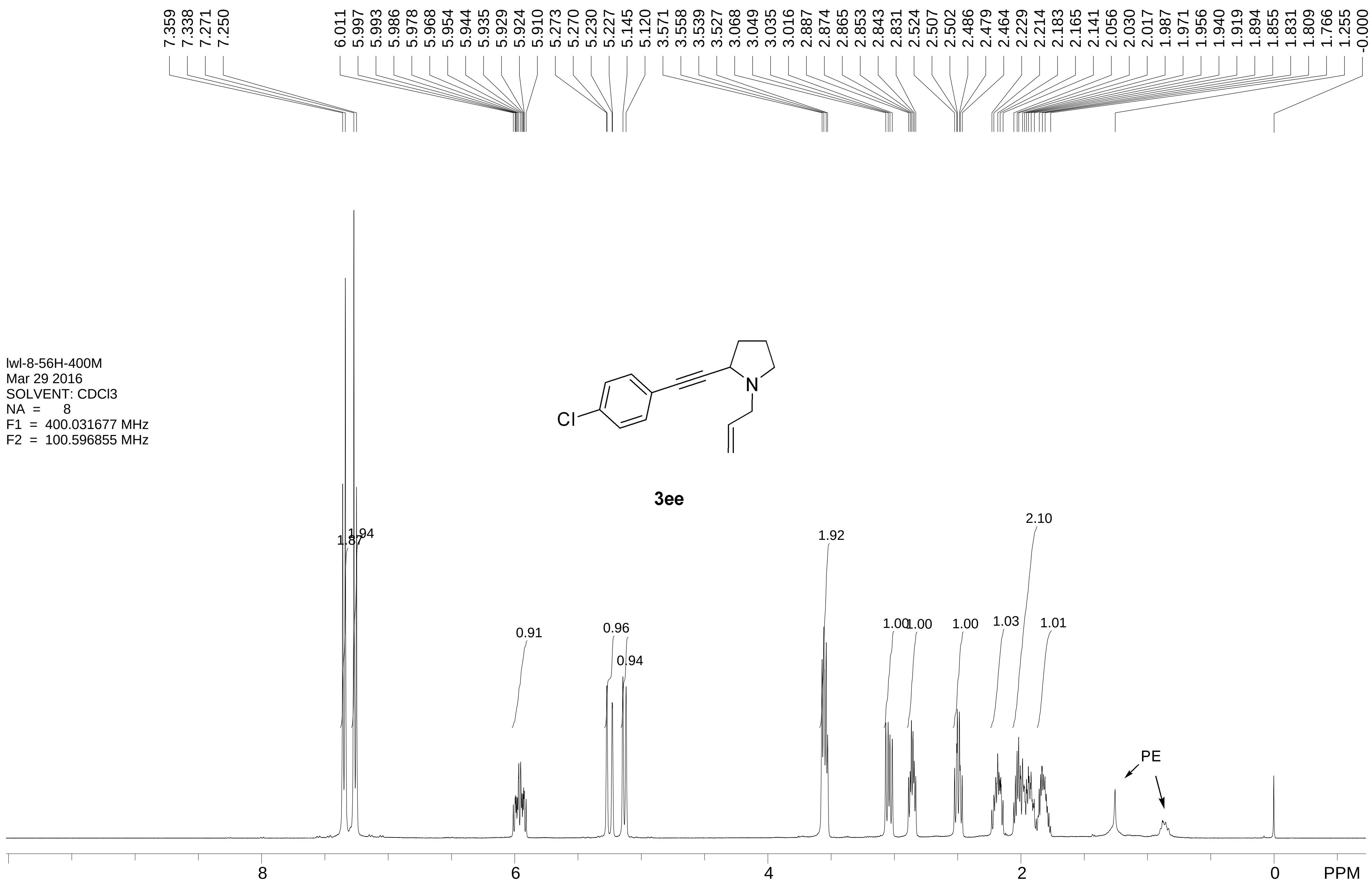
7.359 7.338 7.271 7.250 6.011 5.997 5.993 5.986 5.978 5.968 5.954 5.944 5.935 5.929 5.924 5.910 5.273 5.270 5.230 5.227 5.145 5.120 3.571 3.558 3.539 3.527 3.068 3.049 3.035 3.016 2.887 2.874 2.865 2.853 2.843 2.831 2.524 2.507 2.502 2.486 2.479 2.464 2.229 2.214 2.183 2.165 2.141 2.056 2.030 2.017 1.987 1.971 1.956 1.940 1.919 1.894 1.855 1.831 1.809 1.766 1.255 -0.000
lwl-8-56H-400M
Mar 29 2016
SOLVENT: CDCl3
NA = 8
F1 = 400.031677 MHz
F2 = 100.596855 MHz
Cl
N
3ee
1.87 1.94 0.91 0.96 0.94 1.92 1.00 1.00 1.00 1.03 2.10 1.01
PE
8 6 4 2 0 PPM

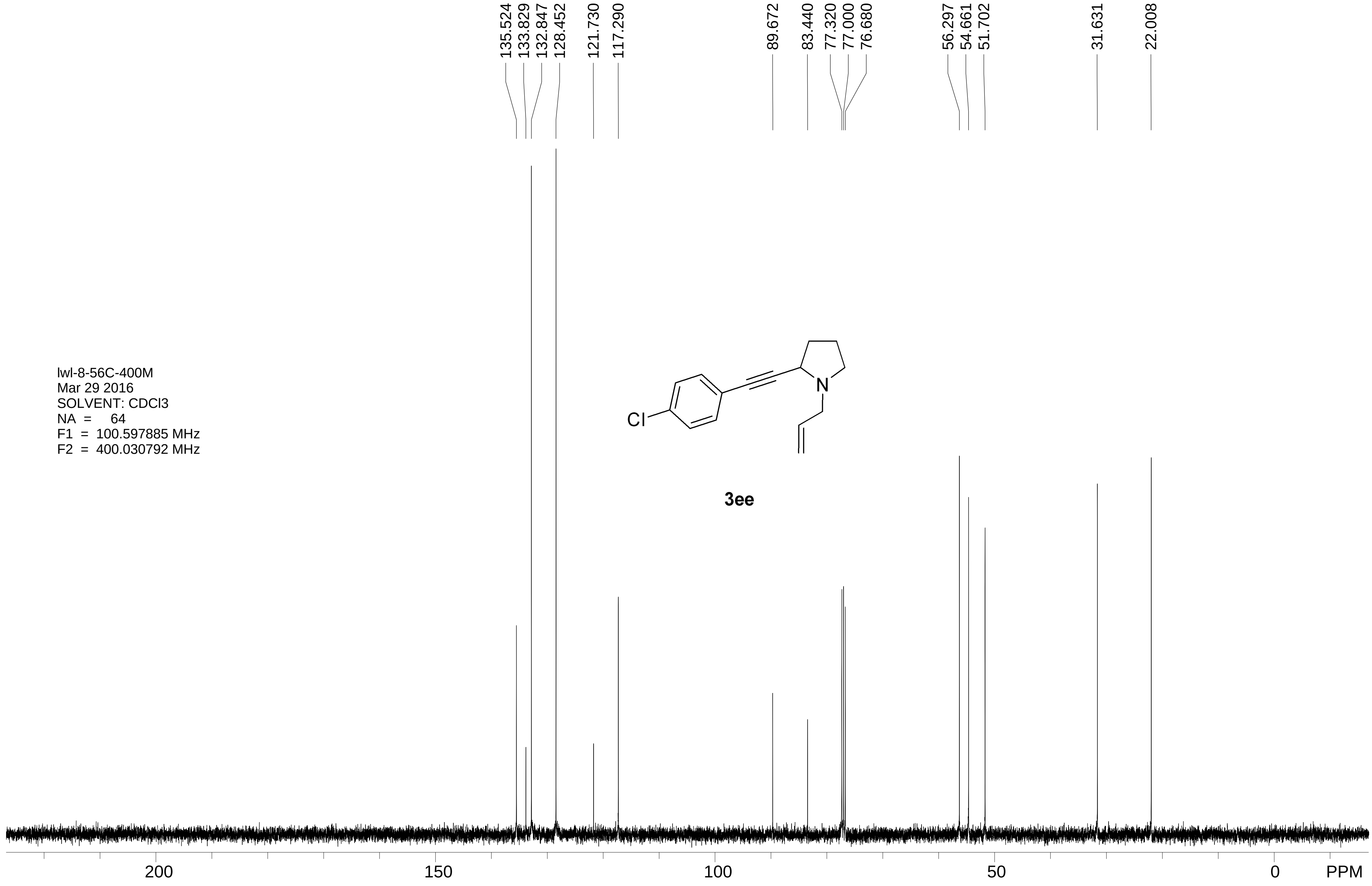
135.524
133.829
132.847
128.452
121.730
117.290
89.672
83.440
77.320
77.000
76.680
56.297
54.661
51.702
31.631
22.008
lwl-8-56C-400M
Mar 29 2016
SOLVENT: CDCl3
NA = 64
F1 = 100.597885 MHz
F2 = 400.030792 MHz
Cl
N
3ee
200
150
100
50
0
PPM

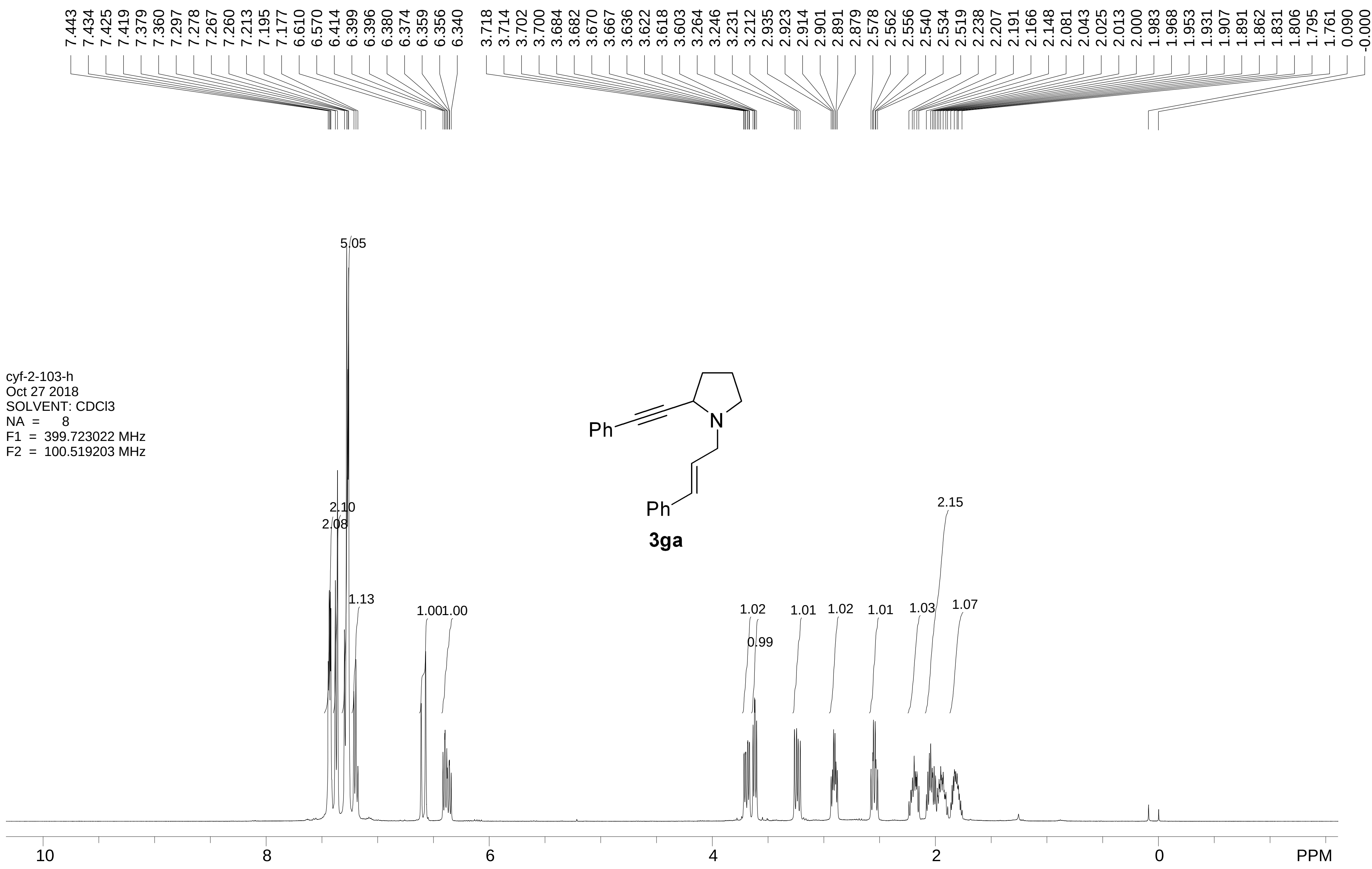
7.443
7.434
7.425
7.419
7.379
7.360
7.297
7.278
7.267
7.260
7.213
7.195
7.177
6.610
6.570
6.414
6.399
6.396
6.380
6.374
6.359
6.356
6.340
3.718
3.714
3.702
3.700
3.684
3.682
3.670
3.667
3.636
3.622
3.618
3.603
3.264
3.246
3.231
3.212
2.935
2.923
2.914
2.901
2.891
2.879
2.578
2.562
2.556
2.540
2.534
2.519
2.238
2.207
2.191
2.166
2.148
2.081
2.043
2.025
2.013
2.000
1.983
1.968
1.953
1.931
1.907
1.891
1.862
1.831
1.806
1.795
1.761
0.090
-0.000
cyf-2-103-h
Oct 27 2018
SOLVENT: CDCl3
NA = 8
F1 = 399.723022 MHz
F2 = 100.519203 MHz
Ph
N
Ph
3ga
5.05
2.10
2.08
1.13
1.00
1.00
1.02
0.99
1.01
1.02
1.01
1.03
2.15
1.07
10
8
6
4
2
0
PPM

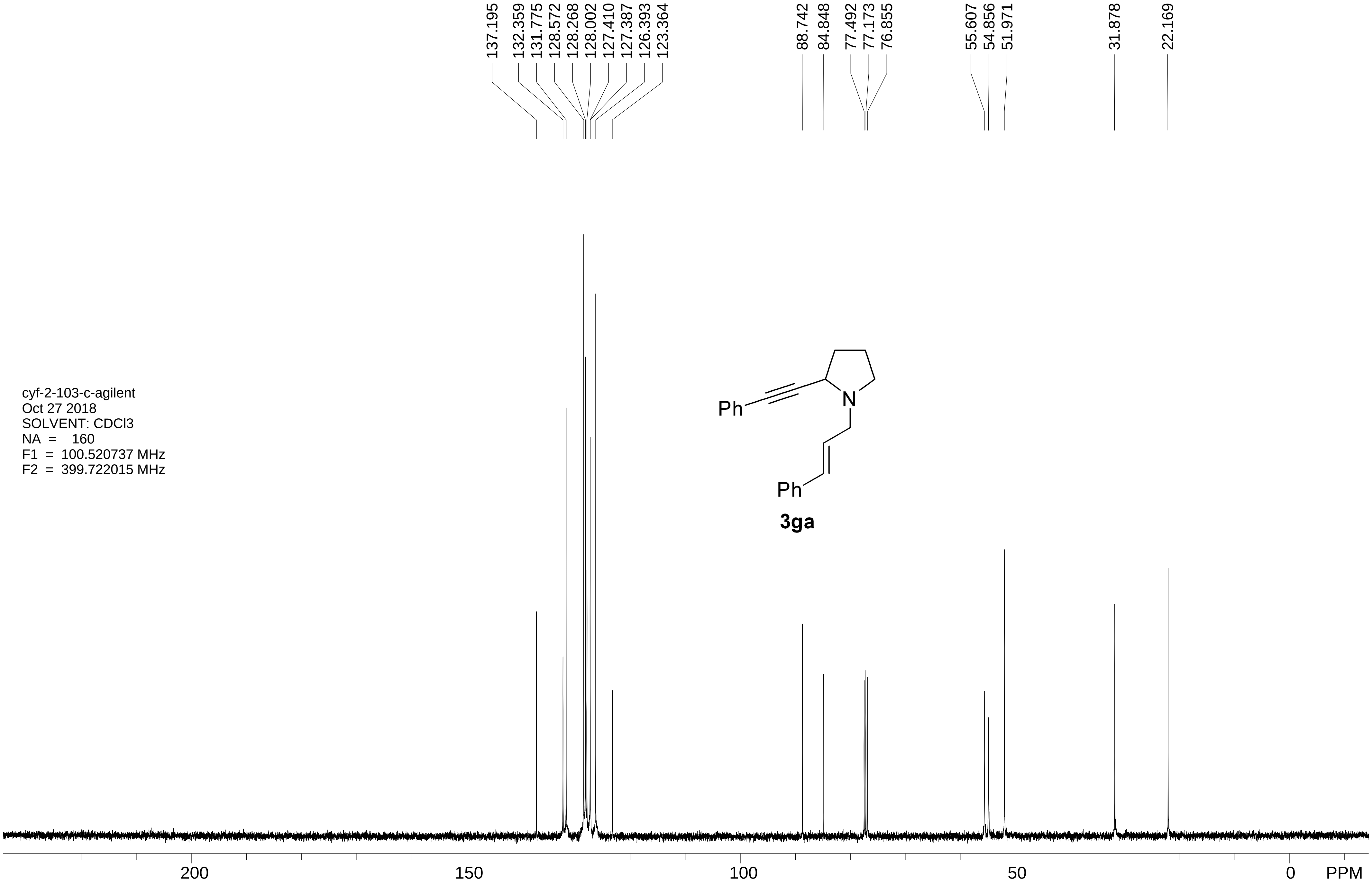
137.195
132.359
131.775
128.572
128.268
128.002
127.410
127.387
126.393
123.364
88.742
84.848
77.492
77.173
76.855
55.607
54.856
51.971
31.878
22.169
cyf-2-103-c-agilent
Oct 27 2018
SOLVENT: CDCl3
NA = 160
F1 = 100.520737 MHz
F2 = 399.722015 MHz
Ph
N
Ph
3ga
200
150
100
50
0
PPM

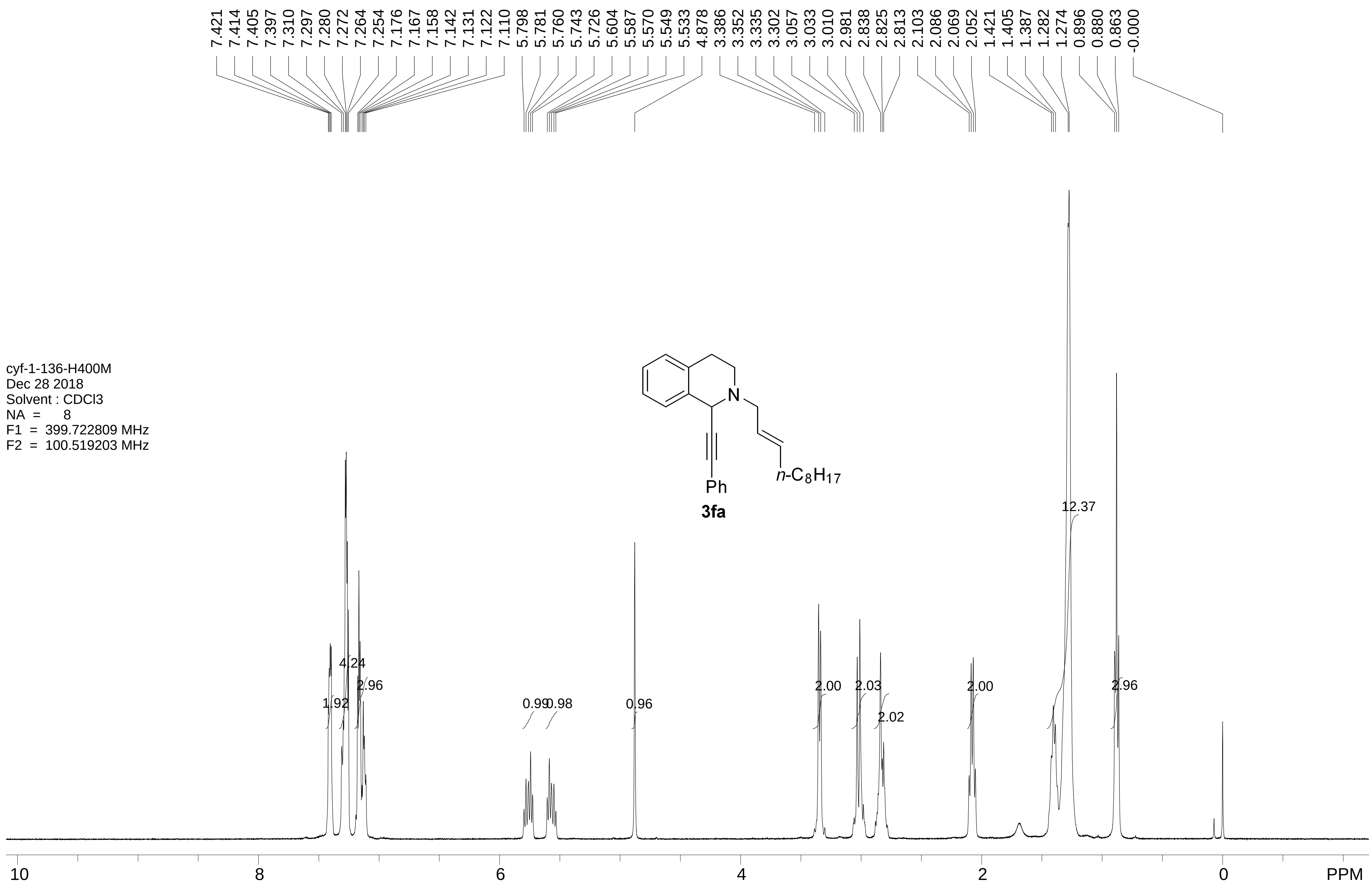
7.421 7.414 7.405 7.397 7.310 7.297 7.280 7.272 7.264 7.254 7.176 7.167 7.158 7.142 7.131 7.122 7.110 5.798 5.781 5.760 5.743 5.726 5.604 5.587 5.570 5.549 5.533 4.878 3.386 3.352 3.335 3.302 3.057 3.033 3.010 2.981 2.838 2.825 2.813 2.103 2.086 2.069 2.052 1.421 1.405 1.387 1.282 1.274 0.896 0.880 0.863 -0.000
cyf-1-136-H400M
Dec 28 2018
Solvent : CDCl3
NA = 8
F1 = 399.722809 MHz
F2 = 100.519203 MHz
N
n-C8H17
Ph
3fa
1.92
4.24
2.96
0.99
0.98
0.96
2.00
2.03
2.02
2.00
12.37
2.96
10
8
6
4
2
0
PPM

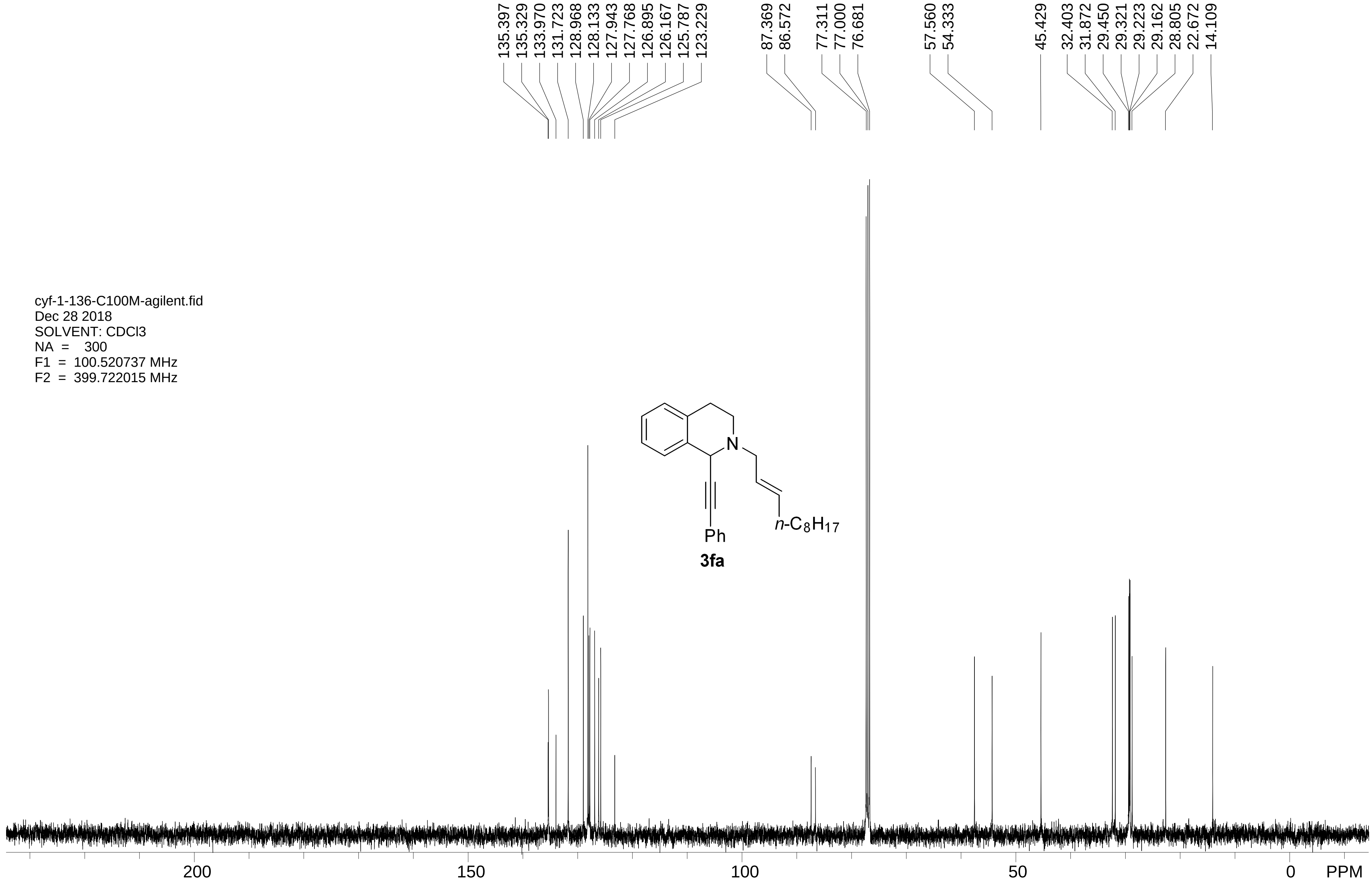
135.397
135.329
133.970
131.723
128.968
128.133
127.943
127.768
126.895
126.167
125.787
123.229
87.369
86.572
77.311
77.000
76.681
57.560
54.333
45.429
32.403
31.872
29.450
29.321
29.223
29.162
28.805
22.672
14.109
cyf-1-136-C100M-agilent.fid
Dec 28 2018
SOLVENT: CDCl3
NA = 300
F1 = 100.520737 MHz
F2 = 399.722015 MHz
N
Ph
n-C8H17
3fa
200
150
100
50
0
PPM

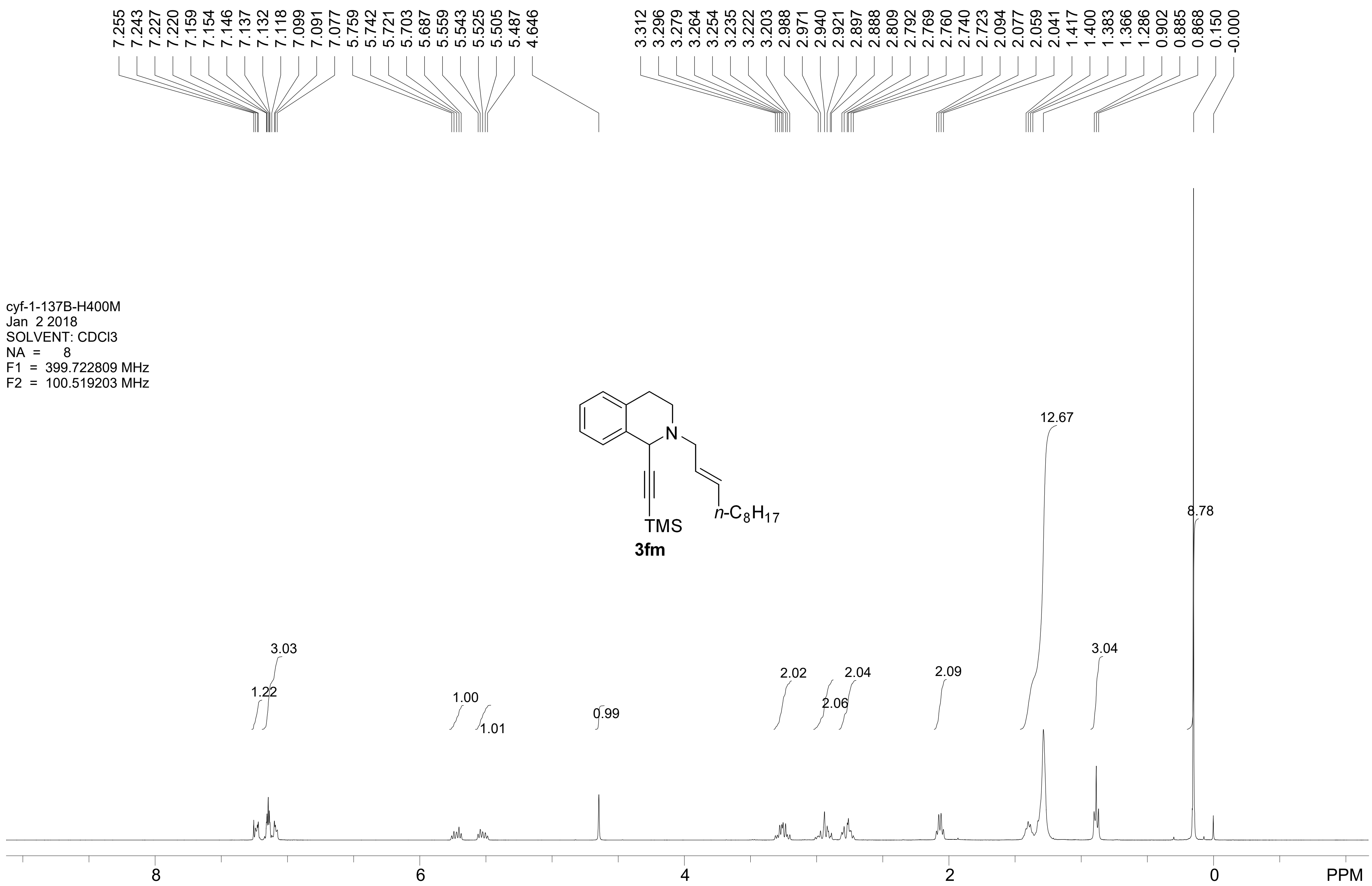

7.255 7.243 7.227 7.220 7.159 7.154 7.146 7.137 7.132 7.118 7.099 7.091 7.077 5.759 5.742 5.721 5.703 5.687 5.559 5.543 5.525 5.505 5.487 4.646
3.312 3.296 3.279 3.264 3.254 3.235 3.222 3.203 2.988 2.971 2.940 2.921 2.897 2.888 2.809 2.792 2.769 2.760 2.740 2.723 2.094 2.077 2.059 2.041 1.417 1.400 1.383 1.366 1.286 0.902 0.885 0.868 0.150 -0.000
cyf-1-137B-H400M
Jan 2 2018
SOLVENT: CDCl3
NA = 8
F1 = 399.722809 MHz
F2 = 100.519203 MHz
N
TMS
n-C8H17
3fm
12.67
8.78
3.03
1.22
1.00
1.01
0.99
2.02
2.06
2.04
2.09
3.04
8
6
4
2
0
PPM

cyf-1-137B-C100M-Varian
Jan 3 2018
SOLVENT: CDCl3
NA = 328
F1 = 100.598389 MHz
F2 = 400.030792 MHz

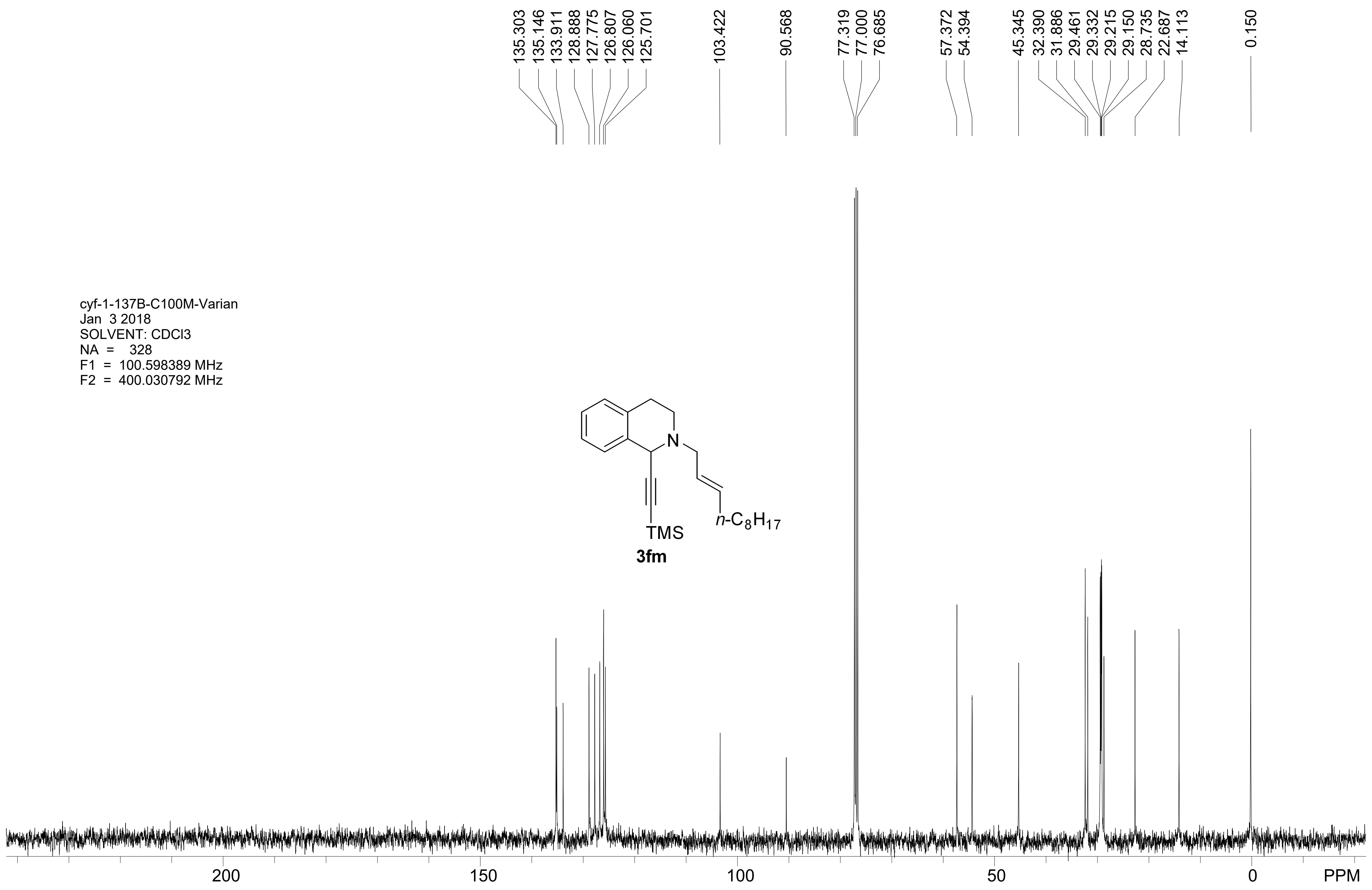

3fm

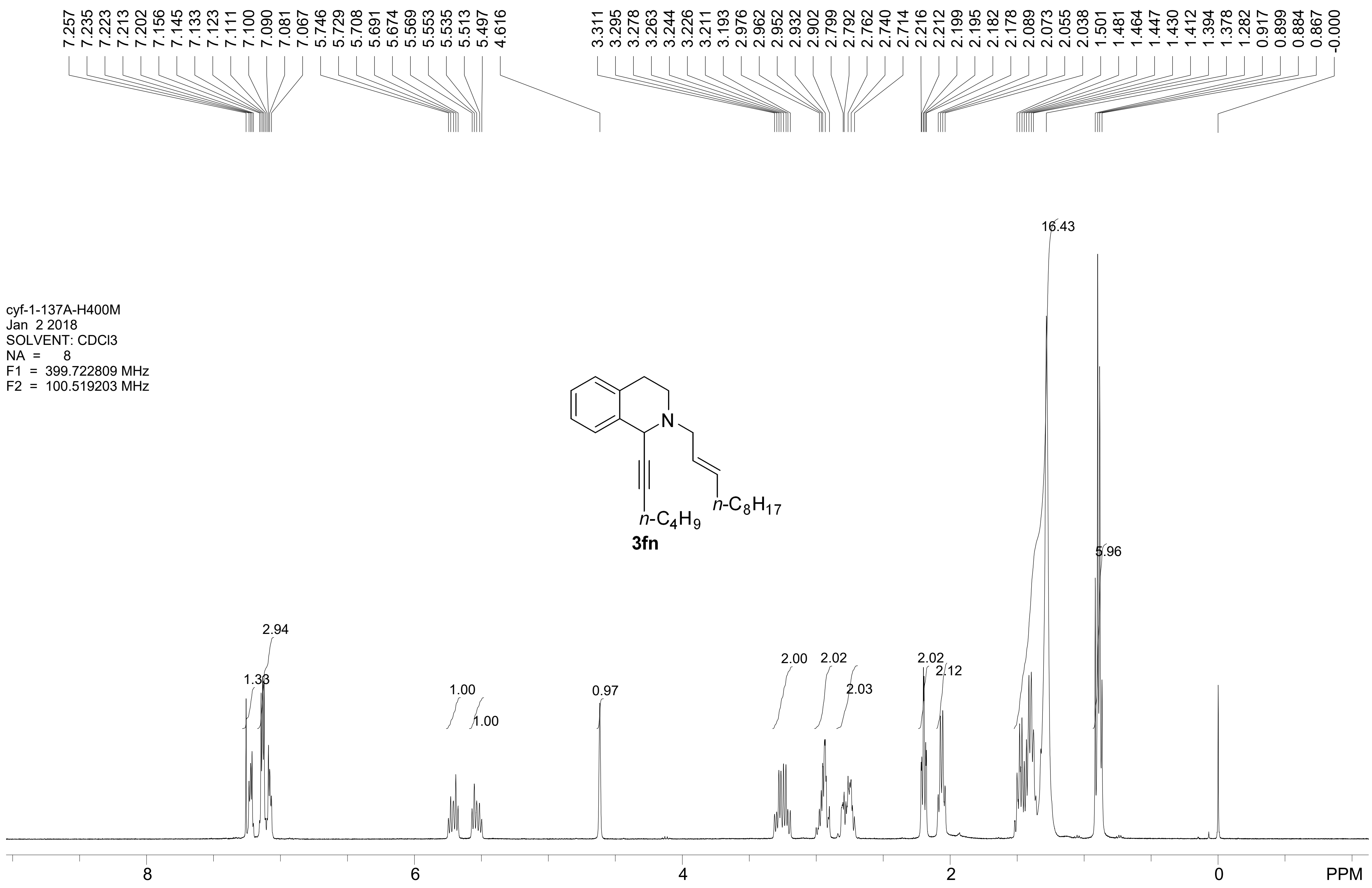
7.257 7.235 7.223 7.213 7.202 7.156 7.145 7.133 7.123 7.111 7.100 7.090 7.081 7.067 5.746 5.729 5.708 5.691 5.674 5.569 5.553 5.535 5.513 5.497 4.616
3.311 3.295 3.278 3.263 3.244 3.226 3.211 3.193 2.976 2.962 2.952 2.932 2.902 2.799 2.792 2.762 2.740 2.714 2.216 2.212 2.199 2.195 2.182 2.178 2.089 2.073 2.055 2.038 1.501 1.481 1.464 1.447 1.430 1.412 1.394 1.378 1.282 0.917 0.899 0.884 0.867 -0.000
cyf-1-137A-H400M
Jan 2 2018
SOLVENT: CDCl3
NA = 8
F1 = 399.722809 MHz
F2 = 100.519203 MHz
N
n-C4H9
n-C8H17
3fn
16.43
5.96
2.94
1.33
1.00
1.00
0.97
2.00
2.02
2.03
2.02
2.12
8
6
4
2
0
PPM

136.232
135.018
133.742
128.854
127.647
126.637
126.349
125.643
86.845
77.615
77.319
77.000
76.681
57.377
53.999
45.240
32.396
31.879
31.037
29.458
29.329
29.230
29.162
28.767
22.679
21.951
18.504
14.109
13.593

cyf-1-137A-C100M-Agilent
Jan 2 2018
SOLVENT: CDCl3
NA = 240
F1 = 100.520737 MHz
F2 = 399.722015 MHz

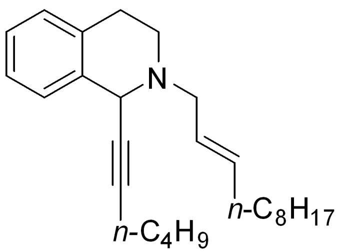

3fn

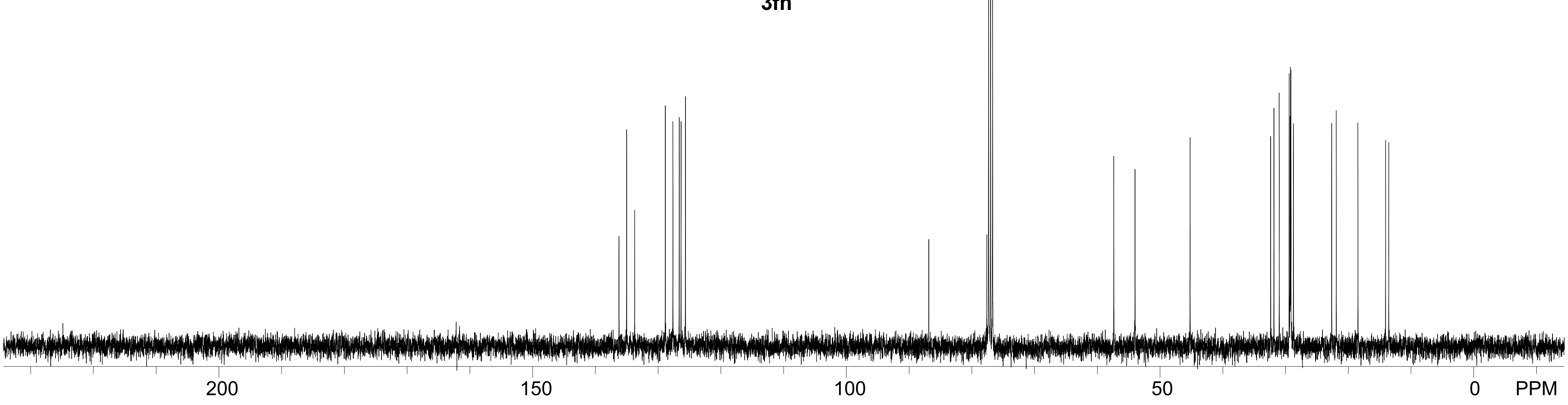

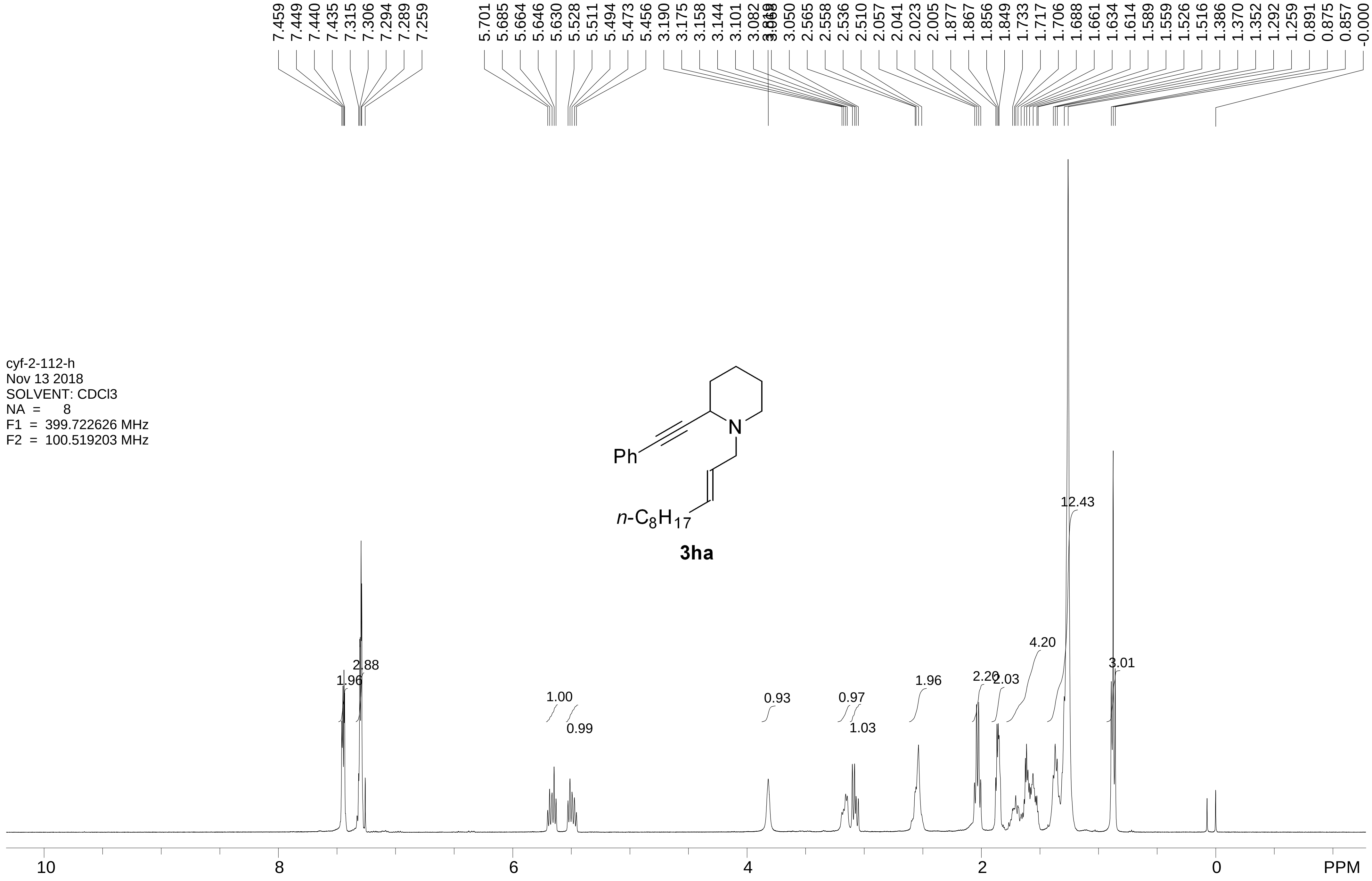

7.459 7.449 7.440 7.435 7.315 7.306 7.294 7.289 7.259
5.701 5.685 5.664 5.646 5.630 5.528 5.511 5.494 5.473 5.456
3.190 3.175 3.158 3.144 3.101 3.082 3.050
2.565 2.558 2.536 2.510 2.057 2.041 2.023 2.005
1.877 1.867 1.856 1.849 1.733 1.717 1.706 1.688 1.661 1.634 1.614 1.589 1.559 1.526 1.516 1.386 1.370 1.352 1.292 1.259
0.891 0.875 0.857 -0.000
cyf-2-112-h
Nov 13 2018
SOLVENT: CDCl3
NA = 8
F1 = 399.722626 MHz
F2 = 100.519203 MHz
Ph
N
n-C8H17
3ha
1.96
2.88
1.00
0.99
0.93
0.97
1.03
1.96
2.20
2.03
4.20
12.43
3.01
10
8
6
4
2
0
PPM

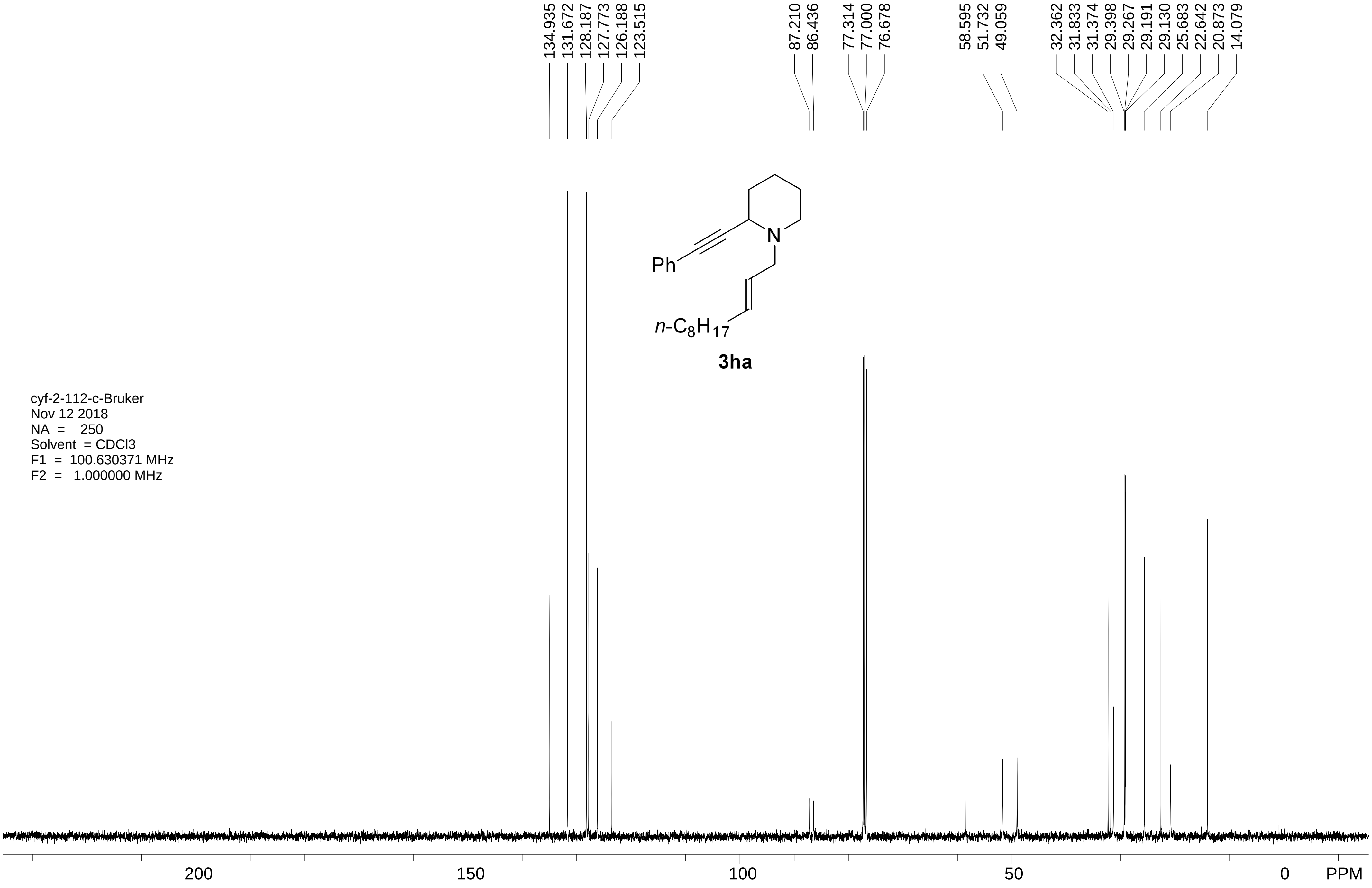
134.935
131.672
128.187
127.773
126.188
123.515
87.210
86.436
77.314
77.000
76.678
58.595
51.732
49.059
32.362
31.833
31.374
29.398
29.267
29.191
29.130
25.683
22.642
20.873
14.079
Ph
N
n-C8H17
3ha
cyf-2-112-c-Bruker
Nov 12 2018
NA = 250
Solvent = CDCl3
F1 = 100.630371 MHz
F2 = 1.000000 MHz
200
150
100
50
0
PPM

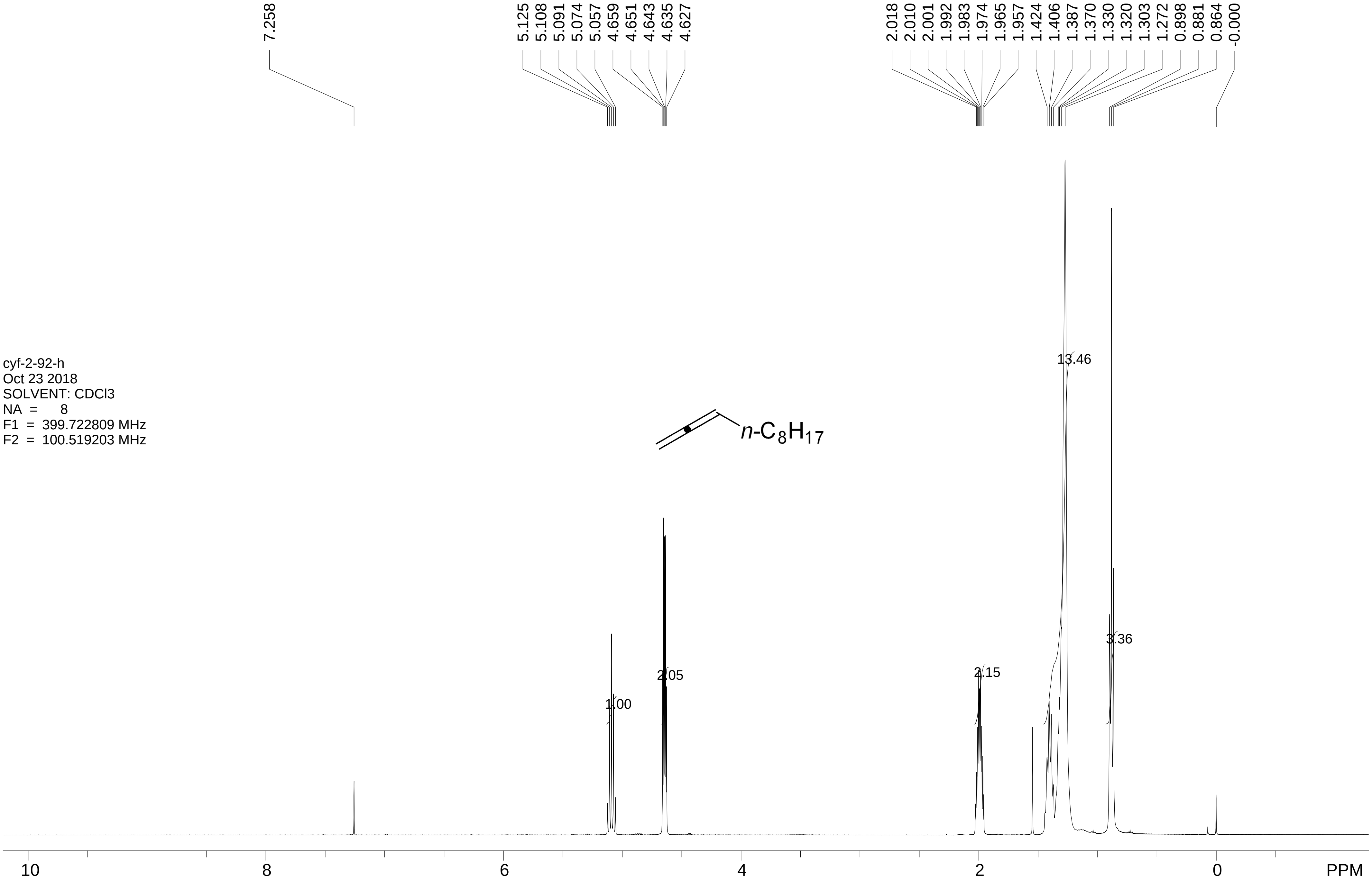
7.258
5.125
5.108
5.091
5.074
5.057
4.659
4.651
4.643
4.635
4.627
2.018
2.010
2.001
1.992
1.983
1.974
1.965
1.957
1.424
1.406
1.387
1.370
1.330
1.320
1.303
1.272
0.898
0.881
0.864
-0.000
cyf-2-92-h
Oct 23 2018
SOLVENT: CDCl3
NA = 8
F1 = 399.722809 MHz
F2 = 100.519203 MHz
n-C8H17
13.46
3.36
2.05
2.15
1.00
10
8
6
4
2
0
PPM

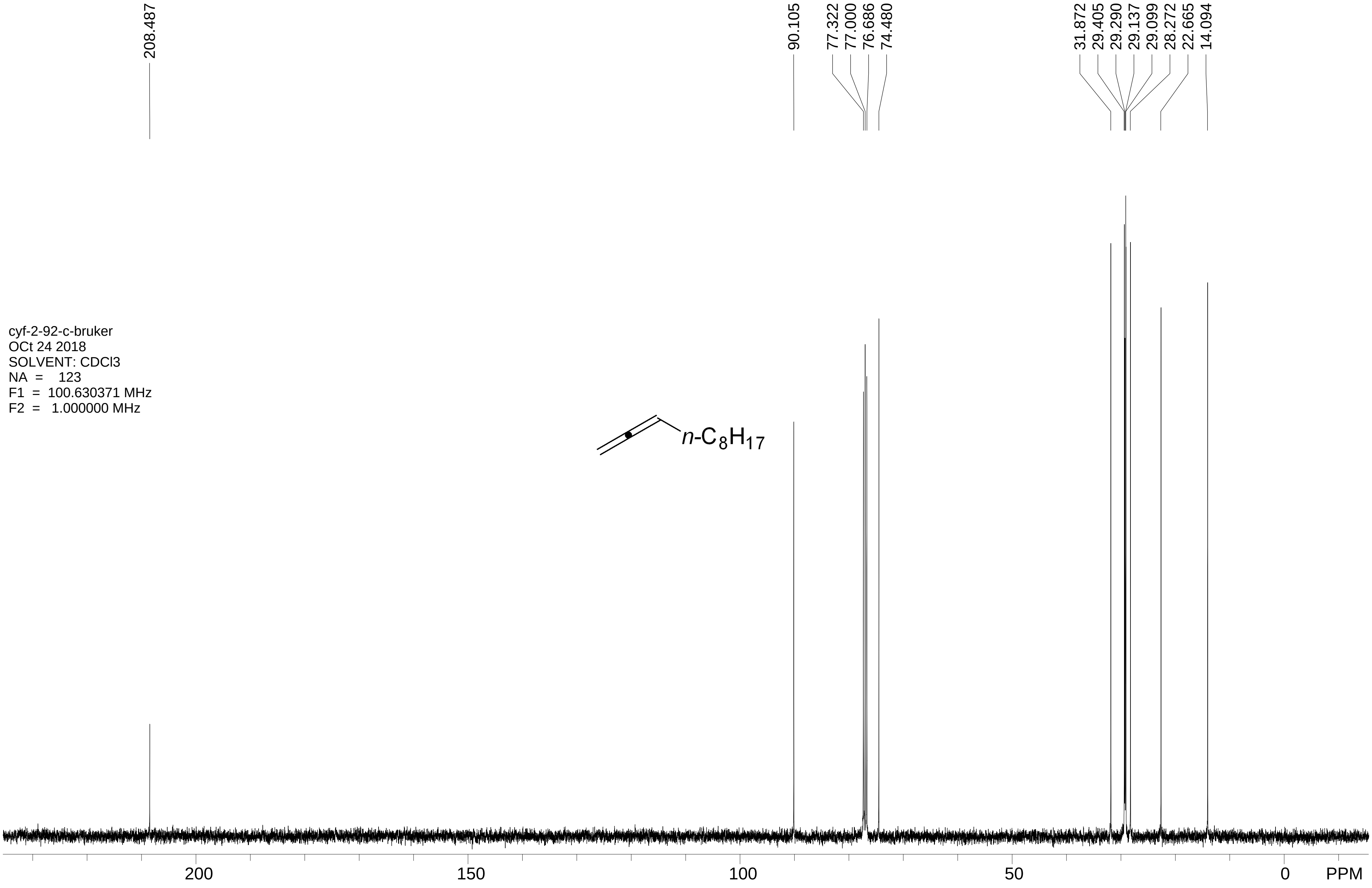
208.487
90.105
77.322
77.000
76.686
74.480
31.872
29.405
29.290
29.137
29.099
28.272
22.665
14.094
cyf-2-92-c-bruker
OCt 24 2018
SOLVENT: CDCl3
NA = 123
F1 = 100.630371 MHz
F2 = 1.000000 MHz
n-C8H17
200
150
100
50
0
PPM

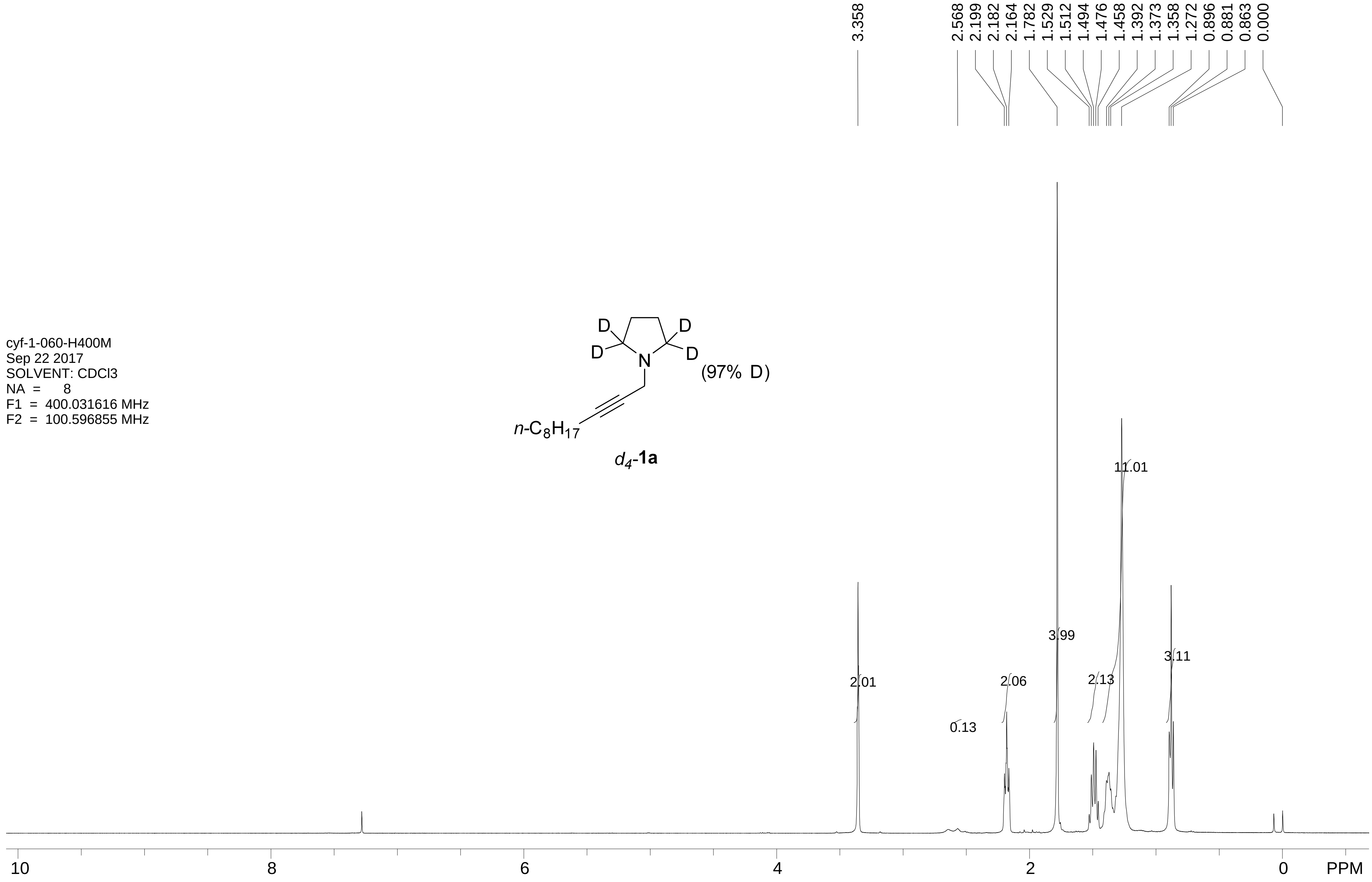
3.358
2.568
2.199
2.182
2.164
1.782
1.529
1.512
1.494
1.476
1.458
1.392
1.373
1.358
1.272
0.896
0.881
0.863
0.000
cyf-1-060-H400M
Sep 22 2017
SOLVENT: CDCl3
NA = 8
F1 = 400.031616 MHz
F2 = 100.596855 MHz
D
D
D
D
N
(97% D)
n-C8H17
d4-1a
11.01
3.99
3.11
2.01
2.06
2.13
0.13
10
8
6
4
2
0
PPM

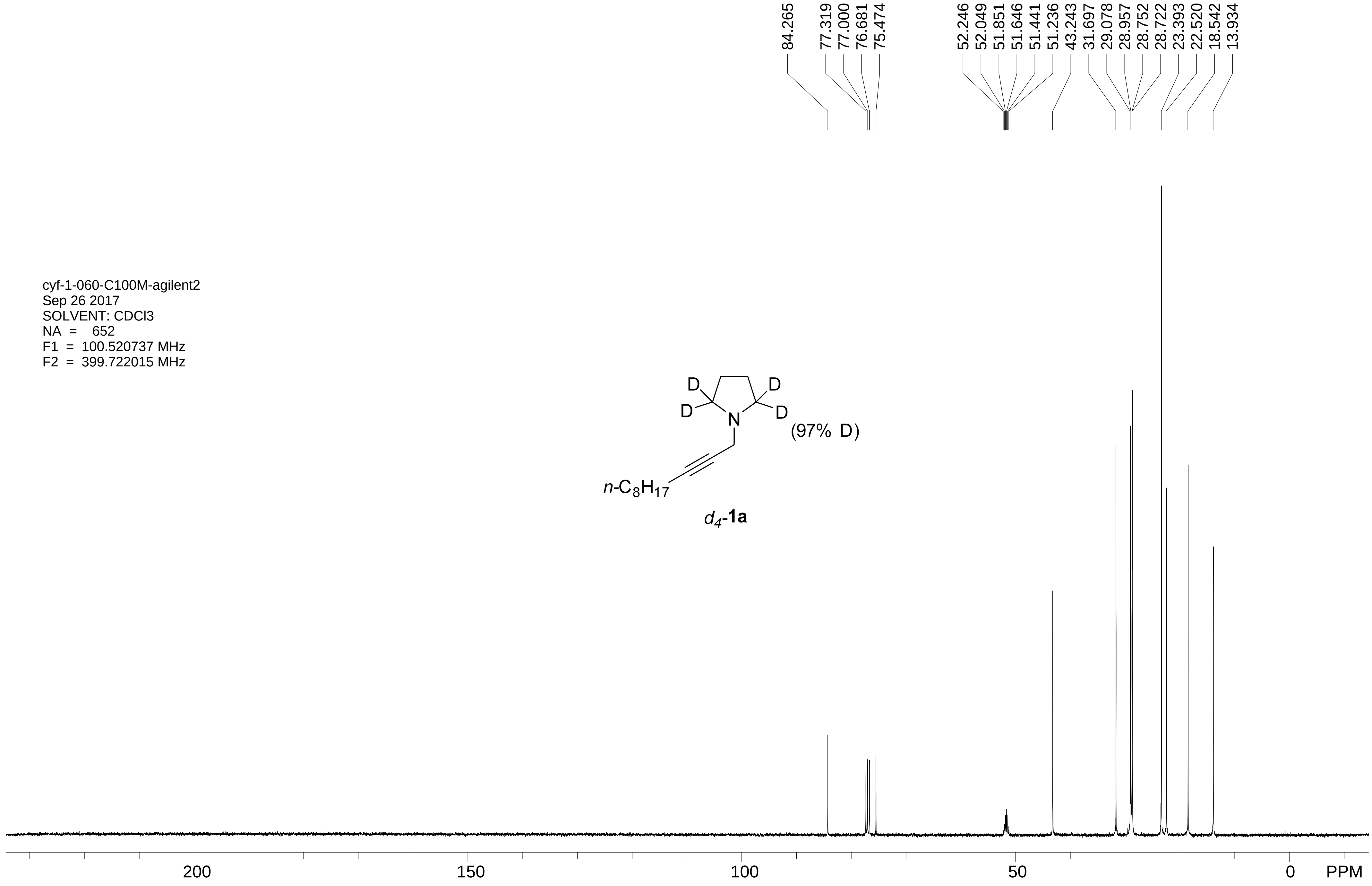
84.265
77.319
77.000
76.681
75.474
52.246
52.049
51.851
51.646
51.441
51.236
43.243
31.697
29.078
28.957
28.752
28.722
23.393
22.520
18.542
13.934
cyf-1-060-C100M-agilent2
Sep 26 2017
SOLVENT: CDCl3
NA = 652
F1 = 100.520737 MHz
F2 = 399.722015 MHz
D
D
D
D
N
(97% D)
n-C8H17
d4-1a
200
150
100
50
0
PPM

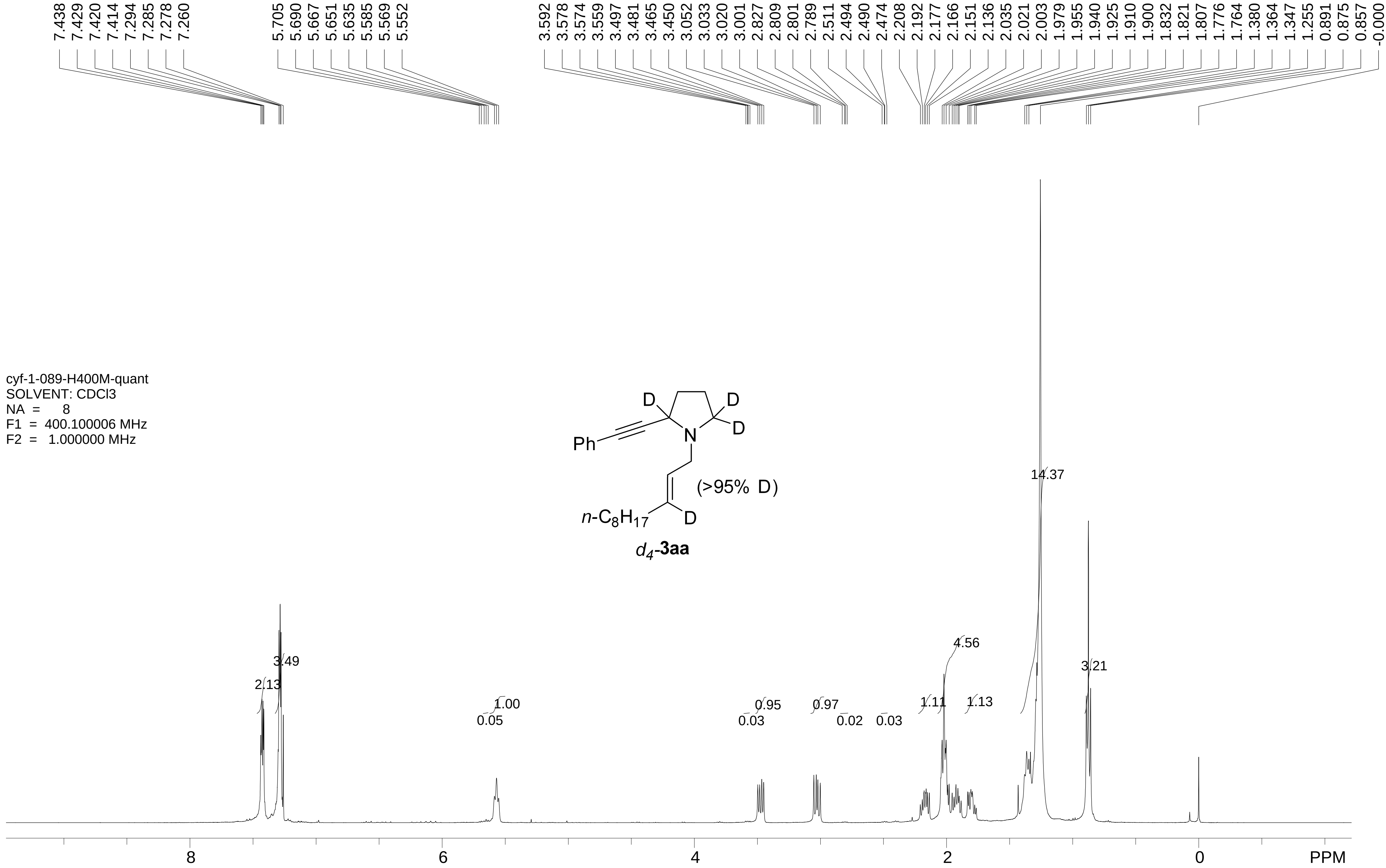

7.438
7.429
7.420
7.414
7.294
7.285
7.278
7.260
5.705
5.690
5.667
5.651
5.635
5.585
5.569
5.552
3.592
3.578
3.574
3.559
3.497
3.481
3.465
3.450
3.052
3.033
3.020
3.001
2.827
2.809
2.801
2.789
2.511
2.494
2.490
2.474
2.208
2.192
2.177
2.166
2.151
2.136
2.035
2.021
2.003
1.979
1.955
1.940
1.925
1.910
1.900
1.832
1.821
1.807
1.776
1.764
1.380
1.364
1.347
1.255
0.891
0.875
0.857
-0.000
cyf-1-089-H400M-quant
SOLVENT: CDCl3
NA = 8
F1 = 400.100006 MHz
F2 = 1.000000 MHz
D
D
D
Ph
N
(>95% D)
n-C8H17
D
d4-3aa
14.37
4.56
3.49
2.13
3.21
1.00
0.05
0.95
0.03
0.97
0.02
0.03
1.11
1.13
8
6
4
2
0
PPM

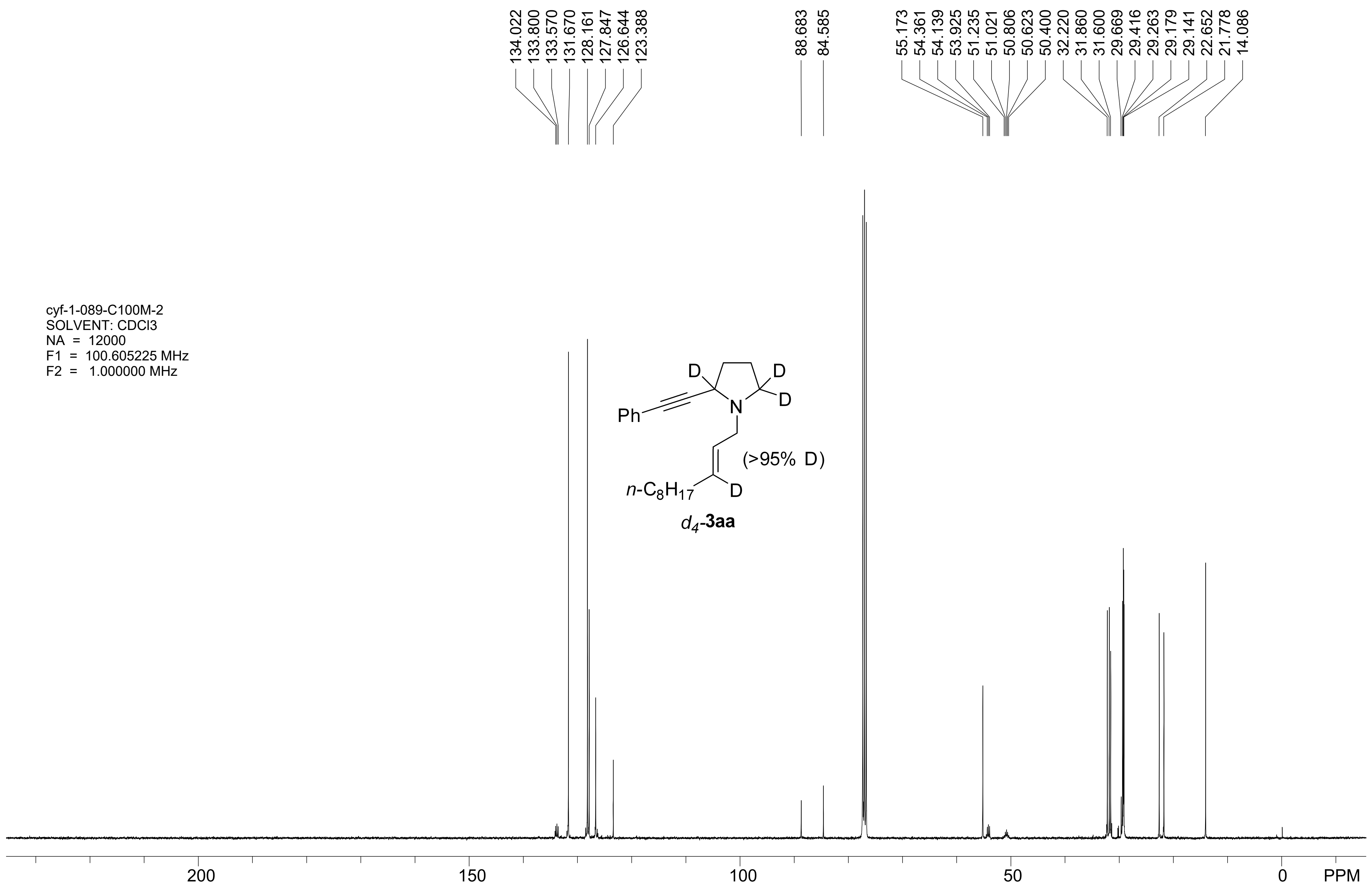
134.022
133.800
133.570
131.670
128.161
127.847
126.644
123.388
88.683
84.585
55.173
54.361
54.139
53.925
51.235
51.021
50.806
50.623
50.400
32.220
31.860
31.600
29.669
29.416
29.263
29.179
29.141
22.652
21.778
14.086
cyf-1-089-C100M-2
SOLVENT: CDCl3
NA = 12000
F1 = 100.605225 MHz
F2 = 1.000000 MHz
Ph
N
D
D
D
(>95% D)
n-C_8H_{17}
D
d_4-**3aa**
200
150
100
50
0
PPM

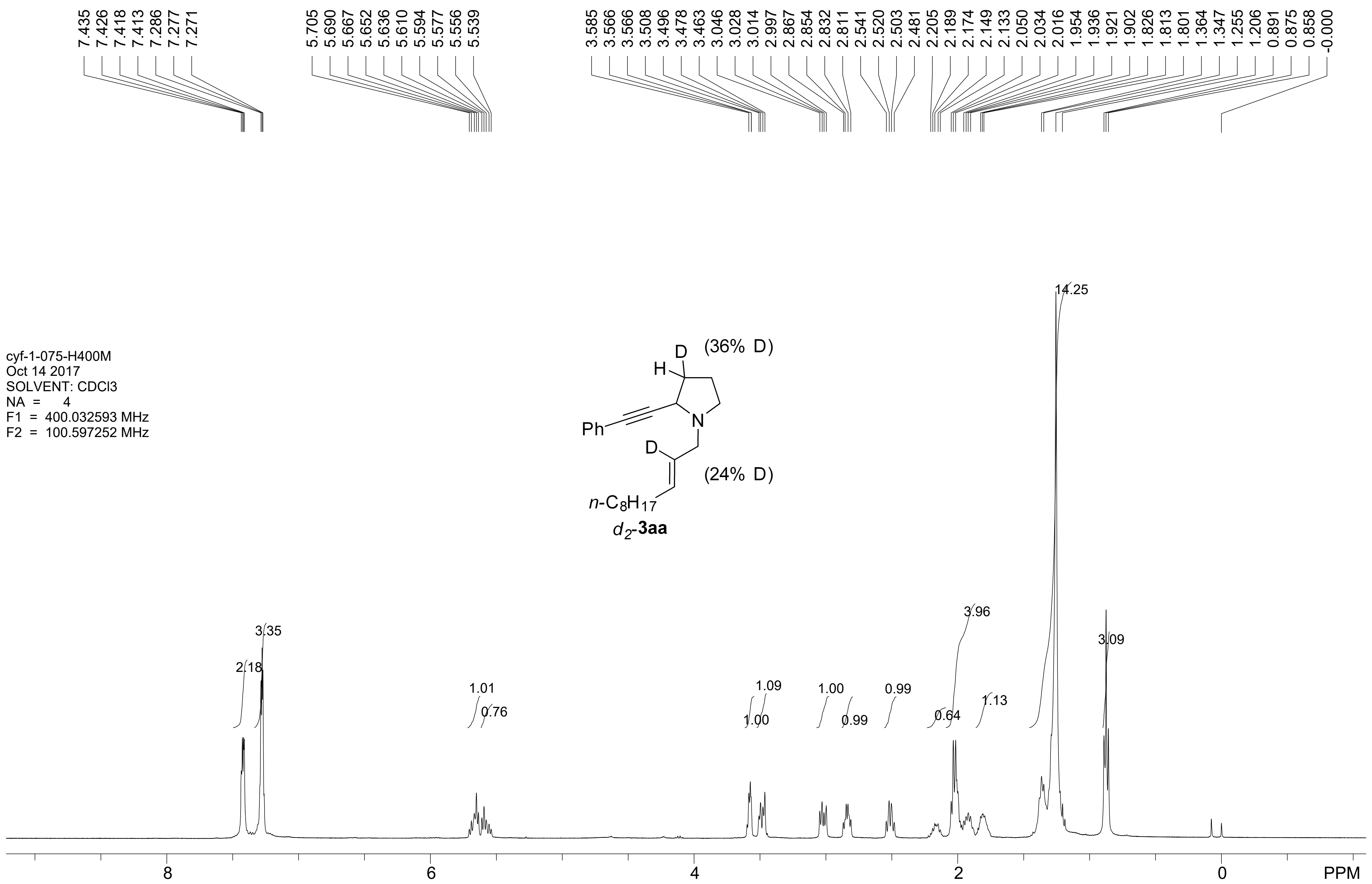
7.435 7.426 7.418 7.413 7.286 7.277 7.271
5.705 5.690 5.667 5.652 5.636 5.610 5.594 5.577 5.556 5.539
3.585 3.566 3.566 3.508 3.496 3.478 3.463 3.046 3.028 3.014 2.997 2.867 2.854 2.832 2.811 2.541 2.520 2.503 2.481 2.205 2.189 2.174 2.149 2.133 2.050 2.034 2.016 1.954 1.936 1.921 1.902 1.826 1.813 1.801 1.364 1.347 1.255 1.206 0.891 0.875 0.858 -0.000
cyf-1-075-H400M
Oct 14 2017
SOLVENT: CDCl3
NA = 4
F1 = 400.032593 MHz
F2 = 100.597252 MHz
(36% D)
(24% D)
Ph
N
D
H
n-C8H17
d2-3aa
14.25
3.96
3.09
3.35
2.18
1.01
0.76
1.09
1.00
1.00
0.99
0.99
0.64
1.13
8
6
4
2
0
PPM

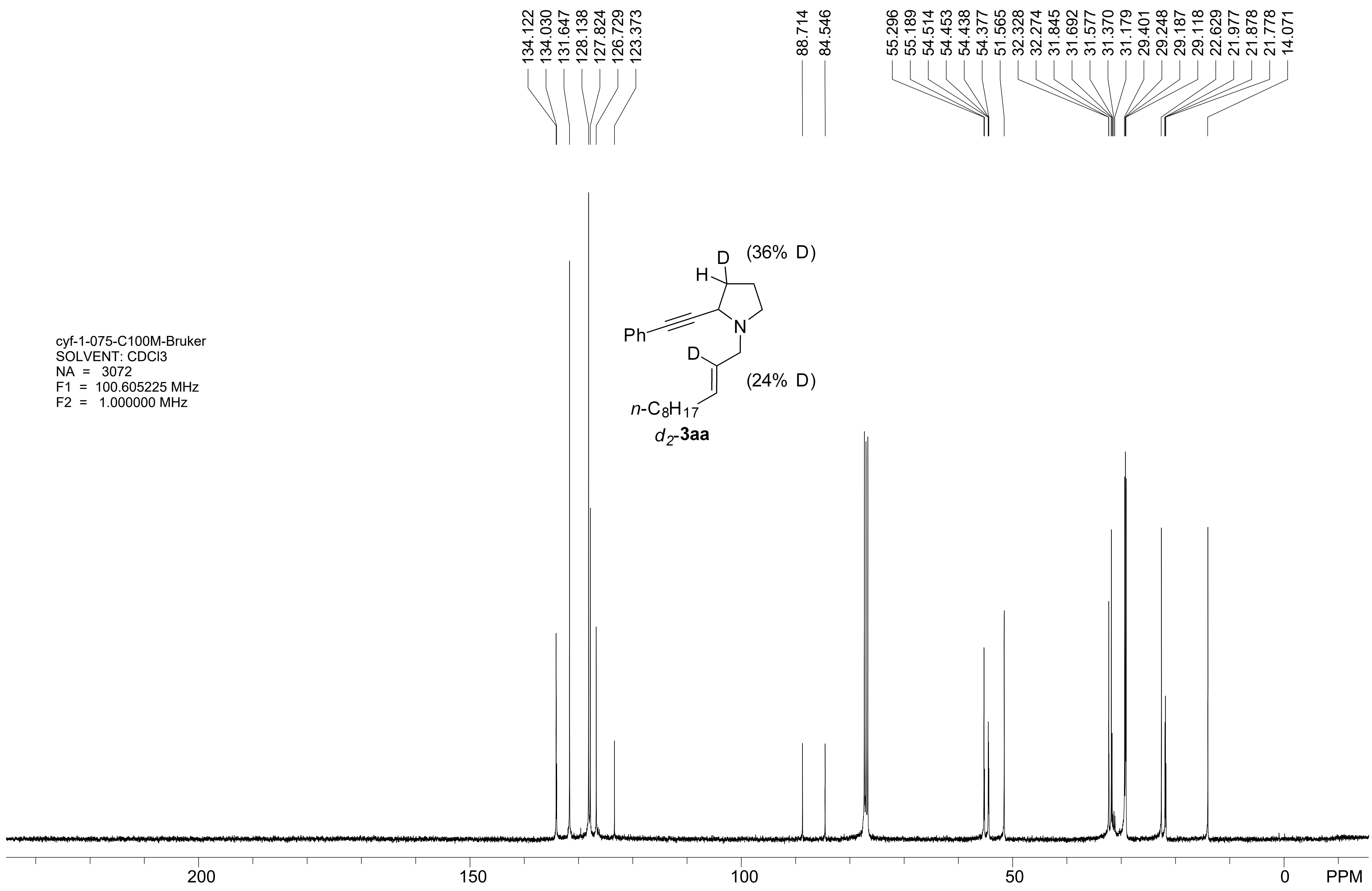
134.122
134.030
131.647
128.138
127.824
126.729
123.373
88.714
84.546
55.296
55.189
54.514
54.453
54.438
54.377
51.565
32.328
32.274
31.845
31.692
31.577
31.370
31.179
29.401
29.248
29.187
29.118
22.629
21.977
21.878
21.778
14.071
(36% D)
D
H
N
Ph
D
(24% D)
n-C8H17
d2-3aa
cyf-1-075-C100M-Bruker
SOLVENT: CDCl3
NA = 3072
F1 = 100.605225 MHz
F2 = 1.000000 MHz
200
150
100
50
0
PPM

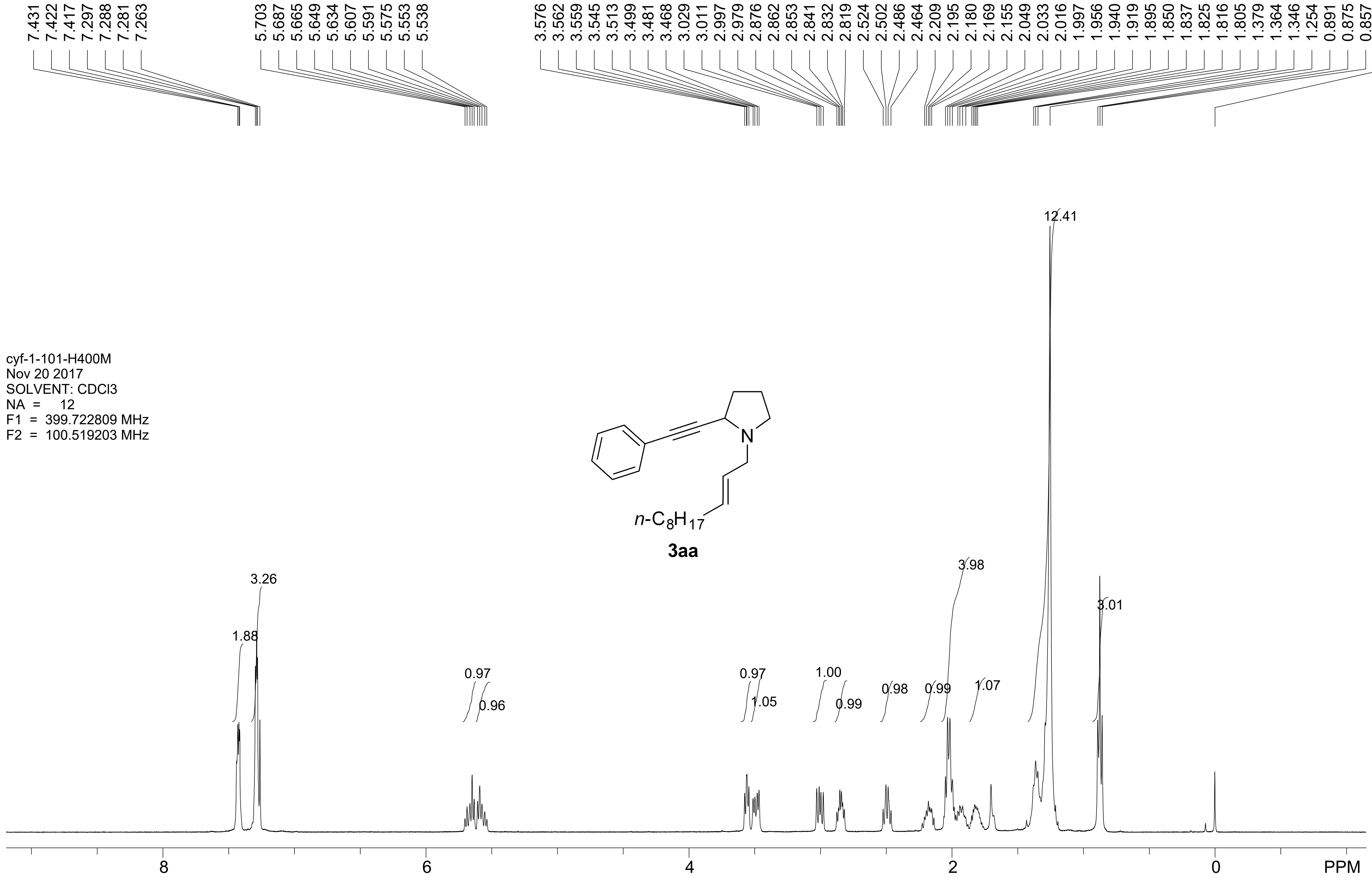
7.431 7.422 7.417 7.297 7.288 7.281 7.263
5.703 5.687 5.665 5.649 5.634 5.607 5.591 5.575 5.553 5.538
3.576 3.562 3.559 3.545 3.513 3.499 3.481 3.468 3.029 3.011 2.997 2.979 2.876 2.862 2.853 2.841 2.832 2.819 2.524 2.502 2.486 2.464 2.209 2.195 2.180 2.169 2.155 2.049 2.033 2.016 1.997 1.956 1.940 1.919 1.895 1.850 1.837 1.825 1.816 1.805 1.379 1.364 1.346 1.254 0.891 0.875 0.857 -0.000
cyf-1-101-H400M
Nov 20 2017
SOLVENT: CDCl3
NA = 12
F1 = 399.722809 MHz
F2 = 100.519203 MHz
N
n-C8H17
3aa
12.41
3.98
3.26
3.01
1.88
0.97
0.96
0.97
1.05
1.00
0.99
0.98
0.99
1.07
8
6
4
2
0
PPM

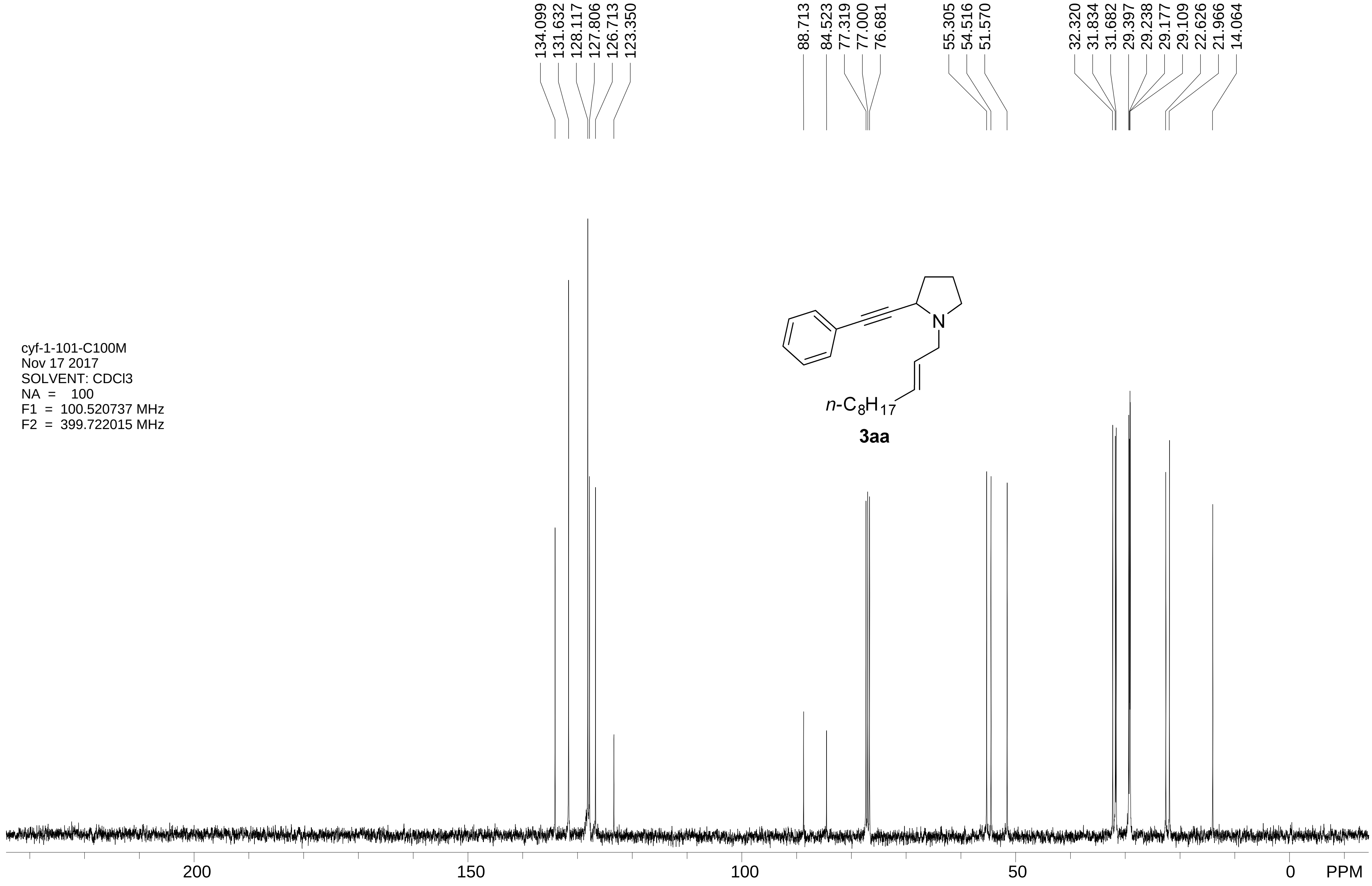
134.099
131.632
128.117
127.806
126.713
123.350
88.713
84.523
77.319
77.000
76.681
55.305
54.516
51.570
32.320
31.834
31.682
29.397
29.238
29.177
29.109
22.626
21.966
14.064
cyf-1-101-C100M
Nov 17 2017
SOLVENT: CDCl3
NA = 100
F1 = 100.520737 MHz
F2 = 399.722015 MHz
N
n-C8H17
3aa
200
150
100
50
0
PPM

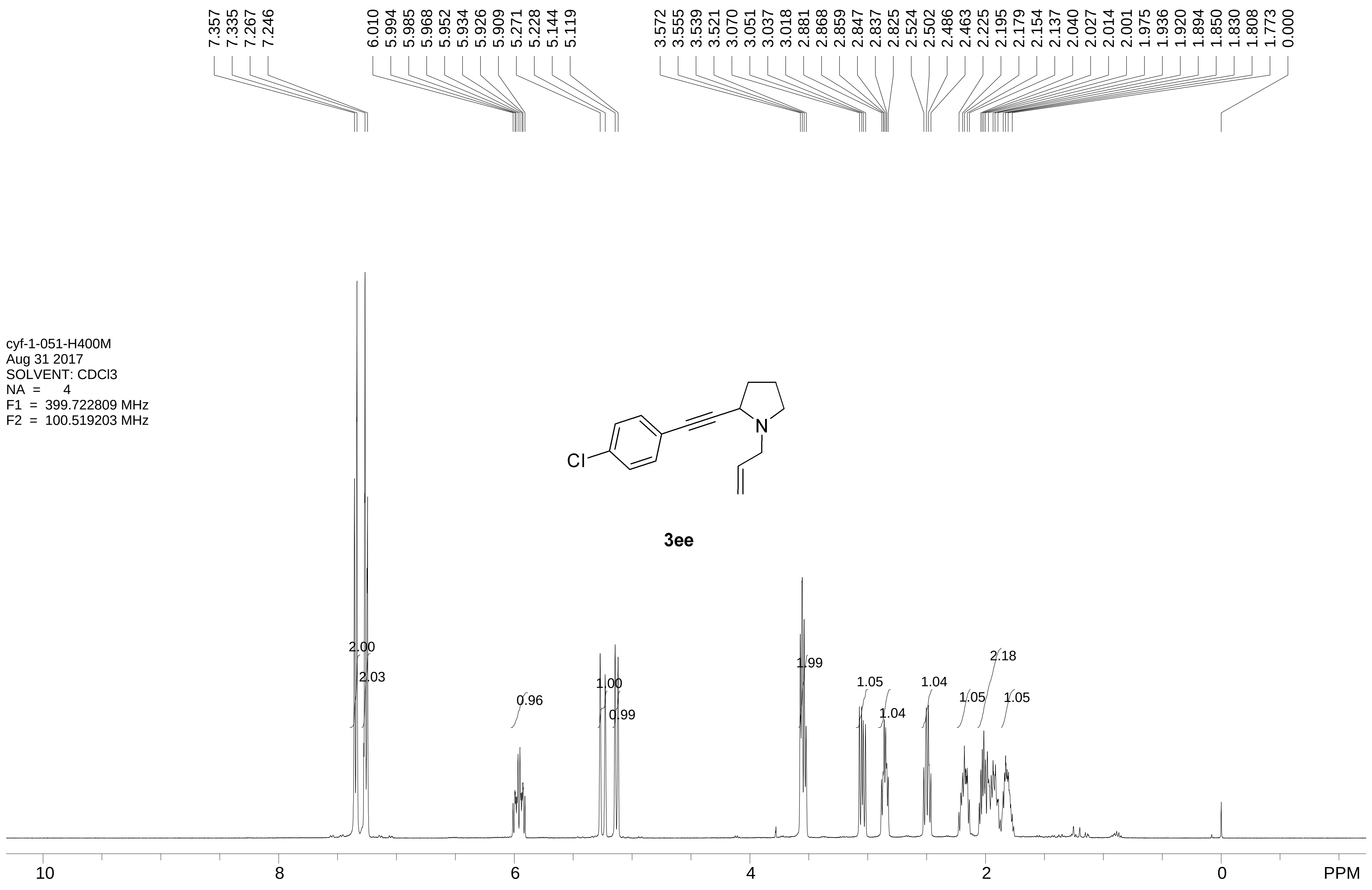
7.357
7.335
7.267
7.246
6.010
5.994
5.985
5.968
5.952
5.934
5.926
5.909
5.271
5.228
5.144
5.119
3.572
3.555
3.539
3.521
3.070
3.051
3.037
3.018
2.881
2.868
2.859
2.847
2.837
2.825
2.524
2.502
2.486
2.463
2.225
2.195
2.179
2.154
2.137
2.040
2.027
2.014
2.001
1.975
1.936
1.920
1.894
1.850
1.830
1.808
1.773
0.000
cyf-1-051-H400M
Aug 31 2017
SOLVENT: CDCl3
NA = 4
F1 = 399.722809 MHz
F2 = 100.519203 MHz
Cl
N
2.00
2.03
0.96
1.00
0.99
1.99
1.05
1.04
1.04
2.18
1.05
1.05
10
8
6
4
2
0
PPM

3ee

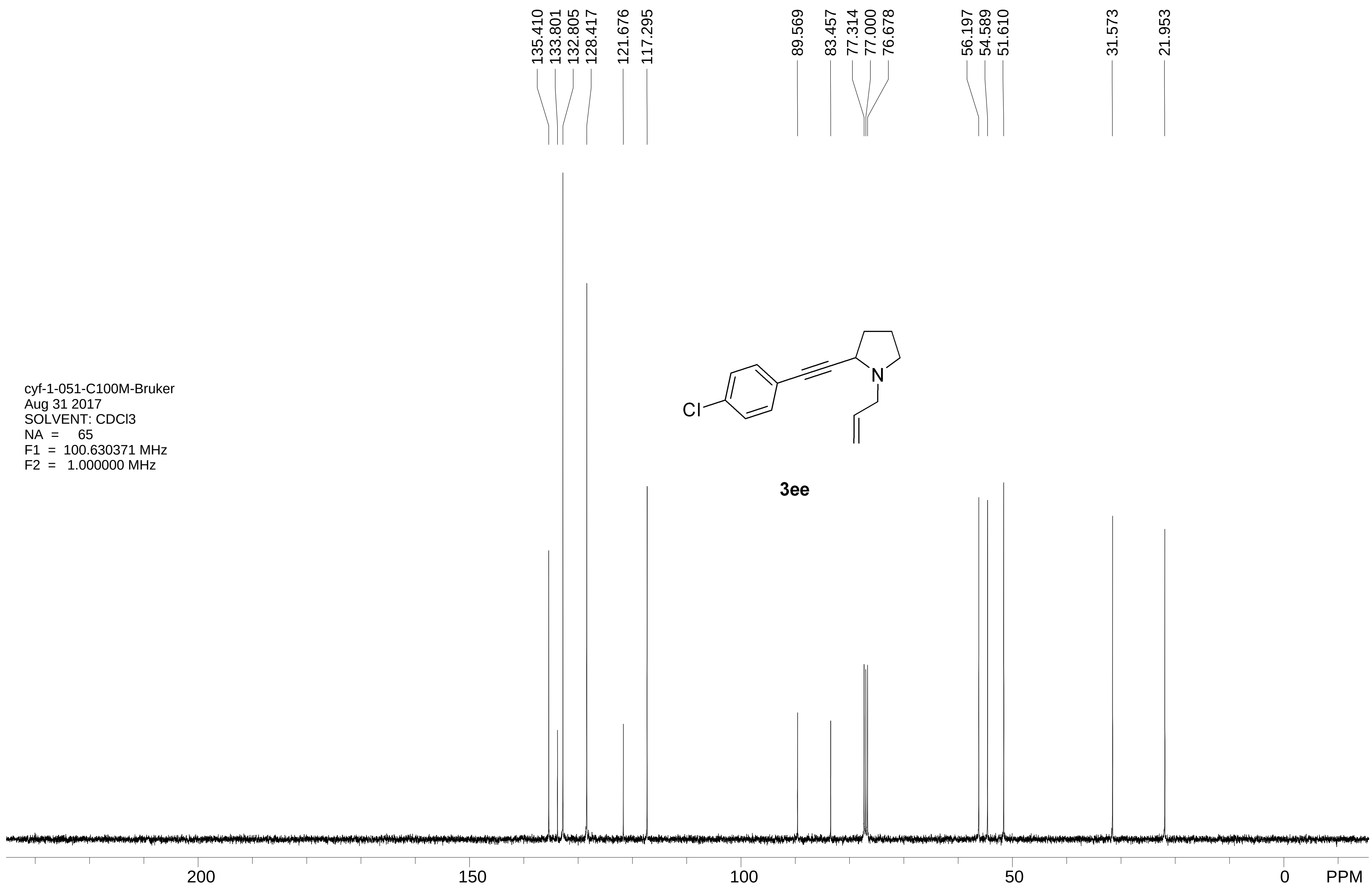
135.410
133.801
132.805
128.417
121.676
117.295
89.569
83.457
77.314
77.000
76.678
56.197
54.589
51.610
31.573
21.953
cyf-1-051-C100M-Bruker
Aug 31 2017
SOLVENT: CDCl3
NA = 65
F1 = 100.630371 MHz
F2 = 1.000000 MHz
Cl
N
3ee
200
150
100
50
0
PPM

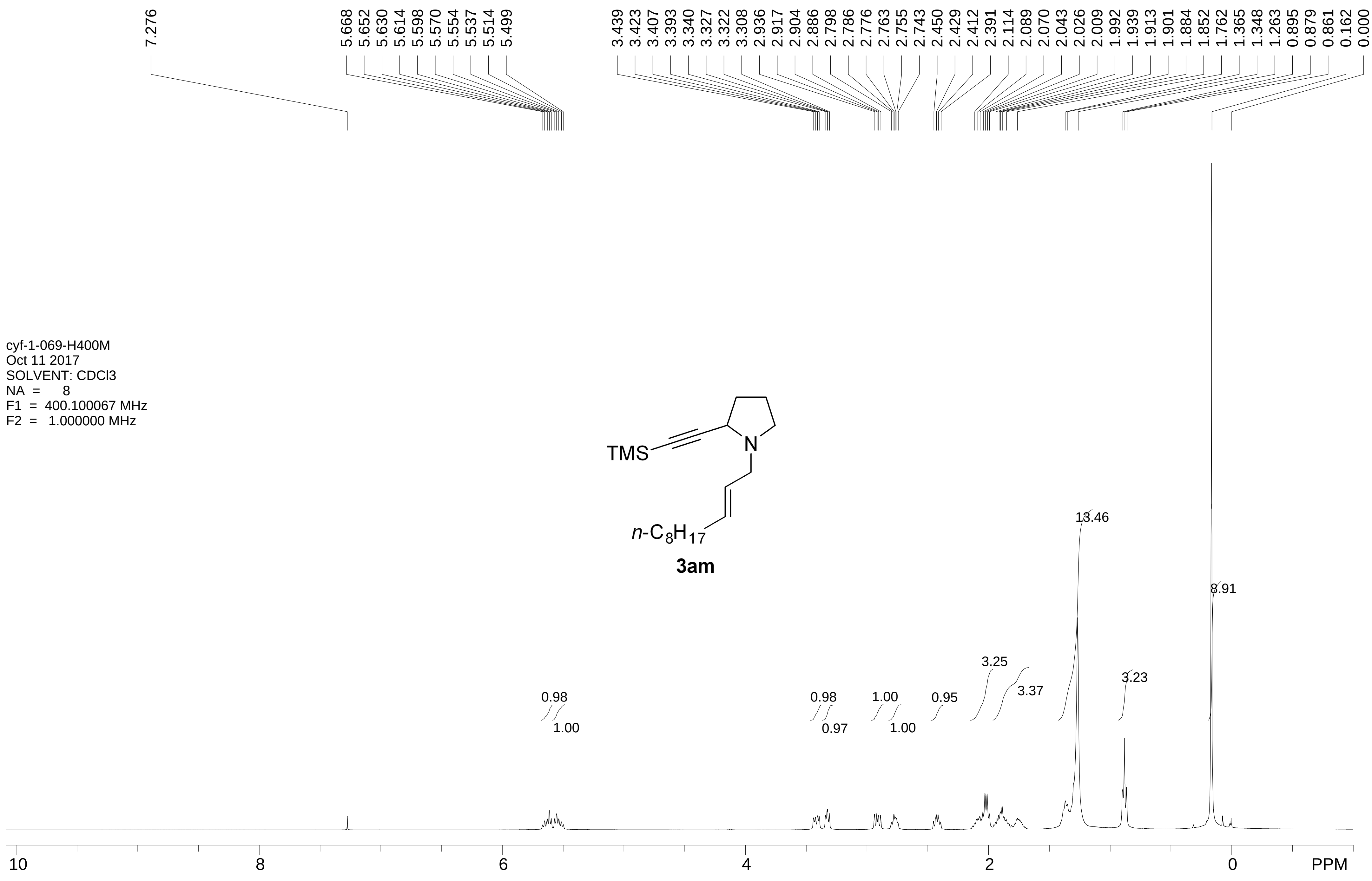
7.276
5.668 5.652 5.630 5.614 5.598 5.570 5.554 5.537 5.514 5.499
3.439 3.423 3.407 3.393 3.340 3.327 3.322 3.308 2.936 2.917 2.904 2.886 2.798 2.786 2.776 2.763 2.755 2.743 2.450 2.429 2.412 2.391 2.114 2.089 2.070 2.043 2.026 2.009 1.992 1.939 1.913 1.901 1.884 1.852 1.762 1.365 1.348 1.263 0.895 0.879 0.861 0.162 0.000
cyf-1-069-H400M
Oct 11 2017
SOLVENT: CDCl3
NA = 8
F1 = 400.100067 MHz
F2 = 1.000000 MHz
TMS
N
n-C8H17
3am
0.98
1.00
0.98
0.97
1.00
1.00
0.95
3.25
3.37
13.46
3.23
8.91
10
8
6
4
2
0
PPM

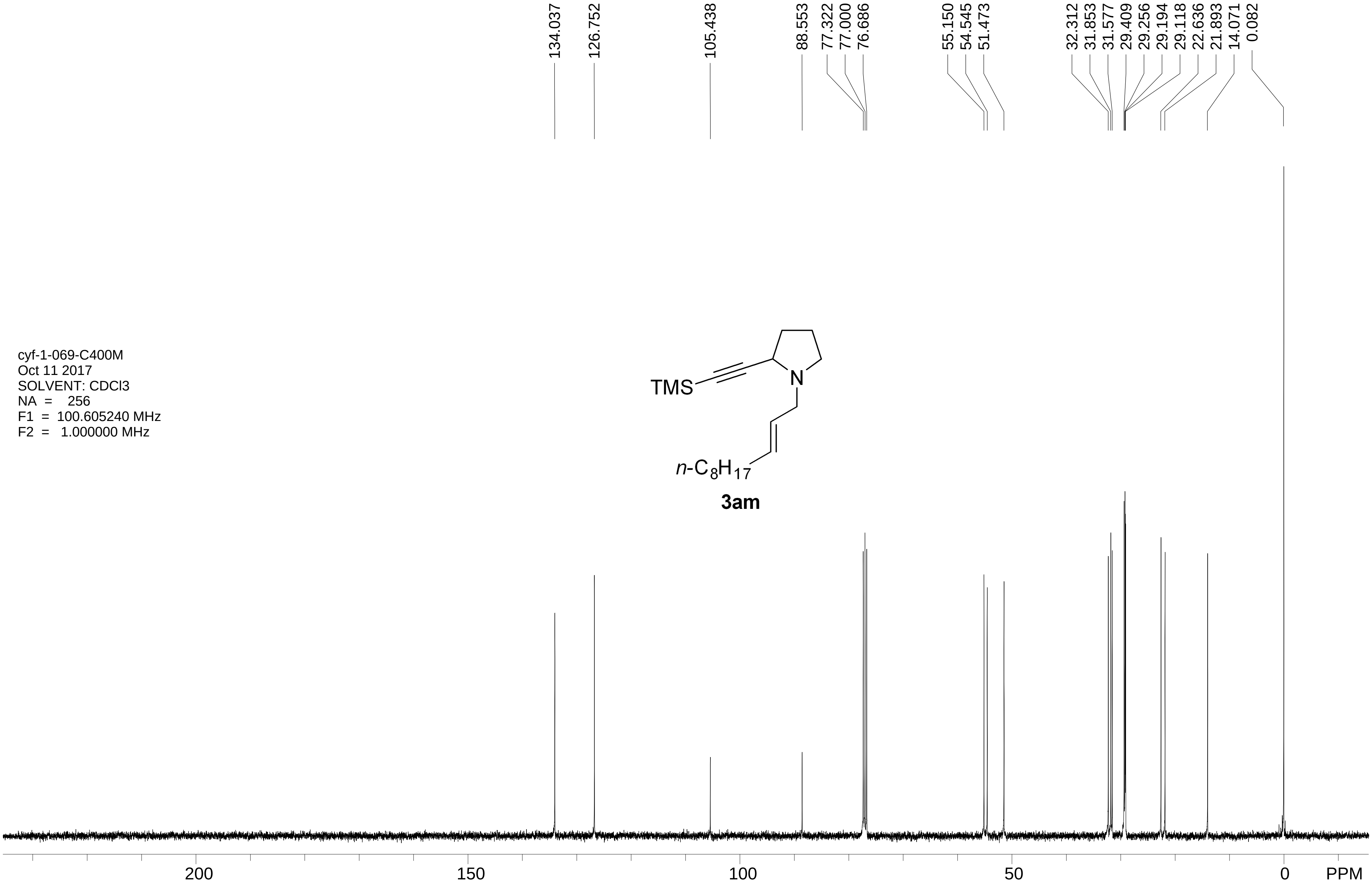
134.037
126.752
105.438
88.553
77.322
77.000
76.686
55.150
54.545
51.473
32.312
31.853
31.577
29.409
29.256
29.194
29.118
22.636
21.893
14.071
0.082
cyf-1-069-C400M
Oct 11 2017
SOLVENT: CDCl3
NA = 256
F1 = 100.605240 MHz
F2 = 1.000000 MHz
TMS
N
n-C8H17
3am
200
150
100
50
0
PPM

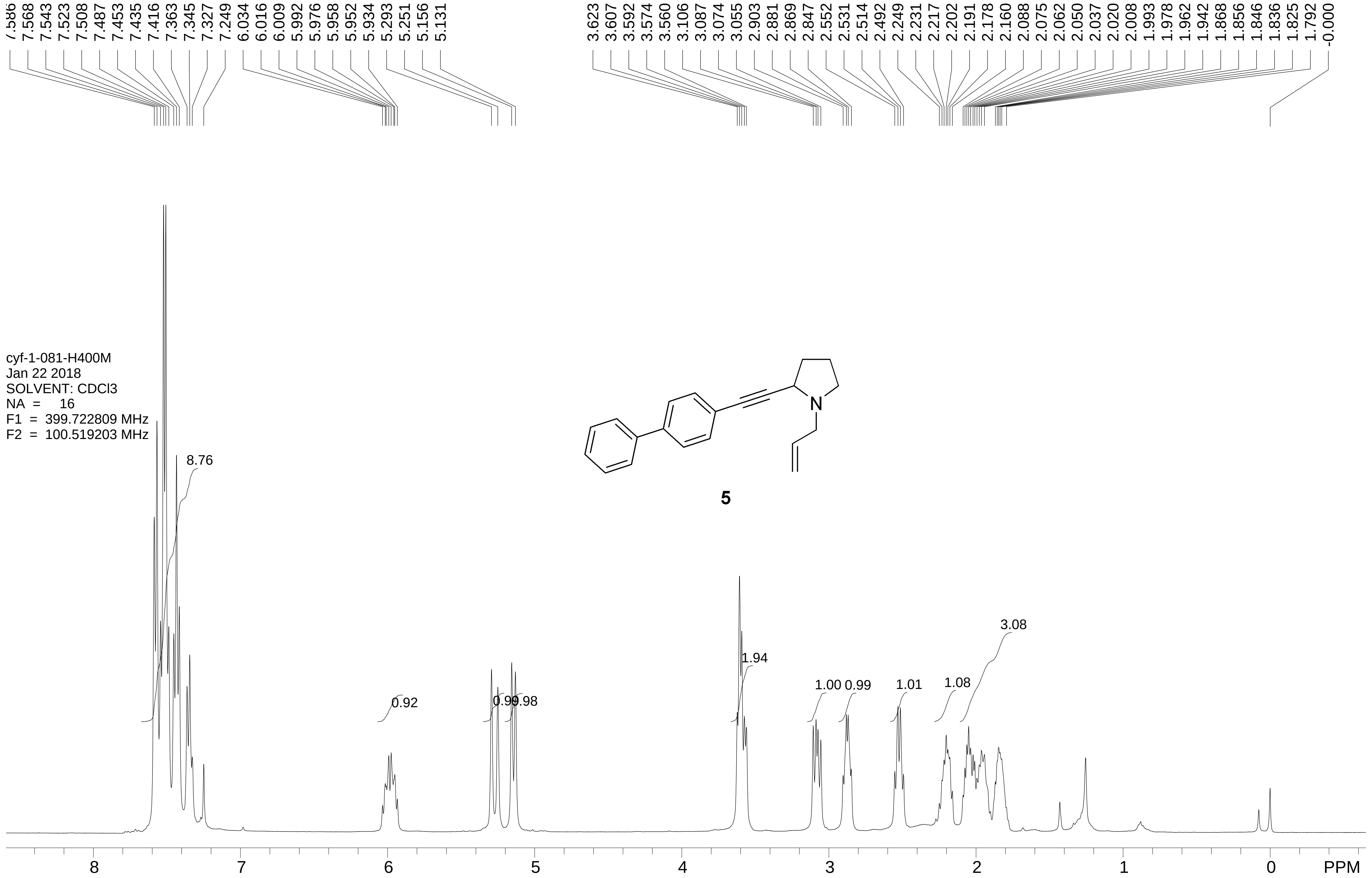
7.586 7.568 7.543 7.523 7.508 7.487 7.453 7.435 7.416 7.363 7.345 7.327 7.249 6.034 6.016 6.009 5.992 5.976 5.958 5.952 5.934 5.293 5.251 5.156 5.131
3.623 3.607 3.592 3.574 3.560 3.106 3.087 3.074 3.055 2.903 2.881 2.869 2.847 2.552 2.531 2.514 2.492 2.249 2.231 2.217 2.202 2.191 2.178 2.160 2.088 2.075 2.062 2.050 2.037 2.020 2.008 1.993 1.978 1.962 1.942 1.868 1.856 1.846 1.836 1.825 1.792 -0.000
cyf-1-081-H400M
Jan 22 2018
SOLVENT: CDCl3
NA = 16
F1 = 399.722809 MHz
F2 = 100.519203 MHz
N
5
8.76
0.92
0.99
0.98
1.94
1.00
0.99
1.01
1.08
3.08
8 7 6 5 4 3 2 1 0 PPM

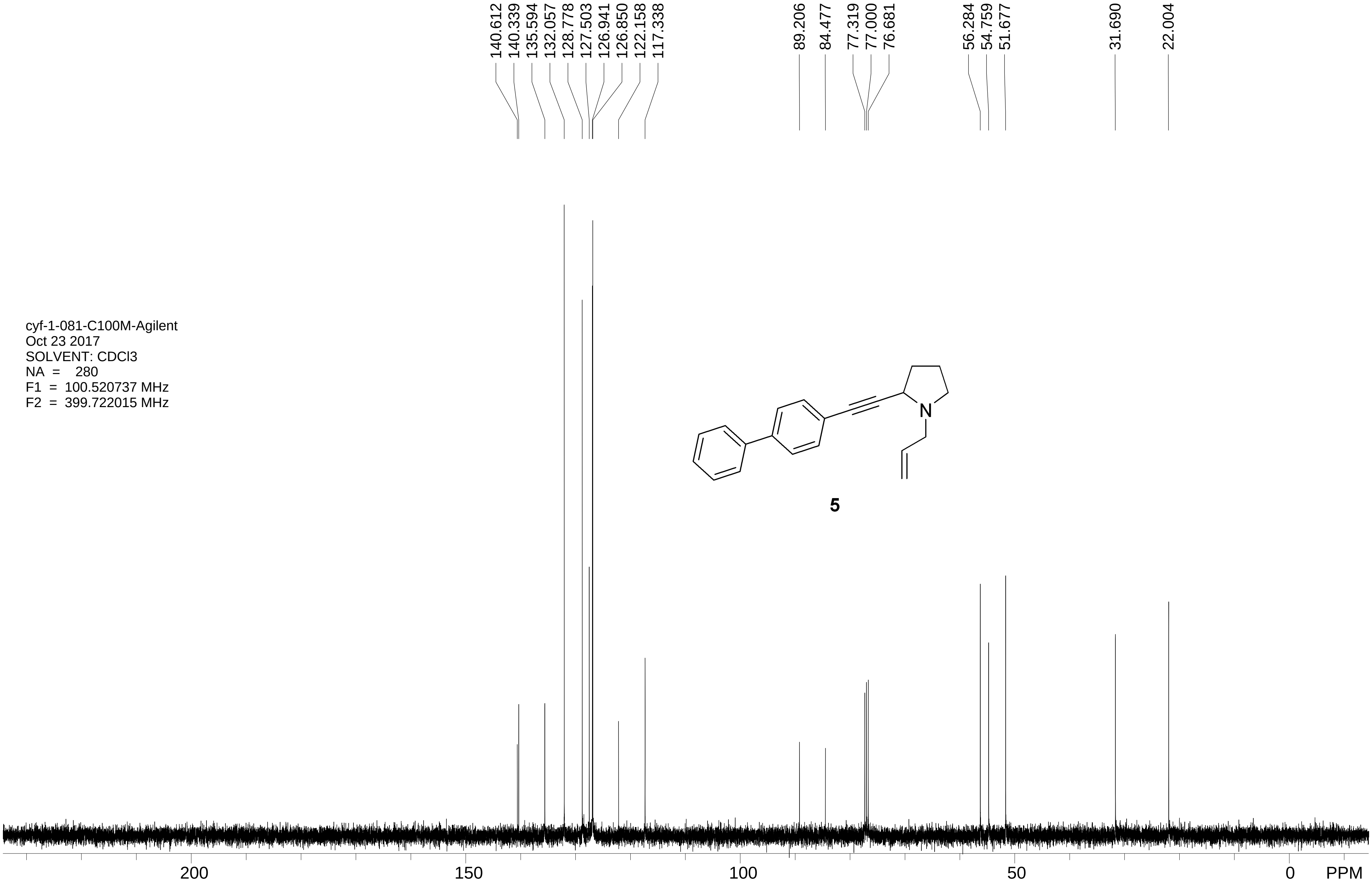
140.612
140.339
135.594
132.057
128.778
127.503
126.941
126.850
122.158
117.338
89.206
84.477
77.319
77.000
76.681
56.284
54.759
51.677
31.690
22.004
cyf-1-081-C100M-Agilent
Oct 23 2017
SOLVENT: CDCl3
NA = 280
F1 = 100.520737 MHz
F2 = 399.722015 MHz
N
5
200
150
100
50
0
PPM

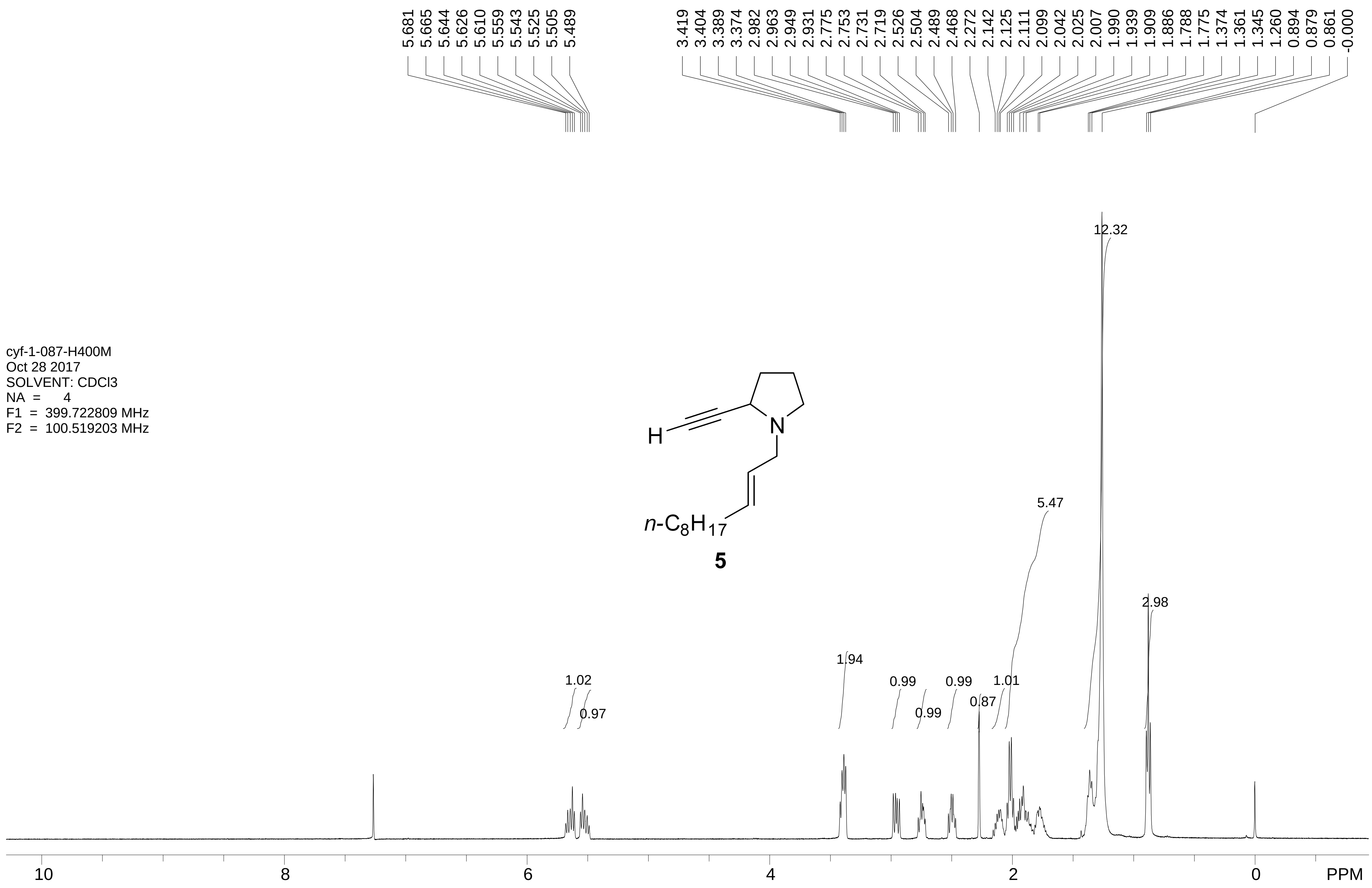
5.681 5.665 5.644 5.626 5.610 5.559 5.543 5.525 5.505 5.489
3.419 3.404 3.389 3.374 2.982 2.963 2.949 2.931 2.775 2.753 2.731 2.719 2.526 2.504 2.489 2.468 2.272 2.142 2.125 2.111 2.099 2.042 2.025 2.007 1.990 1.939 1.909 1.886 1.788 1.775 1.374 1.361 1.345 1.260 0.894 0.879 0.861 -0.000
cyf-1-087-H400M
Oct 28 2017
SOLVENT: CDCl3
NA = 4
F1 = 399.722809 MHz
F2 = 100.519203 MHz
H
N
n-C8H17
5
12.32
5.47
2.98
1.94
1.02
0.97
0.99
0.99
0.99
0.99
0.87
1.01
10
8
6
4
2
0
PPM

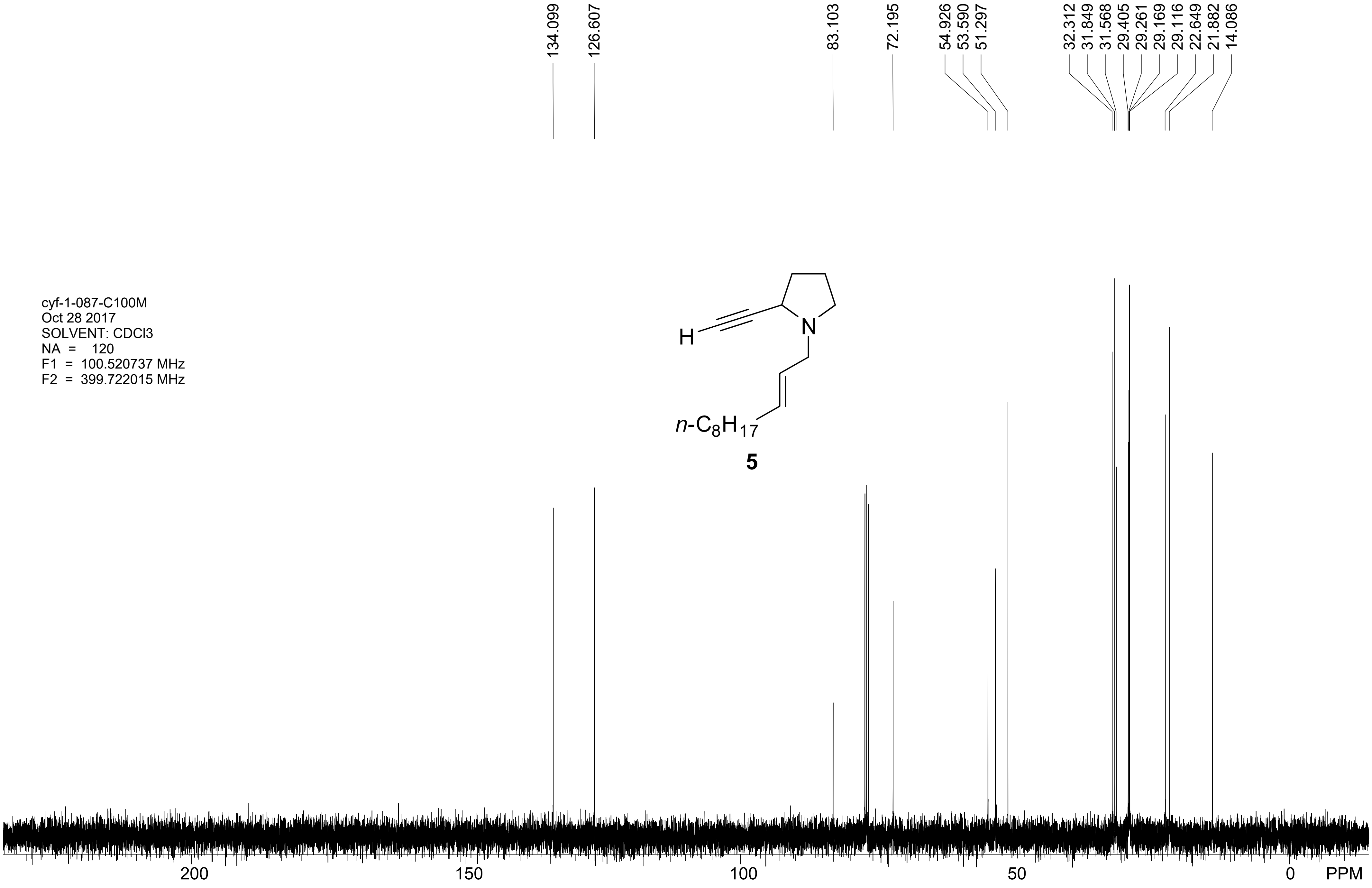
134.099
126.607
83.103
72.195
54.926
53.590
51.297
32.312
31.849
31.568
29.405
29.261
29.169
29.116
22.649
21.882
14.086
cyf-1-087-C100M
Oct 28 2017
SOLVENT: CDCl3
NA = 120
F1 = 100.520737 MHz
F2 = 399.722015 MHz
H
N
n-C8H17
5
200
150
100
50
0
PPM

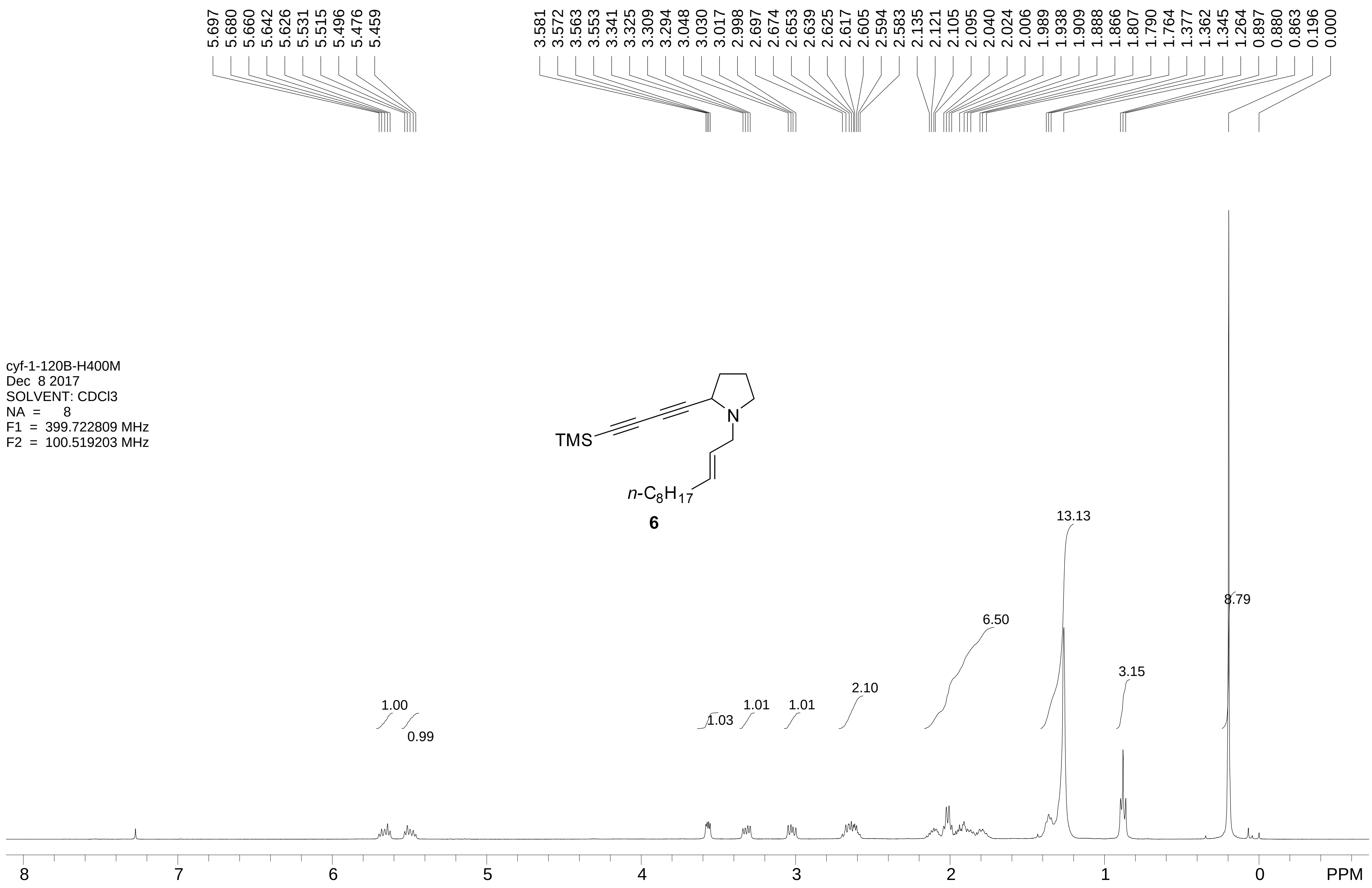
5.697 5.680 5.660 5.642 5.626 5.531 5.515 5.496 5.476 5.459
3.581 3.572 3.563 3.553 3.341 3.325 3.309 3.294 3.048 3.030 3.017 2.998 2.697 2.674 2.653 2.639 2.625 2.617 2.605 2.594 2.583 2.135 2.121 2.105 2.095 2.040 2.024 2.006 1.989 1.938 1.909 1.888 1.866 1.807 1.790 1.764 1.377 1.362 1.345 1.264 0.897 0.880 0.863 0.196 0.000
cyf-1-120B-H400M
Dec 8 2017
SOLVENT: CDCl3
NA = 8
F1 = 399.722809 MHz
F2 = 100.519203 MHz
TMS
N
n-C8H17
6
13.13
8.79
6.50
3.15
2.10
1.00
0.99
1.03
1.01
1.01
8
7
6
5
4
3
2
1
0
PPM

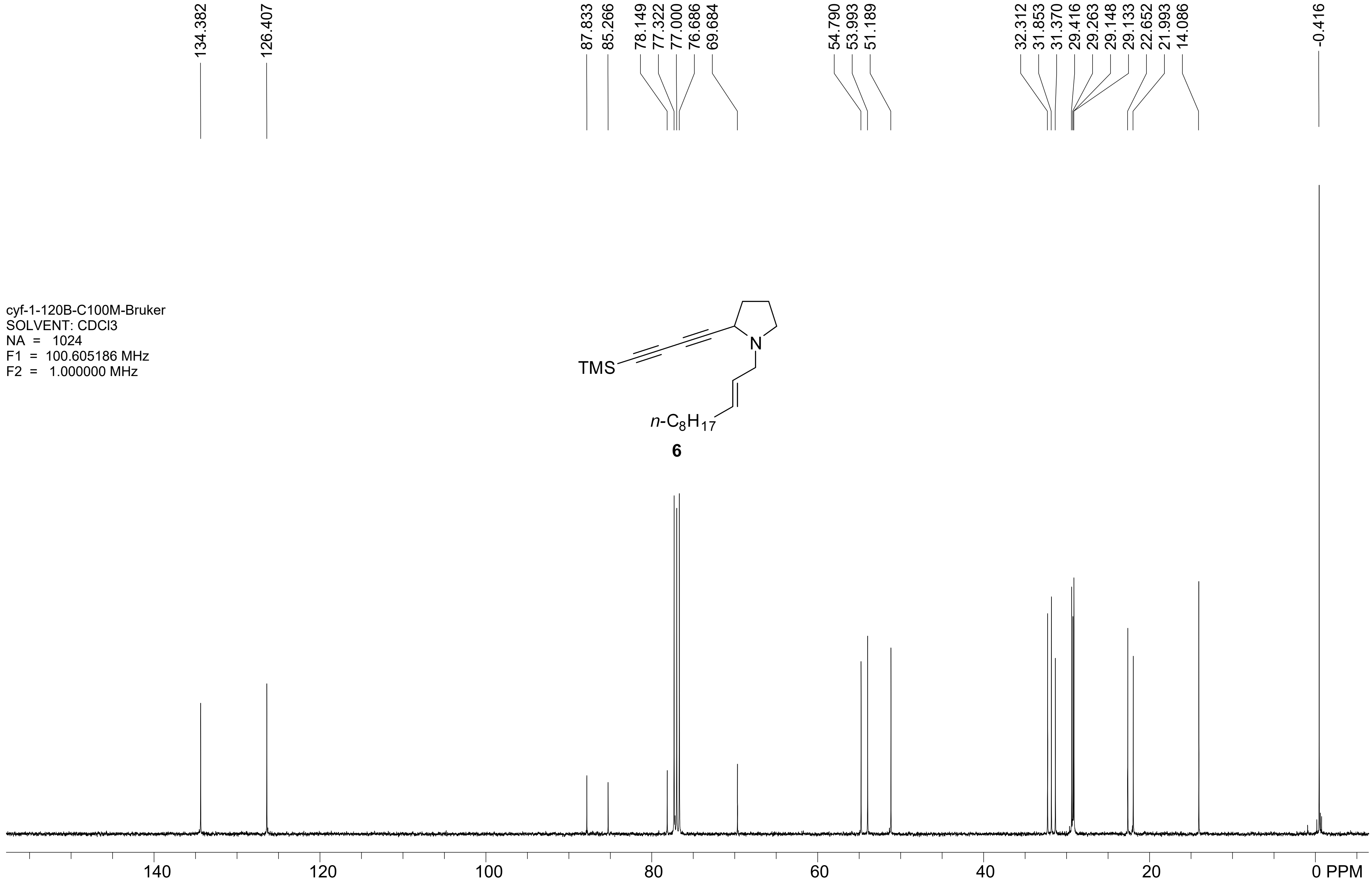
134.382
126.407
87.833
85.266
78.149
77.322
77.000
76.686
69.684
54.790
53.993
51.189
32.312
31.853
31.370
29.416
29.263
29.148
29.133
22.652
21.993
14.086
-0.416
cyf-1-120B-C100M-Bruker
SOLVENT: CDCl3
NA = 1024
F1 = 100.605186 MHz
F2 = 1.000000 MHz
TMS
N
n-C8H17
6
140
120
100
80
60
40
20
0 PPM

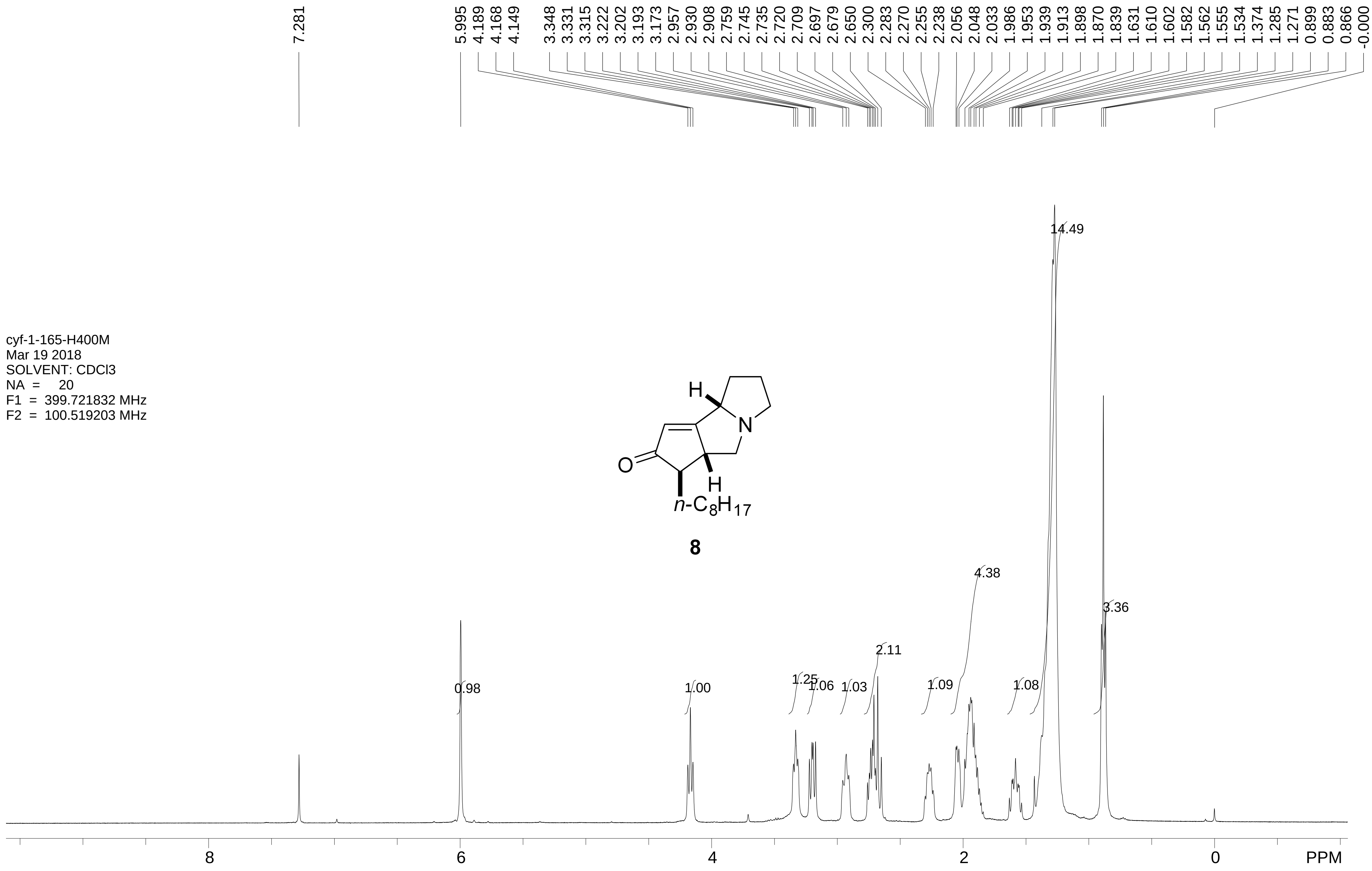
7.281
5.995
4.189
4.168
4.149
3.348
3.331
3.315
3.222
3.202
3.193
3.173
2.957
2.930
2.908
2.759
2.745
2.735
2.720
2.709
2.697
2.679
2.650
2.300
2.283
2.270
2.255
2.238
2.056
2.048
2.033
1.986
1.953
1.939
1.913
1.898
1.870
1.839
1.631
1.610
1.602
1.582
1.562
1.555
1.534
1.374
1.285
1.271
0.899
0.883
0.866
-0.000
cyf-1-165-H400M
Mar 19 2018
SOLVENT: CDCl3
NA = 20
F1 = 399.721832 MHz
F2 = 100.519203 MHz
H
N
O
H
n-C8H17
8
14.49
4.38
3.36
2.11
0.98
1.00
1.25
1.06
1.03
1.09
1.08
8
6
4
2
0
PPM

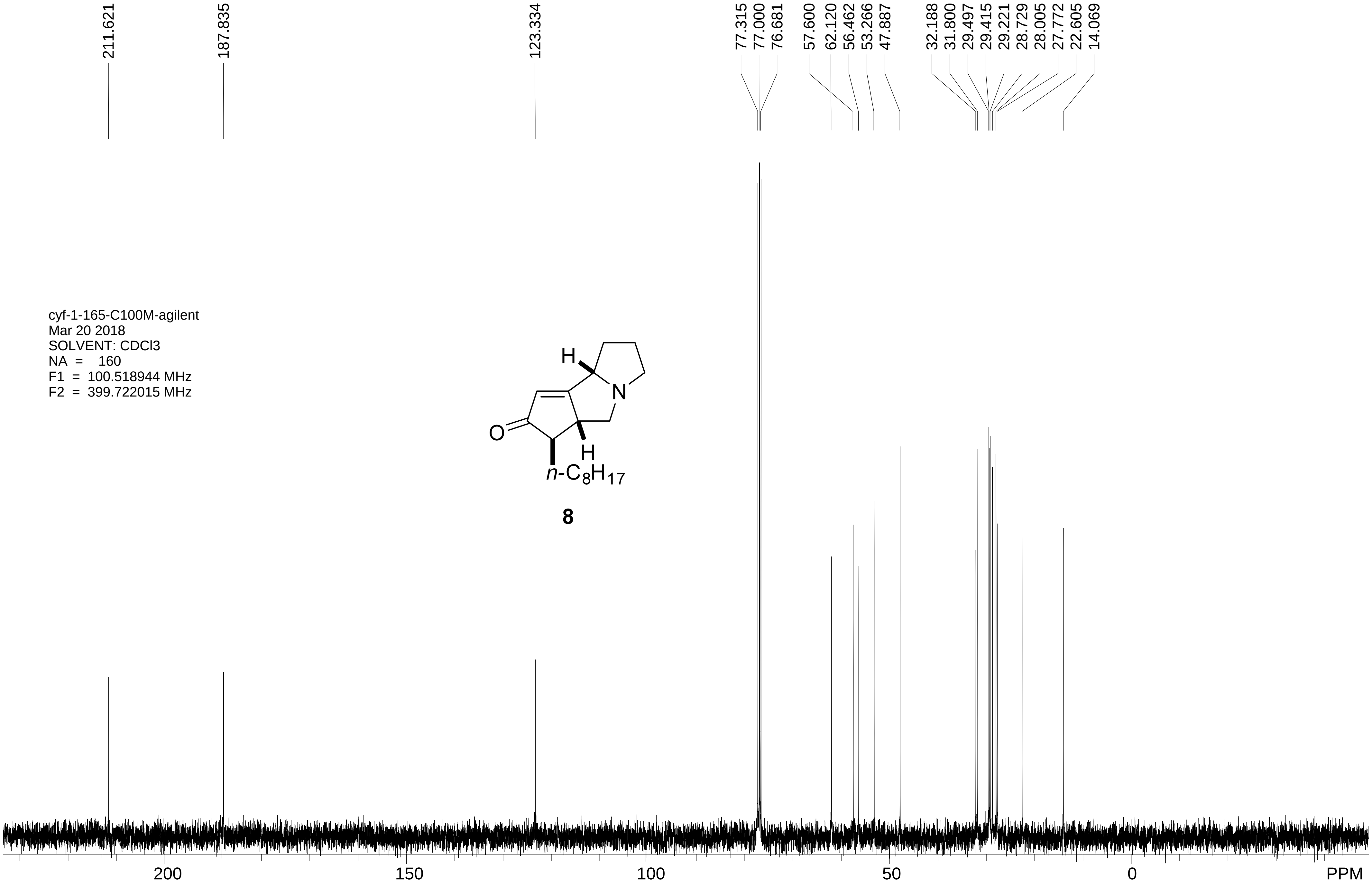
211.621
187.835
123.334
77.315
77.000
76.681
57.600
62.120
56.462
53.266
47.887
32.188
31.800
29.497
29.415
29.221
28.729
28.005
27.772
22.605
14.069
cyf-1-165-C100M-agilent
Mar 20 2018
SOLVENT: CDCl3
NA = 160
F1 = 100.518944 MHz
F2 = 399.722015 MHz
H
N
O
H
n-C8H17
8
200
150
100
50
0
PPM
